# The role of attachment type and bone height in modulating stress distribution in mandibular overdentures: Insights from finite element analysis

**DOI:** 10.1371/journal.pone.0351498

**Published:** 2026-06-16

**Authors:** Burç İhsan Gencel, Melahat Çelik Güven, Uğur Mercan, Süleyman Çağatay Dayan, Onur Geçkili

**Affiliations:** 1 Program of Dental Technician, Istanbul University-Cerrahpaşa, Istanbul, Türkiye; 2 Private Practice, Mersin, Türkiye; 3 University of Istanbul, Faculty of Dentistry, Department of Prosthodontics, Istanbul, Türkiye; International Medical University, MALAYSIA

## Abstract

**Background:**

Mandibular two-implant overdentures are considered the standard of care for edentulous patients. The attachment system and the shape of the bone crest can significantly influence biomechanical behaviours. This study aimed to evaluate stress distribution in mandibular implant-supported overdentures using locator and bar attachments across various bone crest designs.

**Materials and methods:**

FEA was performed on mandibular overdentures supported by two implants. Three crest configurations (flat, convex, and irregular) were modelled with either bar or locator attachments. A vertical force of 100 N was applied in three loading conditions: anterior, unilateral molar, and bilateral molar. Maximum von Mises stresses and principal stresses were assessed.

**Results:**

Anterior loading resulted in the highest stresses across all groups, with peak values reaching 12 N/mm² in the convex and irregular models. Bilateral molar loading consistently produced the lowest and most uniform stress distributions, while unilateral loading caused intermediate stress concentrations on the working side. Uneven platforms significantly increased stress levels, particularly under unilateral bar loading. Locator attachments showed slightly reduced stresses after bilateral loading in irregular crest configurations. Stress concentrations were primarily localized at the implant neck and prosthetic connectors.

**Conclusion:**

Stress distribution in mandibular overdentures is significantly influenced by loading direction, crest shape, and attachment mechanism. Anterior loading poses the highest biomechanical risk, while bilateral posterior loading provides optimal conditions. Vertical discrepancies in implant platforms heighten stress concentrations, underscoring the importance of careful surgical planning. Locator attachments offer limited biomechanical advantages in uneven crest scenarios, supporting their use in anatomically challenging cases.

## Background

In prosthetic dentistry, complete dentures have long been the standard therapy for patients who are completely edentulous [1]. However, the instability and retention issues associated with these prostheses frequently diminish patient satisfaction, particularly in mandibular complete dentures, despite being fabricated under optimal conditions [2]. The introduction of implant-supported overdentures has revolutionized dental practice by allowing patients to regain essential oral functions such as chewing and speaking, while also addressing aesthetic concerns. Mandibular two-implant overdentures have emerged as the minimum standard of care for patients who are completely edentulous [3,4].

The biomechanical behavior of these overdentures is influenced by a myriad of factors, including the type of attachment system employed and the morphology of the supporting bone crest [5]. Common attachment systems include locator and bar attachments, each offering distinct advantages and challenges in terms of retention and ease of use [6]. For instance, locator attachments provide a simpler and more cost-effective solution, while bar attachments may offer enhanced stability and improved load distribution across the implants [7].

Furthermore, the design of the supporting bone crest—characterized as flat, convex, or irregular—plays a significant role in the stress distribution experienced by both the implants and the surrounding bone structure [8]. The morphology of the bone crest can lead to varied stress patterns during functional loading, which may ultimately impact the longevity and success of the implants. Uneven stress distribution can lead to localized bone resorption, implant failure, or complications that jeopardize the overall treatment outcome [5,8–10].

Given these considerations, it is critical to understand how different loading conditions and attachment systems affect the biomechanical performance of mandibular implant-supported overdentures [11–13]. This study aims to evaluate the stress distribution in mandibular overdentures supported by locator and bar attachments across various bone crest shapes. By employing three-dimensional finite element analysis (FEA), we will simulate the mechanical behavior of these systems under functional loads, providing invaluable insights into their performance.

The findings of this research will contribute to the existing body of knowledge regarding implant-supported prosthetics, guiding clinicians in making informed decisions tailored to each patient’s anatomical and functional needs. Ultimately, this study seeks to enhance the clinical outcomes for edentulous patients, ensuring they receive the most effective and durable solutions for their dental rehabilitation.

## Materials and methods

### Model development

A three-dimensional finite element model of an edentulous human mandible was generated from a cone-beam computed tomography (CBCT) dataset (3M Iluma CBCT; 3M, St. Paul, MN, USA; 120 kVp, 3.8 mA, 40 s). Ethical approval for this study was obtained from the Ethics Committee of Istanbul University, Faculty of Medicine (Protocol No. 2018/85). The medical record was accessed for research purposes on 15/12/2018. All data were fully anonymized before access, and the requirement for informed consent was waived by the Ethics Committee. Segmentation was performed using 3D-DOCTOR software (Able Software Corp., Lexington, MA, USA) to isolate cortical and trabecular bone. Overdentures and the supporting soft tissue are incorporated into the model as well. The mucosal thickness is assumed to be constant and utilized in experimental in vitro research [14,15]; hence, potential alterations in denture movement around the fulcrum due to the resilience of the posterior mucosa are overlooked. The mucosal thickness of 1.5 mm around the cortical bone thickness of 2 mm is delineated around the cancellous core.

### Implant and attachments

Dental implants featuring internal conical connections (NobelActive Regular Platform [RP] Nobel Biocare, Yorba Linda, CA, USA) and abutments were longitudinally sectioned utilizing a saw machine (Isomet5000; Buehler, Lake Bluff, IL, USA) and subsequently scanned with an Activity 880 optical scanner (Smart Optics Sensortechnik GmbH, Bochum, Germany) with a precision of 10 mm, and the data were reconstructed in VRMesh Studio (VirtualGrid Inc., Redmond, WA, USA). Dental implants and prosthetic components were designed utilizing Rhinoceros 4.0 software (Robert McNeel & Associates, Seattle, WA, USA). Dental implants (12.00 mm x 4.3 mm diameter) were positioned anteriorly in the right and left lateral regions. Two attachment types were modeled: (i) bar and (ii) locator attachment. Components were aligned on the mandibular models using Rhinoceros 4.0 and integrated via Boolean operations to ensure precise interfaces at implant–abutment, attachment–housing, and bar–clip contacts.

### Crest configurations

Three interimplant bone configurations were simulated:

Group 1: Flat alveolar crest – implants positioned at the same vertical level.
Group 2: Convex alveolar crest (dome-shaped).
Group 3: Irregular alveolar crest – characterized by vertically misaligned implant platforms.

### Mesh generation and refinement

Mesh generation was carried out using VRMesh Studio (VirtualGrid Inc., USA) and Algor Fempro (ALGOR Inc., USA). Eight-node hexahedral elements were used predominantly, with tetrahedral and reduced-node elements applied in geometrically complex regions. Special refinement was applied at implant–bone interfaces and attachment components. Each model consisted of ~141,000–150,000 nodes and ~736,000–748,000 elements. A mesh convergence test was performed to confirm that further refinement resulted in <5% variation in maximum stress values, consistent with established FEA protocols [16].

### Material properties

All materials were considered isotropic, homogeneous, and linearly elastic. Elastic properties (Young’s modulus [MPa]; Poisson’s ratio) were defined as follows: cortical bone (13,700; 0.30), trabecular bone (1,370; 0.30), titanium (110,000; 0.30), PMMA denture base (3,000; 0.35), retentive matrix (4,000; 0.37), clip (100,000; 0.30), and mucosa (10,000; 0.40). These values were adopted from previously validated biomechanical studies [9,17].

### Boundary conditions and loading

The basal surface of the mandible was fully constrained in all degrees of freedom. A static vertical load of 100 N was applied under three different loading scenarios:

1. Anterior loading at the incisal edge.
2. Unilateral molar loading at the occlusal surface of the first molar.
3. Bilateral molar loading at both first molars simultaneously.

The load was distributed across the occlusal contact surfaces to simulate clinical masticatory function. Complete osseointegration was assumed at the implant–bone interface, while prosthetic interfaces were modeled as fully bonded contacts.

### Validation

The finite element models were validated through mesh convergence testing and comparison of stress patterns with previously published experimental and computational data [5,17]. Stress concentrations at the crestal cortical bone and implant neck observed in the present study were consistent with those reported in validated FEA and strain gauge studies, supporting the reliability of the model.

### Outcome measures

Primary outcomes were maximum and minimum principal stresses in cortical and cancellous bone adjacent to implants. Secondary outcomes included von Mises stresses in implants, attachments, and overdenture prostheses. Stress distribution patterns were illustrated using color contour plots and descriptively compared across models

## Results

### Implant stresses

Table 1 delineates the maximum von Mises stresses observed in implants subjected to various loading situations and attachment techniques. In Group 1, anterior loading produced the most stresses, attaining 7.83 N/mm² for the bar and 6.49 N/mm² for the locator. Unilateral molar loading produced considerable stresses of 2.26 N/mm² for the bar and 2.31 N/mm² for the locator, whereas bilateral molar loading resulted in the lowest values of 1.27 N/mm² and 1.33 N/mm², respectively. In Group 2 (convex crest), anterior loading yielded the highest stress values, measuring 11.92 N/mm² for the bar and 11.59 N/mm² for the locator. Unilateral molar loading produced reduced stresses (1.70 N/mm² and 1.66 N/mm²), whereas bilateral molar loading yielded the most advantageous distribution (1.19 N/mm² and 1.15 N/mm²). In Group 3 (irregular crest), anterior loading resulted in elevated stresses of 10.20 N/mm² for the bar and 11.41 N/mm² for the locator. Unilateral molar loading generated intermediate stresses of 2.45 N/mm² and 2.51 N/mm², whereas bilateral molar loading diminished stress levels, however they remained elevated compared to Groups 1 and 2, with values of 2.67 N/mm² for the bar and 2.80 N/mm² for the locator.

**Table 1:** Maximum von mises stress of the overdenture and implants.

Group	Load	Overdenture – Bar (N/mm²)	Overdenture – Locator (N/mm²)	Implant – Bar (N/mm²)	Implant – Locator (N/mm²)
**Group 1**	**Anterior**	7.71	8.10	7.83	6.49
**Group 1**	**Bilateral molars**	1.45	1.81	1.27	1.33
**Group 1**	**Unilateral molar**	2.91	3.62	2.26	2.31
**Group 2**	**Anterior**	7.72	8.10	11.92	11.59
**Group 2**	**Bilateral molars**	1.45	1.81	1.19	1.15
**Group 2**	**Unilateral molar**	2.91	3.62	1.70	1.66
**Group 3**	**Anterior**	8.27	8.73	10.20	11.41
**Group 3**	**Bilateral molars**	6.04	1.67	2.67	2.80
**Group 3**	**Unilateral molar**	12.11	3.05	2.45	2.51

### Stresses in overdentures

In Group 1, anterior loading produced the maximum prosthetic stresses (7.71 N/mm² for the bar; 8.10 N/mm² for the locator). Bilateral molar loading resulted in the lowest values (1.45 N/mm² and 1.81 N/mm²), whilst unilateral loading yielded intermediate levels (2.91 N/mm² and 3.62 N/mm²). In Group 2, a same trend was noted, with anterior loading yielding high stresses (7.72 N/mm² bar; 8.10 N/mm² locator), bilateral loading resulting in the lowest values (1.45 and 1.81 N/mm²), and unilateral loading generating intermediate stresses (2.91 and 3.62 N/mm²). In Group 3, the overall stress magnitudes were elevated. The peak prosthetic stress recorded in the study (12.11 N/mm²) was observed during unilateral molar loading with the bar attachment. Anterior loading generated stresses of 8.27 N/mm² (bar) and 8.73 N/mm² (locator), whereas bilateral molar loading reduced strains to 6.04 N/mm² for the bar and 1.67 N/mm² for the locator.

### Stresses in cortical and trabecular bone

**Fig 1:**
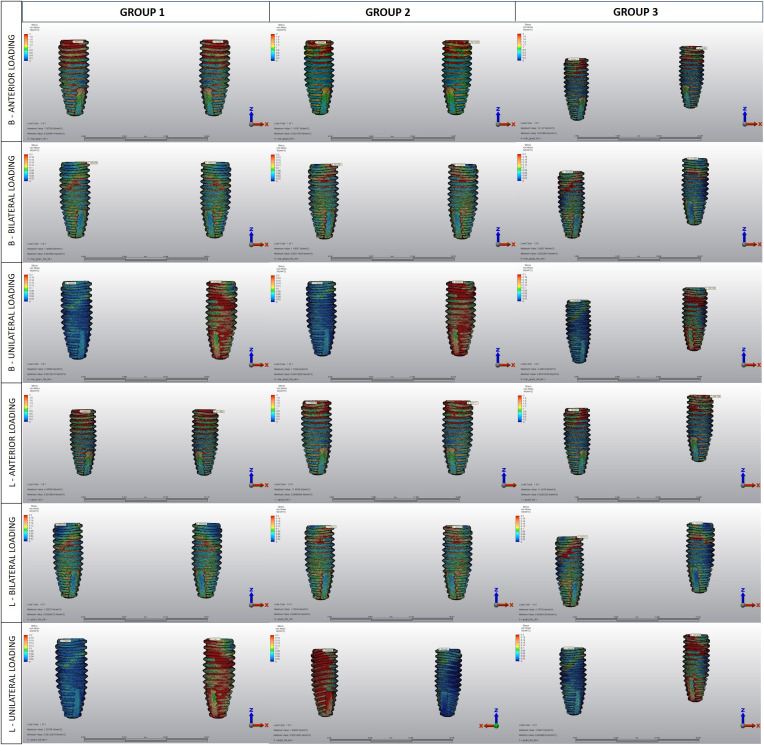
The stress distribution on the implants for both locator and bar attachment groups in the three clinical scenarios.

**Fig 2:**
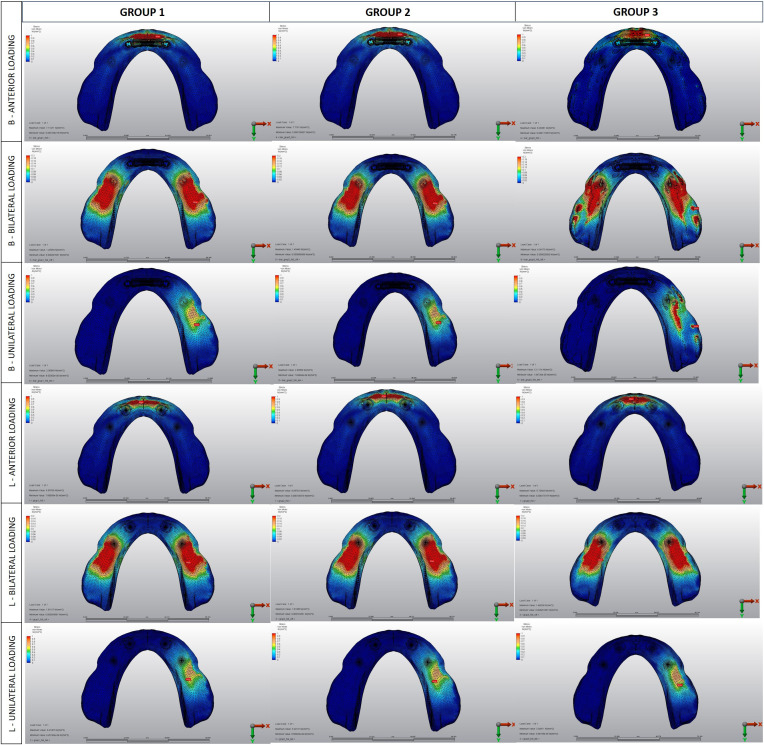
The stress distribution on the prosthesis in all groups.

**Fig 3:**
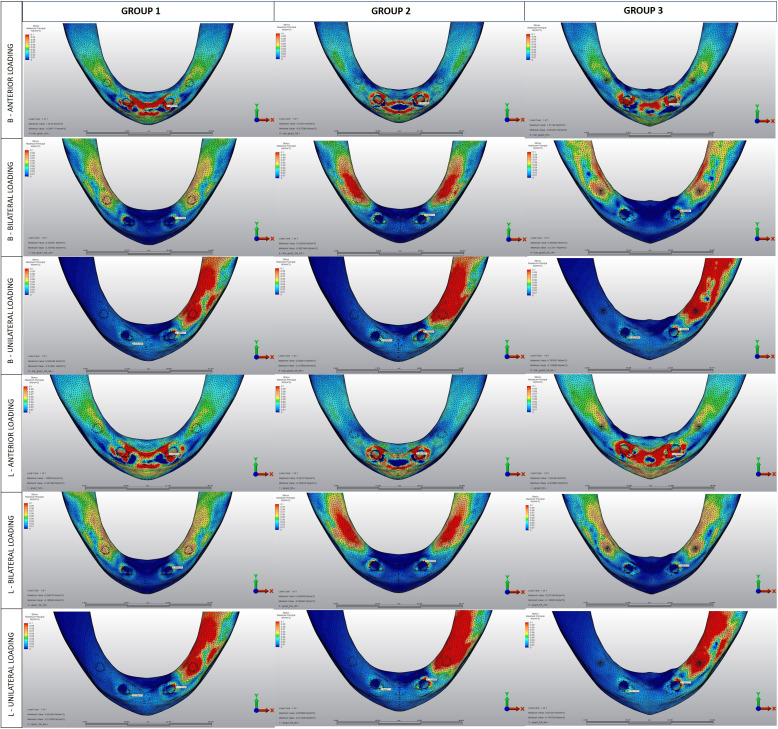
Maximum principal stress values in the cortical bone.

**Fig 4:**
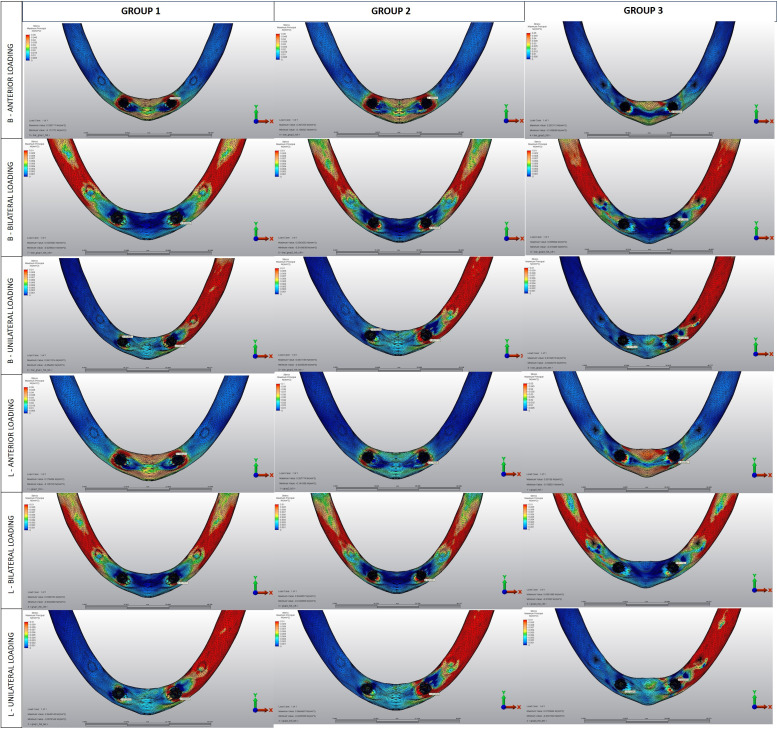
Maximum principal stress values in the trabecular bone.

Finite element analysis of maximal primary stresses (σ_max) revealed that cortical bone consistently endured greater stress levels than cancellous bone in all groups. Overall, anterior loading generated the highest stresses, unilateral loading provided intermediate values, and bilateral loading resulted in the lowest stresses (Table 2). In Group 1, cortical stresses in the bar-retained model were measured at 1.25 MPa during anterior loading, 0.66 MPa during unilateral loading, and 0.33 MPa during bilateral loading. The stresses in cancellous bone were 0.33 MPa, 0.06 MPa, and 0.03 MPa, respectively. The locator-retained model exhibited similar trends, with cortical stresses measuring 1.09, 0.66, and 0.33 MPa, and cancellous stresses of 0.28, 0.06, and 0.03 MPa. In Group 2, anterior loading generated the greatest cortical tensions observed in the study. The bar-retained model had a strength of 2.51 MPa, but the locator-retained model attained 3.29 MPa. Unilateral loading diminished cortical stresses to 0.68 MPa (bar) and 0.63 MPa (locator), while bilateral loading further lowered them to 0.32 MPa and 0.35 MPa, respectively. The stresses in cancellous bone were significantly lower but maintained the same sequence: 0.29, 0.07, and 0.03 MPa for the bar; 0.27, 0.07, and 0.03 MPa for the locator. In Group 3, the bar-retained model demonstrated cortical stresses of 1.22 MPa (anterior), 0.79 MPa (unilateral), and 0.39 MPa (bilateral), with cancellous values of 0.28, 0.07, and 0.06 MPa. The locator-retained model exhibited cortical stresses of 1.42, 0.16, and 0.33 MPa, and cancellous stresses of 0.30, 0.07, and 0.06 MPa for anterior, unilateral, and bilateral loading, respectively (Figs 1–4).

**Table 2:** Maximum principal stresses in cortical and trabecular bone.

	Bar Group 1	Locator Group 1	Bar Group 2	Locator Group 2	Bar Group 3	Locator Group 3
**Anterior – Cortical**	1.251600	1.090590	2.513510	3.291970	1.217260	1.422480
**Anterior – Trabecular**	0.330717	0.278456	0.287253	0.267774	0.282113	0.301360
**Bilateral – Cortical**	0.332501	0.334673	0.322456	0.349053	0.393503	0.327186
**Bilateral – Trabecular**	0.029355	0.029873	0.034200	0.034937	0.055984	0.055189
**Unilateral -Cortical**	0.660244	0.663246	0.684416	0.625938	0.787697	0.156100
**Unilateral -Trabecular**	0.061232	0.064814	0.067215	0.066469	0.072697	0.072904

## Discussion

The current finite element analysis demonstrates that loading direction, alveolar crest morphology, and attachment type collectively influence stress transmission in mandibular two-implant overdentures. Anterior loading consistently produced the highest strains, particularly in convex and uneven crests, with peak stress concentrated at the cervical area of the implants. This aligns with previous findings that identify the crestal bone–implant contact as the initial and most rigid constraint, serving as the primary site for stress overload [1,16,18]. Clinically, these results underscore the need to minimize significant incisal contacts through occlusal harmony and proper tooth alignment.

Unilateral molar loading resulted in intermediate stress levels with distinct laterality, supporting the previously established asymmetric load transfer pattern [19]. In contrast, bilateral posterior loading consistently generated the lowest and most uniform stress fields, indicating a beneficial load-sharing effect across the implants [20]. This finding supports the clinical objective of achieving simultaneous posterior contacts during the occlusal adjustment of mandibular overdentures to reduce localized peak stresses and improve biomechanical conditions.

The shape of the alveolar crest significantly affected stress levels. Under anterior loading, the convex crest exhibited the highest stresses among the aligned-platform groups, suggesting that this morphology increases crestal demand during incisal function [21]. Irregular crests further exacerbated stress, particularly under unilateral bar loading, where the highest peak stress at the prosthesis level was recorded. Collectively, these findings indicate that vertical disparities across implant platforms act as stress concentrators, undermining the advantages of load sharing. From a surgical perspective, precise three-dimensional implant placement to standardize platform height, along with supplementary alveoloplasty when necessary, could alleviate these issues.

The differences in attachment types between splinted bar and non-splinted locator systems were minimal under anterior and unilateral loading conditions [11]. However, under bilateral loading, locators exhibited slightly reduced stresses in scenarios with irregular crests, suggesting a minor biomechanical advantage when vertical discrepancies exist. The splinting function of the bar can redistribute forces at the prosthesis level, offering support when enhanced stability is required or when ridge shape is significantly compromised [22]. Implant-level outcomes varied by group: in the convex crest, locators generally produced lower implant stresses across conditions, while in the flat crest, variations were negligible and inconsistent [23]. Therefore, the choice of attachments should be personalized, taking into account ridge morphology, restorative space, hygiene and maintenance considerations, as well as potential platform incompatibility.

Cortical bone consistently displayed greater main stresses than cancellous bone, regardless of attachment or loading method, which is consistent with material behavior [24]. This finding highlights the risk of marginal bone remodeling when high stresses are concentrated on the crestal cortical shell, particularly in the presence of uneven platforms or significant anterior contacts. Stress concentrations at the overdenture level were localized in connector regions and occlusal contact points, especially under unilateral bar loading, emphasizing the need for adequate denture base thickness and localized reinforcements in high-stress areas to reduce fracture risk [25,26].

It is important to note that this study has inherent limitations associated with deterministic finite element analysis [9,17]. Materials were modeled as linearly elastic, homogeneous, and isotropic, while actual bone and mucosa exhibit anisotropic and viscoelastic properties. Interfaces were assumed to be fully bonded with complete osseointegration, and only static vertical stresses of 100 N were applied [9,13,27]. These assumptions may oversimplify real-life conditions and likely underestimate micromovements and temporal effects observed in vivo [17,28]. Consequently, absolute stress magnitudes should be interpreted comparatively rather than as clinical benchmarks. Future research should incorporate oblique and cyclic loading, more accurate soft tissue characteristics and contact definitions, as well as experimental and clinical validations to correlate predicted stress patterns with long-term outcomes.

In conclusion, the current findings affirm that anterior loading and platform mismatch pose significant biomechanical challenges for mandibular overdentures, while simultaneous bilateral posterior contacts and accurate platform alignment act as protective factors. Locator attachments may offer a slight advantage during bilateral loading in the presence of vertical discrepancies, whereas bars can enhance prosthesis-level stability depending on ridge morphology. These insights can inform evidence-based surgical planning and prosthetic design, ultimately enhancing the durability of implant-retained mandibular overdentures.

## Conclusion

Within the limitations of this study, it may be concluded that:

1. The direction of loading, along with alveolar crest morphology and attachment type, significantly impacts stress distribution in mandibular implant-supported overdentures, highlighting the need for careful consideration in treatment planning.
2. Anterior loading produces the highest strains, emphasizing the importance of precise tooth arrangement and adherence to prosthetic principles to enhance the longevity and functionality of the overdenture.
3. Bilateral posterior contacts facilitate optimal stress distribution, supporting the concept of load sharing. Additionally, locator attachments provide a slight advantage under bilateral loading conditions, particularly in cases of implant misalignment, thereby advocating for their use in challenging anatomical situations

## Supporting information

S1 File Stress distribution of bar attachment groups. This PDF file contains stress distribution images of implants, attachment components, prosthesis, tissue layers and loading conditions of all bar attachment groups.
(PDF)

S2 File Stress distribution of locator attachment groups. This PDF file contains stress distribution images of implants, attachment components, prosthesis, tissue layers and loading conditions of all locator attachment groups.
(PDF)
