# Supplementary material for: The role of attachment type and bone height in modulating stress distribution in mandibular overdentures: Insights from finite element analysis
Source: PLoS One. 2026 Jun 16;21(6):e0351498. doi: 10.1371/journal.pone.0351498 (PMC13271450; doi:10.1371/journal.pone.0351498)
Supplement: S1 File — This PDF file contains stress distribution images of implants, attachment components, prosthesis, tissue layers and loading conditions of all bar attachment groups. (PDF) [file pone.0351498.s001.pdf]

BAR MODEL

GRUP 01

INCISAL

Number of nodes = 150886

Number of elements = 776895

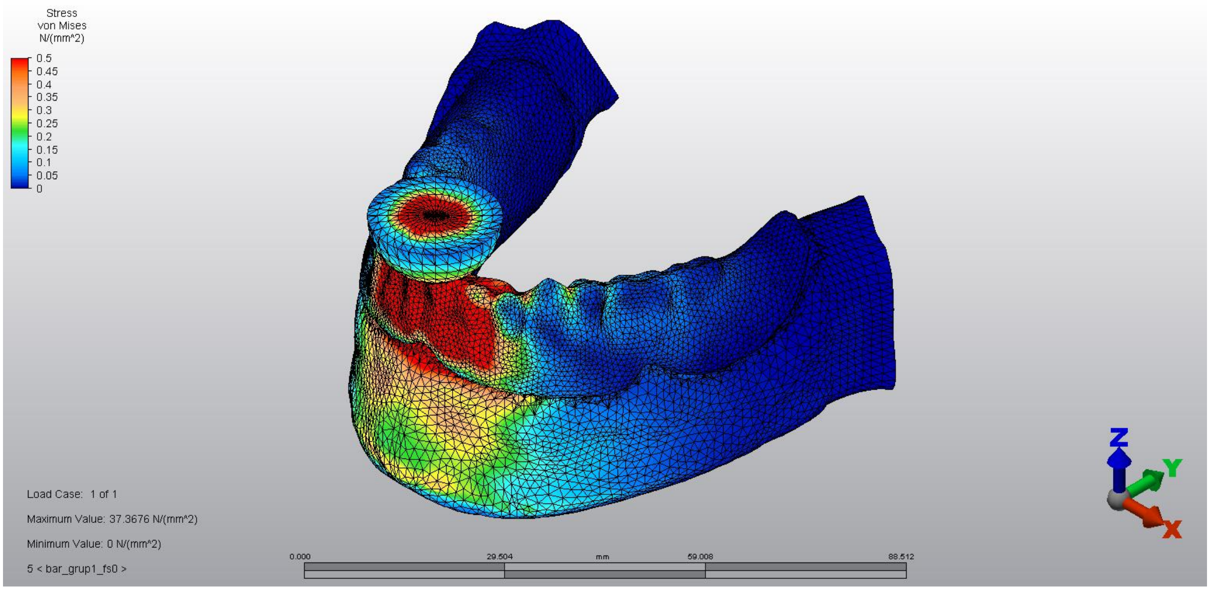

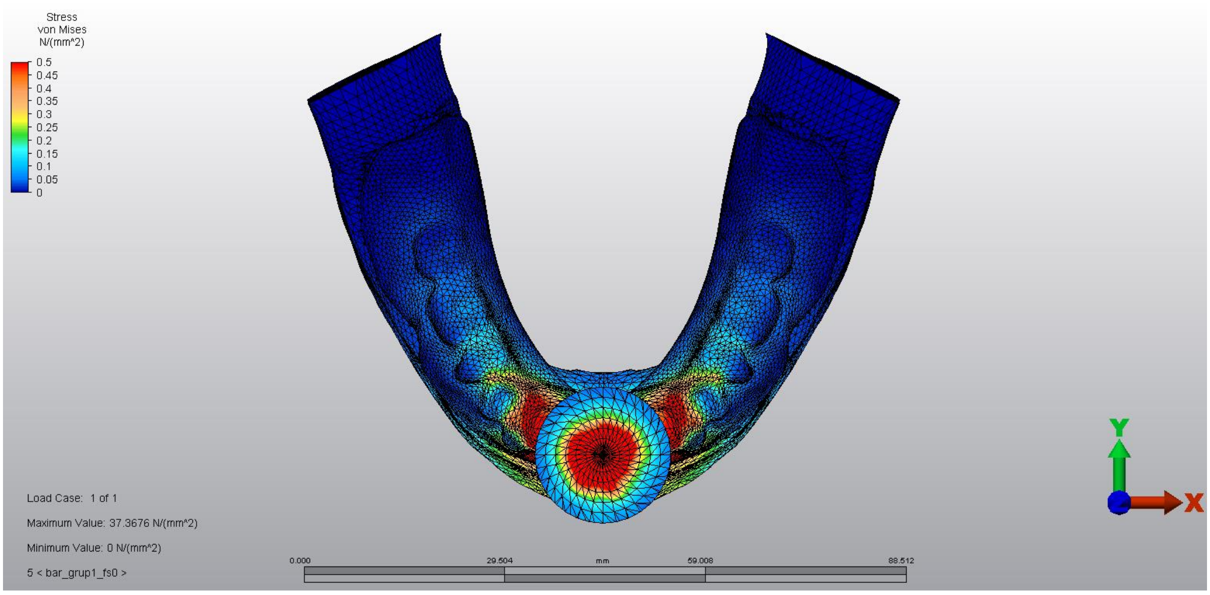

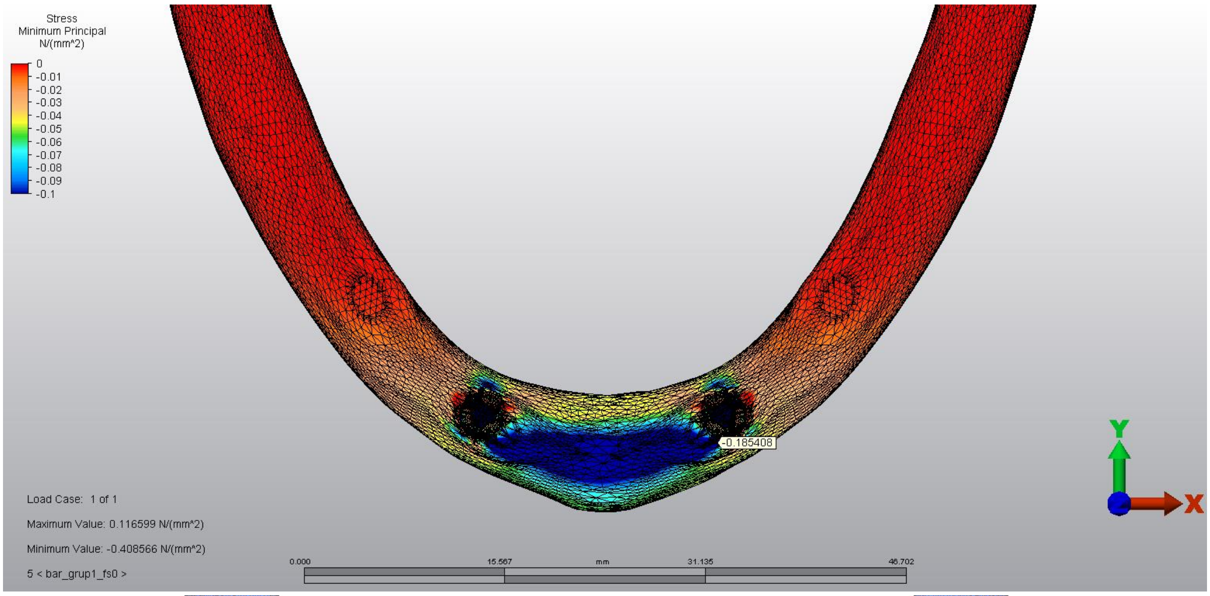
Stress
Minimum Principal
N/(mm^2)
0
-0.01
-0.02
-0.03
-0.04
-0.05
-0.06
-0.07
-0.08
-0.09
-0.1
-0.185408
Load Case: 1 of 1
Maximum Value: 0.116599 N/(mm^2)
Minimum Value: -0.408566 N/(mm^2)
5 < bar_grup1_fs0 >
0.000
15.567
mm
31.135
46.702
Y
X

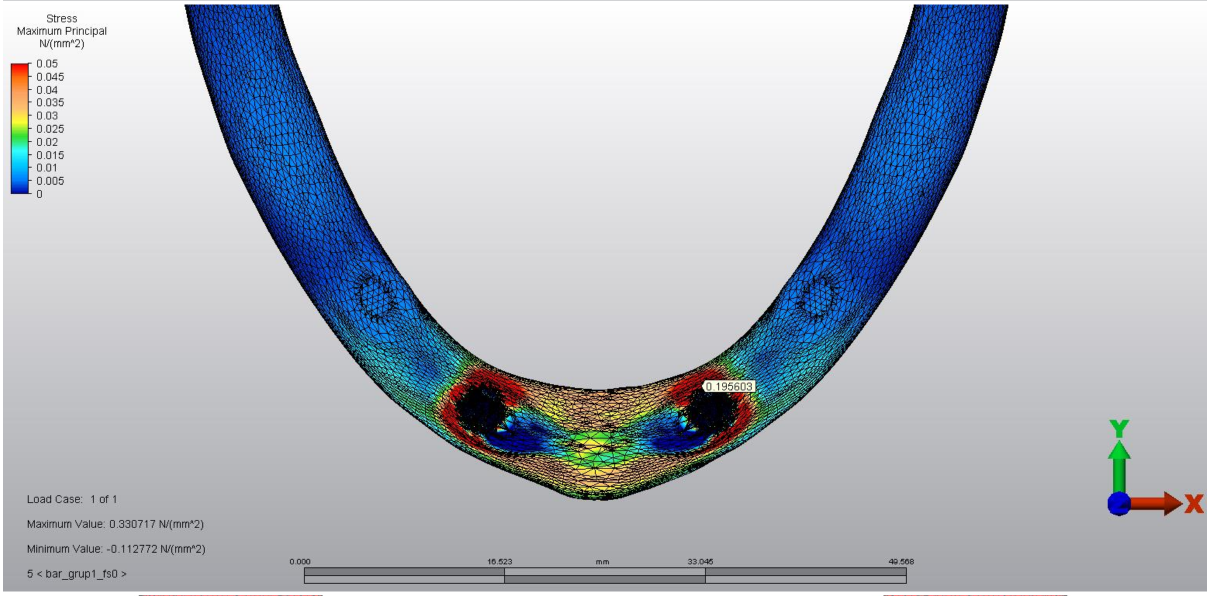
Stress
Maximum Principal
N/(mm^2)
0.05
0.045
0.04
0.035
0.03
0.025
0.02
0.015
0.01
0.005
0
0.195603
Load Case: 1 of 1
Maximum Value: 0.330717 N/(mm^2)
Minimum Value: -0.112772 N/(mm^2)
5 < bar_grup1_fs0 >
0.000
16.523
mm
33.045
49.568
Y
X

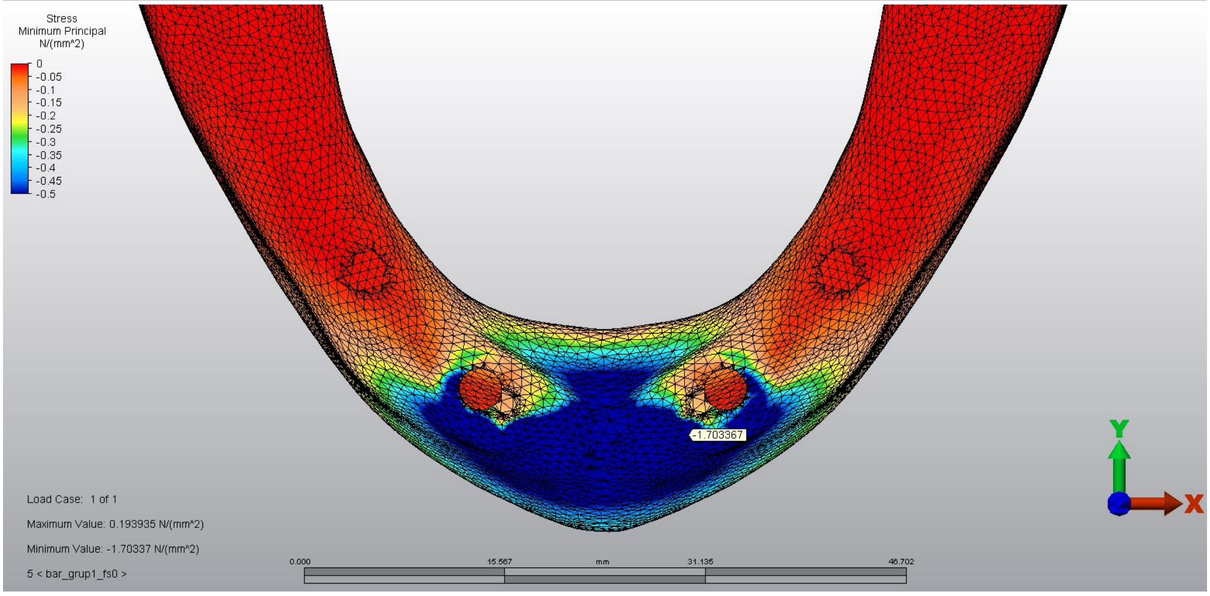
Stress
Minimum Principal
N/(mm^2)
0
-0.05
-0.1
-0.15
-0.2
-0.25
-0.3
-0.35
-0.4
-0.45
-0.5
-1.703367
Load Case: 1 of 1
Maximum Value: 0.193935 N/(mm^2)
Minimum Value: -1.70337 N/(mm^2)
5 < bar_grup1_fs0 >
0.000
15.567
mm
31.135
46.702
Y
X

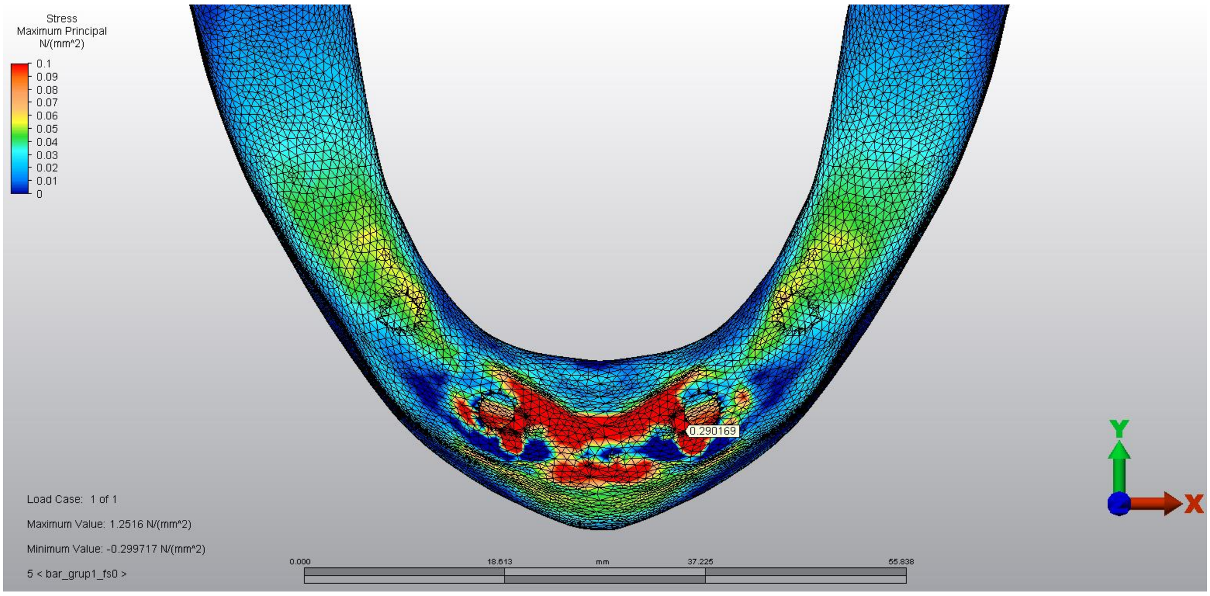
Stress
Maximum Principal
N/(mm^2)
0.1
0.09
0.08
0.07
0.06
0.05
0.04
0.03
0.02
0.01
0
0.290169
Load Case: 1 of 1
Maximum Value: 1.2516 N/(mm^2)
Minimum Value: -0.299717 N/(mm^2)
5 < bar_grup1_fs0 >
0.000
18.613
mm
37.225
55.838
Y
X

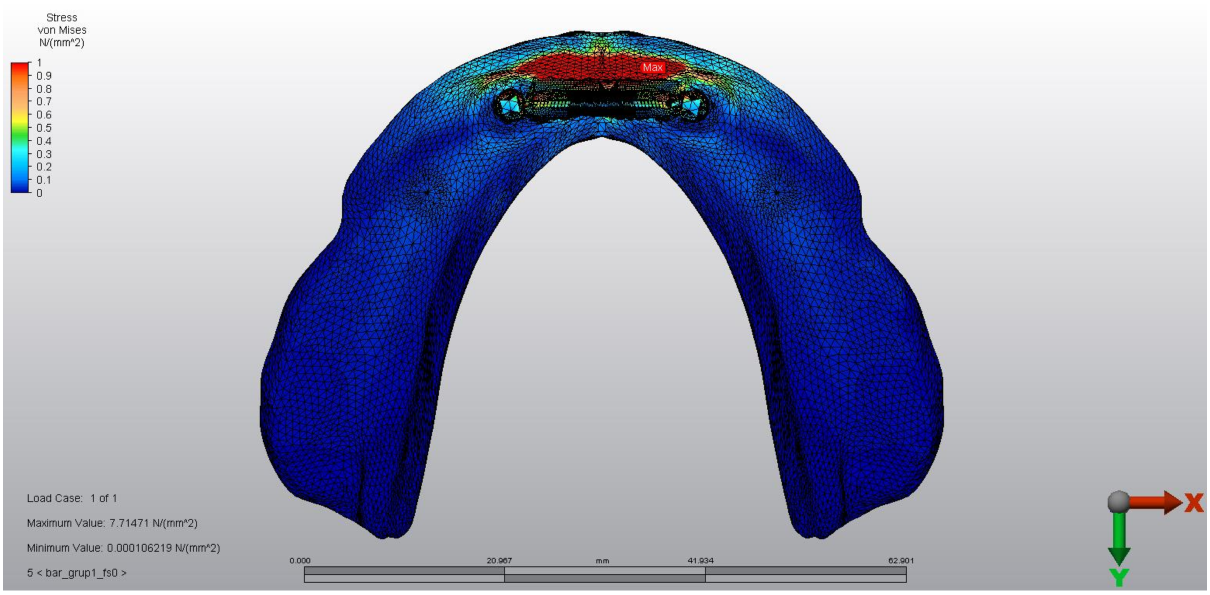
Stress
von Mises
N/(mm^2)
1
0.9
0.8
0.7
0.6
0.5
0.4
0.3
0.2
0.1
0
Max
Load Case: 1 of 1
Maximum Value: 7.71471 N/(mm^2)
Minimum Value: 0.000106219 N/(mm^2)
5 < bar_grup1_fs0 >
0.000
20.967
mm
41.934
62.901
X
Y

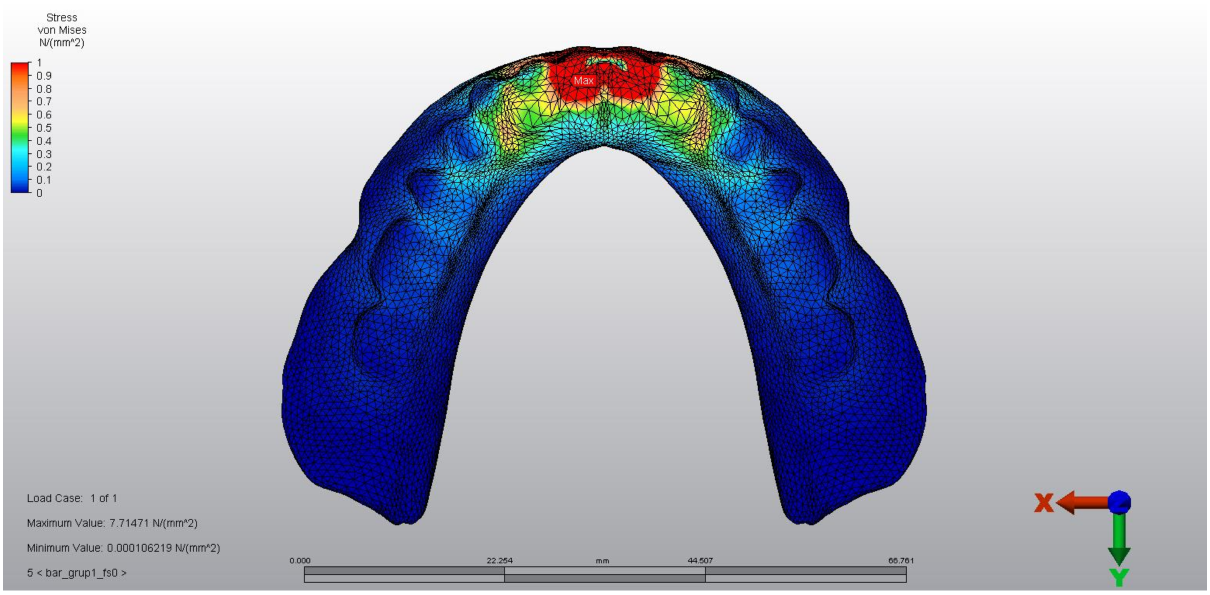
Stress
von Mises
N/(mm^2)
1
0.9
0.8
0.7
0.6
0.5
0.4
0.3
0.2
0.1
0
Max
Load Case: 1 of 1
Maximum Value: 7.71471 N/(mm^2)
Minimum Value: 0.000106219 N/(mm^2)
5 < bar_grup1_fs0 >
0.000
22.254
mm
44.507
66.761
X
Y

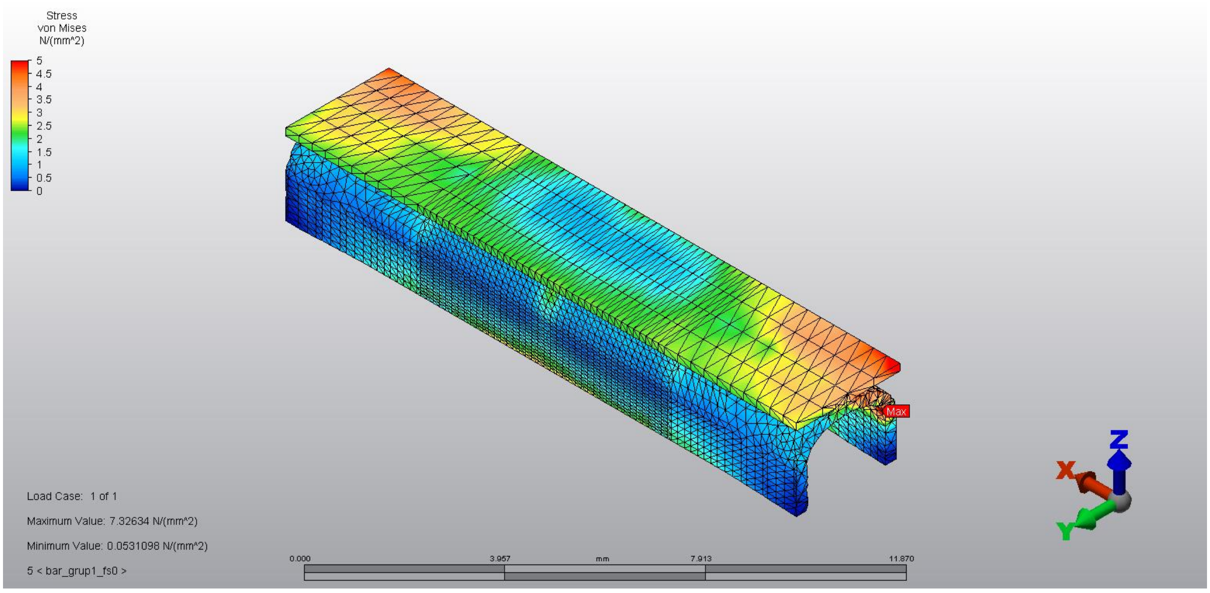
Stress
von Mises
N/(mm^2)
5
4.5
4
3.5
3
2.5
2
1.5
1
0.5
0
Max
Load Case: 1 of 1
Maximum Value: 7.32634 N/(mm^2)
Minimum Value: 0.0531098 N/(mm^2)
5 < bar_grup1_fs0 >
0.000
3.957
mm
7.913
11.870
X
Y
Z

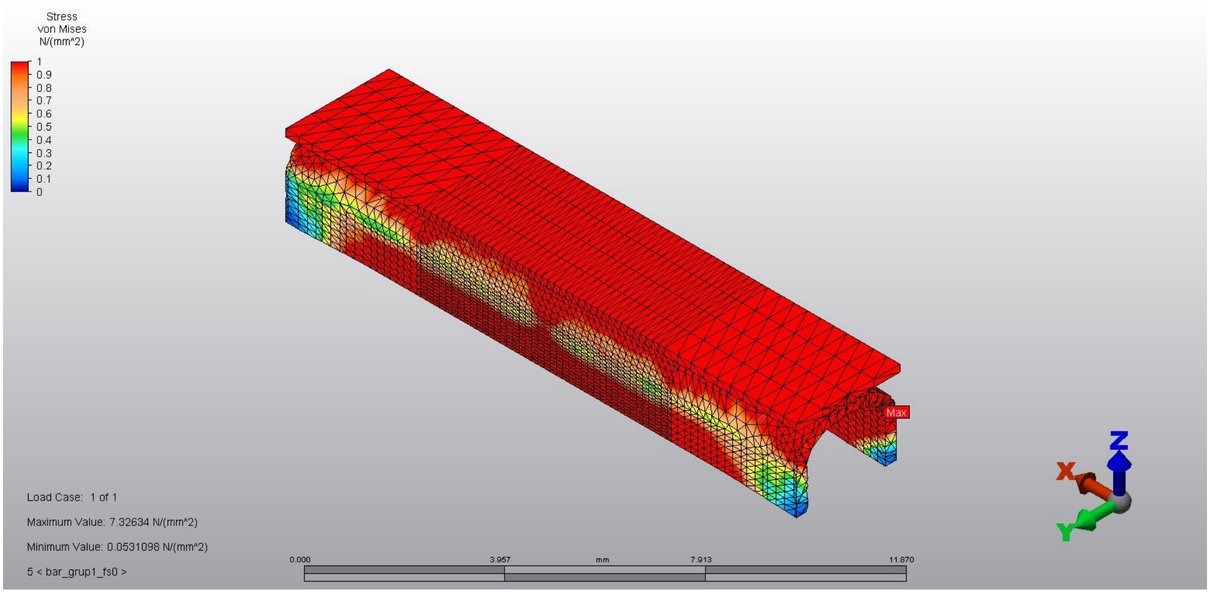
Stress
von Mises
N/(mm^2)
1
0.9
0.8
0.7
0.6
0.5
0.4
0.3
0.2
0.1
0
Max
Load Case: 1 of 1
Maximum Value: 7.32634 N/(mm^2)
Minimum Value: 0.0531098 N/(mm^2)
5 < bar_grup1_fs0 >
0.000
3.957
mm
7.913
11.870
X
Y
Z

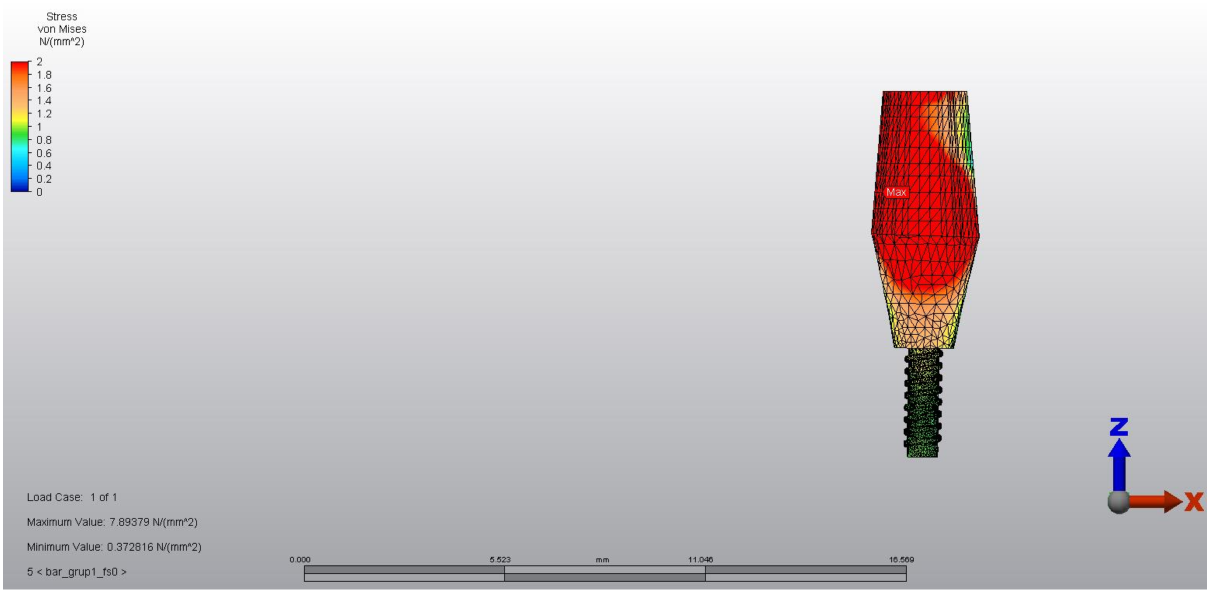
Stress
von Mises
N/(mm^2)
2
1.8
1.6
1.4
1.2
1
0.8
0.6
0.4
0.2
0
Max
Load Case: 1 of 1
Maximum Value: 7.89379 N/(mm^2)
Minimum Value: 0.372816 N/(mm^2)
5 < bar_grup1_fs0 >
0.000
5.523
mm
11.046
16.569
Z
X

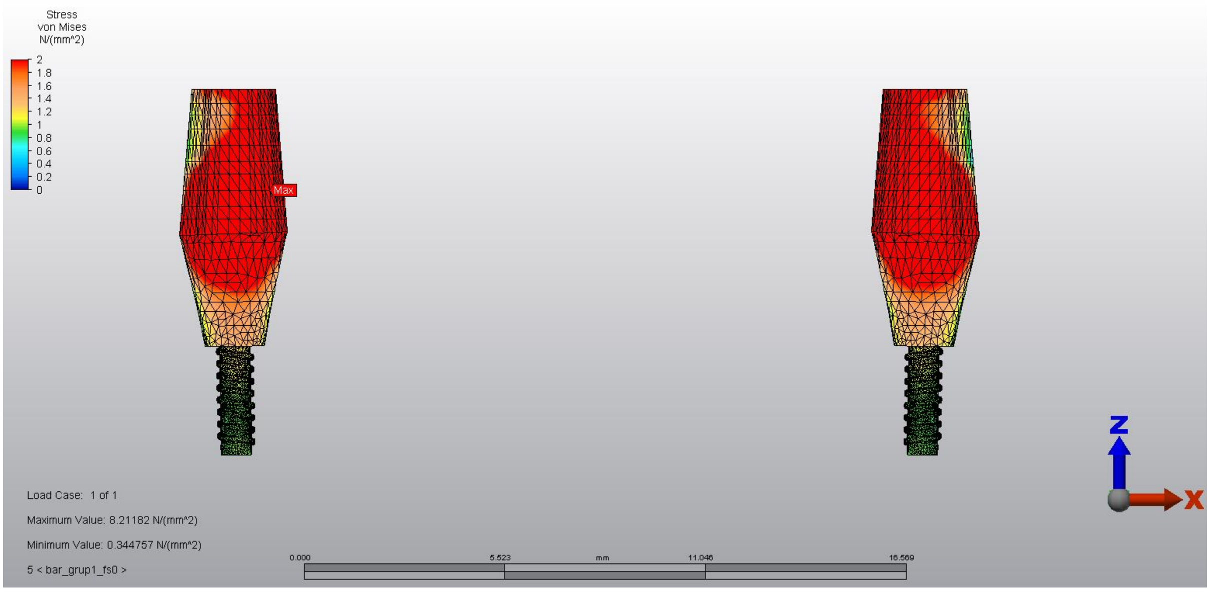
Stress
von Mises
N/(mm^2)
2
1.8
1.6
1.4
1.2
1
0.8
0.6
0.4
0.2
0
Max
Z
X
Load Case: 1 of 1
Maximum Value: 8.21182 N/(mm^2)
Minimum Value: 0.344757 N/(mm^2)
5 < bar_grup1_fs0 >
0.000
5.523
mm
11.046
16.569

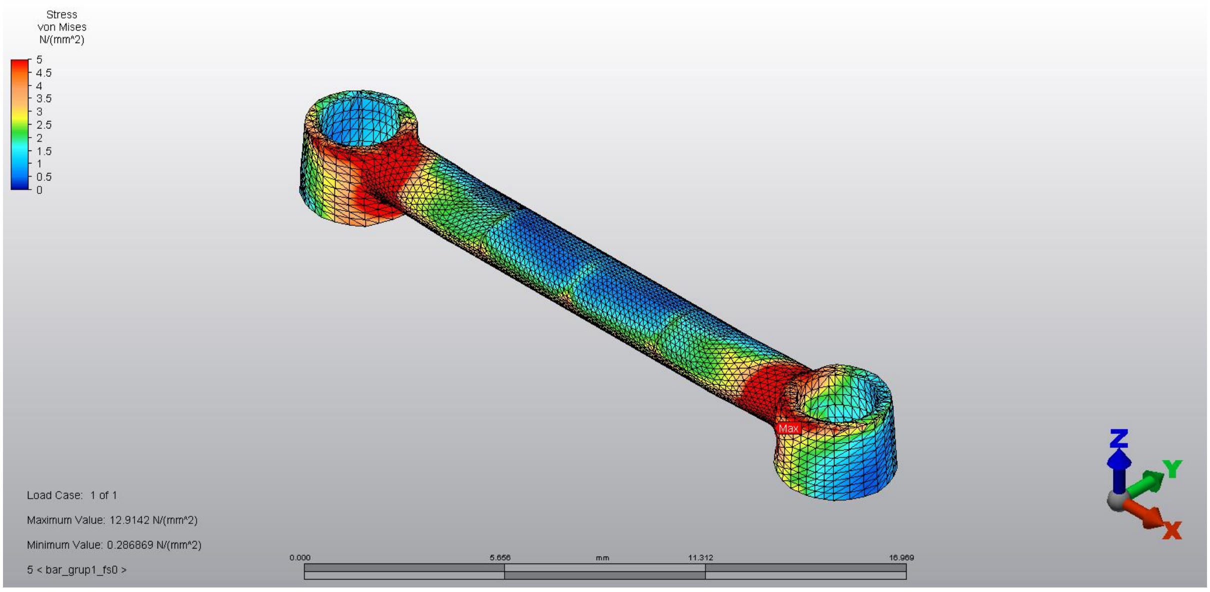
Stress
von Mises
N/(mm^2)
5
4.5
4
3.5
3
2.5
2
1.5
1
0.5
0
Max
Z
Y
X
Load Case: 1 of 1
Maximum Value: 12.9142 N/(mm^2)
Minimum Value: 0.286869 N/(mm^2)
5 < bar_grup1_fs0 >
0.000
5.656
mm
11.312
16.969

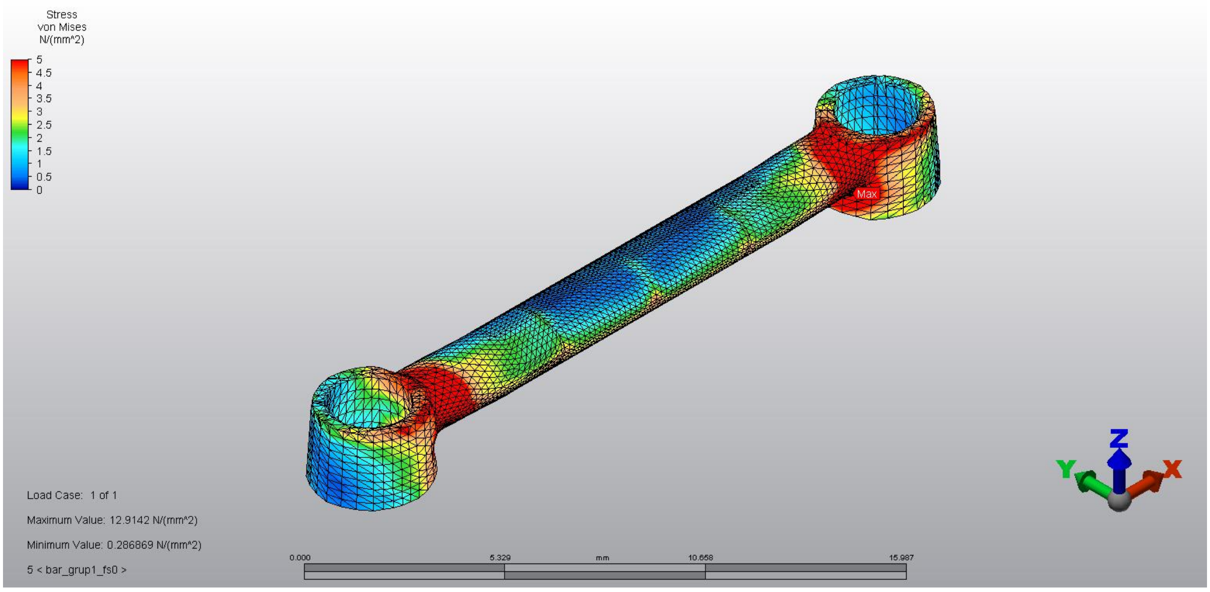

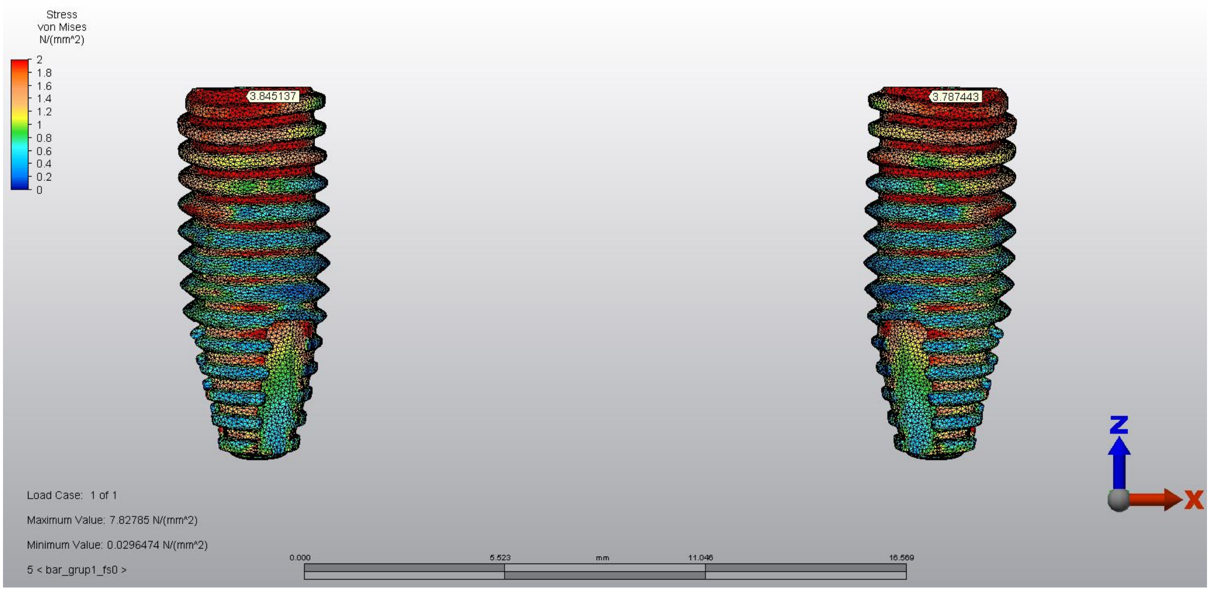

UNILATERAL

Number of nodes = 150853

Number of elements = 776875

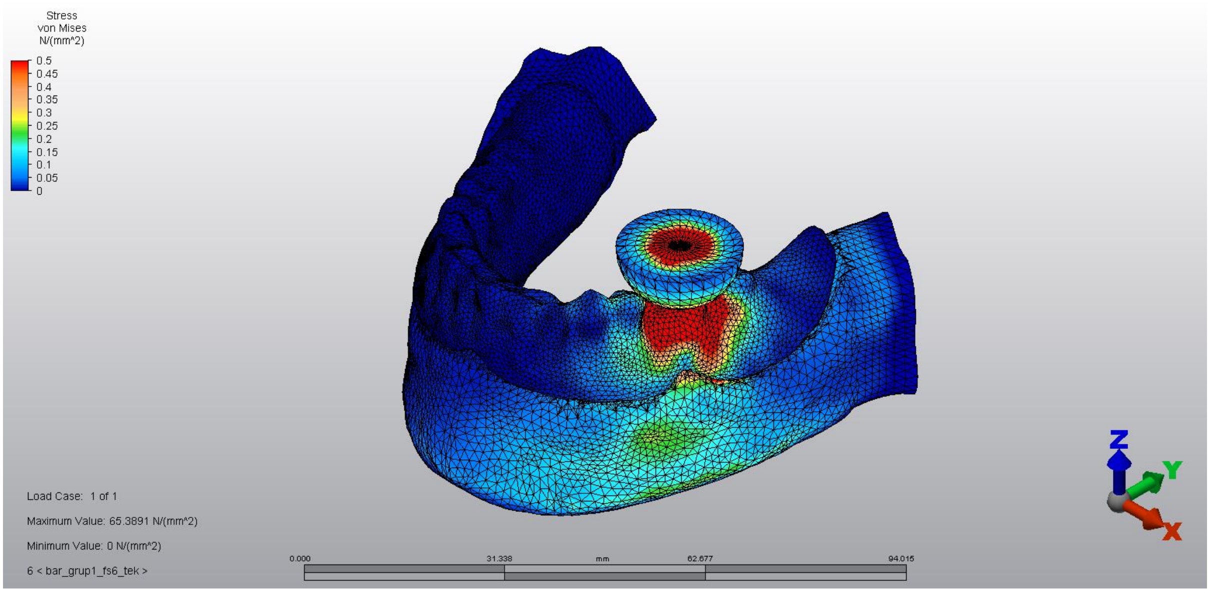
Stress
von Mises
N/(mm^2)
0.5
0.45
0.4
0.35
0.3
0.25
0.2
0.15
0.1
0.05
0
Load Case: 1 of 1
Maximum Value: 65.3891 N/(mm^2)
Minimum Value: 0 N/(mm^2)
6 < bar_grup1_fs6_tek >
0.000
31.338
mm
62.677
94.015
Z
Y
X

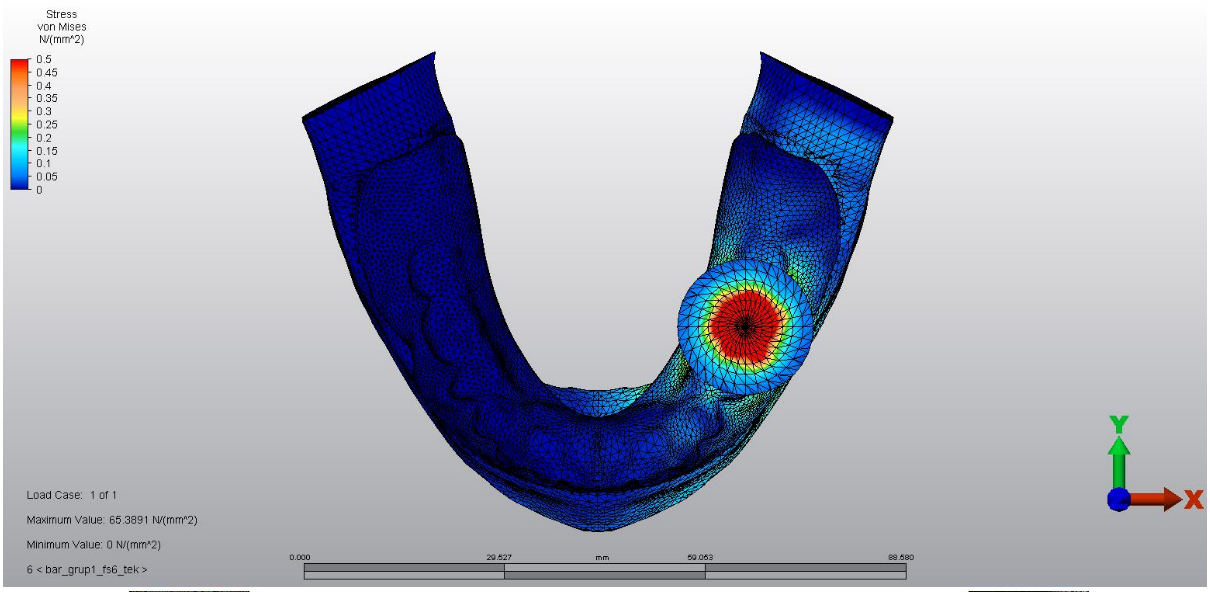
Stress
von Mises
N/(mm^2)
0.5
0.45
0.4
0.35
0.3
0.25
0.2
0.15
0.1
0.05
0
Load Case: 1 of 1
Maximum Value: 65.3891 N/(mm^2)
Minimum Value: 0 N/(mm^2)
6 < bar_grup1_fs6_tek >
0.000
29.527
mm
59.053
88.580
Y
X

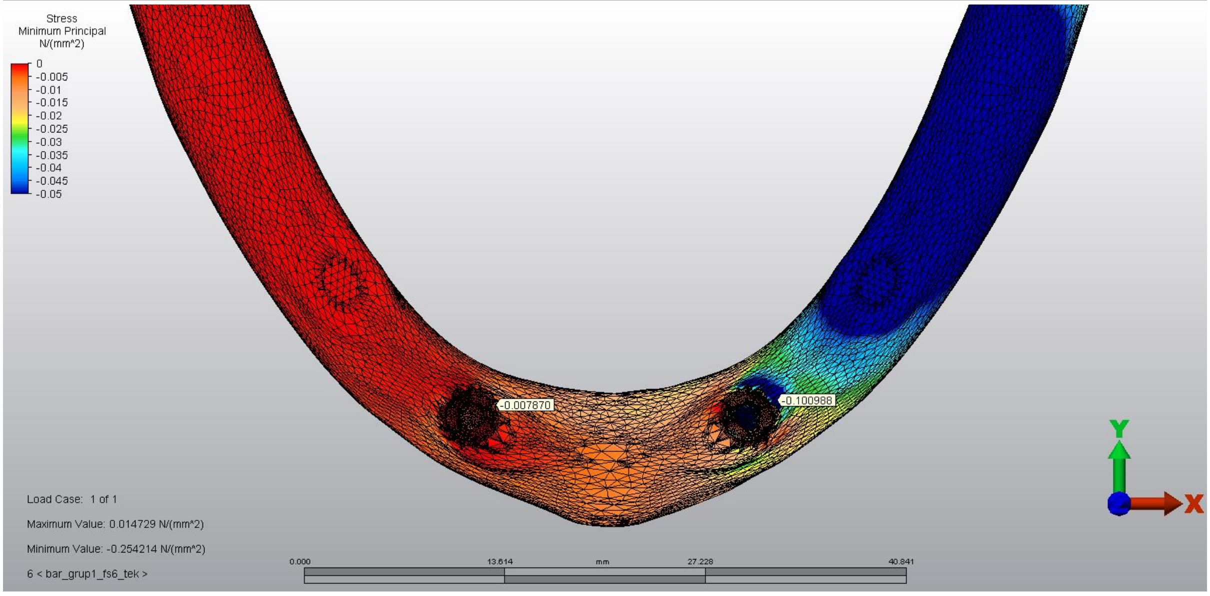
Stress
Minimum Principal
N/(mm^2)
0
-0.005
-0.01
-0.015
-0.02
-0.025
-0.03
-0.035
-0.04
-0.045
-0.05
-0.007870
-0.100988
Load Case: 1 of 1
Maximum Value: 0.014729 N/(mm^2)
Minimum Value: -0.254214 N/(mm^2)
6 < bar_grup1_fs6_tek >
0.000
13.614
mm
27.228
40.841
Y
X

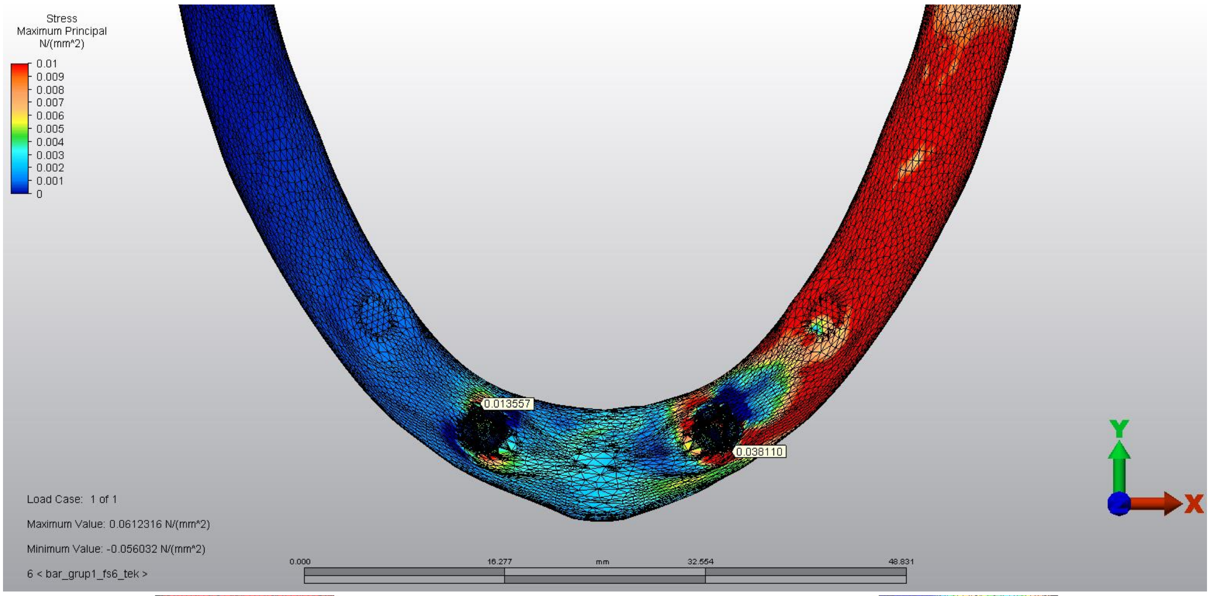
Stress
Maximum Principal
N/(mm^2)
0.01
0.009
0.008
0.007
0.006
0.005
0.004
0.003
0.002
0.001
0
0.013557
0.038110
Load Case: 1 of 1
Maximum Value: 0.0612316 N/(mm^2)
Minimum Value: -0.056032 N/(mm^2)
6 < bar_grup1_fs6_tek >
0.000
16.277
mm
32.554
48.831
Y
X

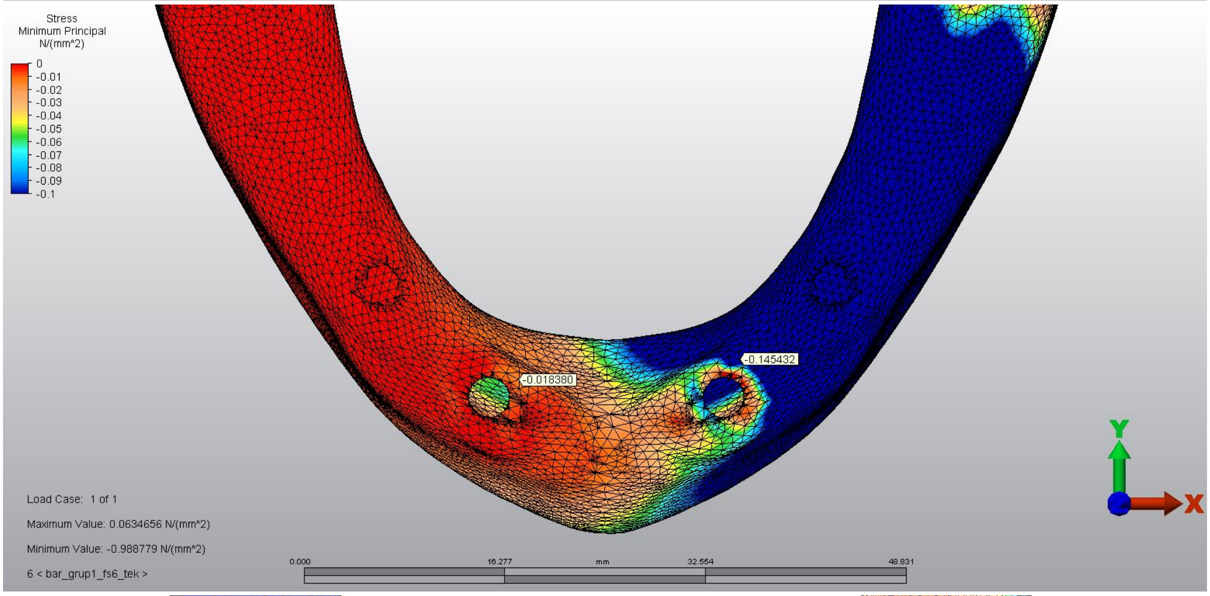
Stress
Minimum Principal
N/(mm^2)
0
-0.01
-0.02
-0.03
-0.04
-0.05
-0.06
-0.07
-0.08
-0.09
-0.1
-0.018380
-0.145432
Load Case: 1 of 1
Maximum Value: 0.0634656 N/(mm^2)
Minimum Value: -0.988779 N/(mm^2)
6 < bar_grup1_fs6_tek >
0.000
16.277
mm
32.554
48.831
Y
X

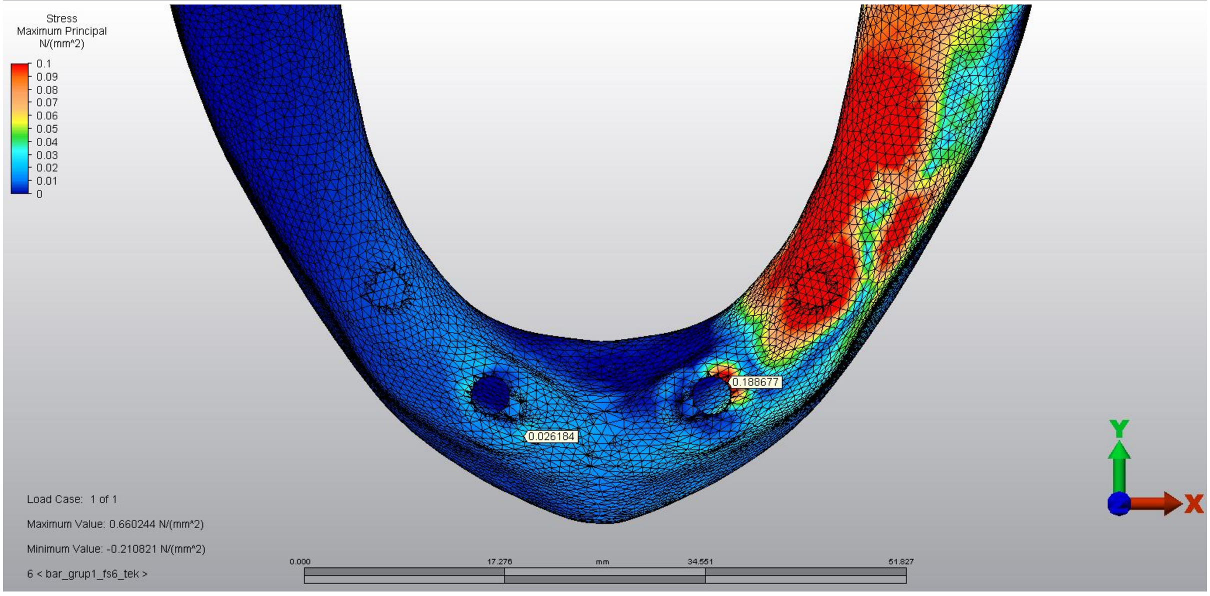
Stress
Maximum Principal
N/(mm^2)
0.1
0.09
0.08
0.07
0.06
0.05
0.04
0.03
0.02
0.01
0
0.188677
0.026184
Load Case: 1 of 1
Maximum Value: 0.660244 N/(mm^2)
Minimum Value: -0.210821 N/(mm^2)
6 < bar_grup1_fs6_tek >
0.000
17.276
mm
34.551
51.827
Y
X

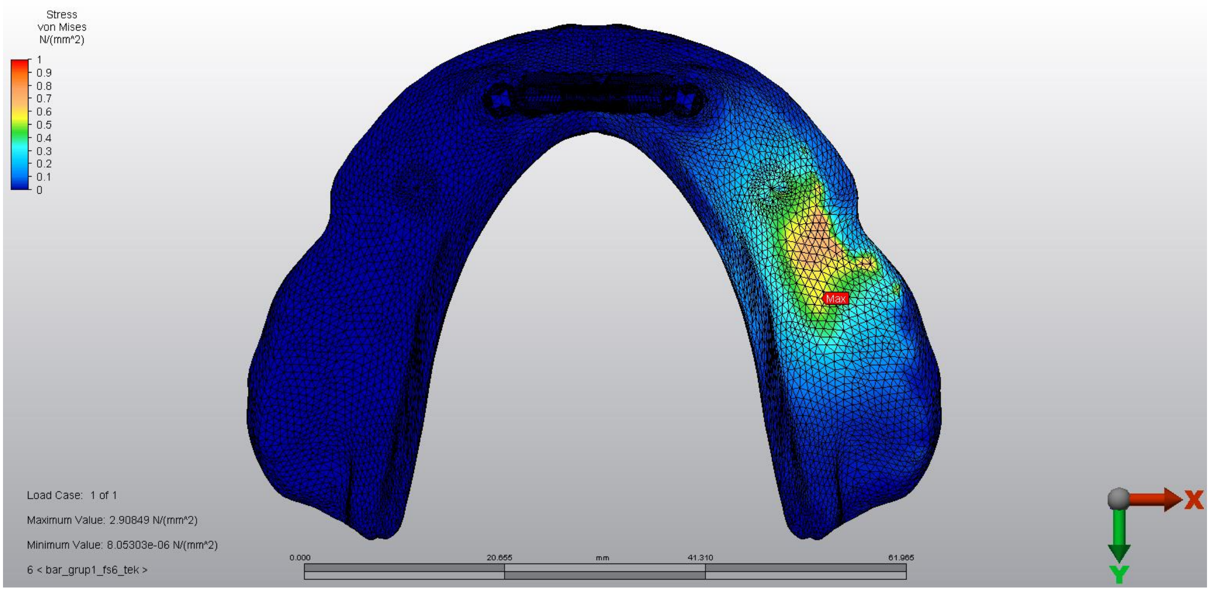
Stress
von Mises
N/(mm^2)
1
0.9
0.8
0.7
0.6
0.5
0.4
0.3
0.2
0.1
0
Max
Load Case: 1 of 1
Maximum Value: 2.90849 N/(mm^2)
Minimum Value: 8.05303e-06 N/(mm^2)
6 < bar_grup1_fs6_tek >
0.000
20.655
mm
41.310
61.965
X
Y

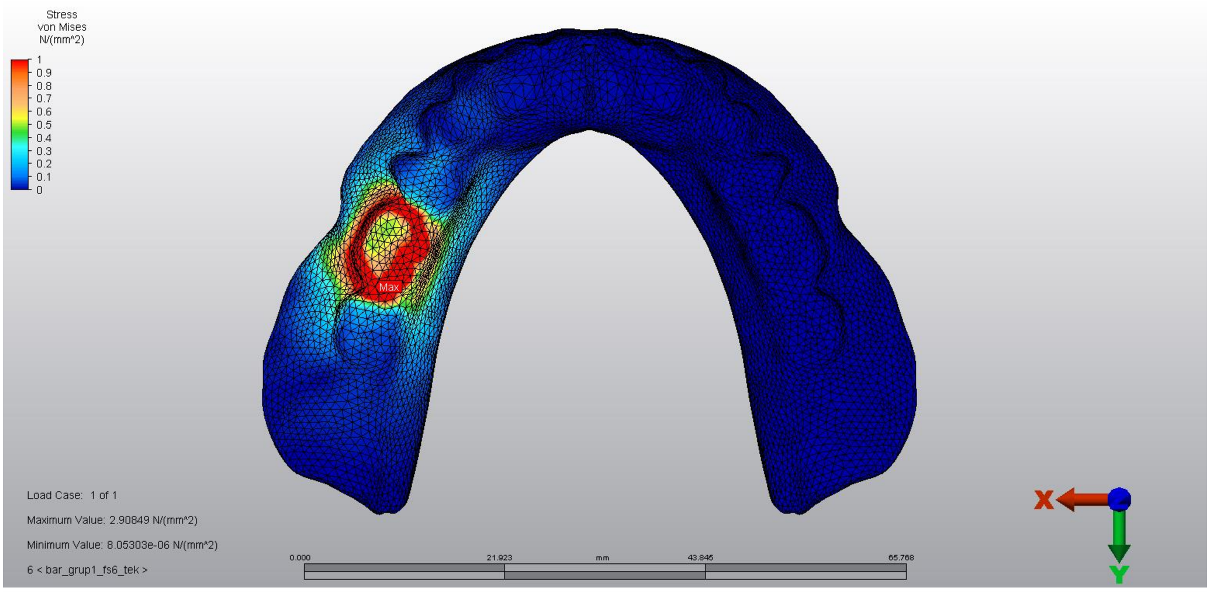
Stress
von Mises
N/(mm^2)
1
0.9
0.8
0.7
0.6
0.5
0.4
0.3
0.2
0.1
0
Max
Load Case: 1 of 1
Maximum Value: 2.90849 N/(mm^2)
Minimum Value: 8.05303e-06 N/(mm^2)
6 < bar_grup1_fs6_tek >
0.000
21.923
mm
43.845
65.768
X
Y

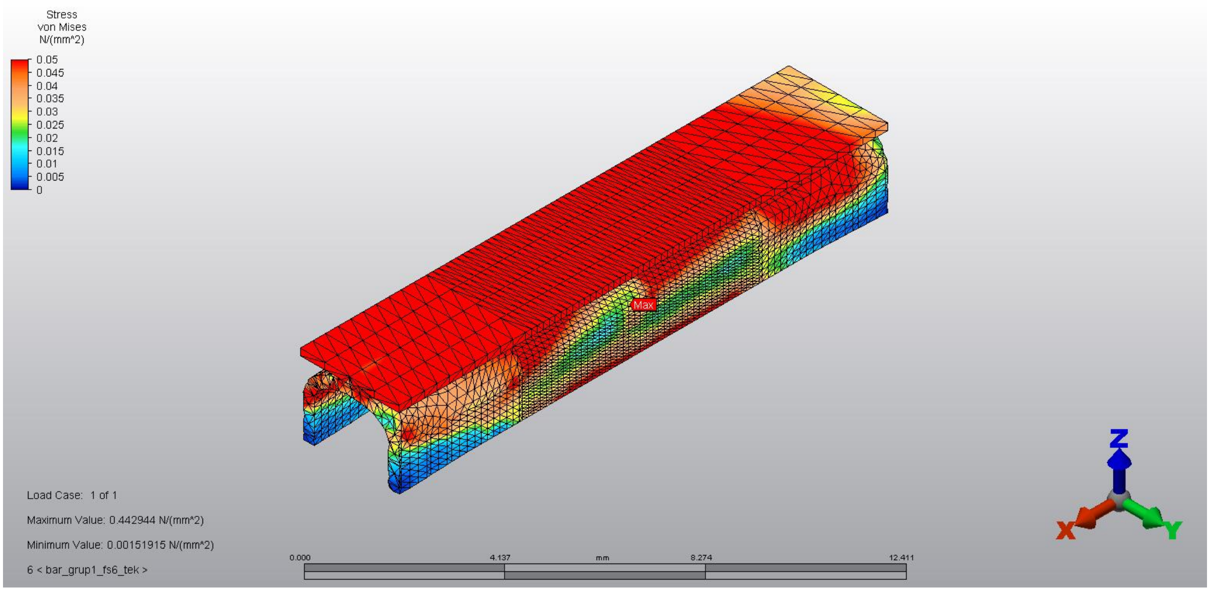
Stress
von Mises
N/(mm^2)
0.05
0.045
0.04
0.035
0.03
0.025
0.02
0.015
0.01
0.005
0
Max
Load Case: 1 of 1
Maximum Value: 0.442944 N/(mm^2)
Minimum Value: 0.00151915 N/(mm^2)
6 < bar_grup1_fs6_tek >
0.000
4.137
mm
8.274
12.411
Z
X
Y

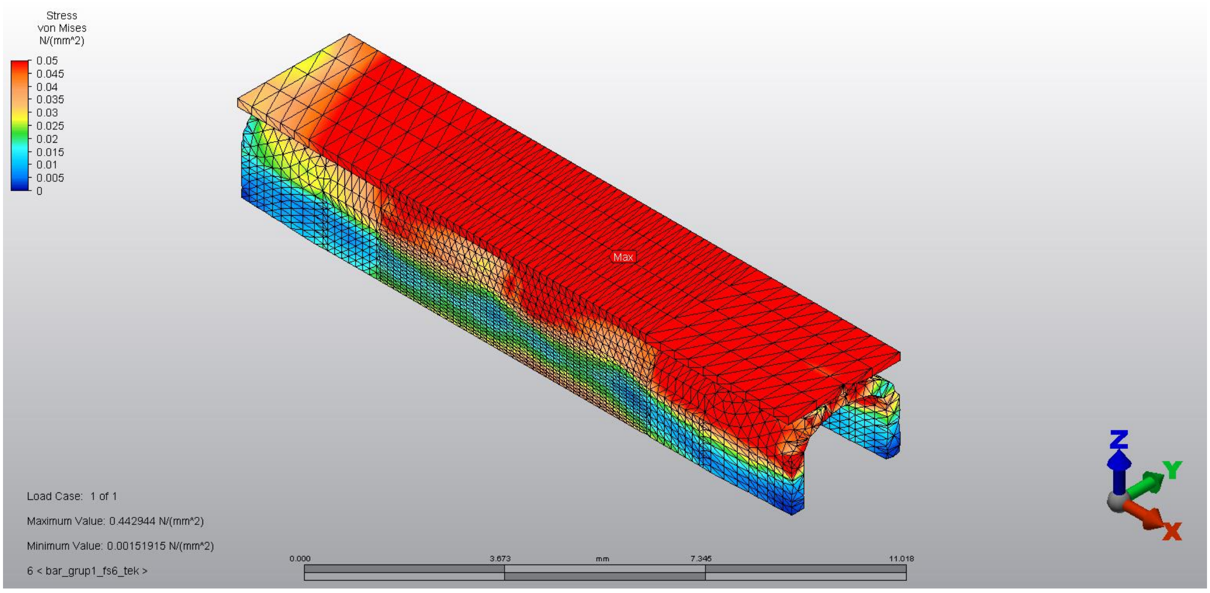
Stress
von Mises
N/(mm^2)
0.05
0.045
0.04
0.035
0.03
0.025
0.02
0.015
0.01
0.005
0
Max
Load Case: 1 of 1
Maximum Value: 0.442944 N/(mm^2)
Minimum Value: 0.00151915 N/(mm^2)
6 < bar_grup1_fs6_tek >
0.000
3.673
mm
7.345
11.018
Z
Y
X

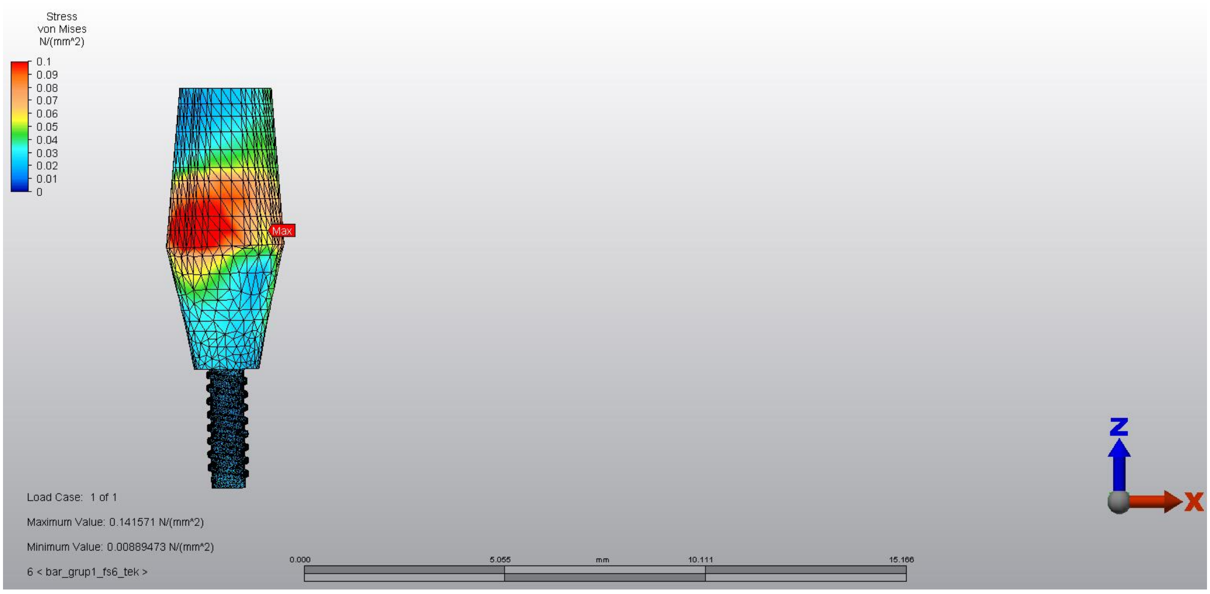
Stress
von Mises
N/(mm^2)
0.1
0.09
0.08
0.07
0.06
0.05
0.04
0.03
0.02
0.01
0
Max
Load Case: 1 of 1
Maximum Value: 0.141571 N/(mm^2)
Minimum Value: 0.00889473 N/(mm^2)
6 < bar_grup1_fs6_tek >
0.000
5.055
mm
10.111
15.166
Z
X

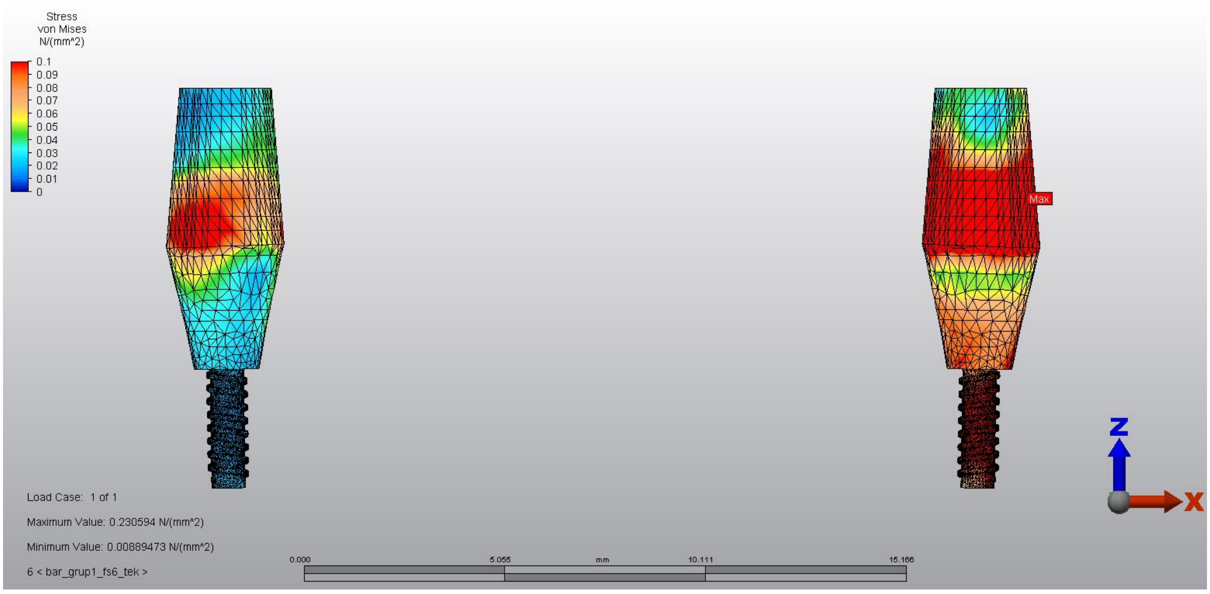
Stress
von Mises
N/(mm^2)
0.1
0.09
0.08
0.07
0.06
0.05
0.04
0.03
0.02
0.01
0
Max
Load Case: 1 of 1
Maximum Value: 0.230594 N/(mm^2)
Minimum Value: 0.00889473 N/(mm^2)
6 < bar_grup1_fs6_tek >
0.000
5.055
mm
10.111
15.166
Z
X

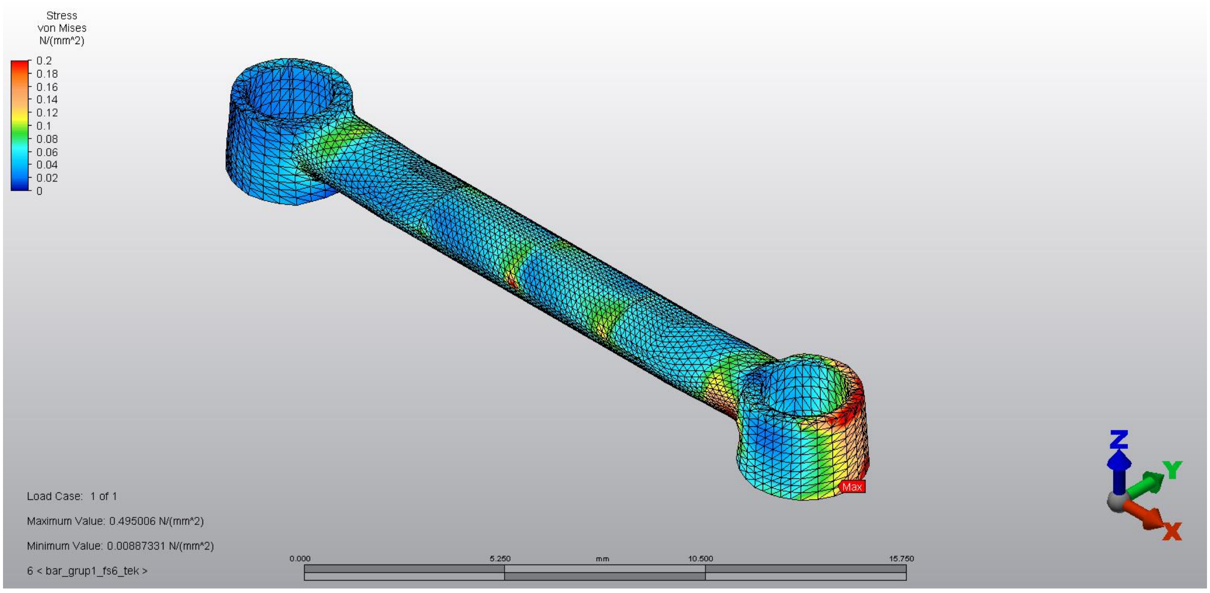
Stress
von Mises
N/(mm^2)
0.2
0.18
0.16
0.14
0.12
0.1
0.08
0.06
0.04
0.02
0
Max
Load Case: 1 of 1
Maximum Value: 0.495006 N/(mm^2)
Minimum Value: 0.00887331 N/(mm^2)
6 < bar_grup1_fs6_tek >
0.000
5.250
mm
10.500
15.750
Z
Y
X

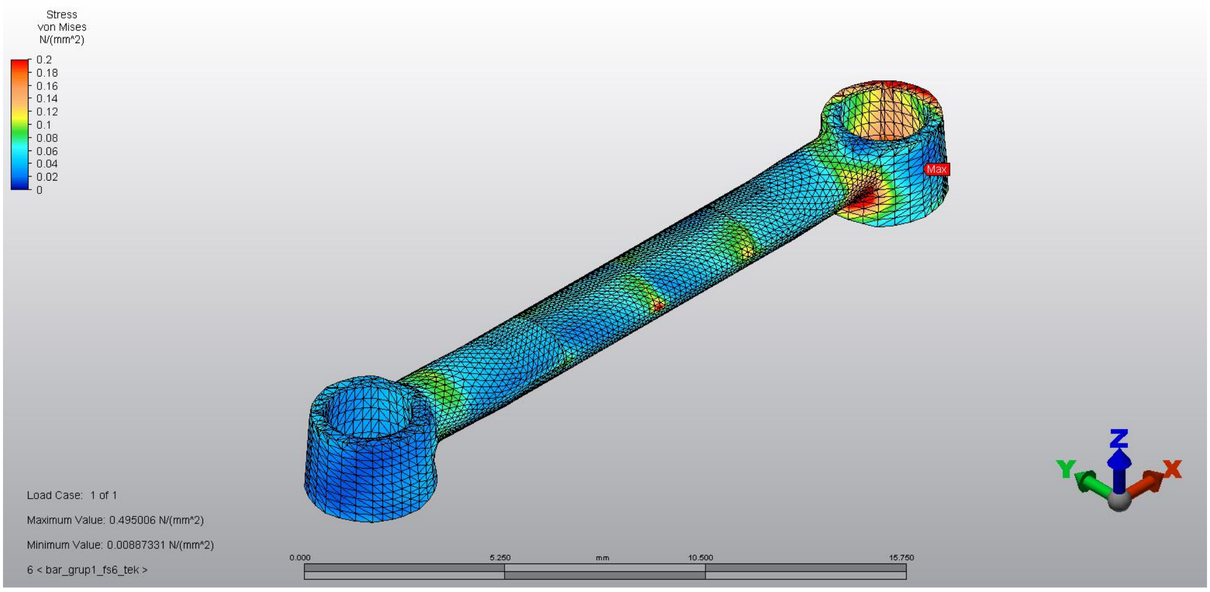
Stress
von Mises
N/(mm^2)
0.2
0.18
0.16
0.14
0.12
0.1
0.08
0.06
0.04
0.02
0
Max
Load Case: 1 of 1
Maximum Value: 0.495006 N/(mm^2)
Minimum Value: 0.00887331 N/(mm^2)
6 < bar_grup1_fs6_tek >
0.000
5.250
mm
10.500
15.750
Z
Y
X

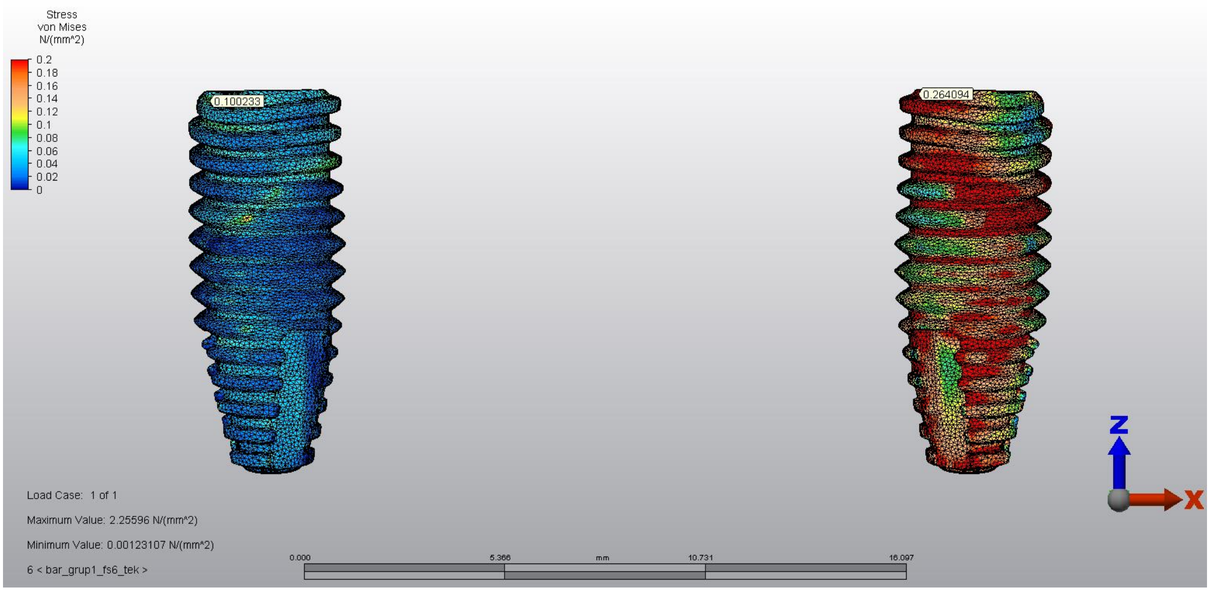
Stress
von Mises
N/(mm^2)
0.2
0.18
0.16
0.14
0.12
0.1
0.08
0.06
0.04
0.02
0
0.100233
0.264094
Load Case: 1 of 1
Maximum Value: 2.25596 N/(mm^2)
Minimum Value: 0.00123107 N/(mm^2)
6 < bar_grup1_fs6_tek >
0.000
5.366
mm
10.731
16.097
Z
X

BILATERAL

Number of nodes = 151565

Number of elements = 779423

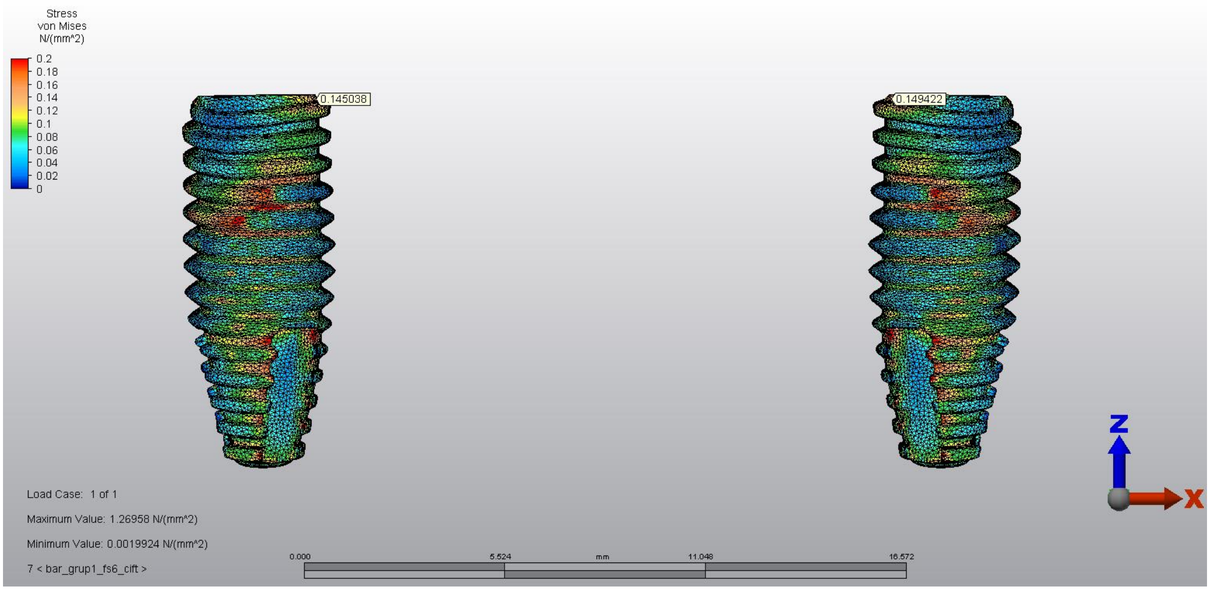

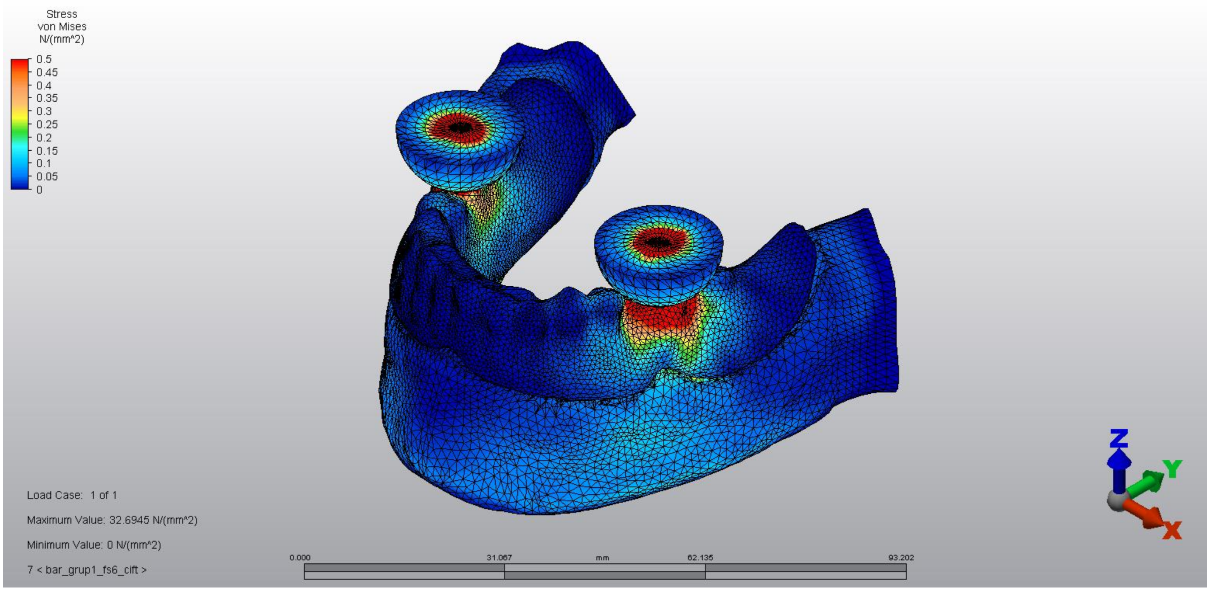

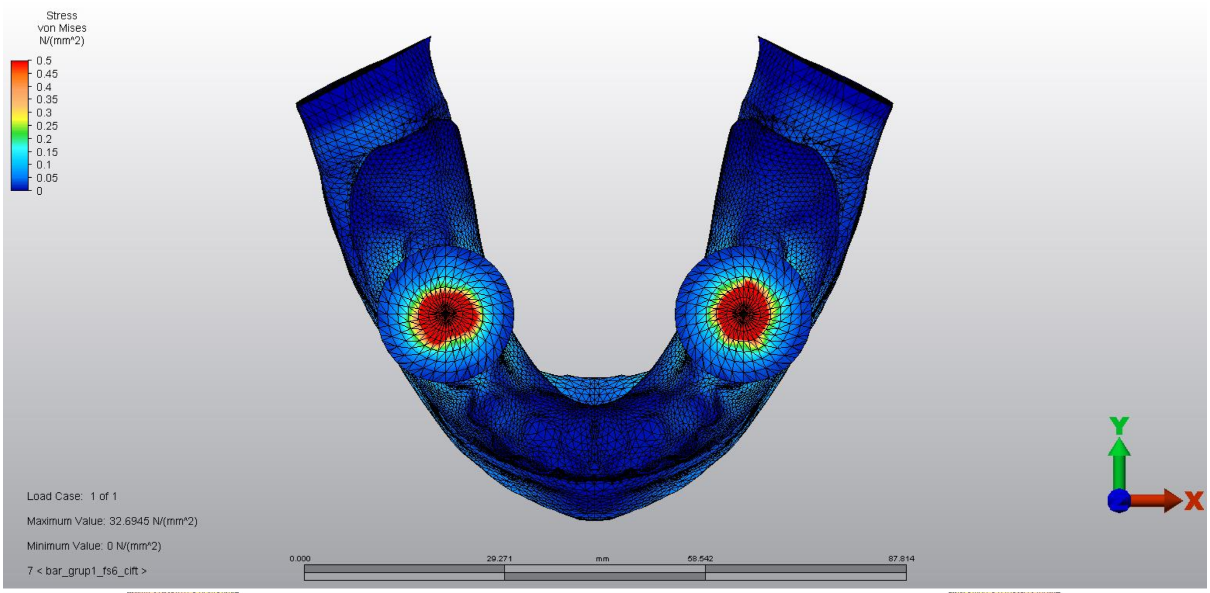
Stress
von Mises
N/(mm^2)
0.5
0.45
0.4
0.35
0.3
0.25
0.2
0.15
0.1
0.05
0
Load Case: 1 of 1
Maximum Value: 32.6945 N/(mm^2)
Minimum Value: 0 N/(mm^2)
7 < bar_grup1_fs6_cift >
0.000
29.271
mm
58.542
87.814
Y
X

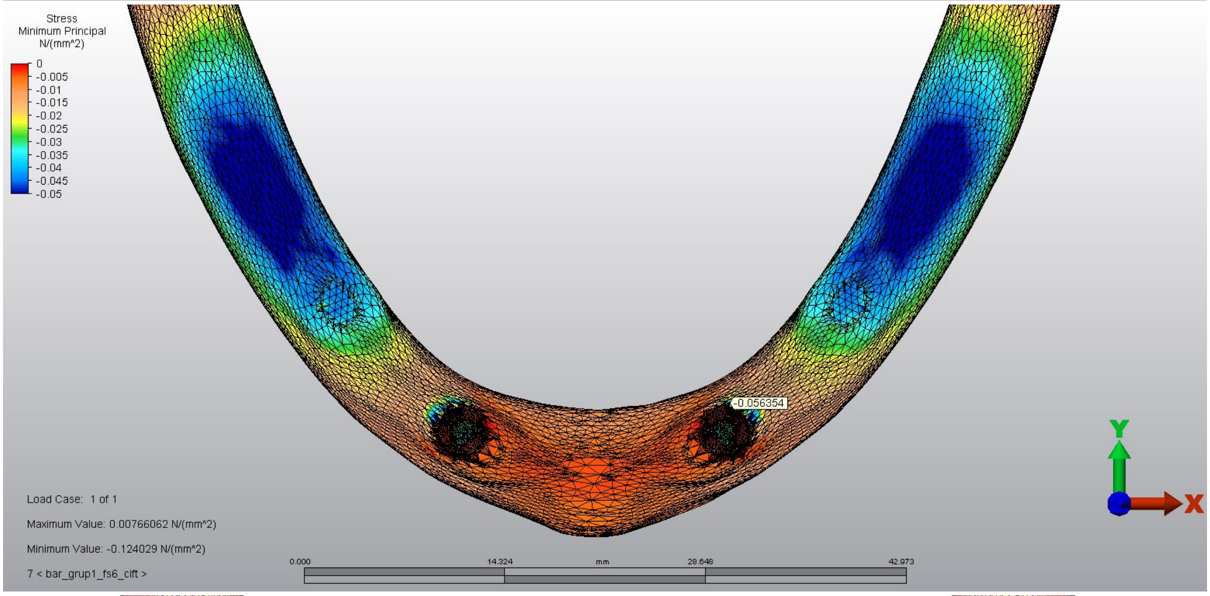
Stress
Minimum Principal
N/(mm^2)
0
-0.005
-0.01
-0.015
-0.02
-0.025
-0.03
-0.035
-0.04
-0.045
-0.05
-0.056354
Load Case: 1 of 1
Maximum Value: 0.00766062 N/(mm^2)
Minimum Value: -0.124029 N/(mm^2)
7 < bar_grup1_fs6_cift >
0.000
14.324
mm
28.648
42.973
Y
X

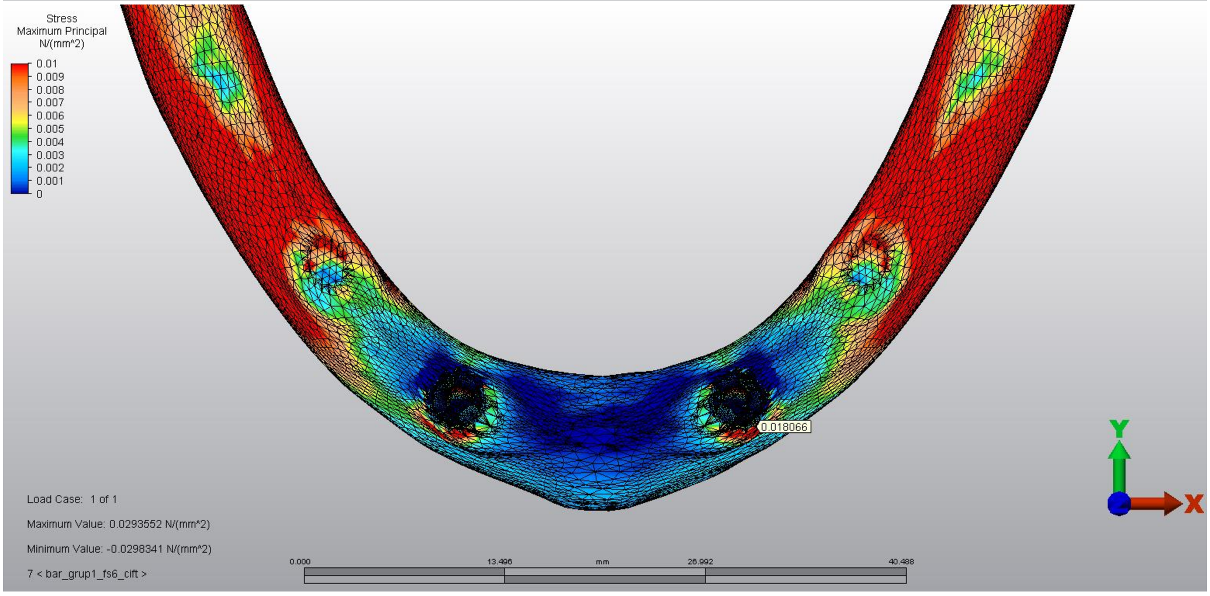
Stress
Maximum Principal
N/(mm^2)
0.01
0.009
0.008
0.007
0.006
0.005
0.004
0.003
0.002
0.001
0
0.018066
Load Case: 1 of 1
Maximum Value: 0.0293552 N/(mm^2)
Minimum Value: -0.0298341 N/(mm^2)
7 < bar_grup1_fs6_cift >
0.000
13.496
mm
26.992
40.488
Y
X

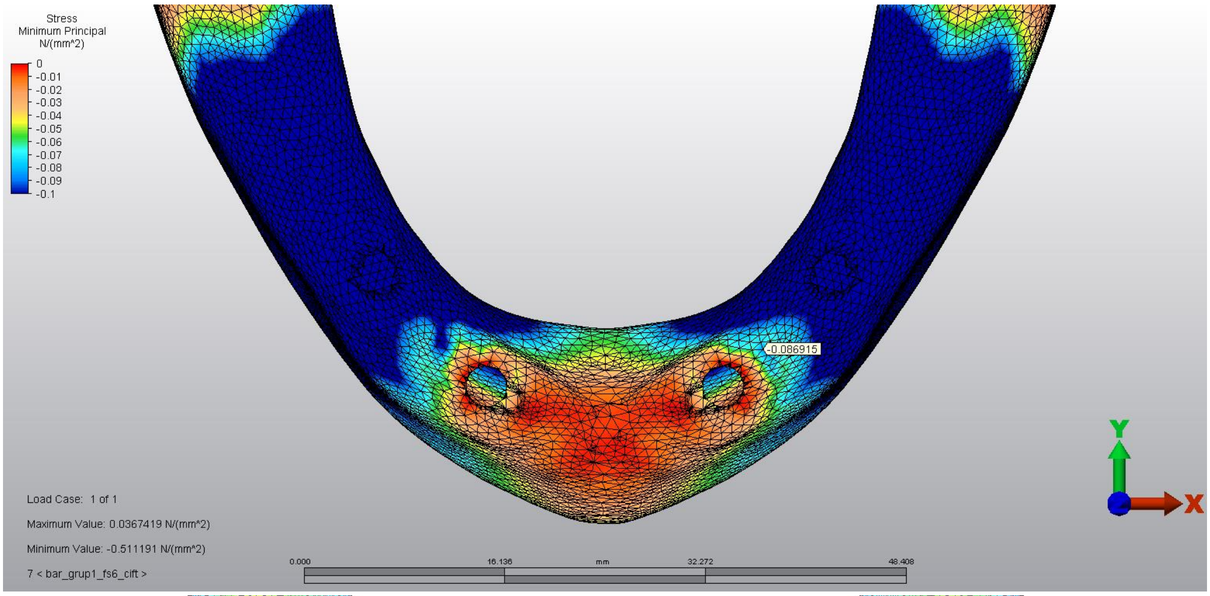
Stress
Minimum Principal
N/(mm^2)
0
-0.01
-0.02
-0.03
-0.04
-0.05
-0.06
-0.07
-0.08
-0.09
-0.1
-0.086915
Load Case: 1 of 1
Maximum Value: 0.0367419 N/(mm^2)
Minimum Value: -0.511191 N/(mm^2)
7 < bar_grup1_fs6_cift >
0.000
16.136
mm
32.272
48.408
Y
X

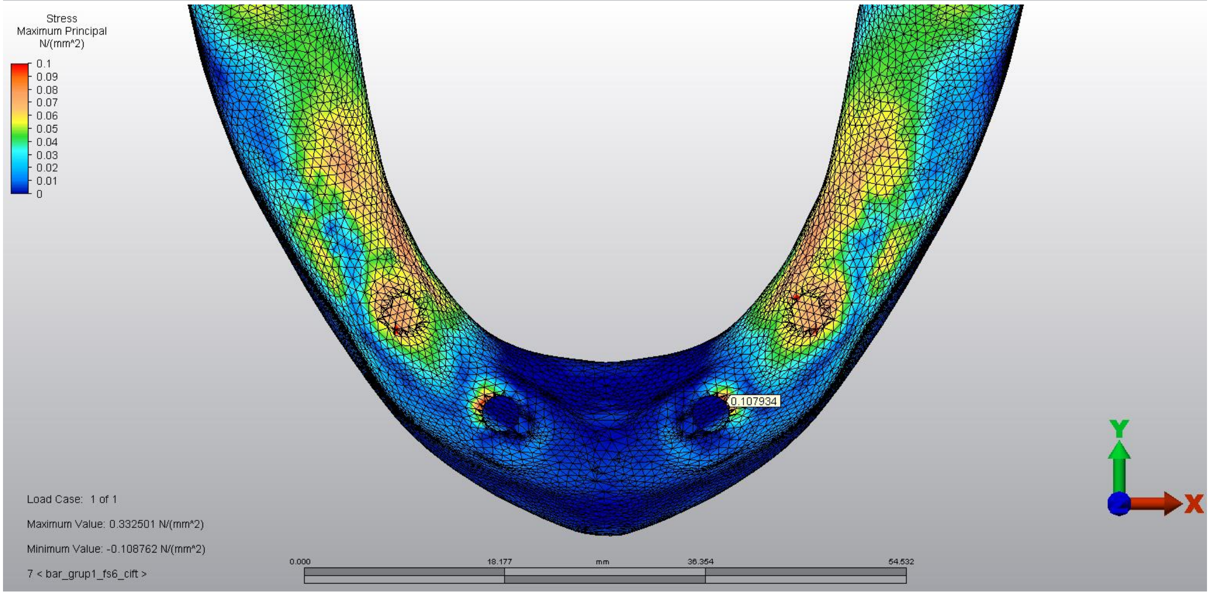
Stress
Maximum Principal
N/(mm^2)
0.1
0.09
0.08
0.07
0.06
0.05
0.04
0.03
0.02
0.01
0
0.107934
Load Case: 1 of 1
Maximum Value: 0.332501 N/(mm^2)
Minimum Value: -0.108762 N/(mm^2)
7 < bar_grup1_fs6_cift >
0.000
18.177
mm
36.354
54.532
Y
X

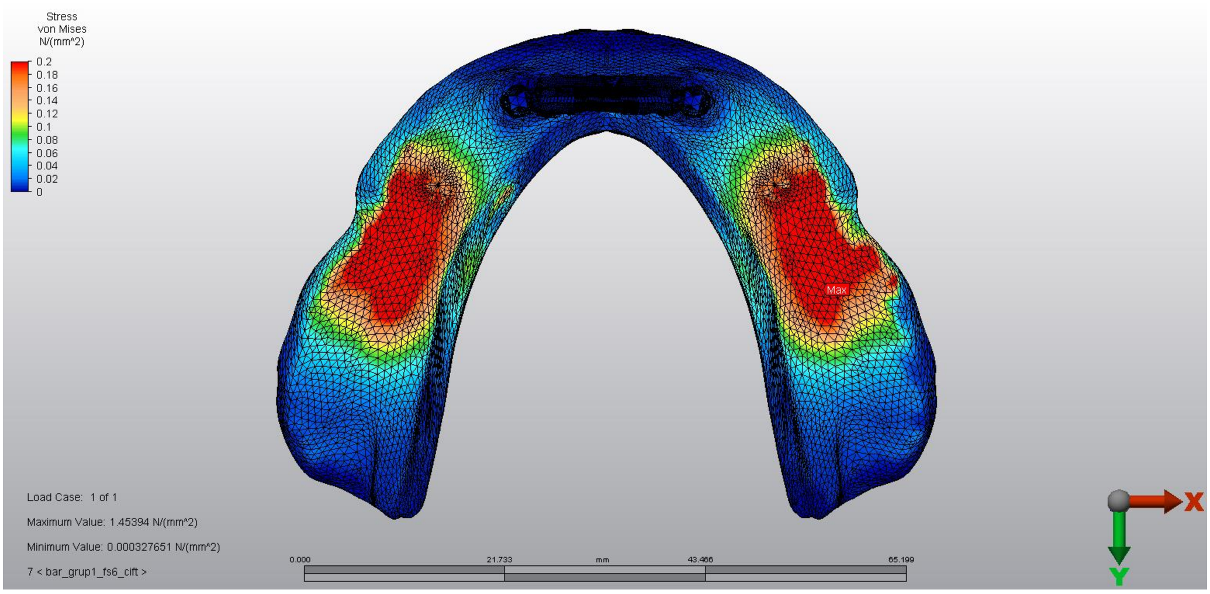
Stress
von Mises
N/(mm^2)
0.2
0.18
0.16
0.14
0.12
0.1
0.08
0.06
0.04
0.02
0
Max
Load Case: 1 of 1
Maximum Value: 1.45394 N/(mm^2)
Minimum Value: 0.000327651 N/(mm^2)
7 < bar_grup1_fs6_cift >
0.000
21.733
mm
43.466
65.199
X
Y

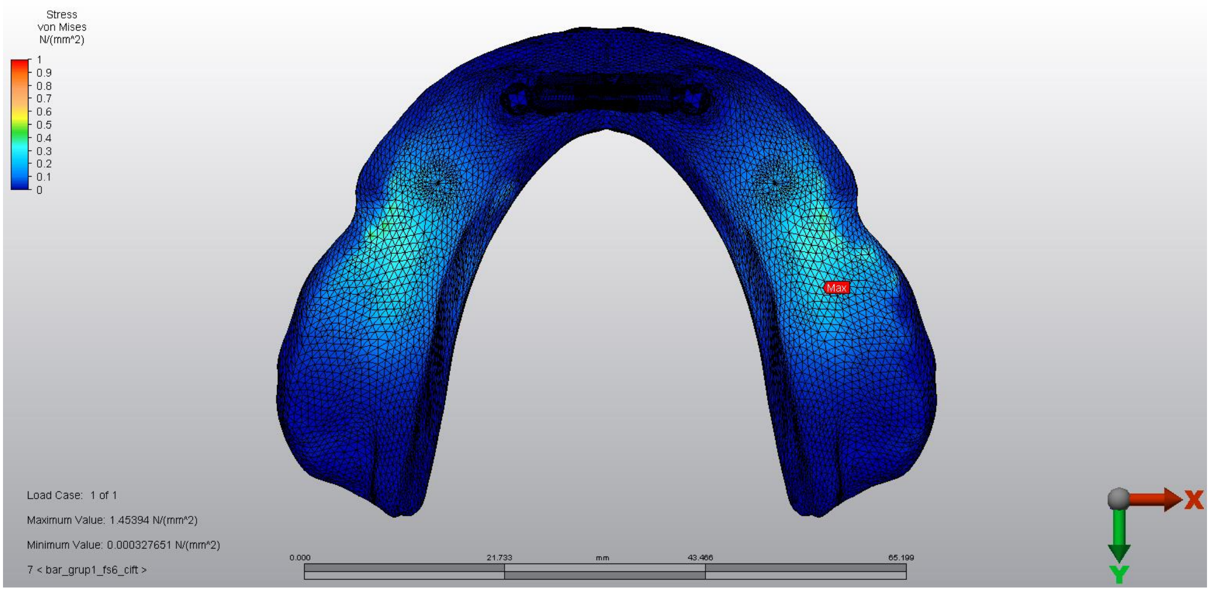
Stress
von Mises
N/(mm^2)
1
0.9
0.8
0.7
0.6
0.5
0.4
0.3
0.2
0.1
0
Max
Load Case: 1 of 1
Maximum Value: 1.45394 N/(mm^2)
Minimum Value: 0.000327651 N/(mm^2)
7 < bar_grup1_fs6_cift >
0.000
21.733
mm
43.466
65.199
X
Y

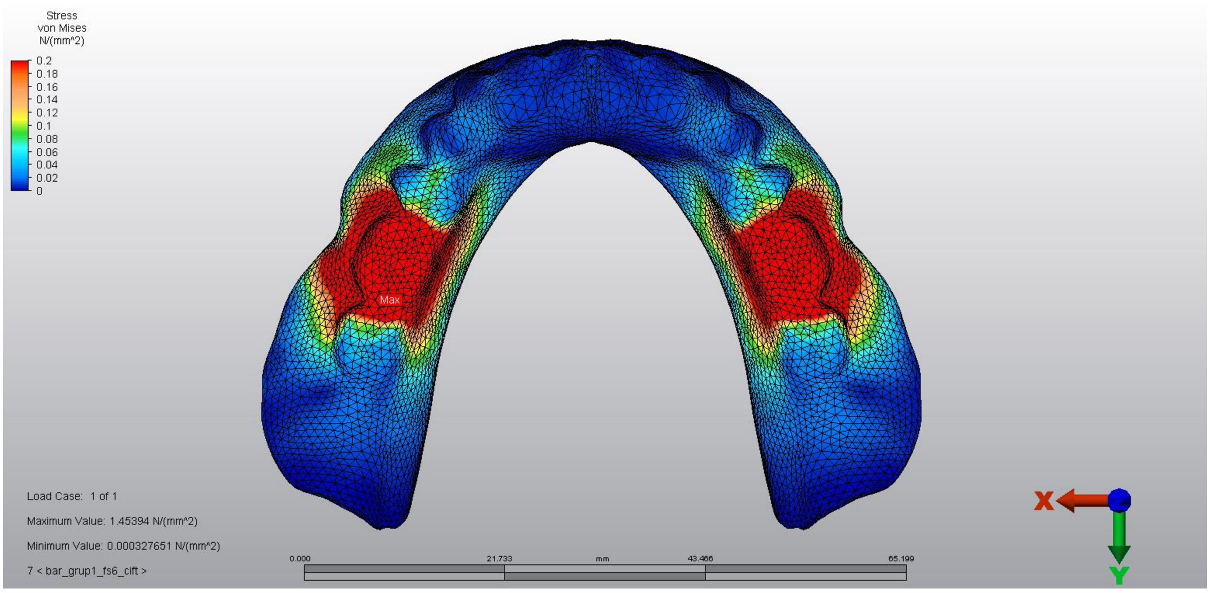
Stress
von Mises
N/(mm^2)
0.2
0.18
0.16
0.14
0.12
0.1
0.08
0.06
0.04
0.02
0
Max
Load Case: 1 of 1
Maximum Value: 1.45394 N/(mm^2)
Minimum Value: 0.000327651 N/(mm^2)
7 < bar_grup1_fs6_cift >
0.000
21.733
mm
43.466
65.199
X
Y

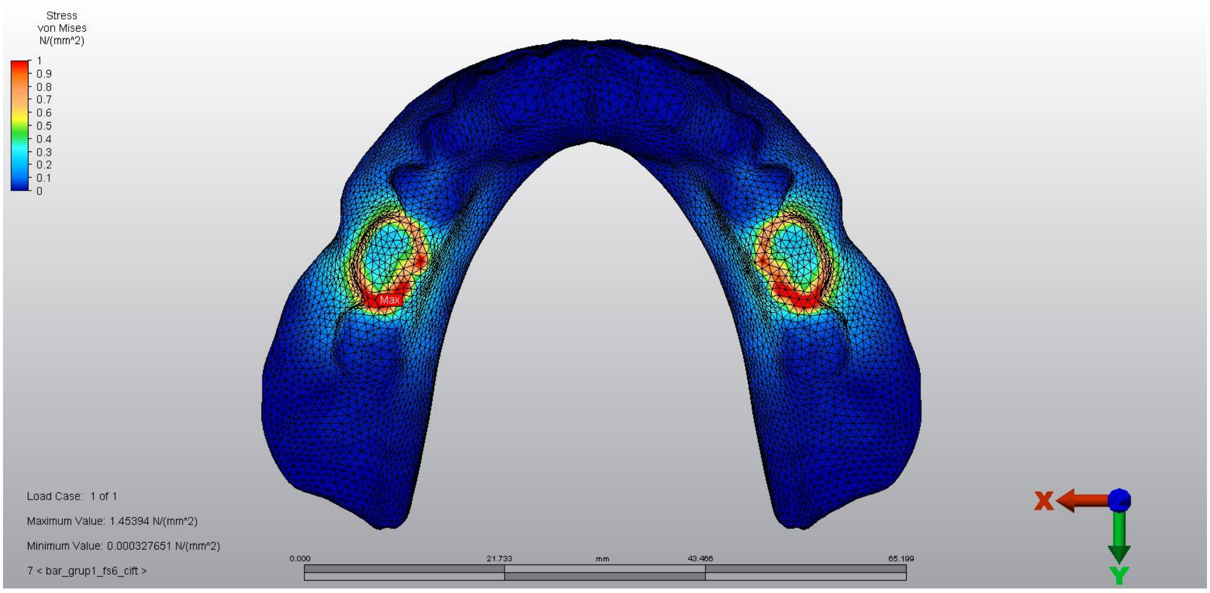
Stress
von Mises
N/(mm^2)
1
0.9
0.8
0.7
0.6
0.5
0.4
0.3
0.2
0.1
0
Max
Load Case: 1 of 1
Maximum Value: 1.45394 N/(mm^2)
Minimum Value: 0.000327651 N/(mm^2)
7 < bar_grup1_fs6_cift >
0.000
21.733
mm
43.466
65.199
X
Y

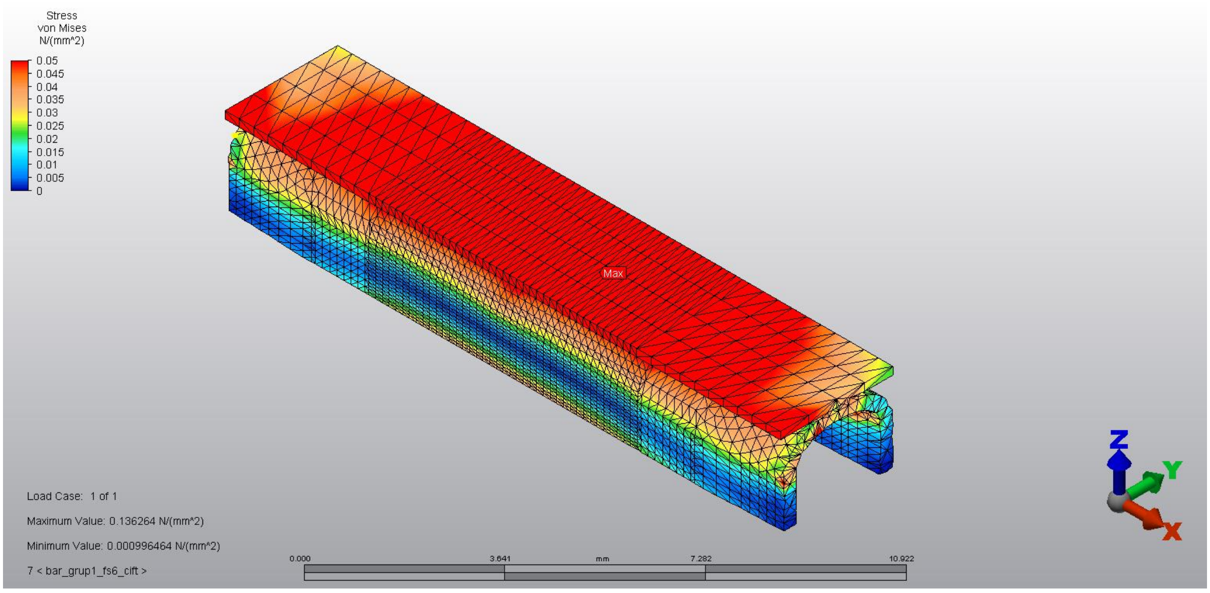
Stress
von Mises
N/(mm^2)
0.05
0.045
0.04
0.035
0.03
0.025
0.02
0.015
0.01
0.005
0
Max
Load Case: 1 of 1
Maximum Value: 0.136264 N/(mm^2)
Minimum Value: 0.000996464 N/(mm^2)
7 < bar_grup1_fs6_cift >
0.000
3.641
mm
7.282
10.922
Z
Y
X

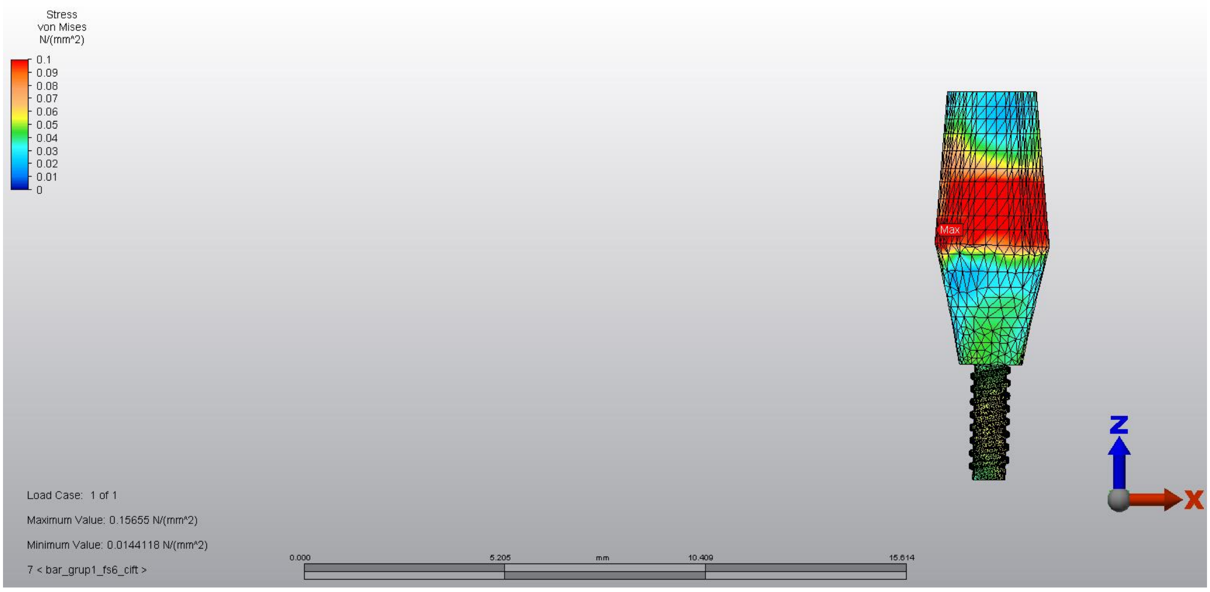
Stress
von Mises
N/(mm^2)
0.1
0.09
0.08
0.07
0.06
0.05
0.04
0.03
0.02
0.01
0
Max
Load Case: 1 of 1
Maximum Value: 0.15655 N/(mm^2)
Minimum Value: 0.0144118 N/(mm^2)
7 < bar_grup1_fs6_cift >
0.000
5.205
mm
10.409
15.614
Z
X

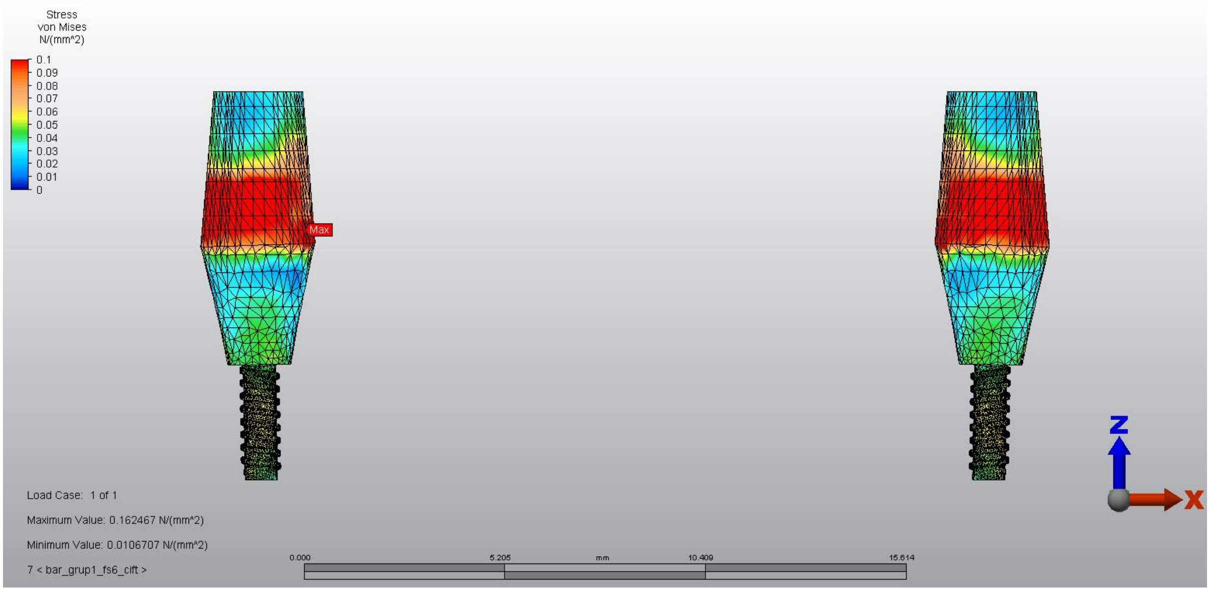
Stress
von Mises
N/(mm^2)
0.1
0.09
0.08
0.07
0.06
0.05
0.04
0.03
0.02
0.01
0
Max
Load Case: 1 of 1
Maximum Value: 0.162467 N/(mm^2)
Minimum Value: 0.0106707 N/(mm^2)
7 < bar_grup1_fs6_cift >
0.000
5.205
mm
10.409
15.614
Z
X

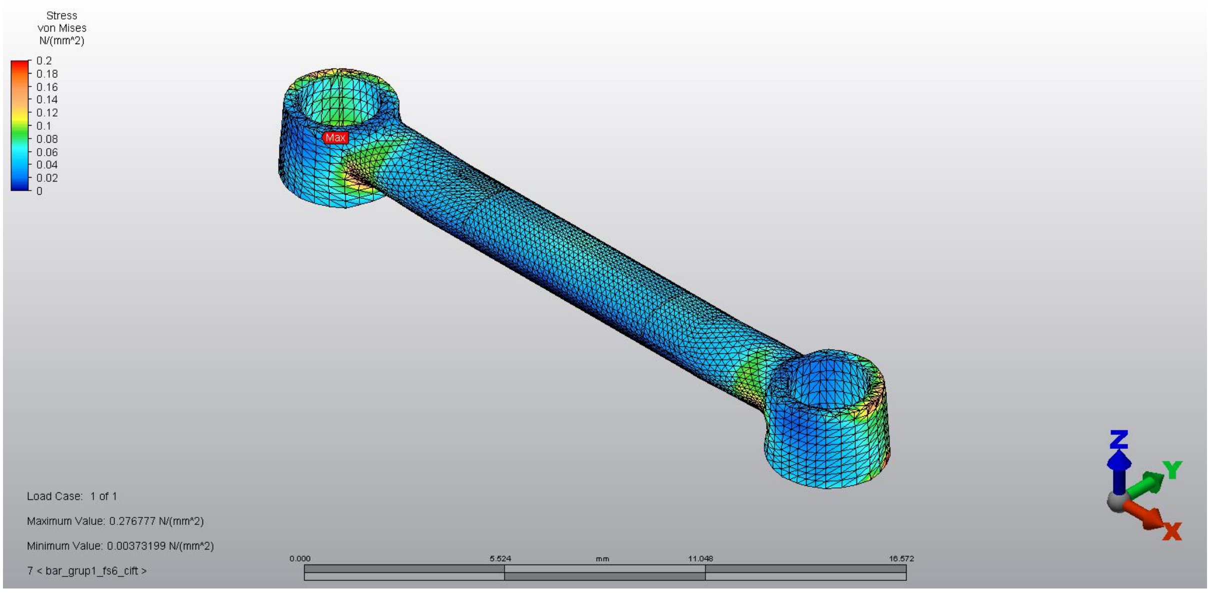

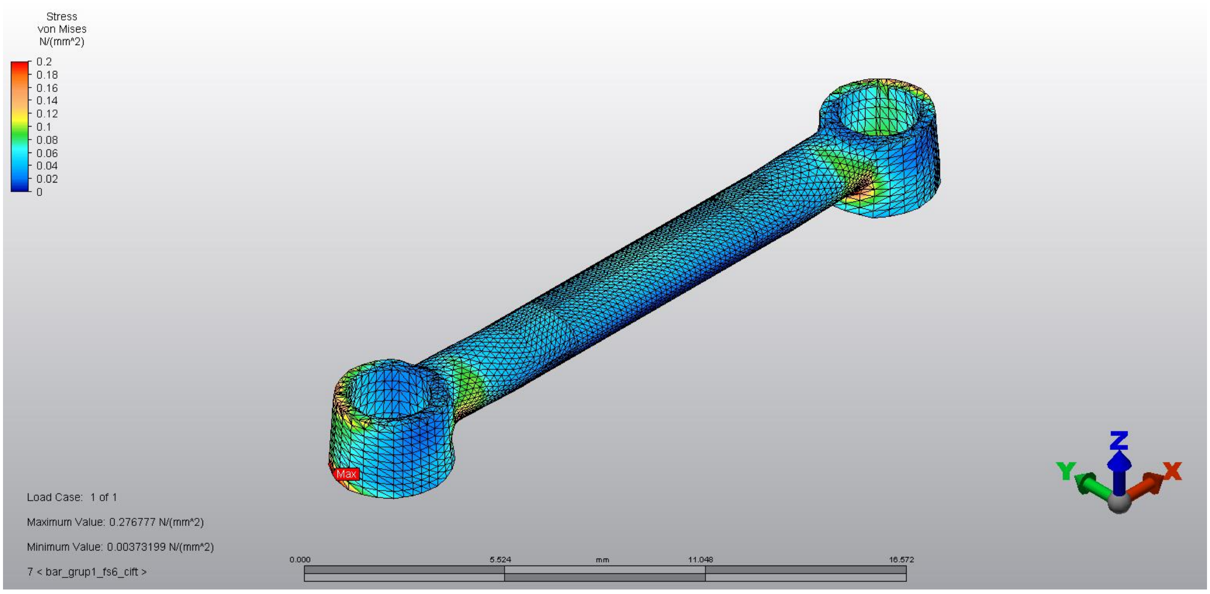

GRUP 02

INCISAL

Number of nodes = 157037

Number of elements = 781101

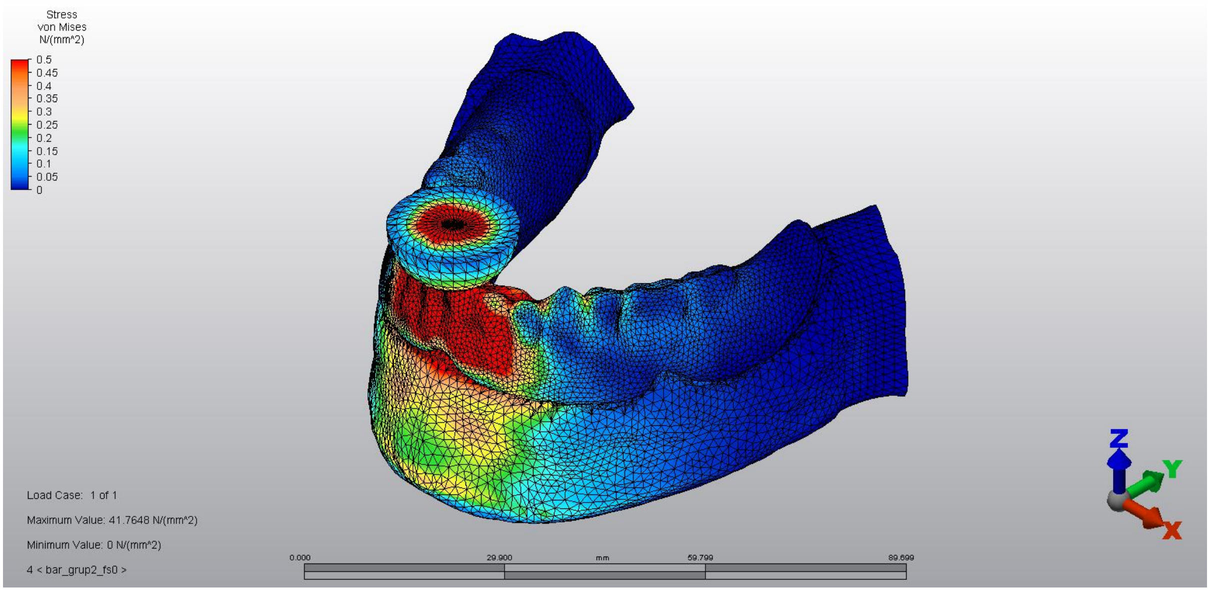
Stress
von Mises
N/(mm^2)
0.5
0.45
0.4
0.35
0.3
0.25
0.2
0.15
0.1
0.05
0
Load Case: 1 of 1
Maximum Value: 41.7648 N/(mm^2)
Minimum Value: 0 N/(mm^2)
4 < bar_grup2_fs0 >
0.000
29.900
mm
59.799
89.699
Z
Y
X

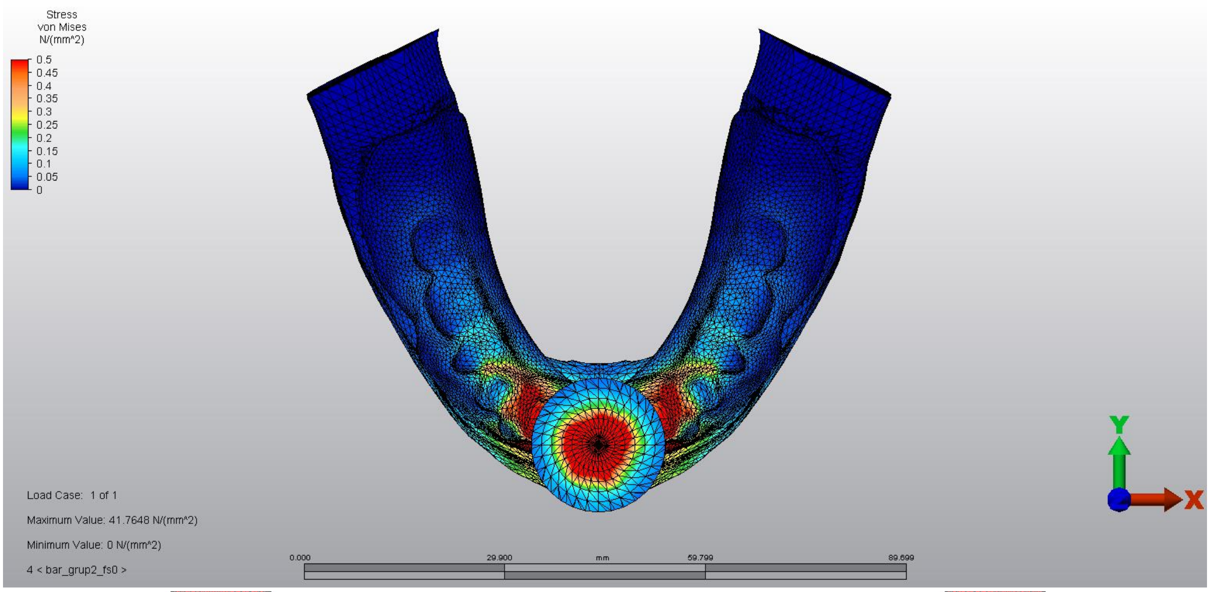
Stress
von Mises
N/(mm^2)
0.5
0.45
0.4
0.35
0.3
0.25
0.2
0.15
0.1
0.05
0
Load Case: 1 of 1
Maximum Value: 41.7648 N/(mm^2)
Minimum Value: 0 N/(mm^2)
4 < bar_grup2_fs0 >
0.000
29.900
mm
59.799
89.699
Y
X

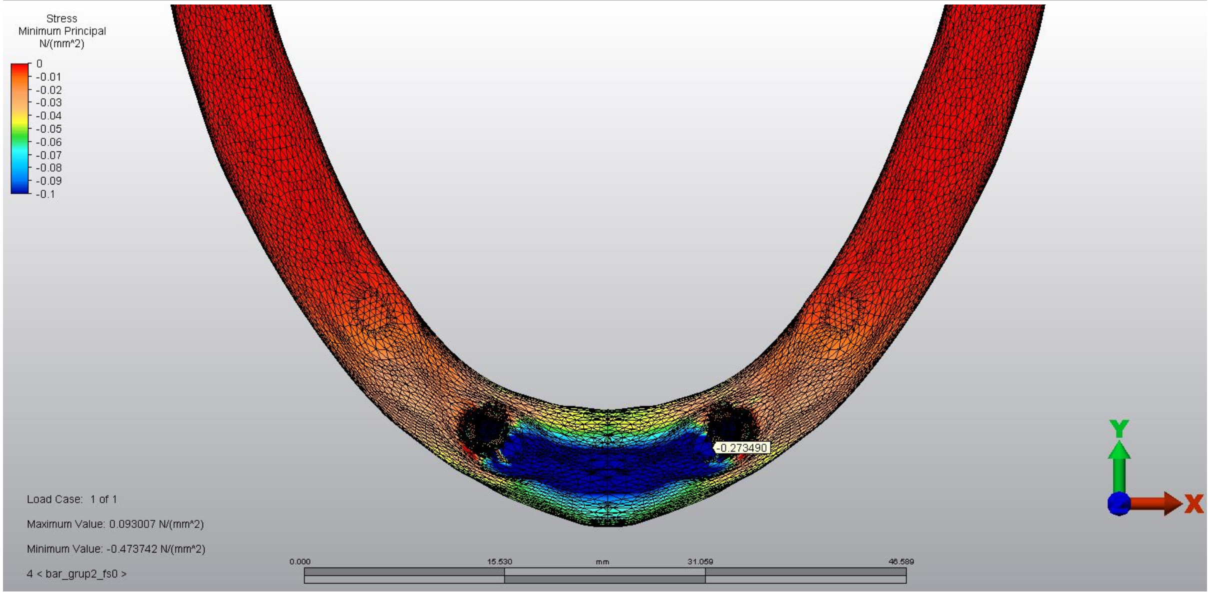
Stress
Minimum Principal
N/(mm^2)
0
-0.01
-0.02
-0.03
-0.04
-0.05
-0.06
-0.07
-0.08
-0.09
-0.1
-0.273490
Load Case: 1 of 1
Maximum Value: 0.093007 N/(mm^2)
Minimum Value: -0.473742 N/(mm^2)
4 < bar_grup2_fs0 >
0.000
15.530
mm
31.059
46.589
Y
X

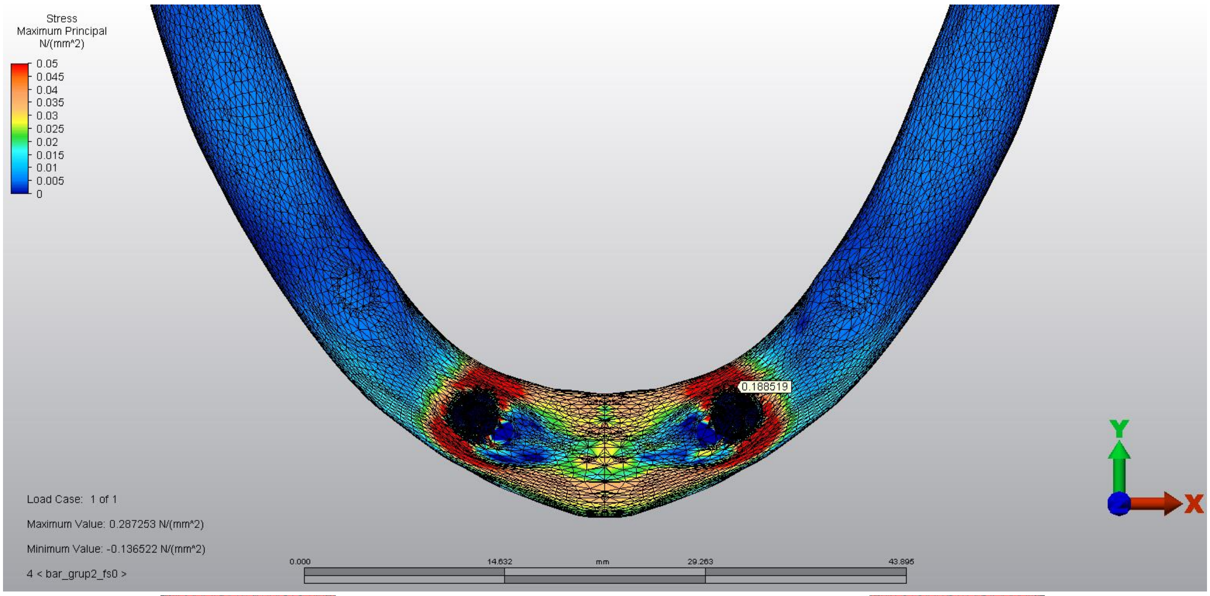
Stress
Maximum Principal
N/(mm^2)
0.05
0.045
0.04
0.035
0.03
0.025
0.02
0.015
0.01
0.005
0
0.188519
Load Case: 1 of 1
Maximum Value: 0.287253 N/(mm^2)
Minimum Value: -0.136522 N/(mm^2)
4 < bar_grup2_fs0 >
0.000
14.632
mm
29.263
43.895
Y
X

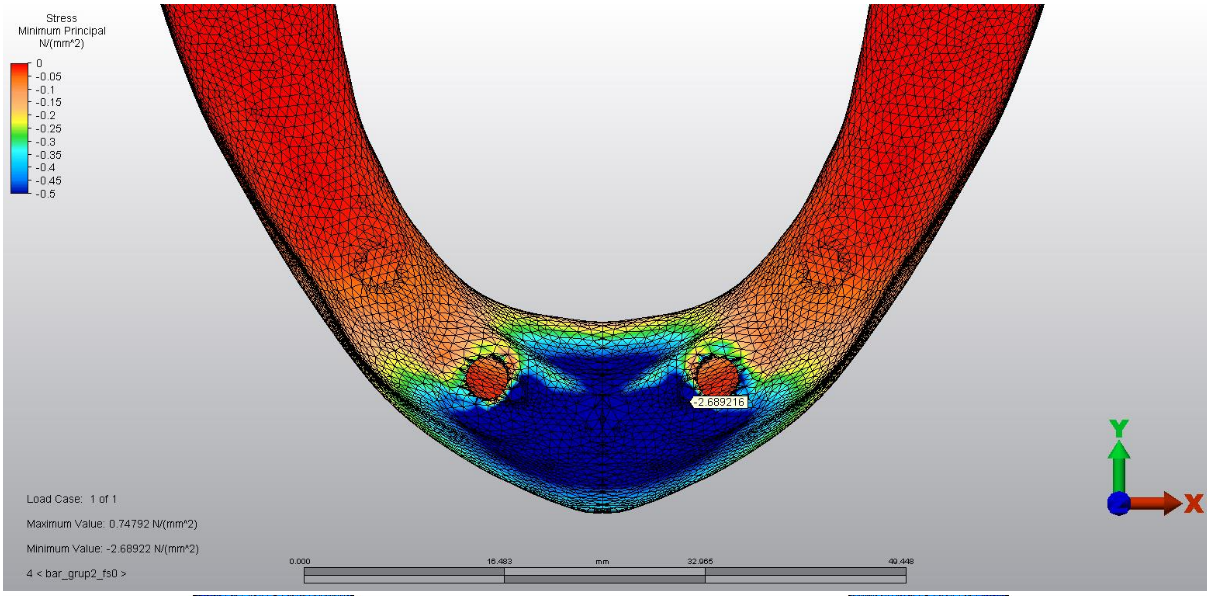
Stress
Minimum Principal
N/(mm^2)
0
-0.05
-0.1
-0.15
-0.2
-0.25
-0.3
-0.35
-0.4
-0.45
-0.5
-2.689216
Load Case: 1 of 1
Maximum Value: 0.74792 N/(mm^2)
Minimum Value: -2.68922 N/(mm^2)
4 < bar_grup2_fs0 >
0.000
16.483
mm
32.965
49.448
Y
X

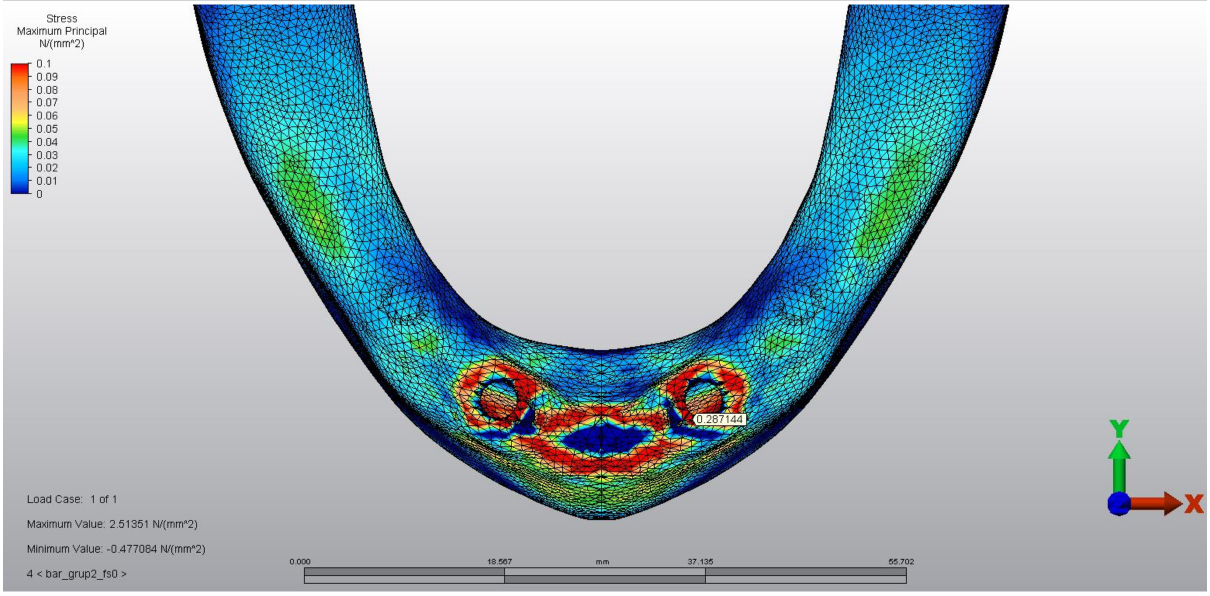
Stress
Maximum Principal
N/(mm^2)
0.1
0.09
0.08
0.07
0.06
0.05
0.04
0.03
0.02
0.01
0
0.287144
Load Case: 1 of 1
Maximum Value: 2.51351 N/(mm^2)
Minimum Value: -0.477084 N/(mm^2)
4 < bar_grup2_fs0 >
0.000
18.567
mm
37.135
55.702
Y
X

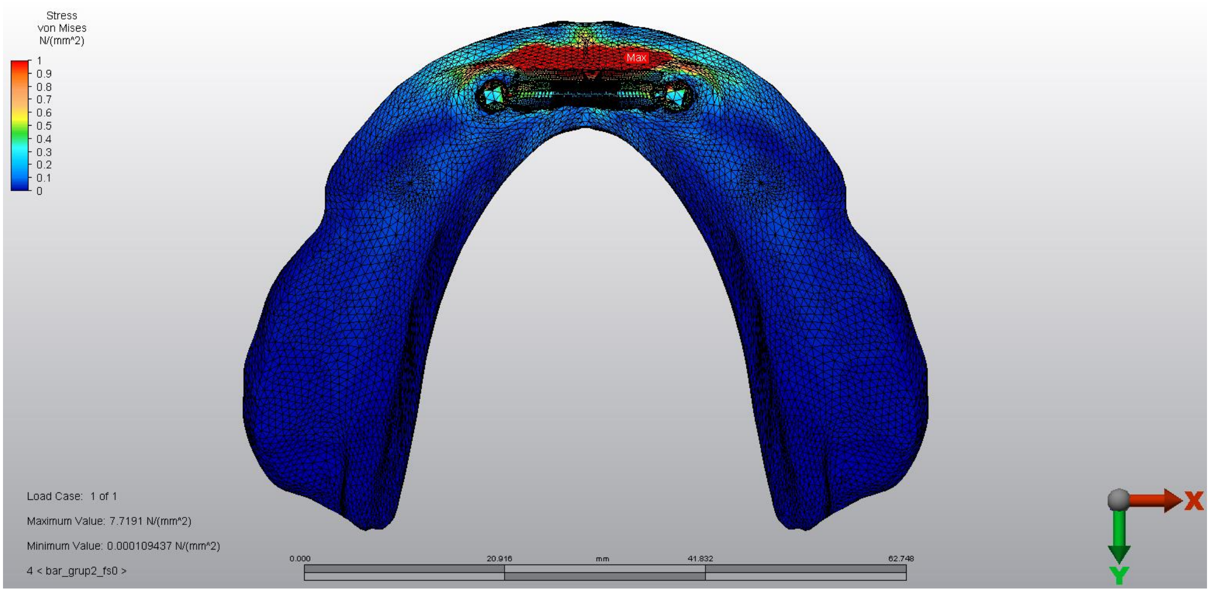
Stress
von Mises
N/(mm^2)
Max
Load Case: 1 of 1
Maximum Value: 7.7191 N/(mm^2)
Minimum Value: 0.000109437 N/(mm^2)
4 < bar_grup2_fs0 >
0.000
20.916
mm
41.832
62.748
X
Y

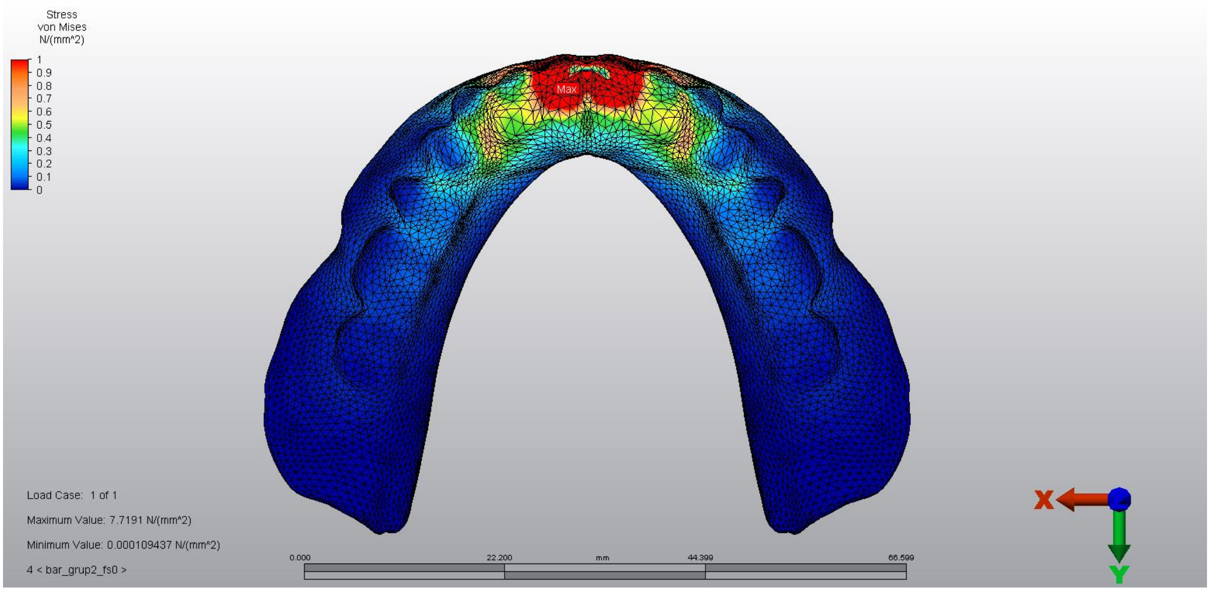
Stress
von Mises
N/(mm^2)
Max
Load Case: 1 of 1
Maximum Value: 7.7191 N/(mm^2)
Minimum Value: 0.000109437 N/(mm^2)
4 < bar_grup2_fs0 >
0.000
22.200
mm
44.399
66.599
X
Y

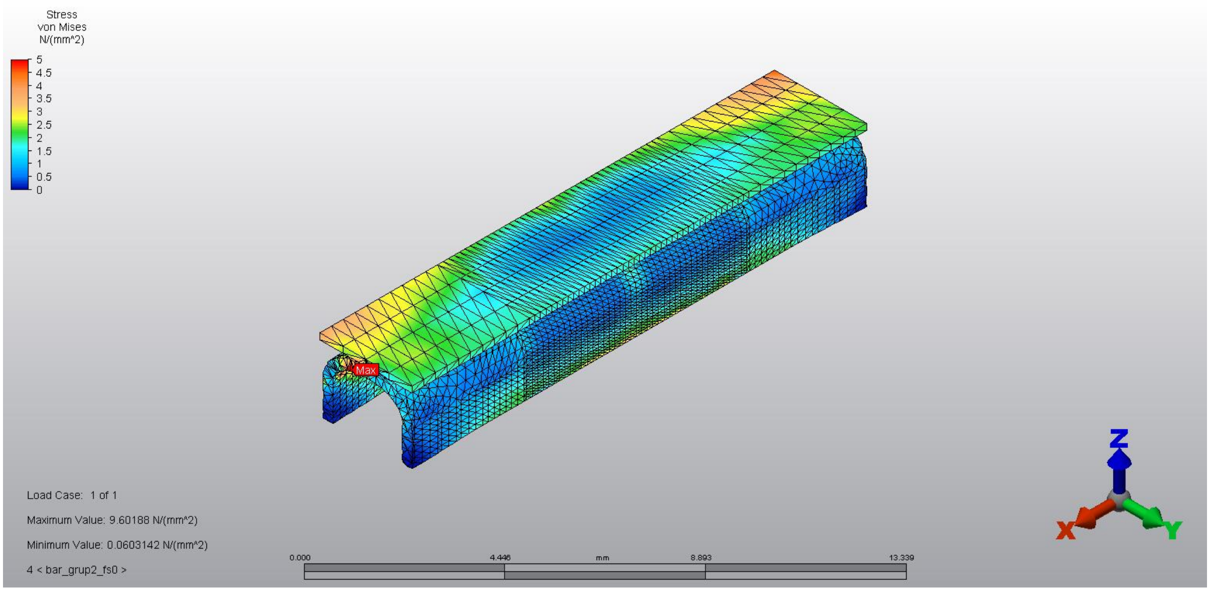
Stress
von Mises
N/(mm^2)
Max
Load Case: 1 of 1
Maximum Value: 9.60188 N/(mm^2)
Minimum Value: 0.0603142 N/(mm^2)
4 < bar_grup2_fs0 >
0.000
4.446
mm
8.893
13.339
Z
X
Y

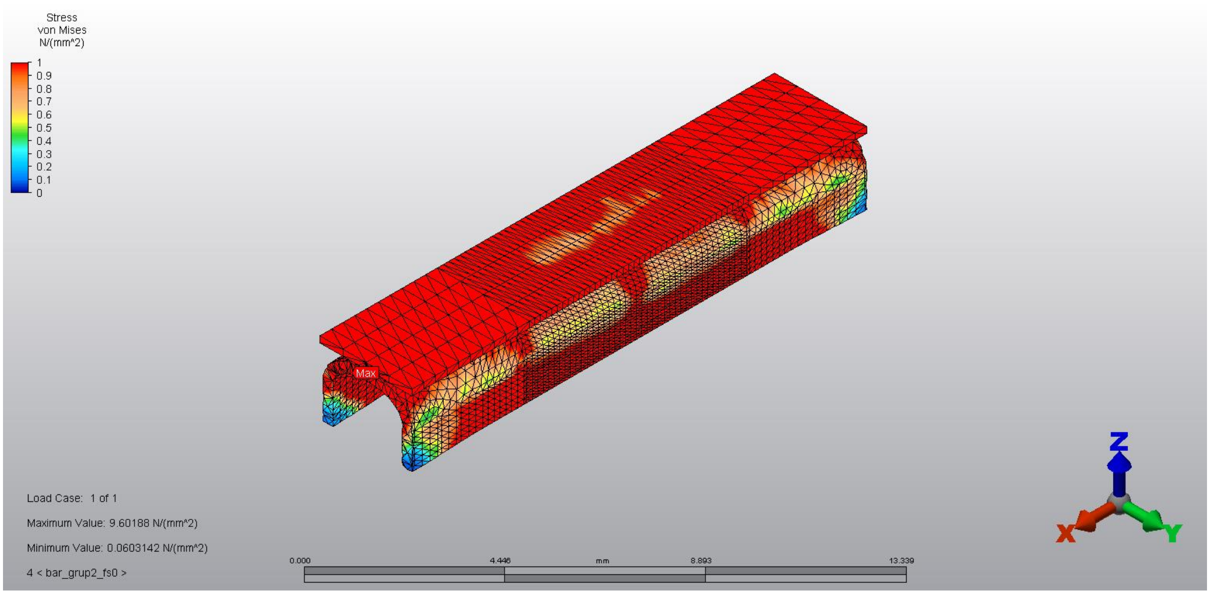
Stress
von Mises
N/(mm^2)
Max
Load Case: 1 of 1
Maximum Value: 9.60188 N/(mm^2)
Minimum Value: 0.0603142 N/(mm^2)
4 < bar_grup2_fs0 >
0.000
4.446
mm
8.893
13.339
Z
X
Y

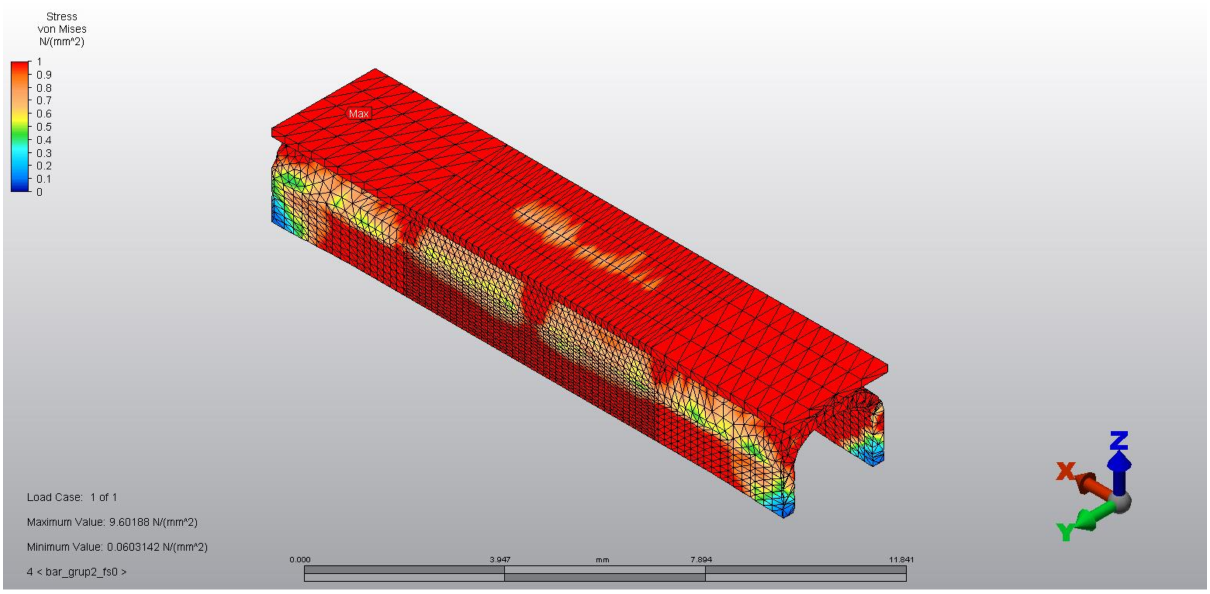
Stress
von Mises
N/(mm^2)
Max
Load Case: 1 of 1
Maximum Value: 9.60188 N/(mm^2)
Minimum Value: 0.0603142 N/(mm^2)
4 < bar_grup2_fs0 >
0.000
3.947
mm
7.894
11.841
Z
X
Y

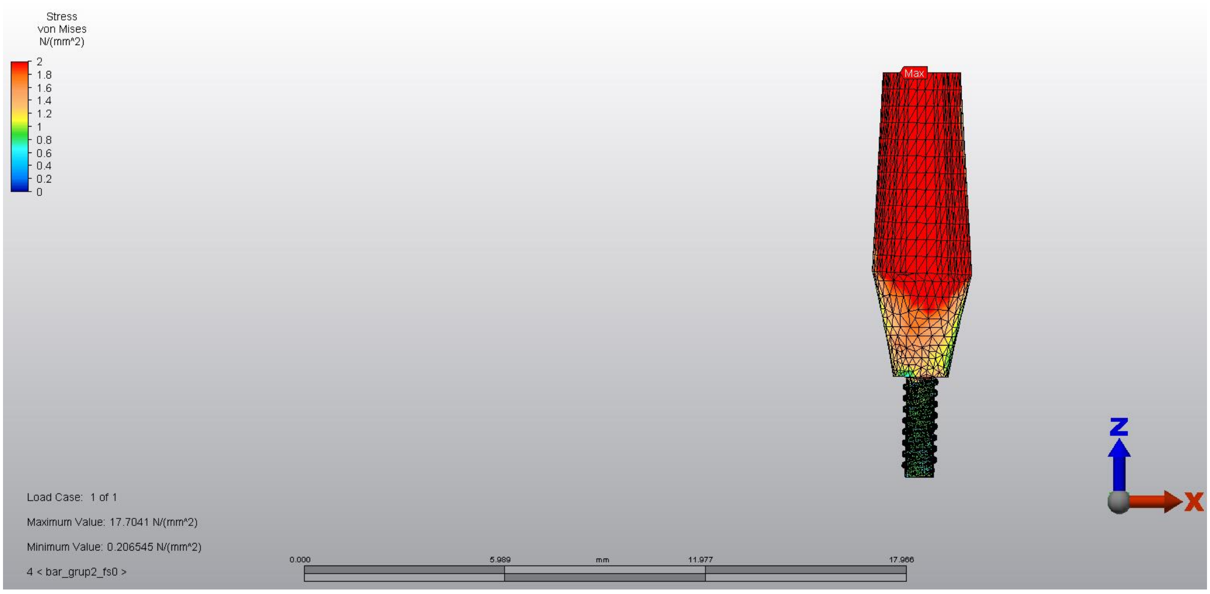
Stress
von Mises
N/(mm^2)
Max
Load Case: 1 of 1
Maximum Value: 17.7041 N/(mm^2)
Minimum Value: 0.206545 N/(mm^2)
4 < bar_grup2_fs0 >
0.000
5.989
mm
11.977
17.966
Z
X

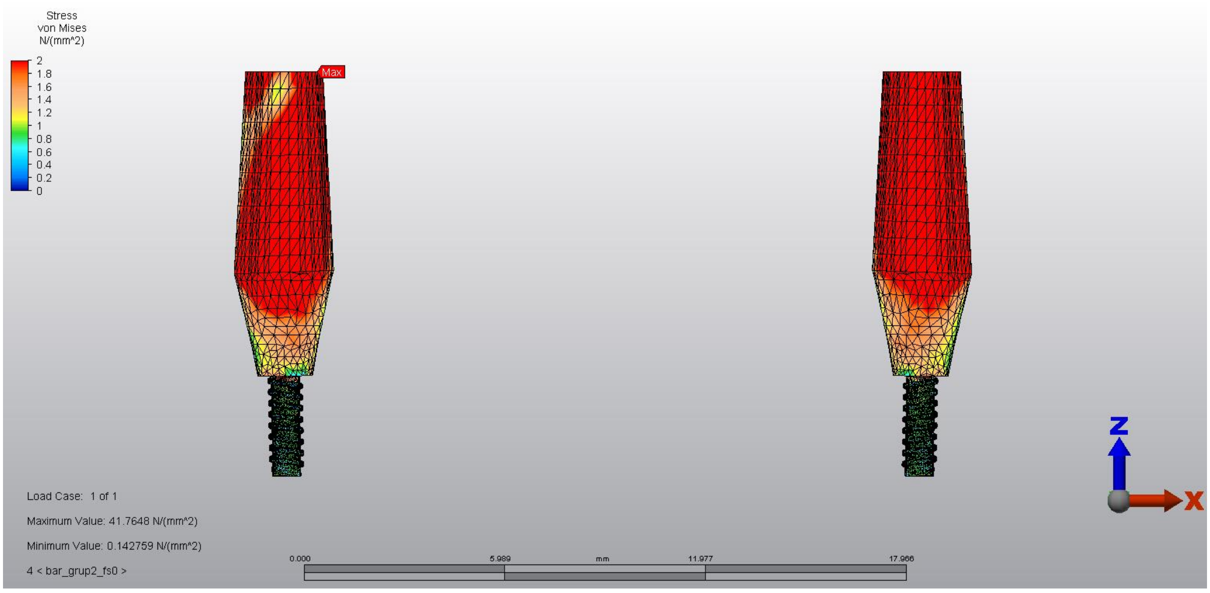
Stress
von Mises
N/(mm^2)
2
1.8
1.6
1.4
1.2
1
0.8
0.6
0.4
0.2
0
Max
Load Case: 1 of 1
Maximum Value: 41.7648 N/(mm^2)
Minimum Value: 0.142759 N/(mm^2)
4 < bar_grup2_fs0 >
0.000
5.989
mm
11.977
17.966
Z
X

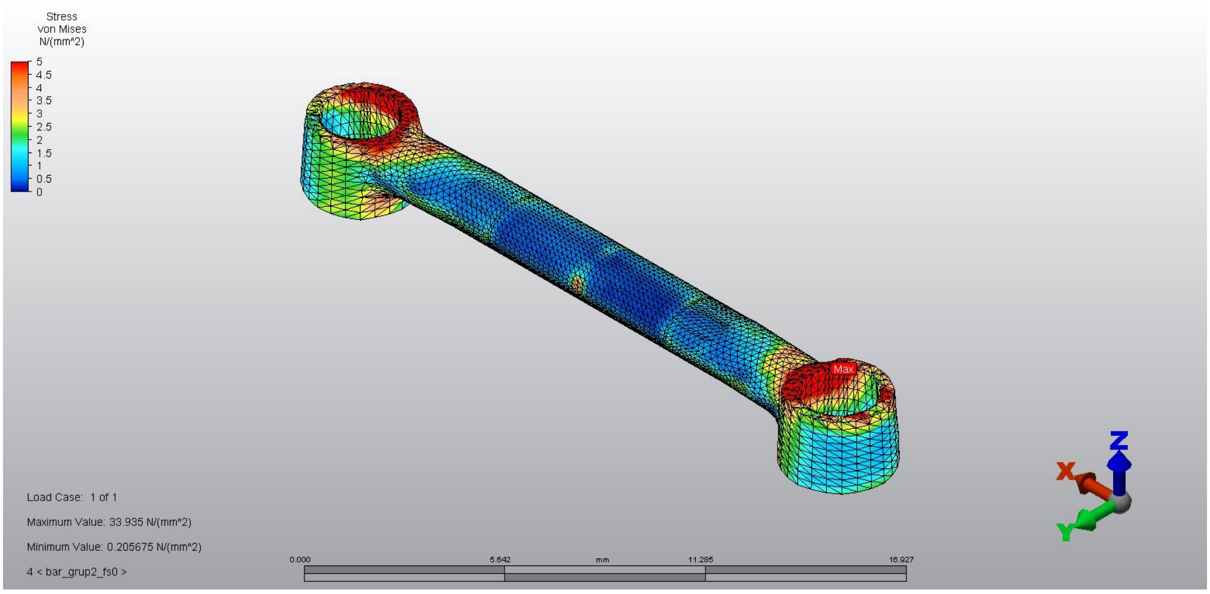
Stress
von Mises
N/(mm^2)
5
4.5
4
3.5
3
2.5
2
1.5
1
0.5
0
Max
Load Case: 1 of 1
Maximum Value: 33.935 N/(mm^2)
Minimum Value: 0.205675 N/(mm^2)
4 < bar_grup2_fs0 >
0.000
5.642
mm
11.285
16.927
Z
X
Y

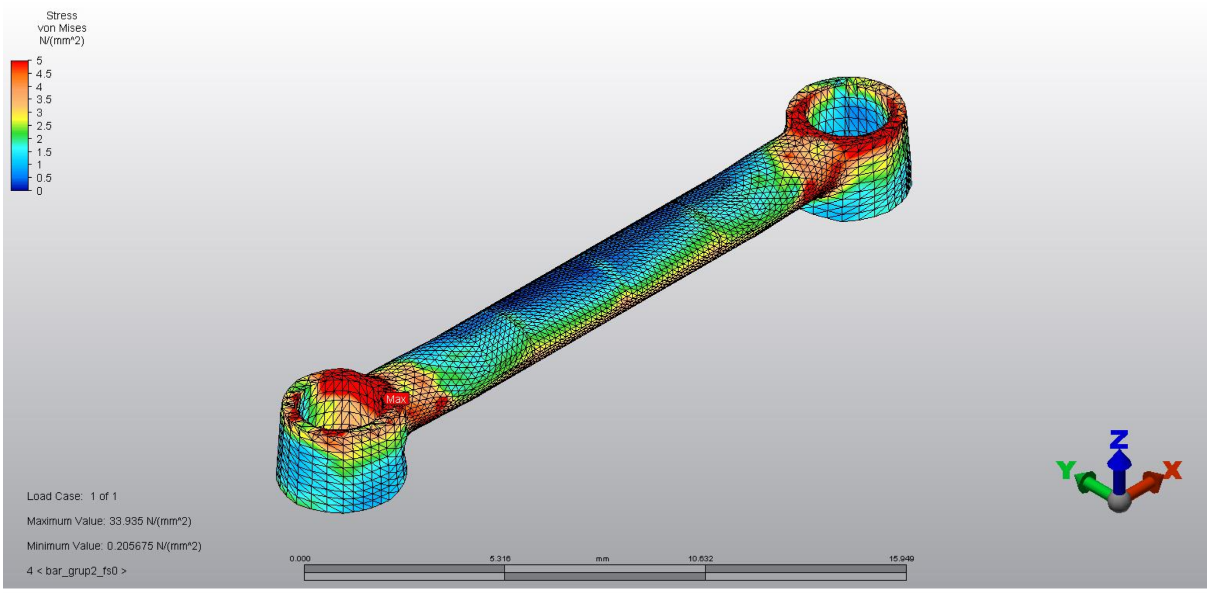

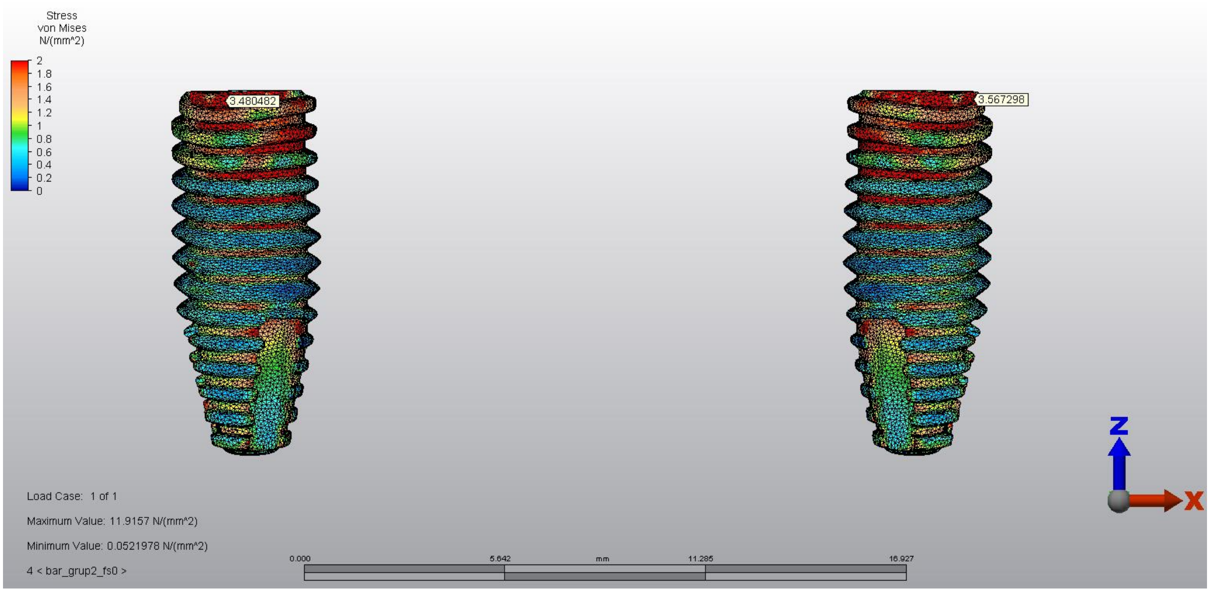

UNILATERAL

Number of nodes = 157004

Number of elements = 781081

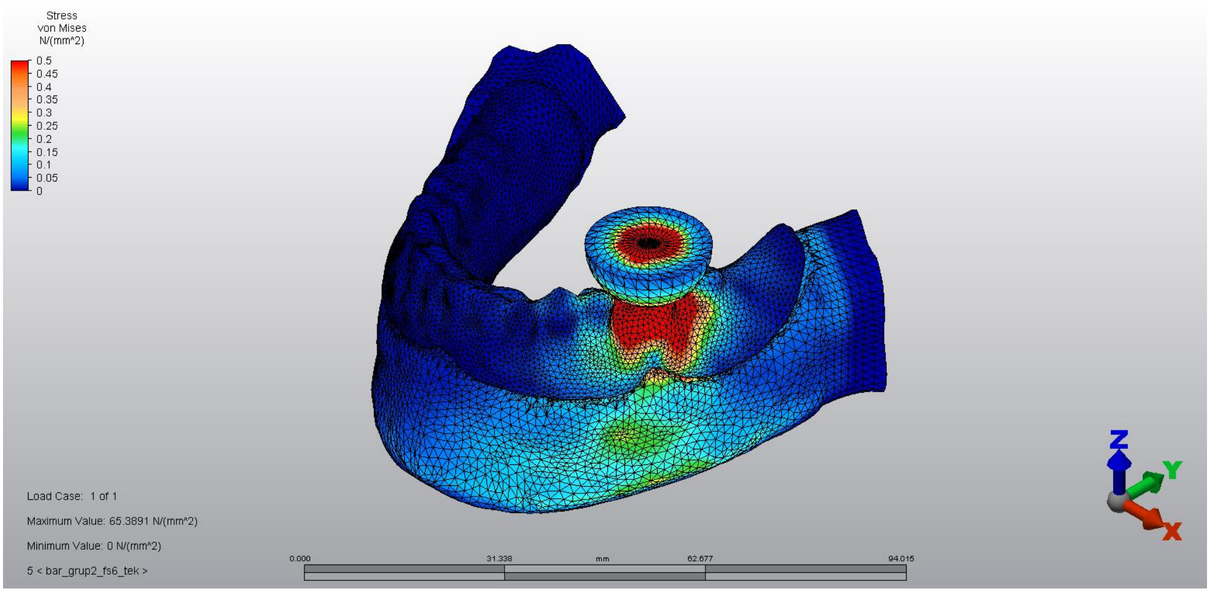
Stress
von Mises
N/(mm^2)
0.5
0.45
0.4
0.35
0.3
0.25
0.2
0.15
0.1
0.05
0
Load Case: 1 of 1
Maximum Value: 65.3891 N/(mm^2)
Minimum Value: 0 N/(mm^2)
5 < bar_grup2_fs6_tek >
0.000
31.338
mm
62.677
94.015
Z
Y
X

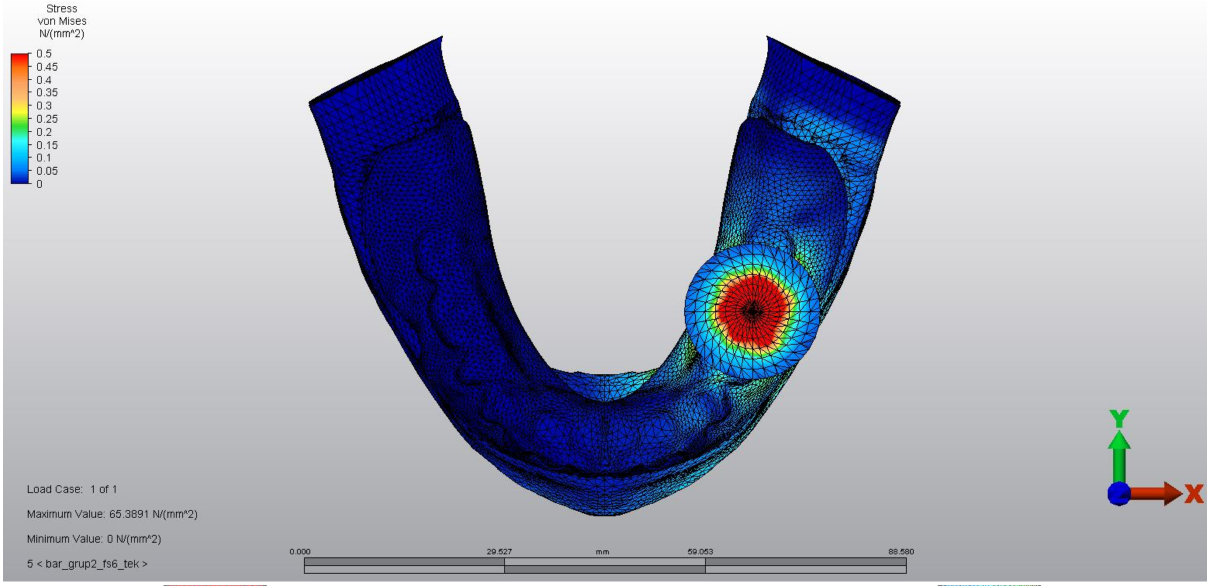
Stress
von Mises
N/(mm^2)
0.5
0.45
0.4
0.35
0.3
0.25
0.2
0.15
0.1
0.05
0
Load Case: 1 of 1
Maximum Value: 65.3891 N/(mm^2)
Minimum Value: 0 N/(mm^2)
5 < bar_grup2_fs6_tek >
0.000
29.527
mm
59.053
88.580
Y
X

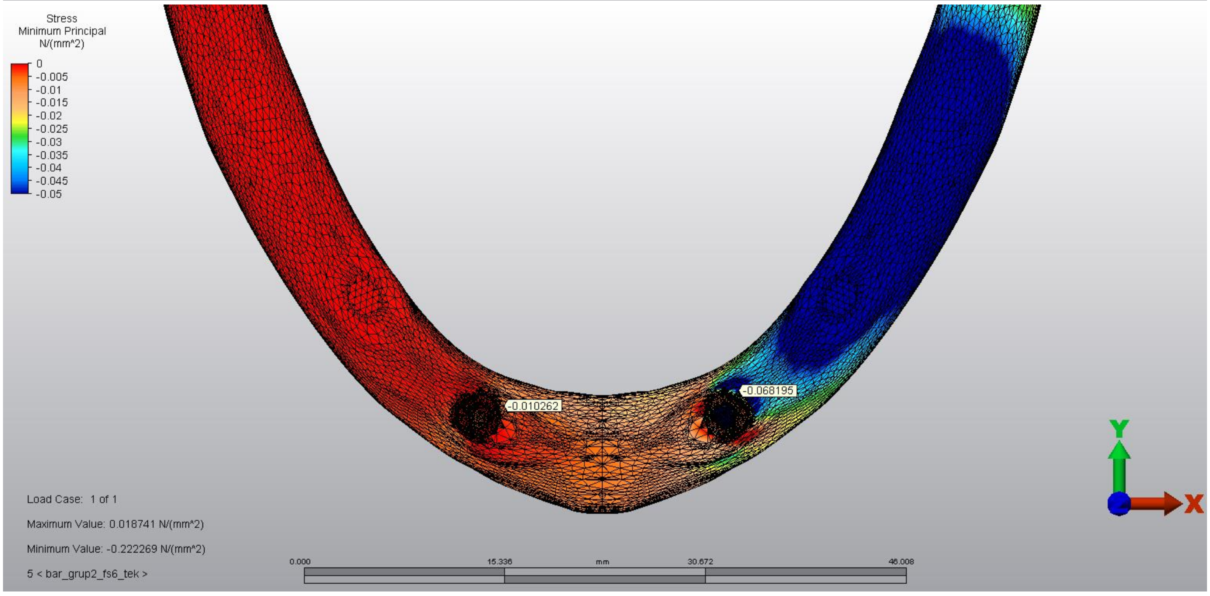
Stress
Minimum Principal
N/(mm^2)
0
-0.005
-0.01
-0.015
-0.02
-0.025
-0.03
-0.035
-0.04
-0.045
-0.05
-0.010262
-0.068195
Load Case: 1 of 1
Maximum Value: 0.018741 N/(mm^2)
Minimum Value: -0.222269 N/(mm^2)
5 < bar_grup2_fs6_tek >
0.000
15.336
mm
30.672
46.008
Y
X

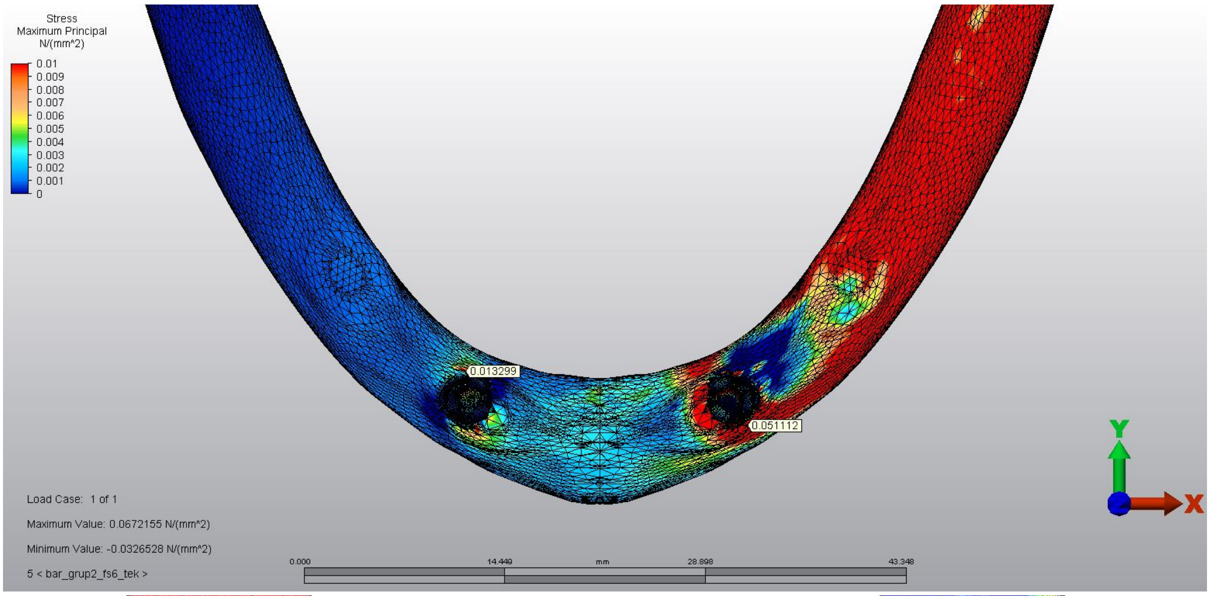
Stress
Maximum Principal
N/(mm^2)
0.01
0.009
0.008
0.007
0.006
0.005
0.004
0.003
0.002
0.001
0
0.013299
0.051112
Load Case: 1 of 1
Maximum Value: 0.0672155 N/(mm^2)
Minimum Value: -0.0326528 N/(mm^2)
5 < bar_grup2_fs6_tek >
0.000
14.449
mm
28.898
43.348
Y
X

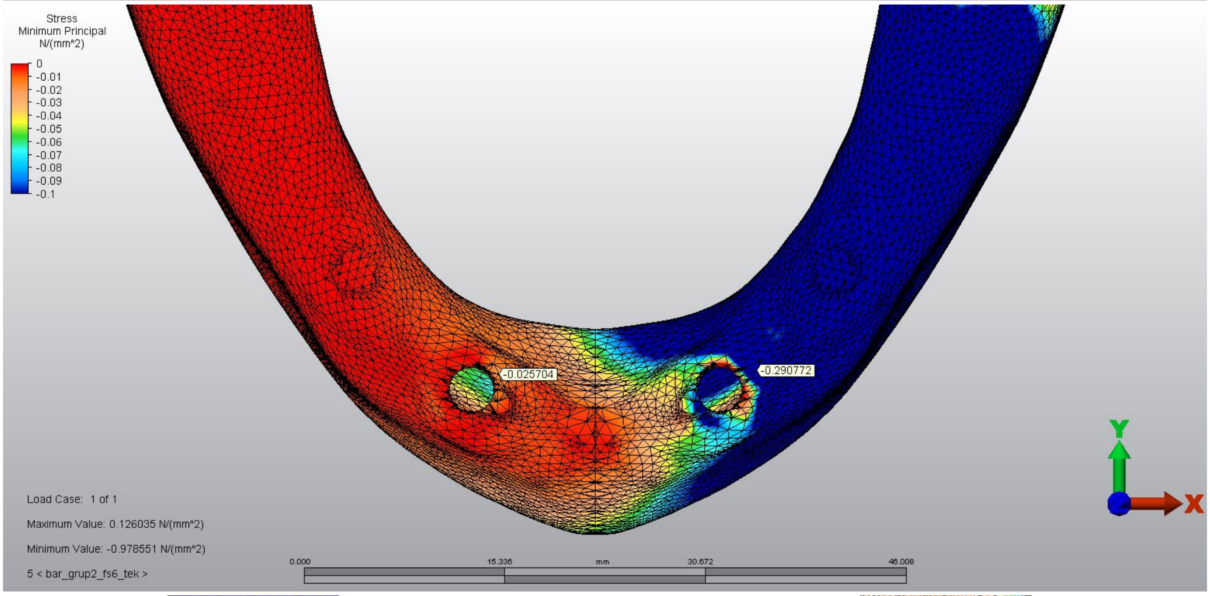
Stress
Minimum Principal
N/(mm^2)
0
-0.01
-0.02
-0.03
-0.04
-0.05
-0.06
-0.07
-0.08
-0.09
-0.1
-0.025704
-0.290772
Load Case: 1 of 1
Maximum Value: 0.126035 N/(mm^2)
Minimum Value: -0.978551 N/(mm^2)
5 < bar_grup2_fs6_tek >
0.000
15.336
mm
30.672
46.008
Y
X

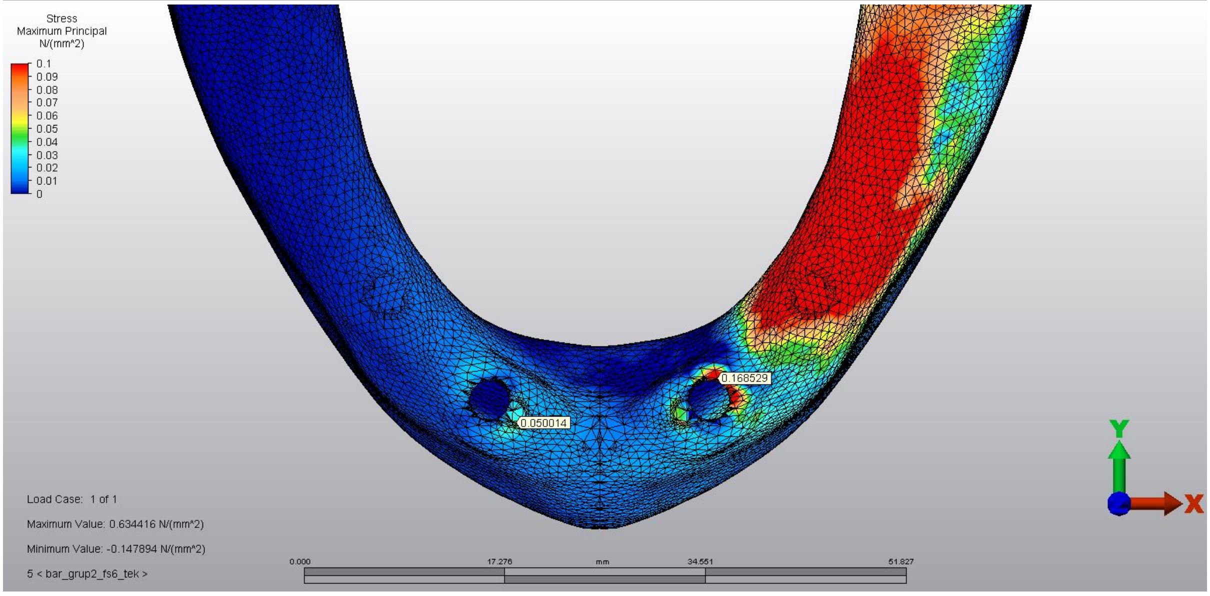
Stress
Maximum Principal
N/(mm^2)
0.1
0.09
0.08
0.07
0.06
0.05
0.04
0.03
0.02
0.01
0
0.168529
0.050014
Load Case: 1 of 1
Maximum Value: 0.634416 N/(mm^2)
Minimum Value: -0.147894 N/(mm^2)
5 < bar_grup2_fs6_tek >
0.000
17.276
mm
34.551
51.827
Y
X

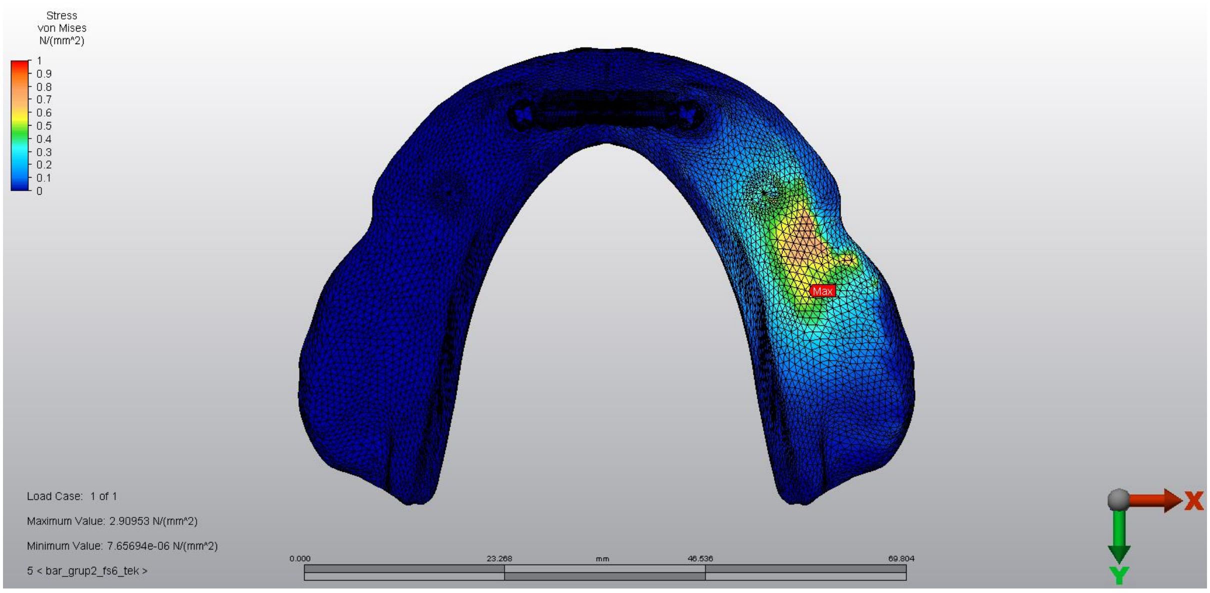
Stress
von Mises
N/(mm^2)
1
0.9
0.8
0.7
0.6
0.5
0.4
0.3
0.2
0.1
0
Max
Load Case: 1 of 1
Maximum Value: 2.90953 N/(mm^2)
Minimum Value: 7.65694e-06 N/(mm^2)
5 < bar_grup2_fs6_tek >
0.000
23.268
mm
46.536
69.804
X
Y

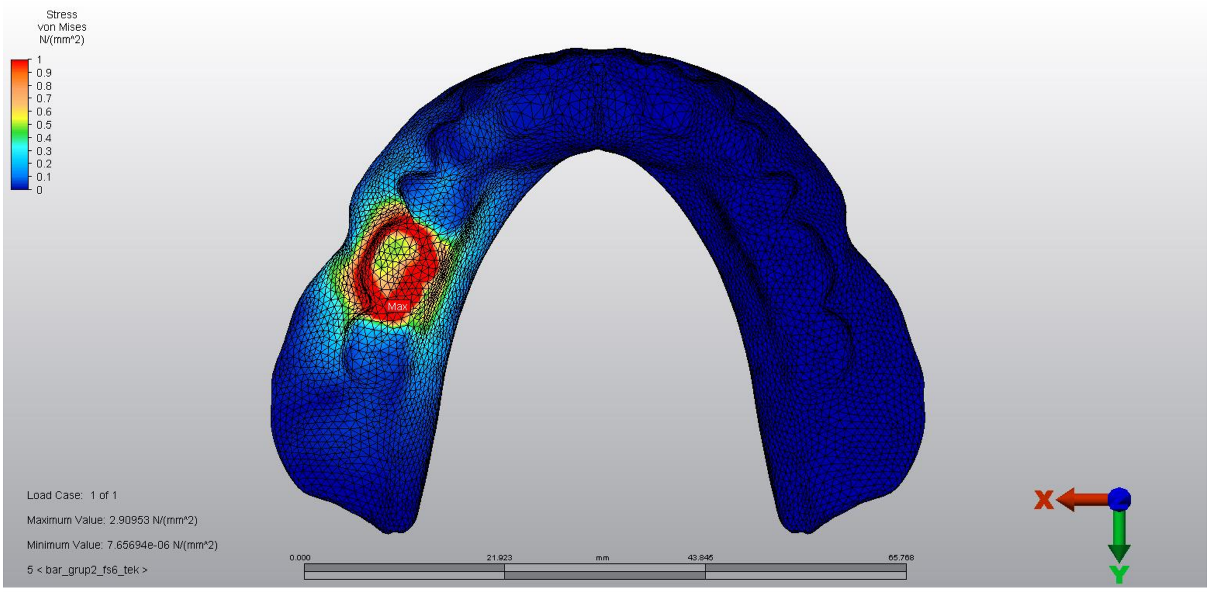
Stress
von Mises
N/(mm^2)
1
0.9
0.8
0.7
0.6
0.5
0.4
0.3
0.2
0.1
0
Max
Load Case: 1 of 1
Maximum Value: 2.90953 N/(mm^2)
Minimum Value: 7.65694e-06 N/(mm^2)
5 < bar_grup2_fs6_tek >
0.000
21.923
mm
43.846
65.768
X
Y

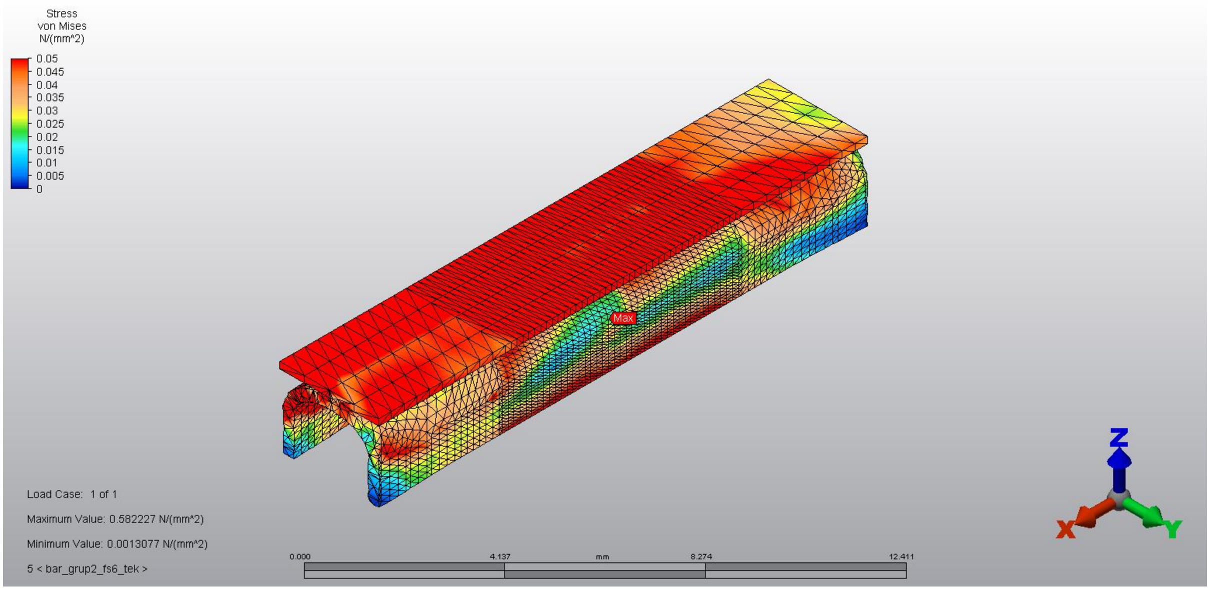
Stress
von Mises
N/(mm^2)
0.05
0.045
0.04
0.035
0.03
0.025
0.02
0.015
0.01
0.005
0
Max
Load Case: 1 of 1
Maximum Value: 0.582227 N/(mm^2)
Minimum Value: 0.0013077 N/(mm^2)
5 < bar_grup2_fs6_tek >
0.000
4.137
mm
8.274
12.411
Z
X
Y

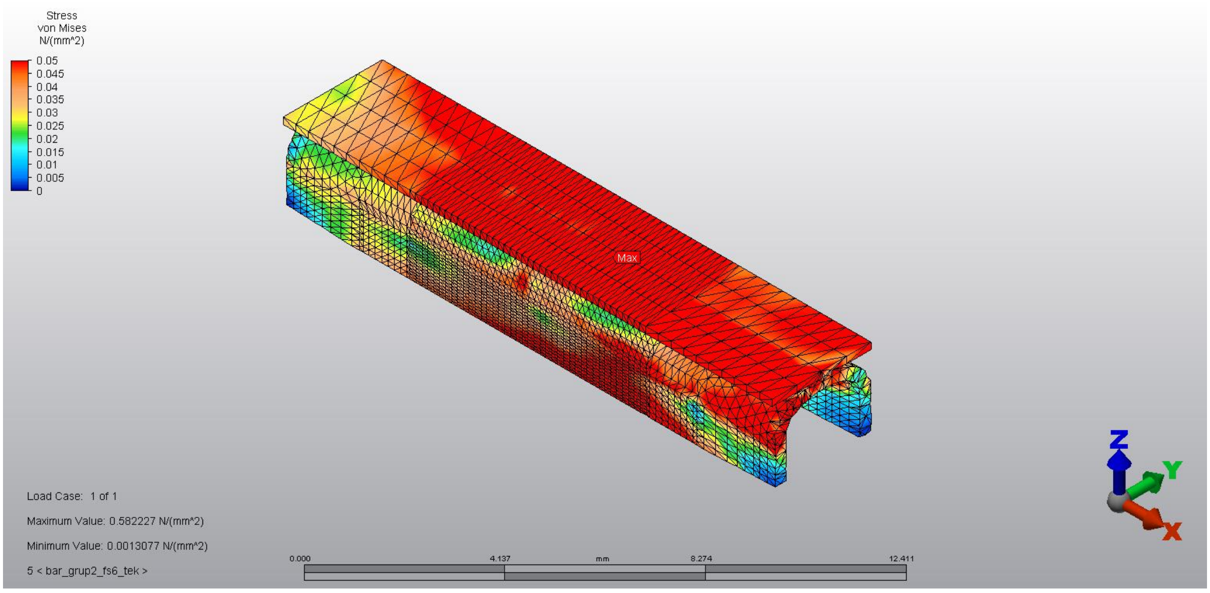
Stress
von Mises
N/(mm^2)
0.05
0.045
0.04
0.035
0.03
0.025
0.02
0.015
0.01
0.005
0
Max
Load Case: 1 of 1
Maximum Value: 0.582227 N/(mm^2)
Minimum Value: 0.0013077 N/(mm^2)
5 < bar_grup2_fs6_tek >
0.000
4.137
mm
8.274
12.411
Z
Y
X

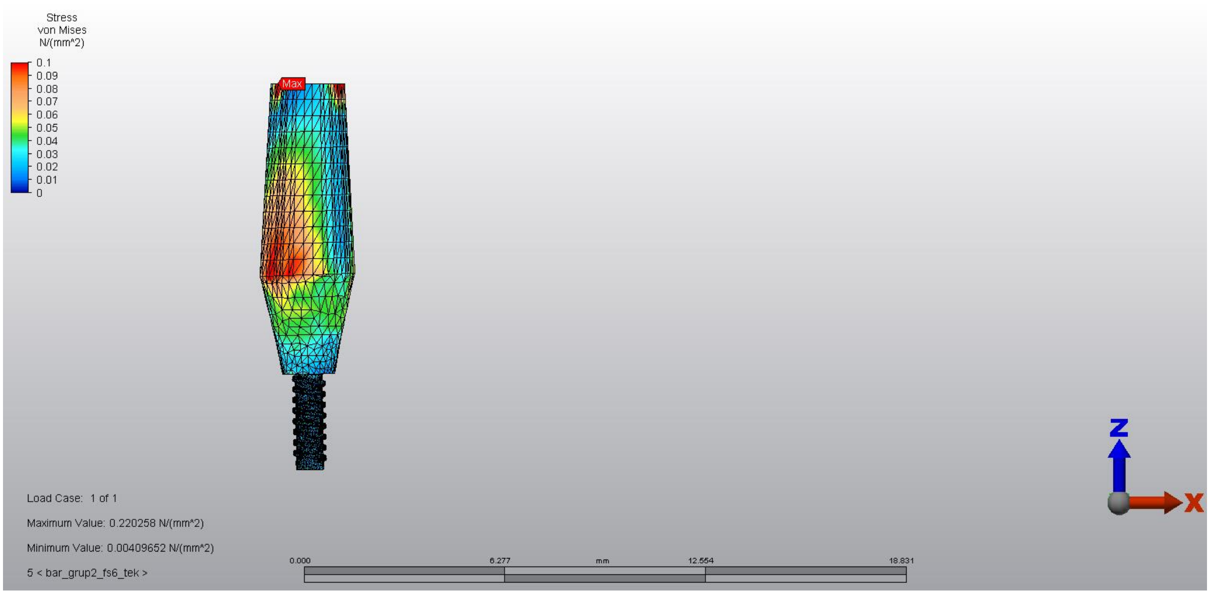
Stress
von Mises
N/(mm^2)
0.1
0.09
0.08
0.07
0.06
0.05
0.04
0.03
0.02
0.01
0
Max
Load Case: 1 of 1
Maximum Value: 0.220258 N/(mm^2)
Minimum Value: 0.00409652 N/(mm^2)
5 < bar_grup2_fs6_tek >
0.000
6.277
mm
12.554
18.831
Z
X

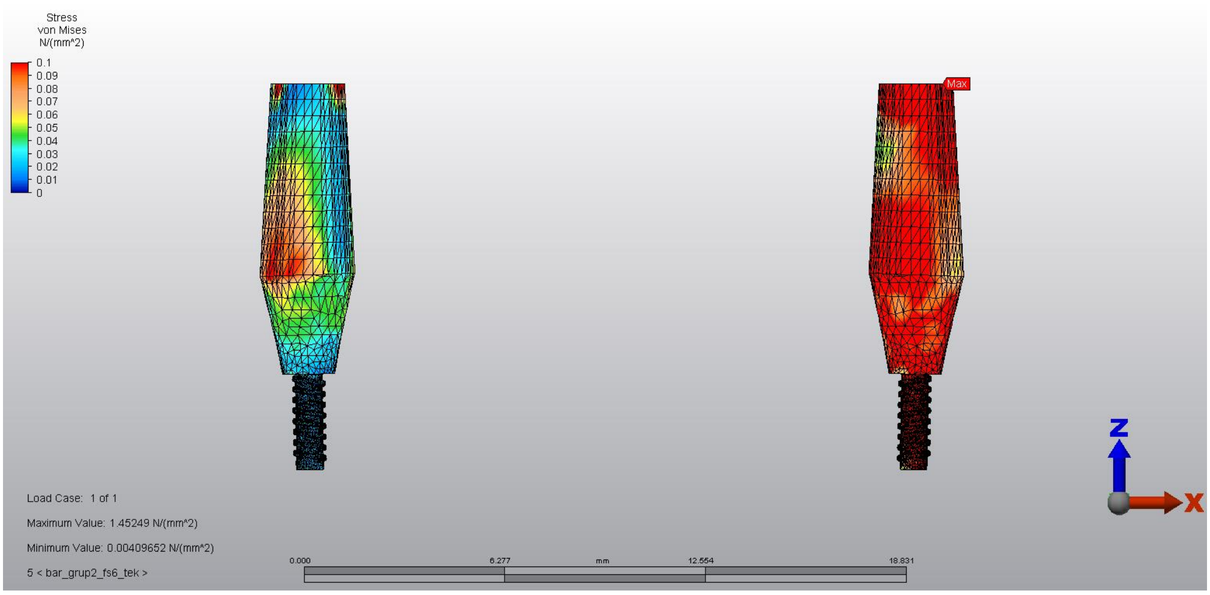
Stress
von Mises
N/(mm^2)
0.1
0.09
0.08
0.07
0.06
0.05
0.04
0.03
0.02
0.01
0
Max
Load Case: 1 of 1
Maximum Value: 1.45249 N/(mm^2)
Minimum Value: 0.00409652 N/(mm^2)
5 < bar_grup2_fs6_tek >
0.000
6.277
mm
12.554
18.831
Z
X

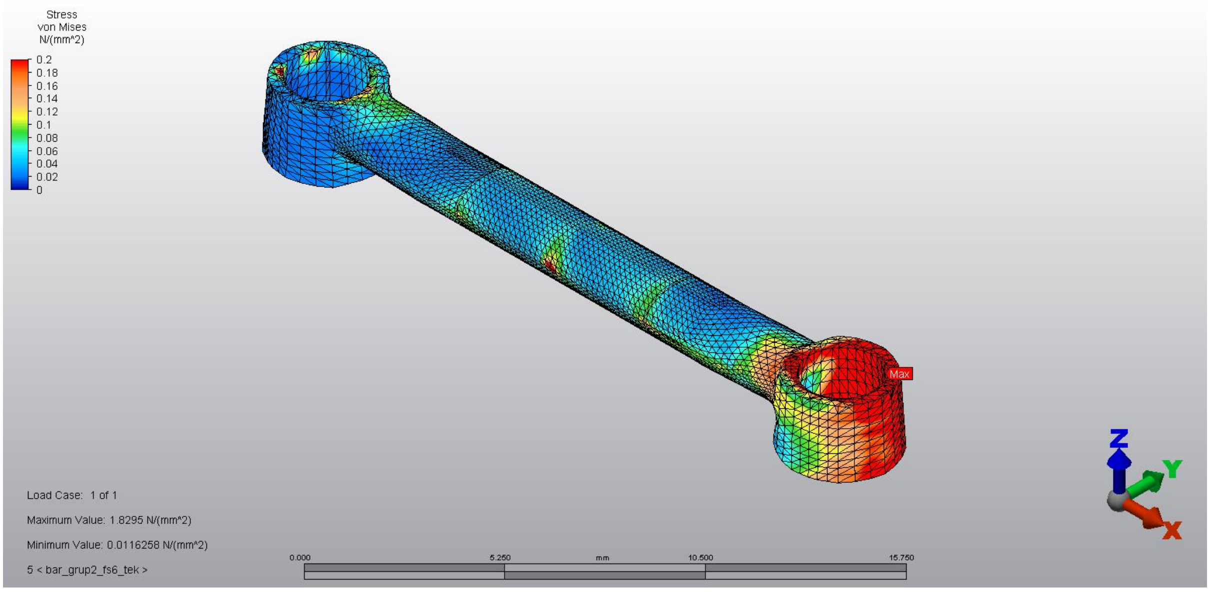
Stress
von Mises
N/(mm^2)
0.2
0.18
0.16
0.14
0.12
0.1
0.08
0.06
0.04
0.02
0
Max
Load Case: 1 of 1
Maximum Value: 1.8295 N/(mm^2)
Minimum Value: 0.0116258 N/(mm^2)
5 < bar_grup2_fs6_tek >
0.000
5.250
mm
10.500
15.750
Z
Y
X

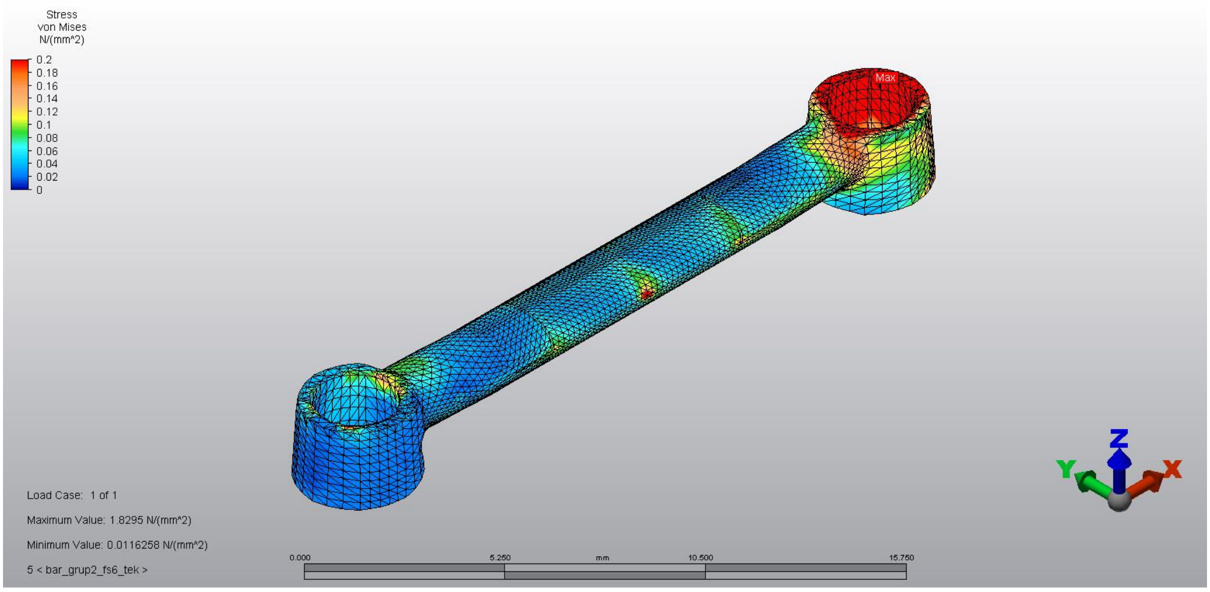
Stress
von Mises
N/(mm^2)
0.2
0.18
0.16
0.14
0.12
0.1
0.08
0.06
0.04
0.02
0
Max
Load Case: 1 of 1
Maximum Value: 1.8295 N/(mm^2)
Minimum Value: 0.0116258 N/(mm^2)
5 < bar_grup2_fs6_tek >
0.000
5.250
mm
10.500
15.750
Z
Y
X

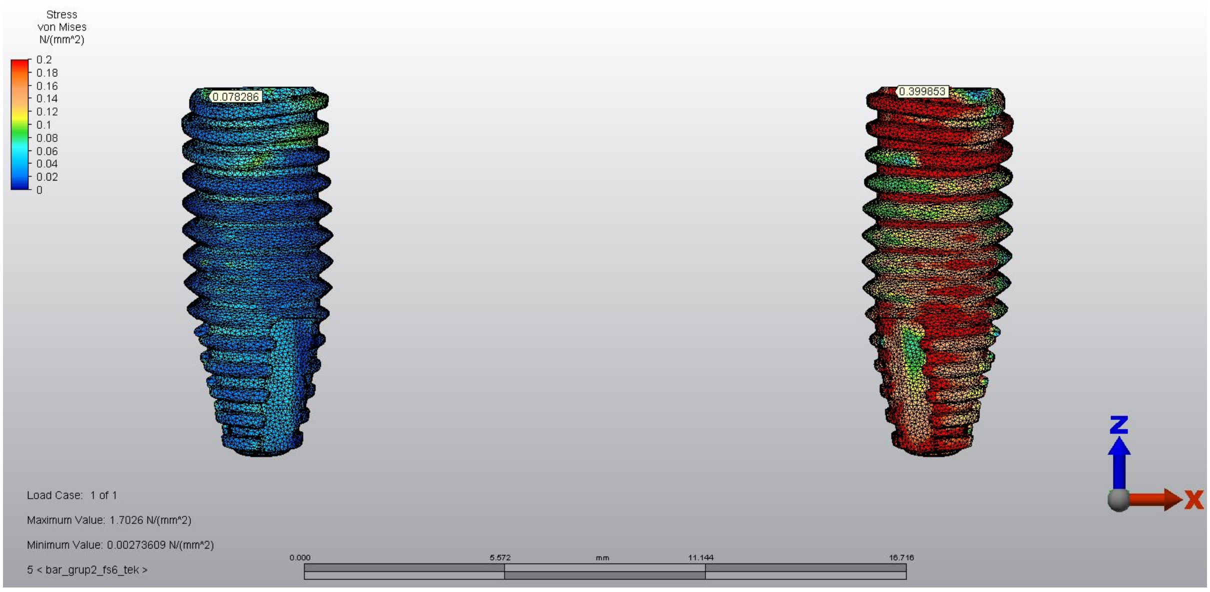
Stress
von Mises
N/(mm^2)
0.2
0.18
0.16
0.14
0.12
0.1
0.08
0.06
0.04
0.02
0
0.078286
0.399853
Load Case: 1 of 1
Maximum Value: 1.7026 N/(mm^2)
Minimum Value: 0.00273609 N/(mm^2)
5 < bar_grup2_fs6_tek >
0.000
5.572
mm
11.144
16.716
Z
X

fs6_cift

Number of nodes = 157716

Number of elements = 783629

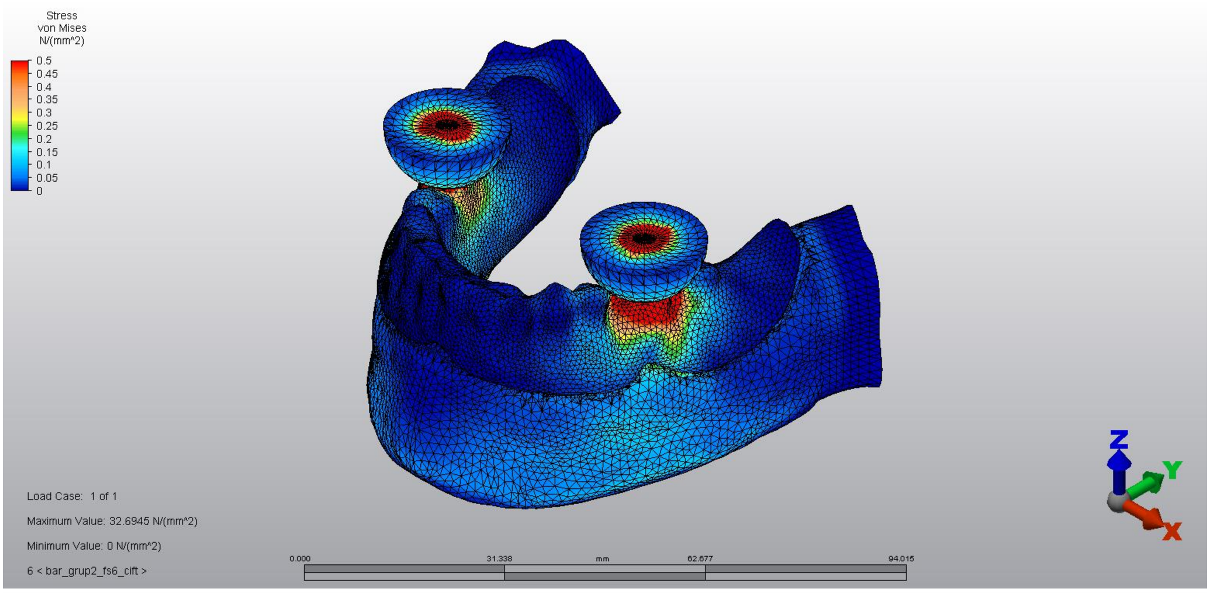

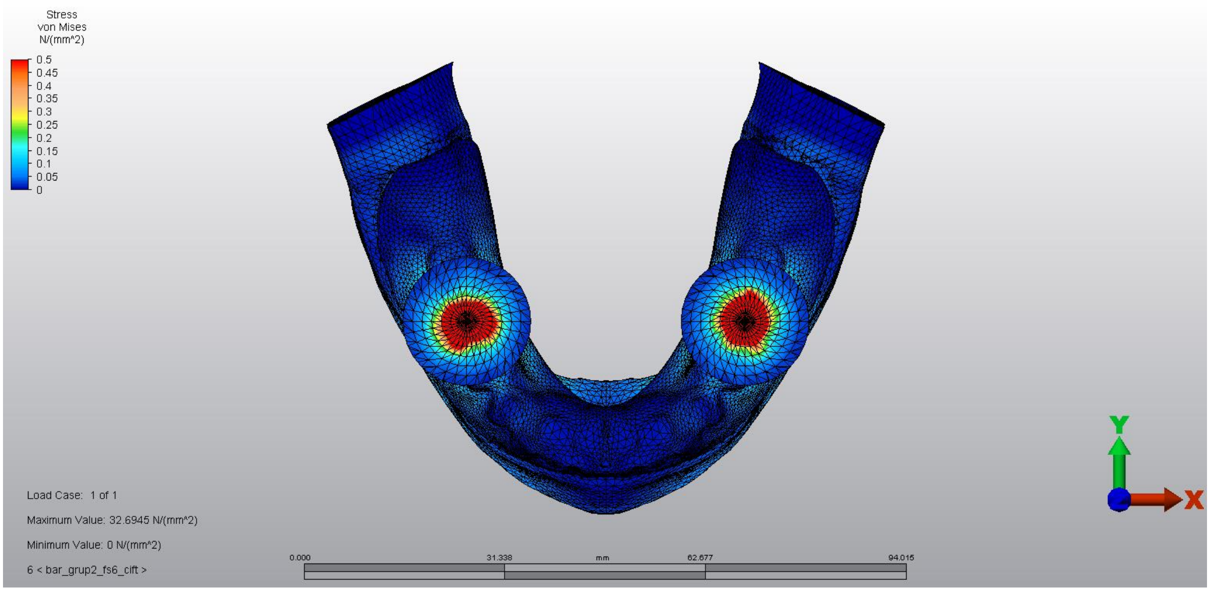

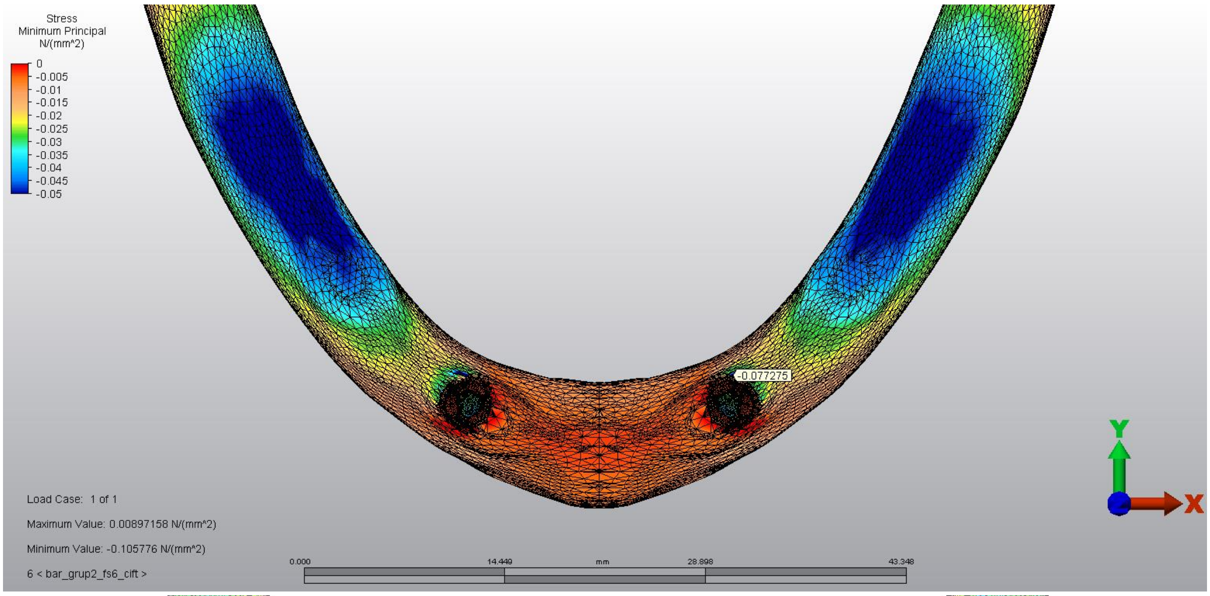
Stress
Minimum Principal
N/(mm^2)
0
-0.005
-0.01
-0.015
-0.02
-0.025
-0.03
-0.035
-0.04
-0.045
-0.05
-0.077275
Load Case: 1 of 1
Maximum Value: 0.00897158 N/(mm^2)
Minimum Value: -0.105776 N/(mm^2)
6 < bar_grup2_fs6_cift >
0.000
14.449
mm
28.898
43.348
Y
X

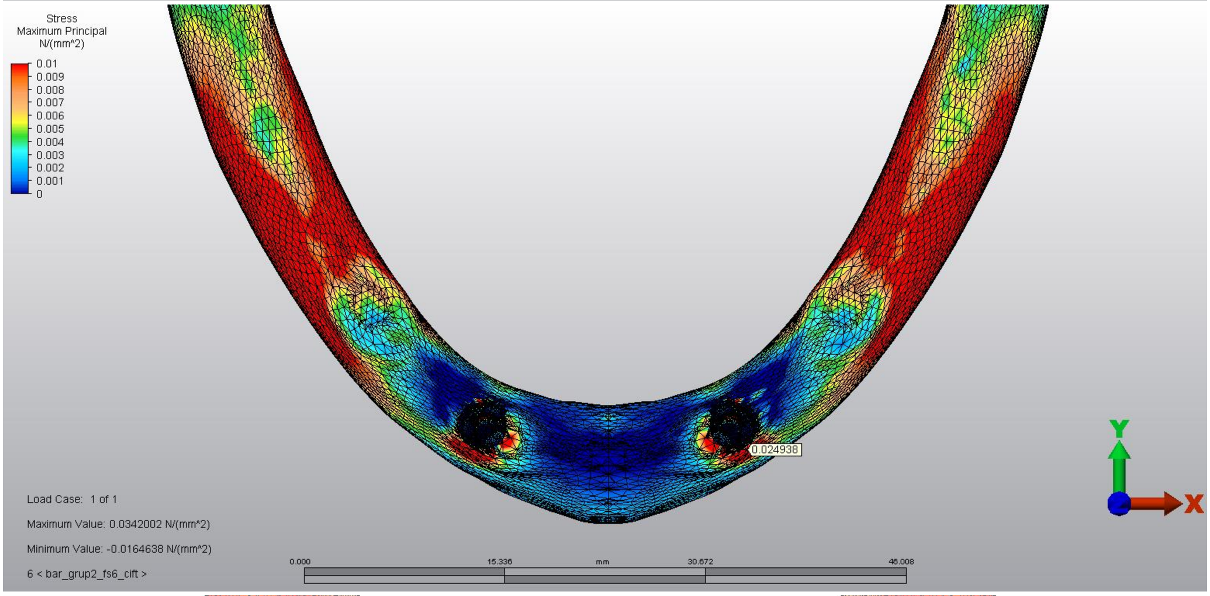
Stress
Maximum Principal
N/(mm^2)
0.01
0.009
0.008
0.007
0.006
0.005
0.004
0.003
0.002
0.001
0
0.024938
Load Case: 1 of 1
Maximum Value: 0.0342002 N/(mm^2)
Minimum Value: -0.0164638 N/(mm^2)
6 < bar_grup2_fs6_cift >
0.000
15.336
mm
30.672
46.008
Y
X

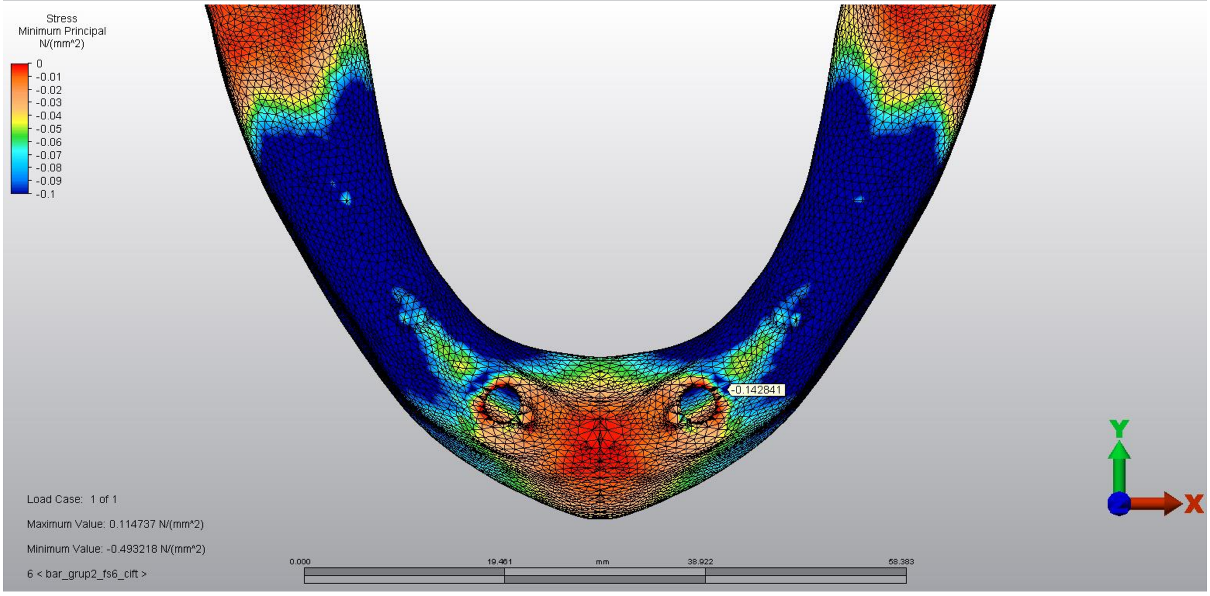
Stress
Minimum Principal
N/(mm^2)
0
-0.01
-0.02
-0.03
-0.04
-0.05
-0.06
-0.07
-0.08
-0.09
-0.1
-0.142841
Load Case: 1 of 1
Maximum Value: 0.114737 N/(mm^2)
Minimum Value: -0.493218 N/(mm^2)
6 < bar_grup2_fs6_cift >
0.000
19.461
mm
38.922
58.383
Y
X

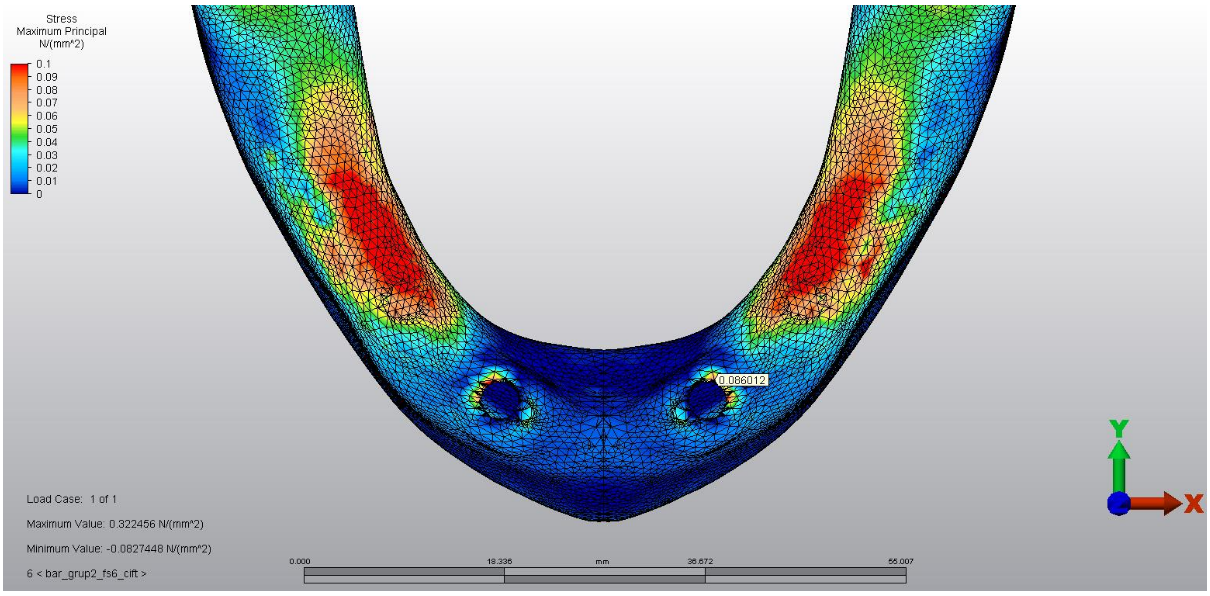
Stress
Maximum Principal
N/(mm^2)
0.1
0.09
0.08
0.07
0.06
0.05
0.04
0.03
0.02
0.01
0
0.086012
Load Case: 1 of 1
Maximum Value: 0.322456 N/(mm^2)
Minimum Value: -0.0827448 N/(mm^2)
6 < bar_grup2_fs6_cift >
0.000
18.336
mm
36.672
55.007
Y
X

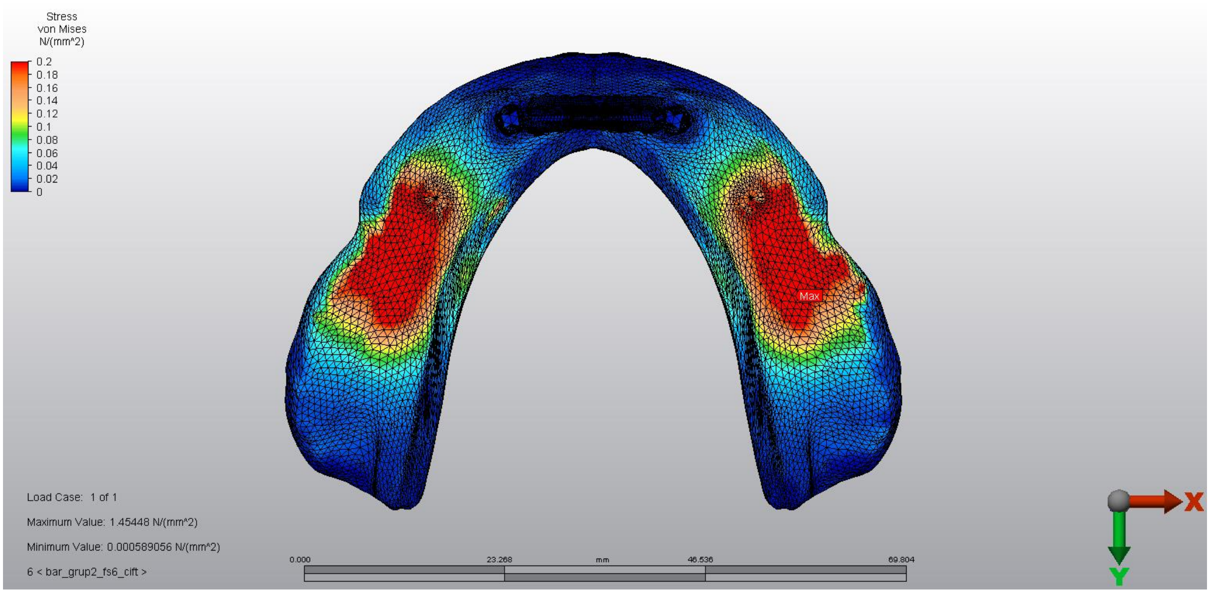
Stress
von Mises
N/(mm^2)
0.2
0.18
0.16
0.14
0.12
0.1
0.08
0.06
0.04
0.02
0
Max
Load Case: 1 of 1
Maximum Value: 1.45448 N/(mm^2)
Minimum Value: 0.000589056 N/(mm^2)
6 < bar_grup2_fs6_cift >
0.000
23.268
mm
46.536
69.804
X
Y

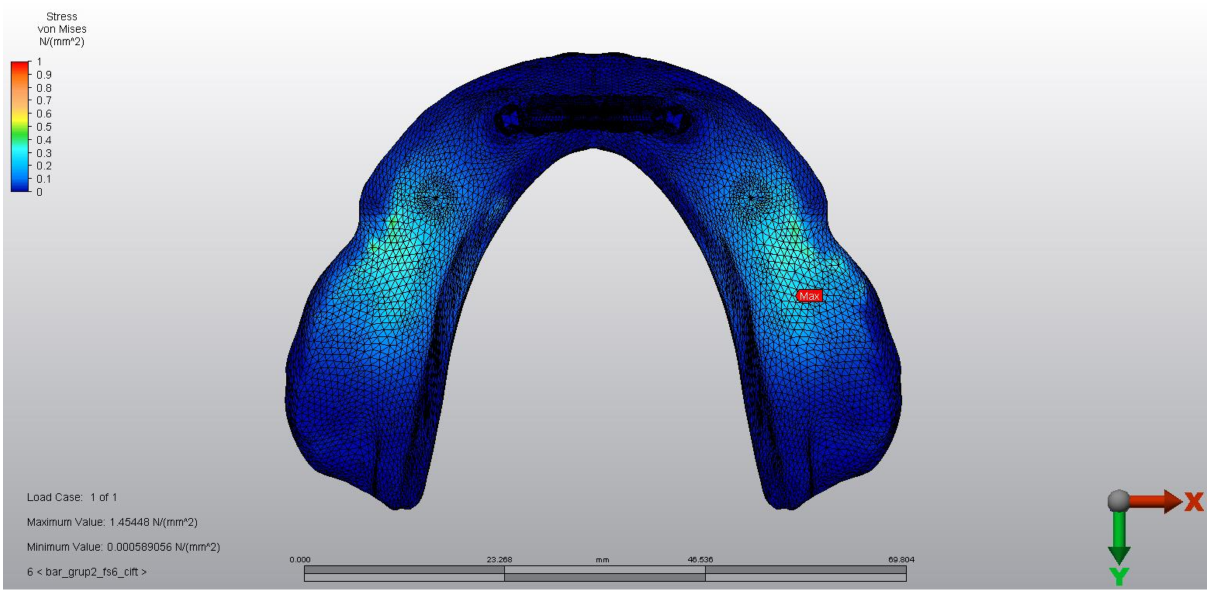
Stress
von Mises
N/(mm^2)
1
0.9
0.8
0.7
0.6
0.5
0.4
0.3
0.2
0.1
0
Max
Load Case: 1 of 1
Maximum Value: 1.45448 N/(mm^2)
Minimum Value: 0.000589056 N/(mm^2)
6 < bar_grup2_fs6_cift >
0.000
23.268
mm
46.536
69.804
X
Y

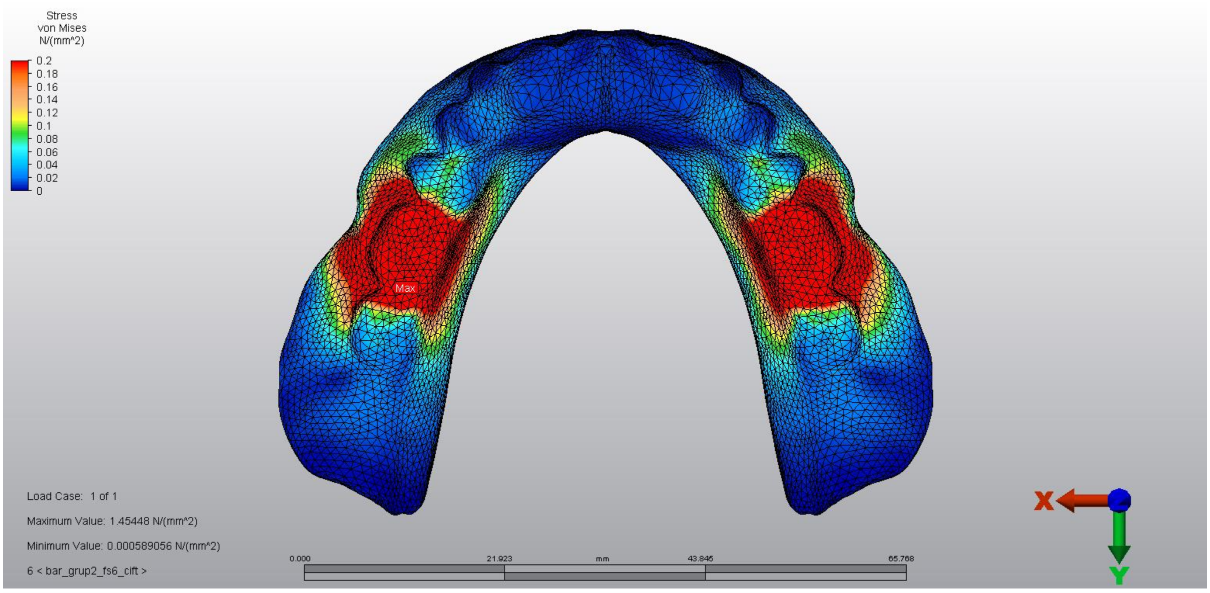
Stress
von Mises
N/(mm^2)
0.2
0.18
0.16
0.14
0.12
0.1
0.08
0.06
0.04
0.02
0
Max
Load Case: 1 of 1
Maximum Value: 1.45448 N/(mm^2)
Minimum Value: 0.000589056 N/(mm^2)
6 < bar_grup2_fs6_cift >
0.000
21.923
mm
43.845
65.768
X
Y

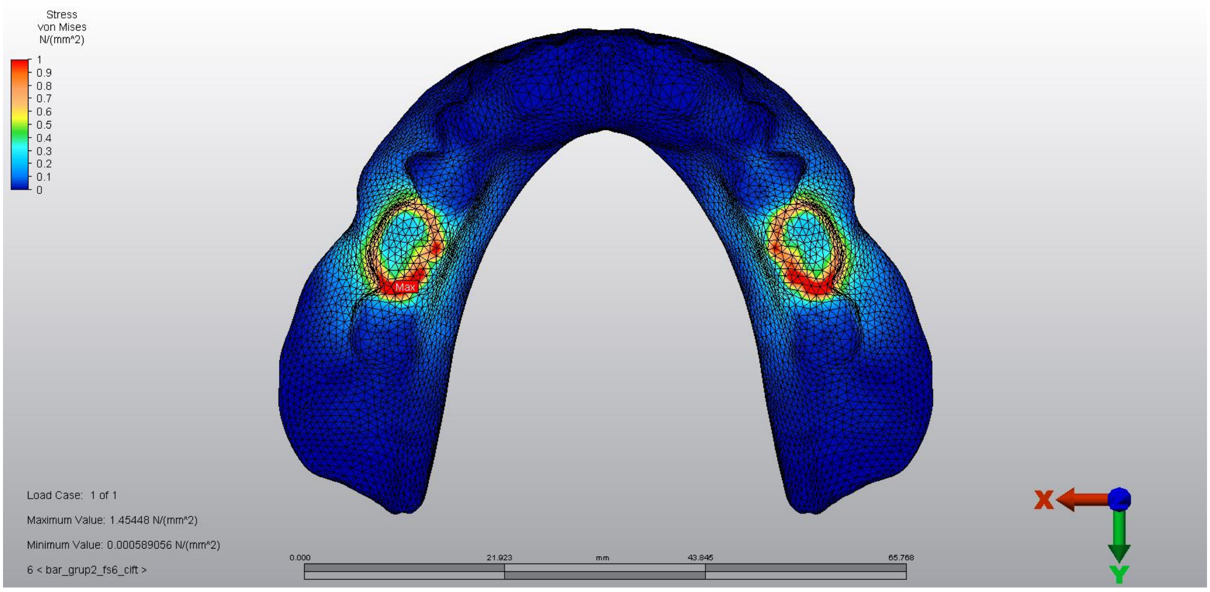
Stress
von Mises
N/(mm^2)
1
0.9
0.8
0.7
0.6
0.5
0.4
0.3
0.2
0.1
0
Max
Load Case: 1 of 1
Maximum Value: 1.45448 N/(mm^2)
Minimum Value: 0.000589056 N/(mm^2)
6 < bar_grup2_fs6_cift >
0.000
21.923
mm
43.845
65.768
X
Y

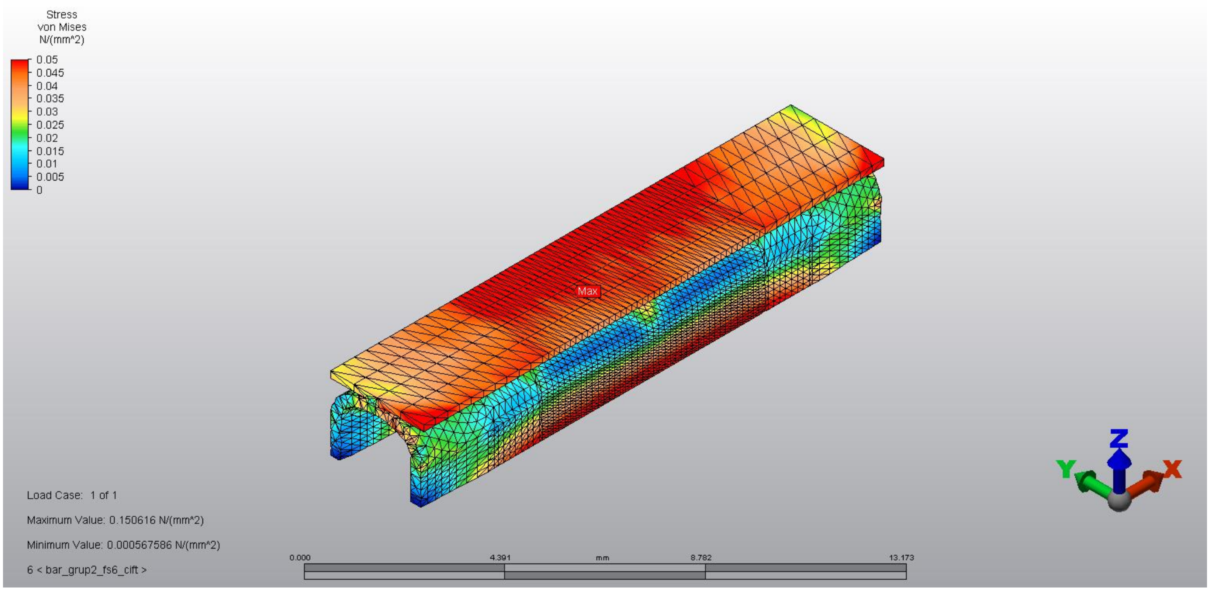
Stress
von Mises
N/(mm^2)
0.05
0.045
0.04
0.035
0.03
0.025
0.02
0.015
0.01
0.005
0
Max
Load Case: 1 of 1
Maximum Value: 0.150616 N/(mm^2)
Minimum Value: 0.000567586 N/(mm^2)
6 < bar_grup2_fs6_cift >
0.000
4.391
mm
8.782
13.173
Z
Y
X

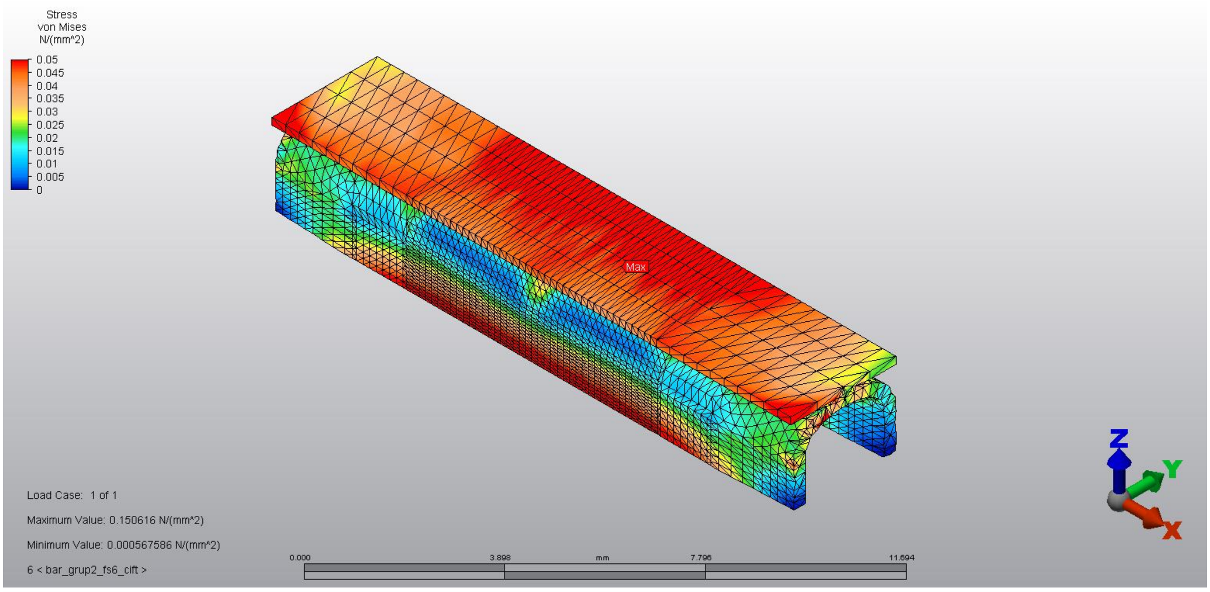
Stress
von Mises
N/(mm^2)
0.05
0.045
0.04
0.035
0.03
0.025
0.02
0.015
0.01
0.005
0
Max
Load Case: 1 of 1
Maximum Value: 0.150616 N/(mm^2)
Minimum Value: 0.000567586 N/(mm^2)
6 < bar_grup2_fs6_cift >
0.000
3.898
mm
7.796
11.694
Z
Y
X

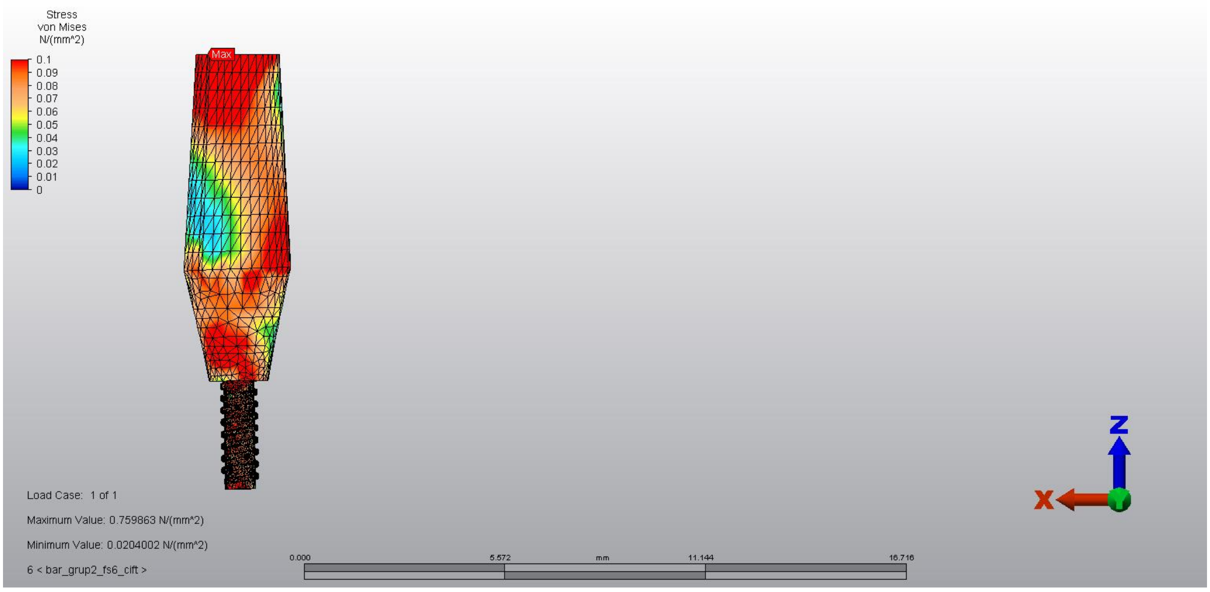
Stress
von Mises
N/(mm^2)
0.1
0.09
0.08
0.07
0.06
0.05
0.04
0.03
0.02
0.01
0
Max
Load Case: 1 of 1
Maximum Value: 0.759863 N/(mm^2)
Minimum Value: 0.0204002 N/(mm^2)
6 < bar_grup2_fs6_cift >
0.000
5.572
mm
11.144
16.716
Z
X

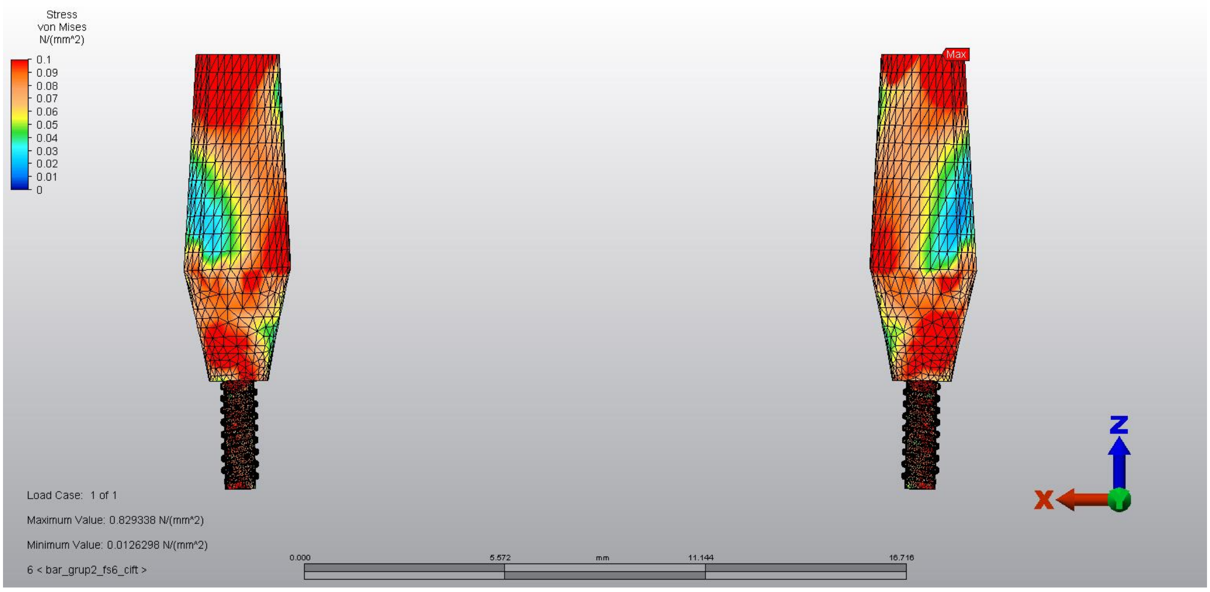
Stress
von Mises
N/(mm^2)
0.1
0.09
0.08
0.07
0.06
0.05
0.04
0.03
0.02
0.01
0
Max
Load Case: 1 of 1
Maximum Value: 0.829338 N/(mm^2)
Minimum Value: 0.0126298 N/(mm^2)
6 < bar_grup2_fs6_cift >
0.000
5.572
mm
11.144
16.716
Z
X

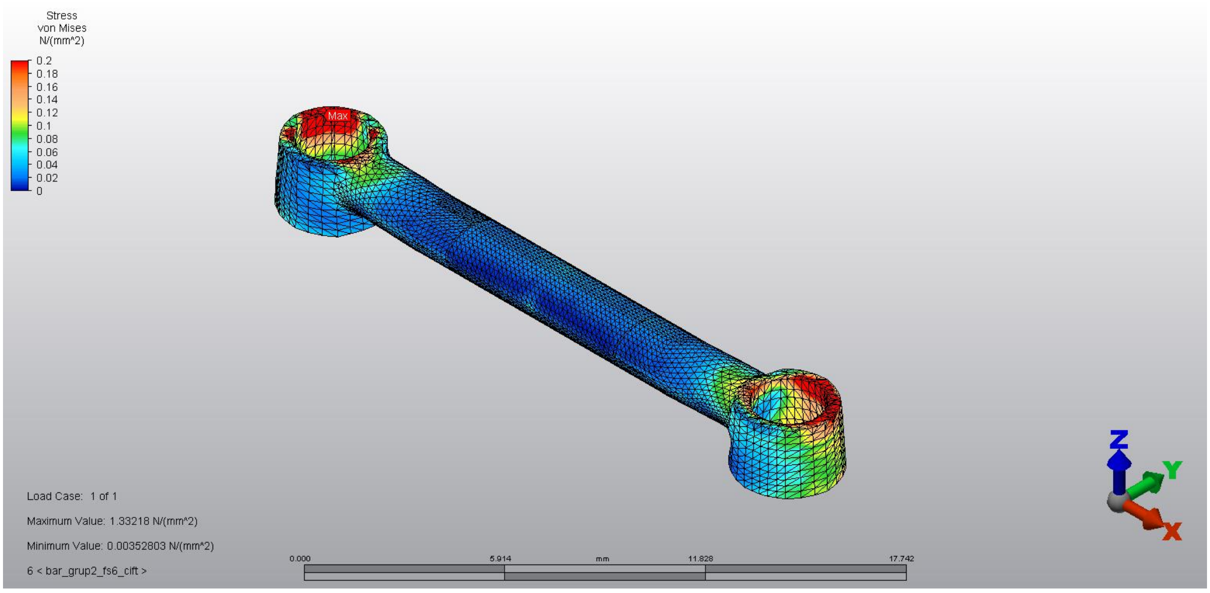
Stress
von Mises
N/(mm^2)
0.2
0.18
0.16
0.14
0.12
0.1
0.08
0.06
0.04
0.02
0
Max
Load Case: 1 of 1
Maximum Value: 1.33218 N/(mm^2)
Minimum Value: 0.00352803 N/(mm^2)
6 < bar_grup2_fs6_cift >
0.000
5.914
mm
11.828
17.742
Z
Y
X

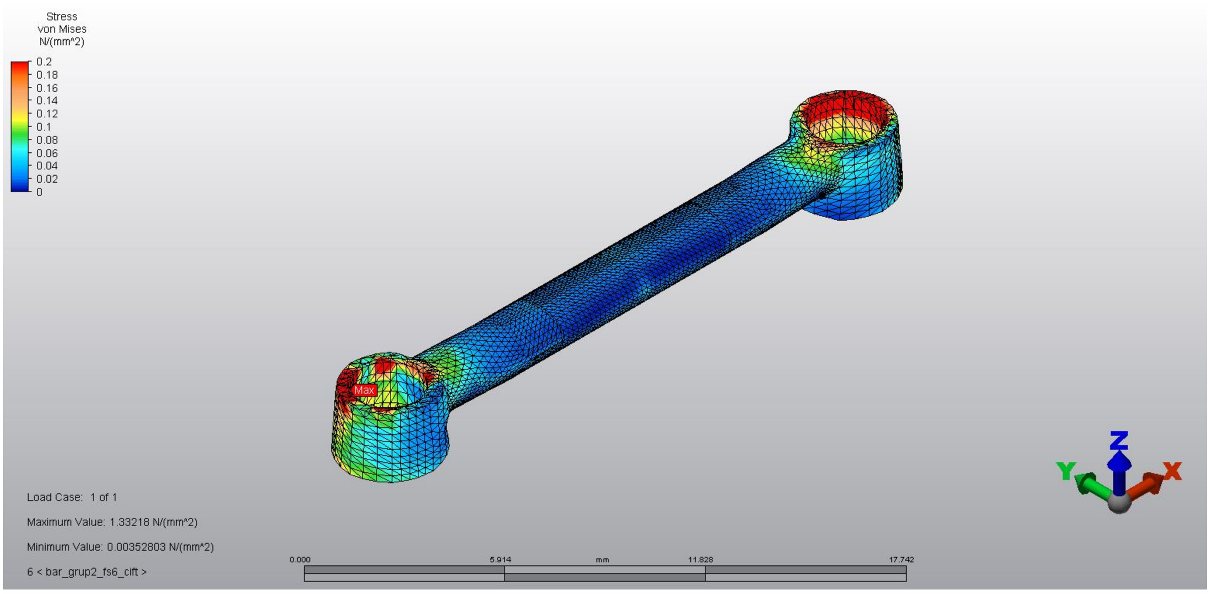
Stress
von Mises
N/(mm^2)
0.2
0.18
0.16
0.14
0.12
0.1
0.08
0.06
0.04
0.02
0
Max
Load Case: 1 of 1
Maximum Value: 1.33218 N/(mm^2)
Minimum Value: 0.00352803 N/(mm^2)
6 < bar_grup2_fs6_cift >
0.000
5.914
mm
11.828
17.742
Z
Y
X

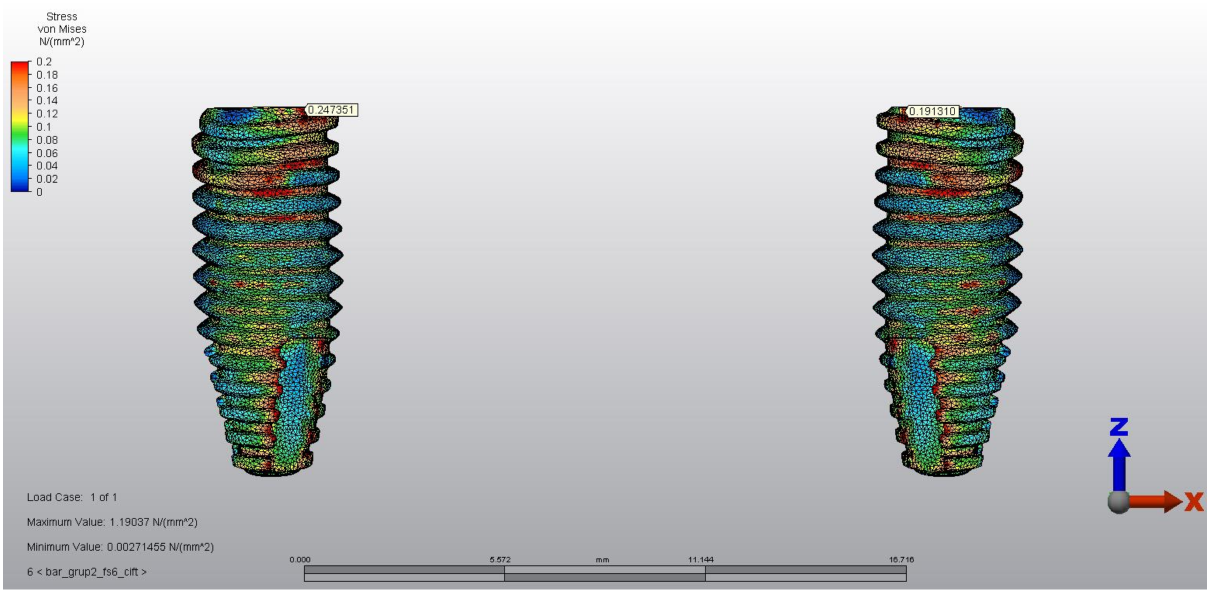
Stress
von Mises
N/(mm^2)
0.2
0.18
0.16
0.14
0.12
0.1
0.08
0.06
0.04
0.02
0
0.247351
0.191310
Load Case: 1 of 1
Maximum Value: 1.19037 N/(mm^2)
Minimum Value: 0.00271455 N/(mm^2)
6 < bar_grup2_fs6_cift >
0.000
5.572
mm
11.144
16.716
Z
X

GRUP 03

INCISAL

Number of nodes = 160972

Number of elements = 792744

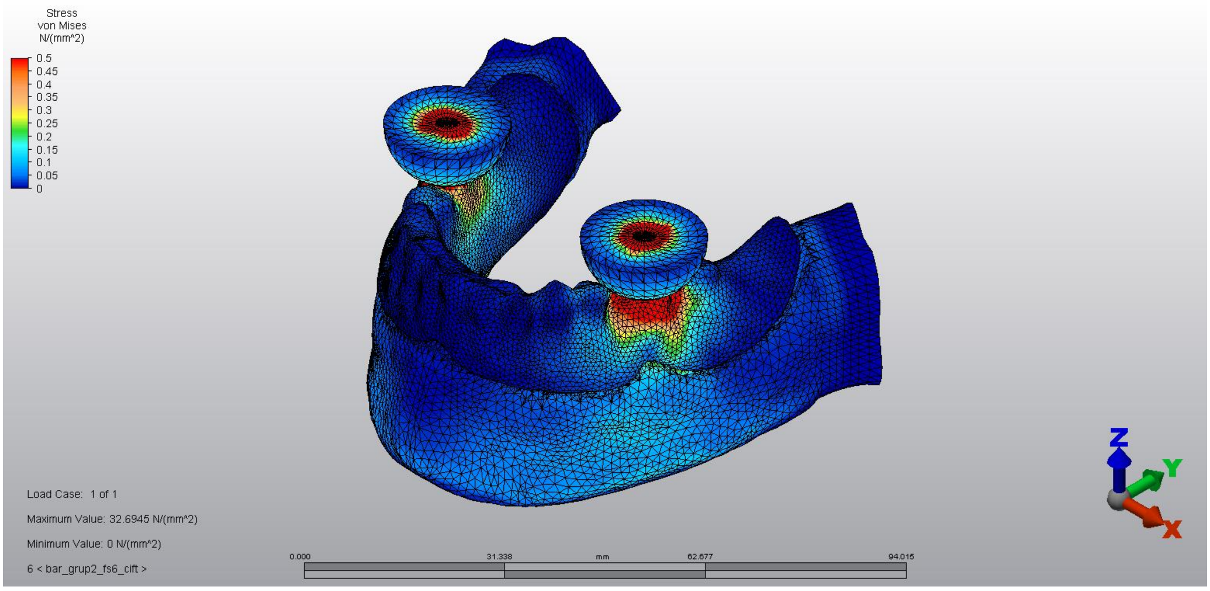

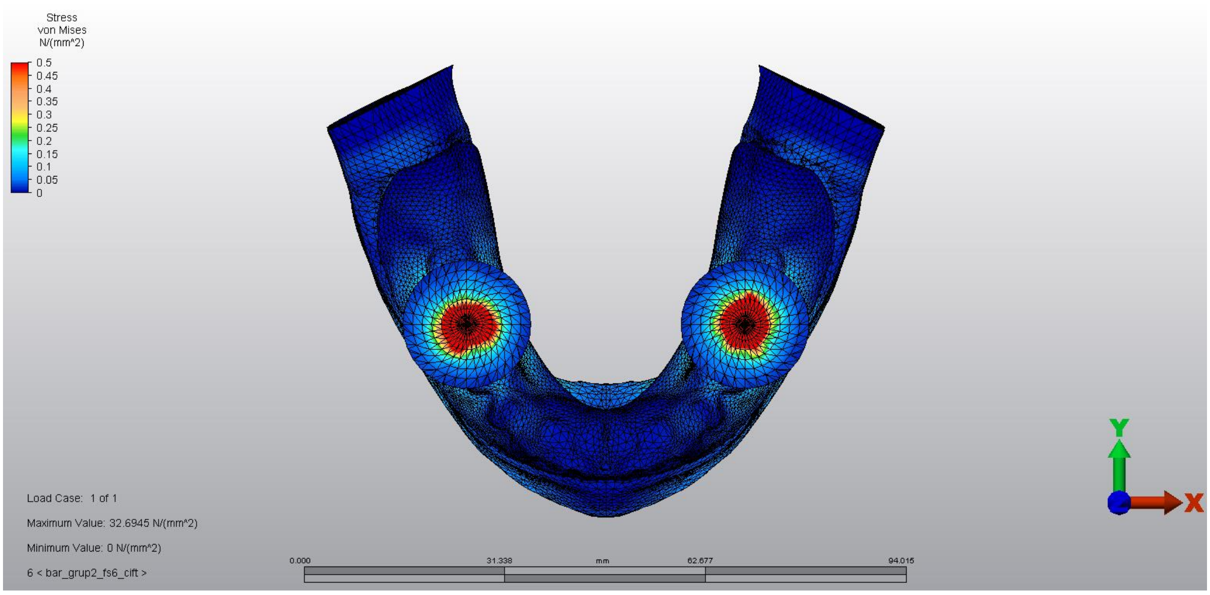

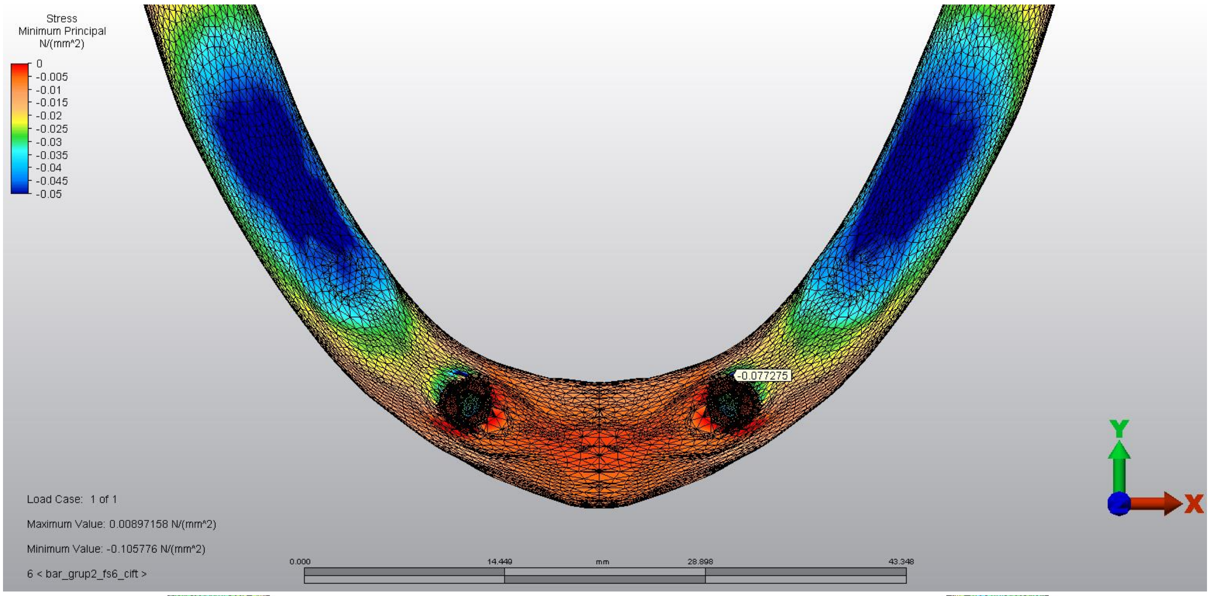
Stress
Minimum Principal
N/(mm^2)
0
-0.005
-0.01
-0.015
-0.02
-0.025
-0.03
-0.035
-0.04
-0.045
-0.05
-0.077275
Load Case: 1 of 1
Maximum Value: 0.00897158 N/(mm^2)
Minimum Value: -0.105776 N/(mm^2)
6 < bar_grup2_fs6_cift >
0.000
14.449
mm
28.898
43.348
Y
X

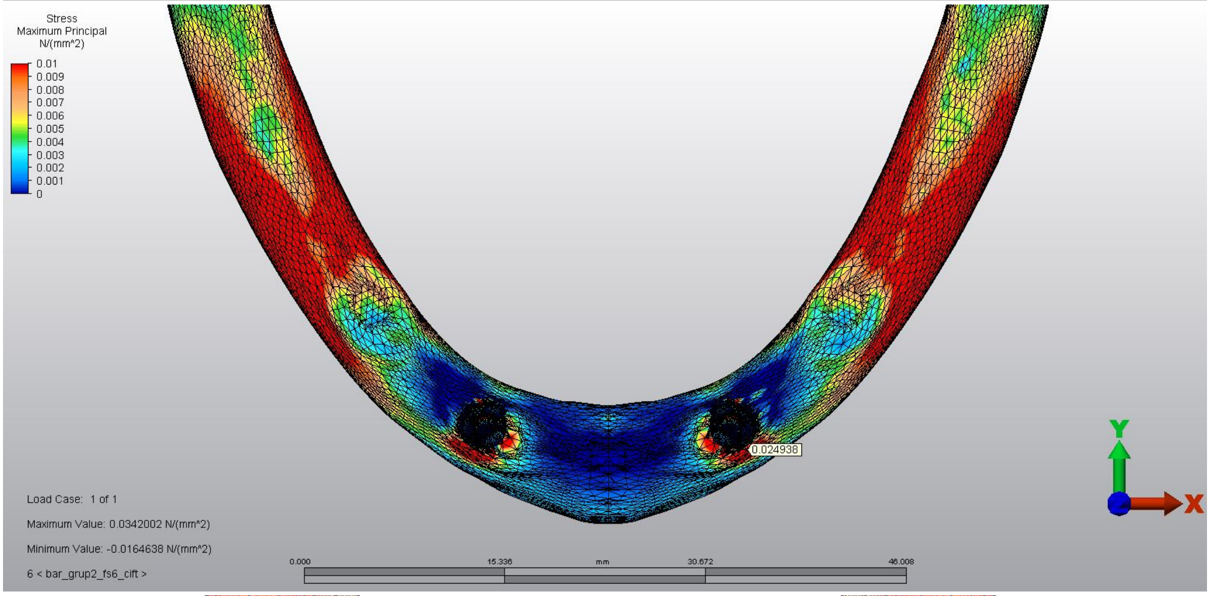
Stress
Maximum Principal
N/(mm^2)
0.01
0.009
0.008
0.007
0.006
0.005
0.004
0.003
0.002
0.001
0
0.024938
Load Case: 1 of 1
Maximum Value: 0.0342002 N/(mm^2)
Minimum Value: -0.0164638 N/(mm^2)
6 < bar_grup2_fs6_cift >
0.000
15.336
mm
30.672
46.008
Y
X

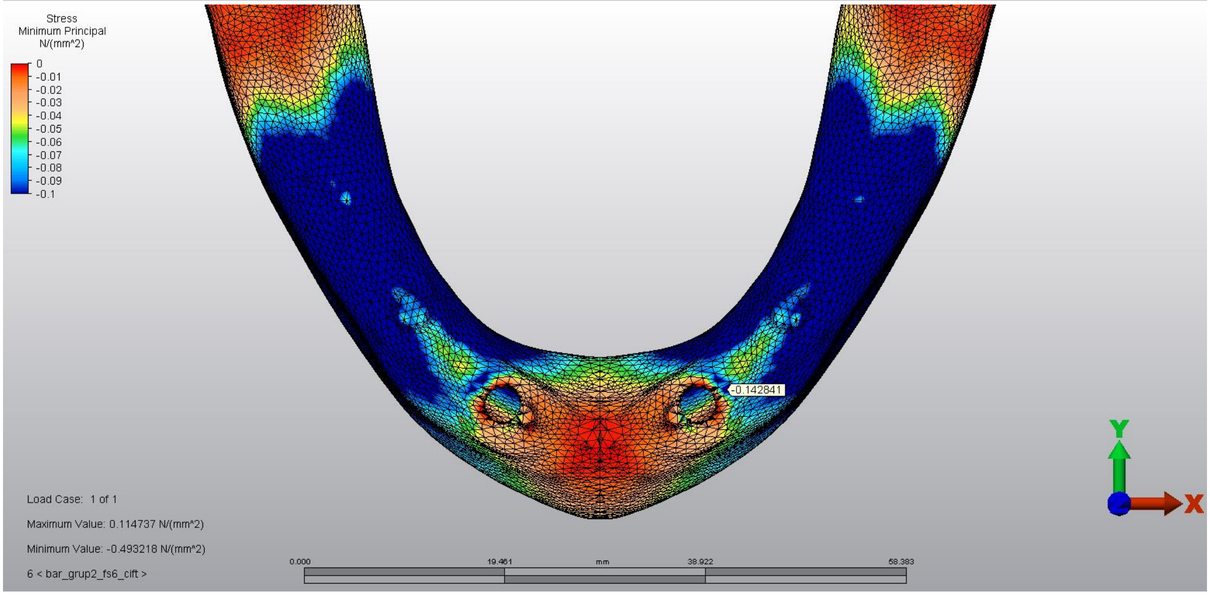
Stress
Minimum Principal
N/(mm^2)
0
-0.01
-0.02
-0.03
-0.04
-0.05
-0.06
-0.07
-0.08
-0.09
-0.1
-0.142841
Load Case: 1 of 1
Maximum Value: 0.114737 N/(mm^2)
Minimum Value: -0.493218 N/(mm^2)
6 < bar_grup2_fs6_cift >
0.000
19.461
mm
38.922
58.383
Y
X

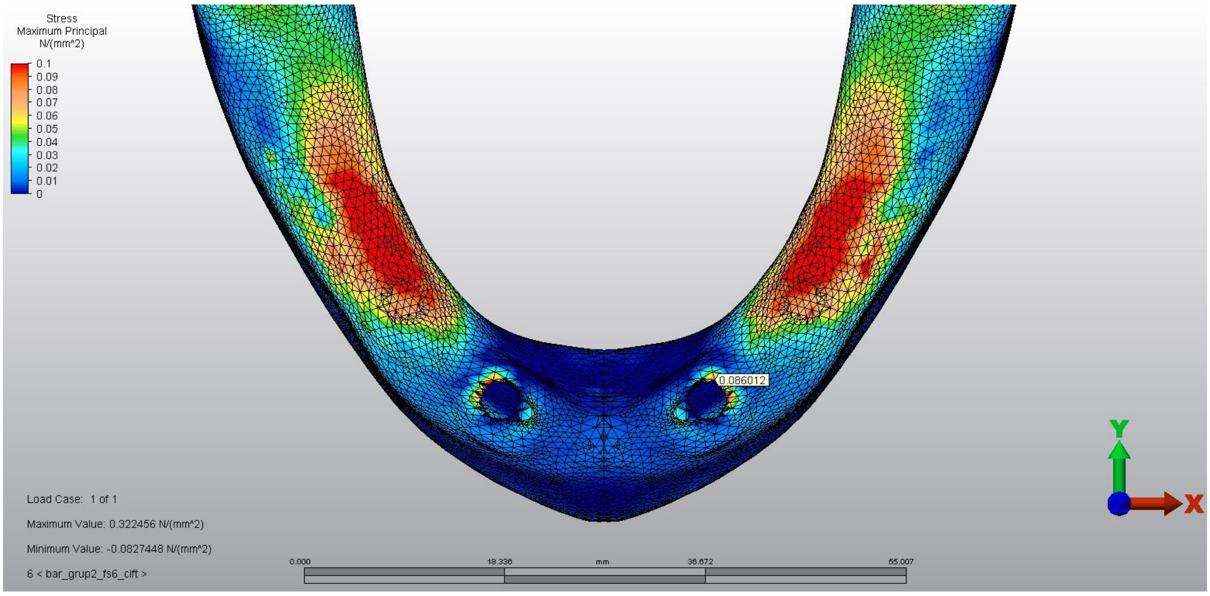
Stress
Maximum Principal
N/(mm^2)
0.1
0.09
0.08
0.07
0.06
0.05
0.04
0.03
0.02
0.01
0
0.086012
Load Case: 1 of 1
Maximum Value: 0.322456 N/(mm^2)
Minimum Value: -0.0827448 N/(mm^2)
6 < bar_grup2_fs6_cift >
0.000
18.336
mm
36.672
55.007
Y
X

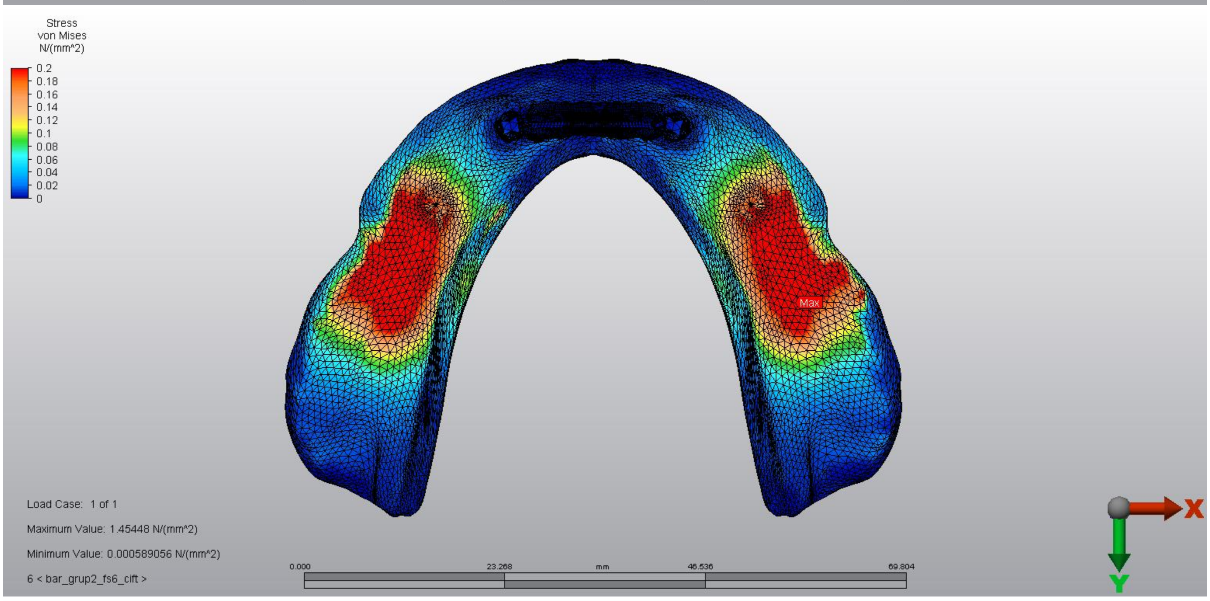
Stress
von Mises
N/(mm^2)
0.2
0.18
0.16
0.14
0.12
0.1
0.08
0.06
0.04
0.02
0
Max
Load Case: 1 of 1
Maximum Value: 1.45448 N/(mm^2)
Minimum Value: 0.000589056 N/(mm^2)
6 < bar_grup2_fs6_cift >
0.000
23.268
mm
46.536
69.804
X
Y

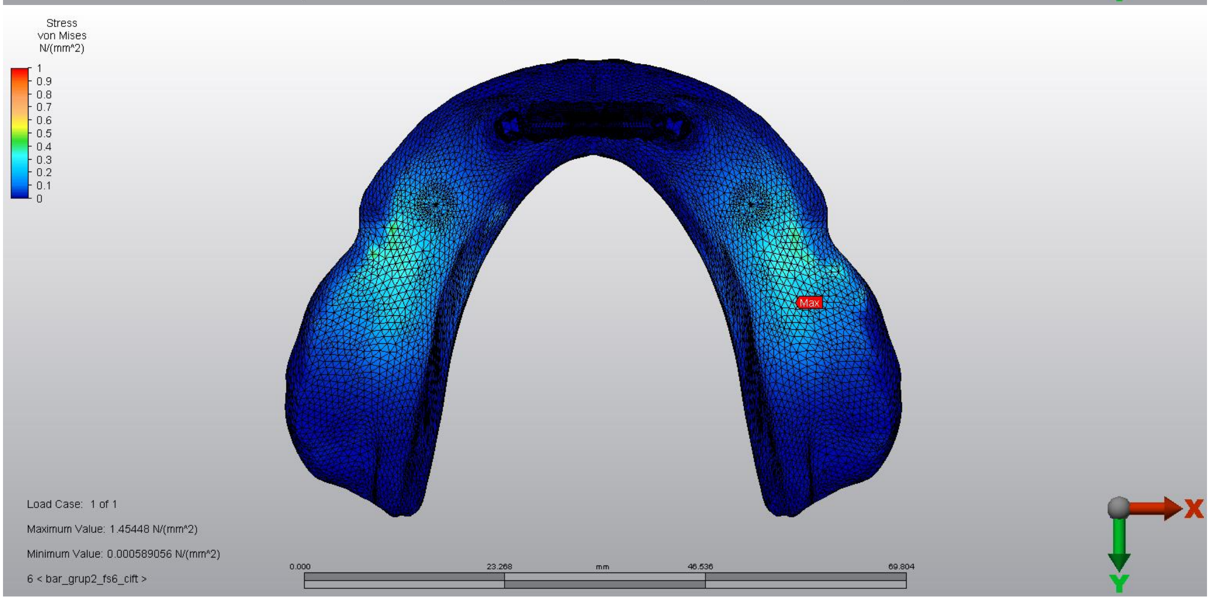
Stress
von Mises
N/(mm^2)
1
0.9
0.8
0.7
0.6
0.5
0.4
0.3
0.2
0.1
0
Max
Load Case: 1 of 1
Maximum Value: 1.45448 N/(mm^2)
Minimum Value: 0.000589056 N/(mm^2)
6 < bar_grup2_fs6_cift >
0.000
23.268
mm
46.536
69.804
X
Y

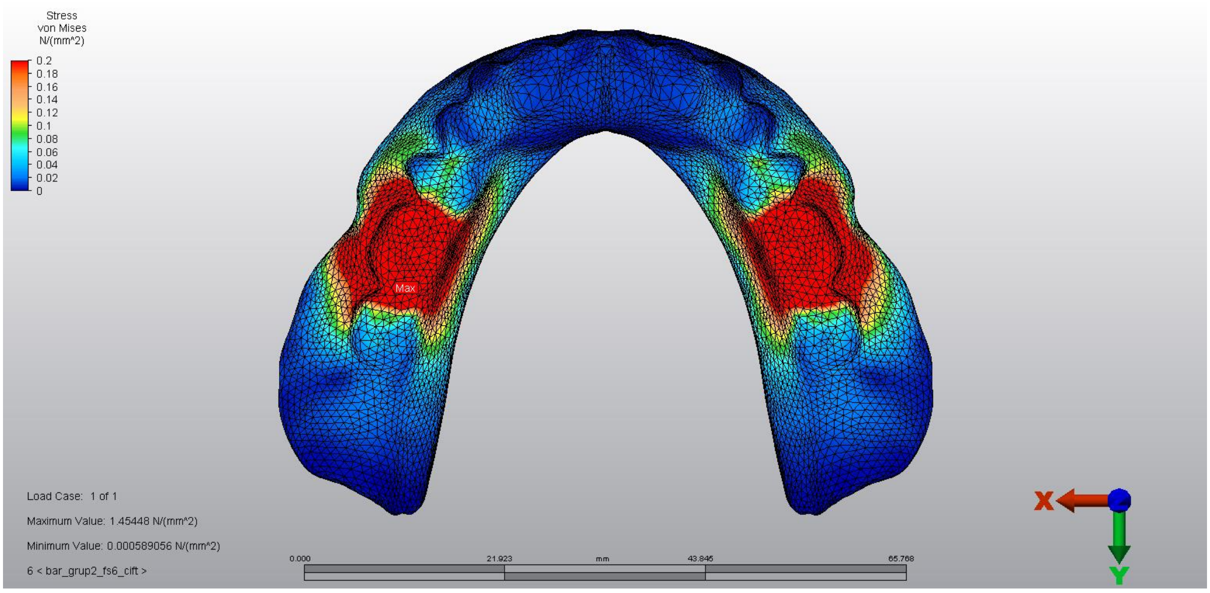
Stress
von Mises
N/(mm^2)
0.2
0.18
0.16
0.14
0.12
0.1
0.08
0.06
0.04
0.02
0
Max
Load Case: 1 of 1
Maximum Value: 1.45448 N/(mm^2)
Minimum Value: 0.000589056 N/(mm^2)
6 < bar_grup2_fs6_cift >
0.000
21.923
mm
43.845
65.768
X
Y

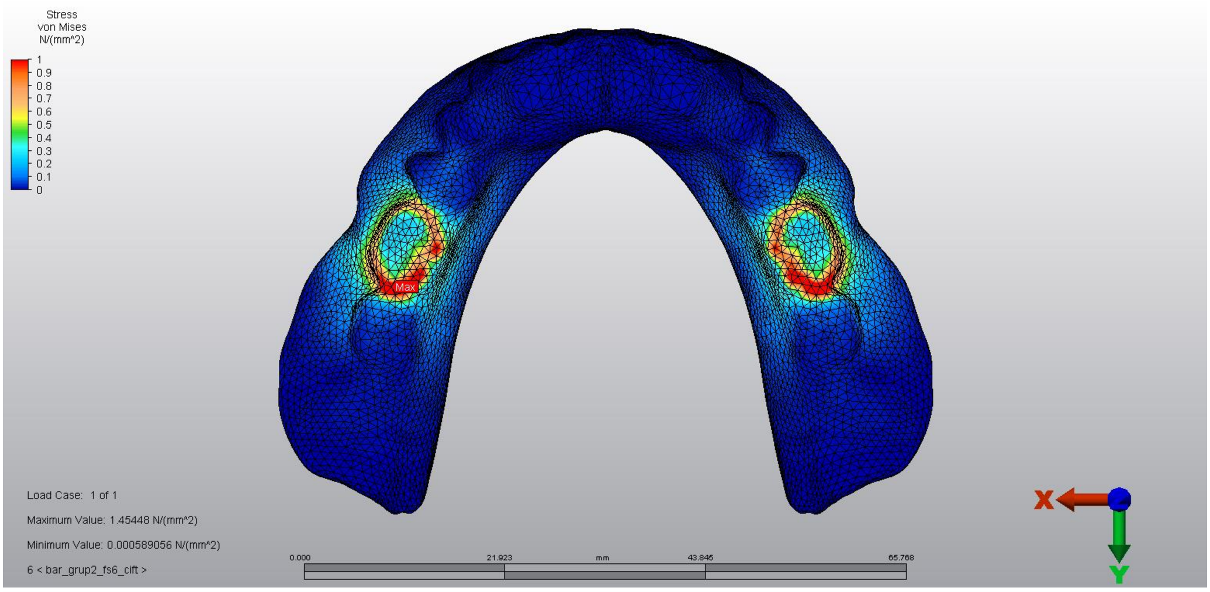
Stress
von Mises
N/(mm^2)
1
0.9
0.8
0.7
0.6
0.5
0.4
0.3
0.2
0.1
0
Max
Load Case: 1 of 1
Maximum Value: 1.45448 N/(mm^2)
Minimum Value: 0.000589056 N/(mm^2)
6 < bar_grup2_fs6_cift >
0.000
21.923
mm
43.845
65.768
X
Y

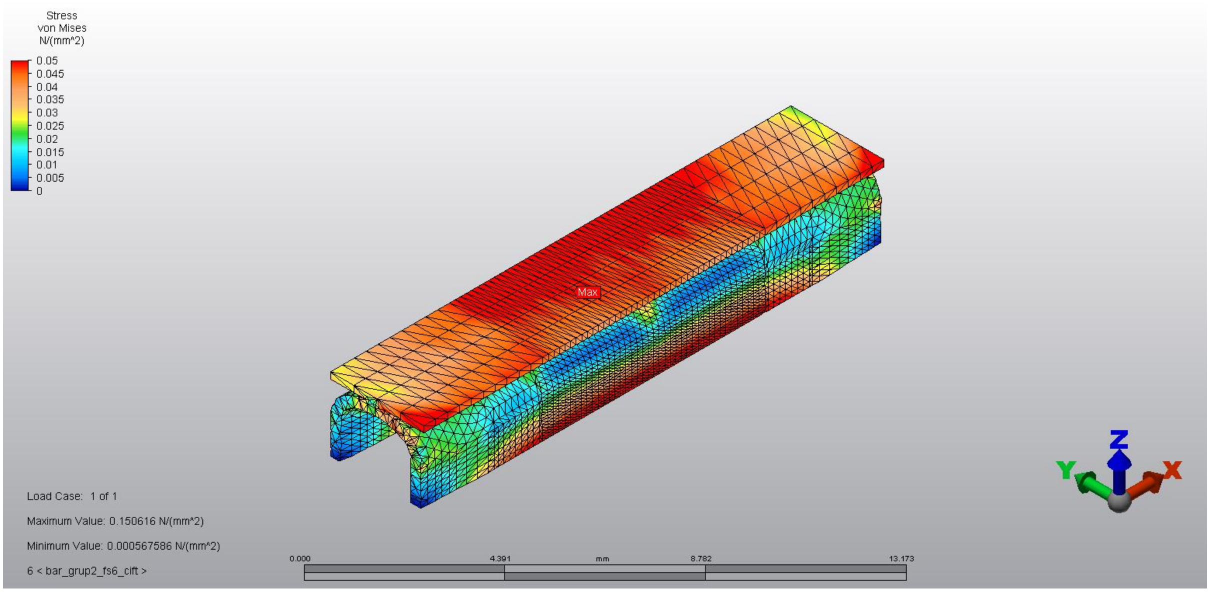
Stress
von Mises
N/(mm^2)
0.05
0.045
0.04
0.035
0.03
0.025
0.02
0.015
0.01
0.005
0
Max
Load Case: 1 of 1
Maximum Value: 0.150616 N/(mm^2)
Minimum Value: 0.000567586 N/(mm^2)
6 < bar_grup2_fs6_cift >
0.000
4.391
mm
8.782
13.173
Z
Y
X

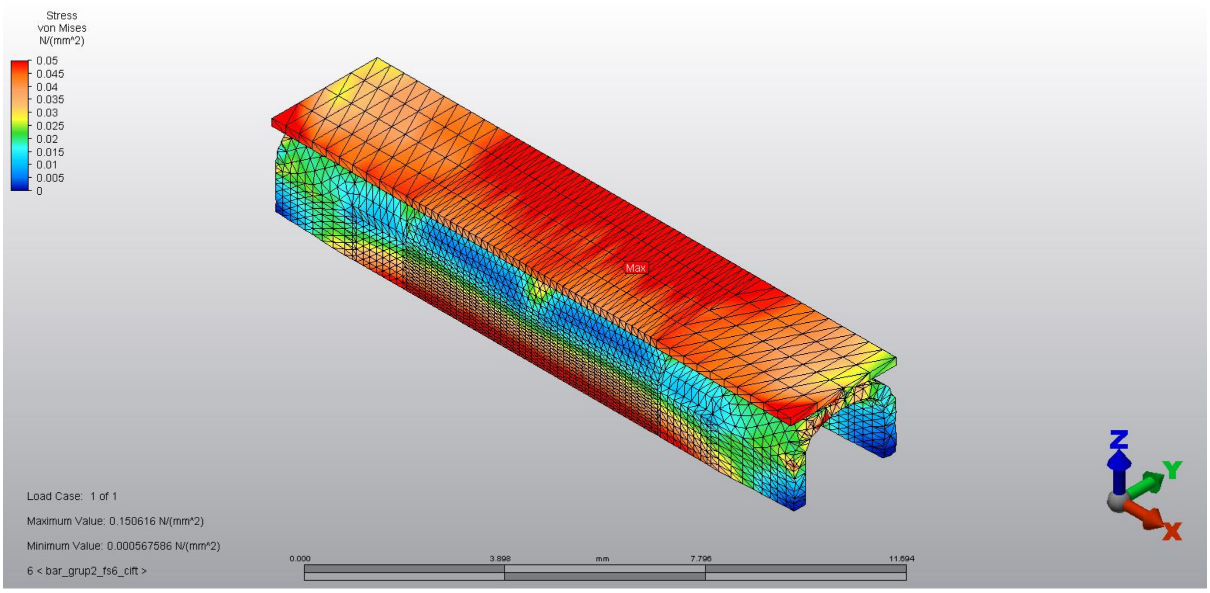
Stress
von Mises
N/(mm^2)
0.05
0.045
0.04
0.035
0.03
0.025
0.02
0.015
0.01
0.005
0
Max
Load Case: 1 of 1
Maximum Value: 0.150616 N/(mm^2)
Minimum Value: 0.000567586 N/(mm^2)
6 < bar_grup2_fs6_cift >
0.000
3.898
mm
7.796
11.694
Z
Y
X

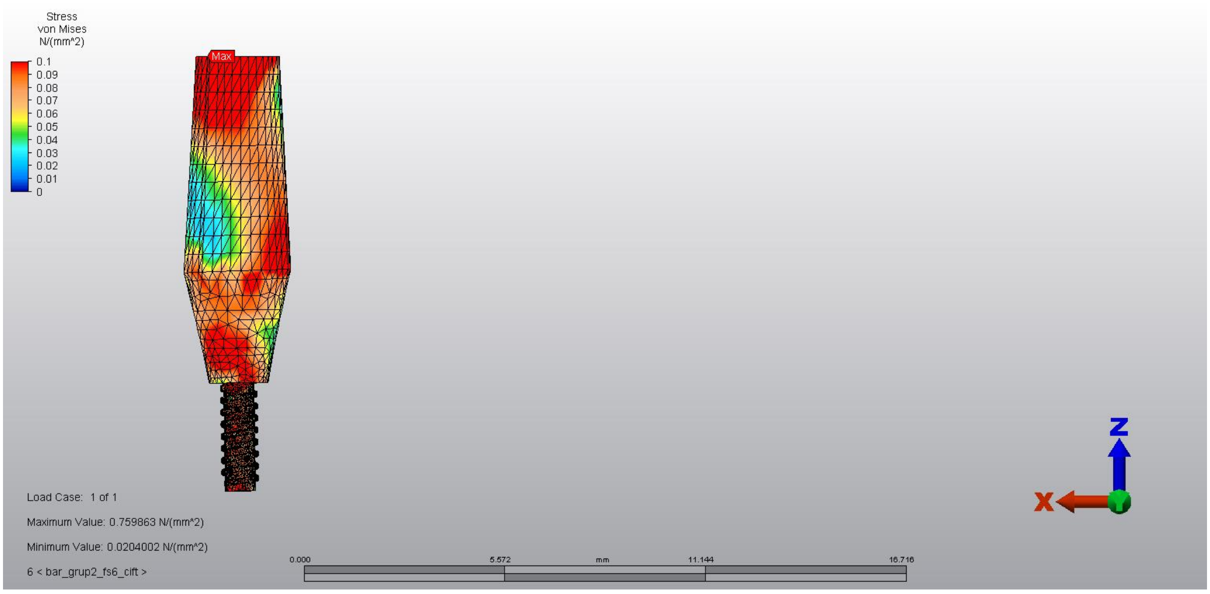
Stress
von Mises
N/(mm^2)
0.1
0.09
0.08
0.07
0.06
0.05
0.04
0.03
0.02
0.01
0
Max
Load Case: 1 of 1
Maximum Value: 0.759863 N/(mm^2)
Minimum Value: 0.0204002 N/(mm^2)
6 < bar_grup2_fs6_cift >
0.000
5.572
mm
11.144
16.716
Z
X

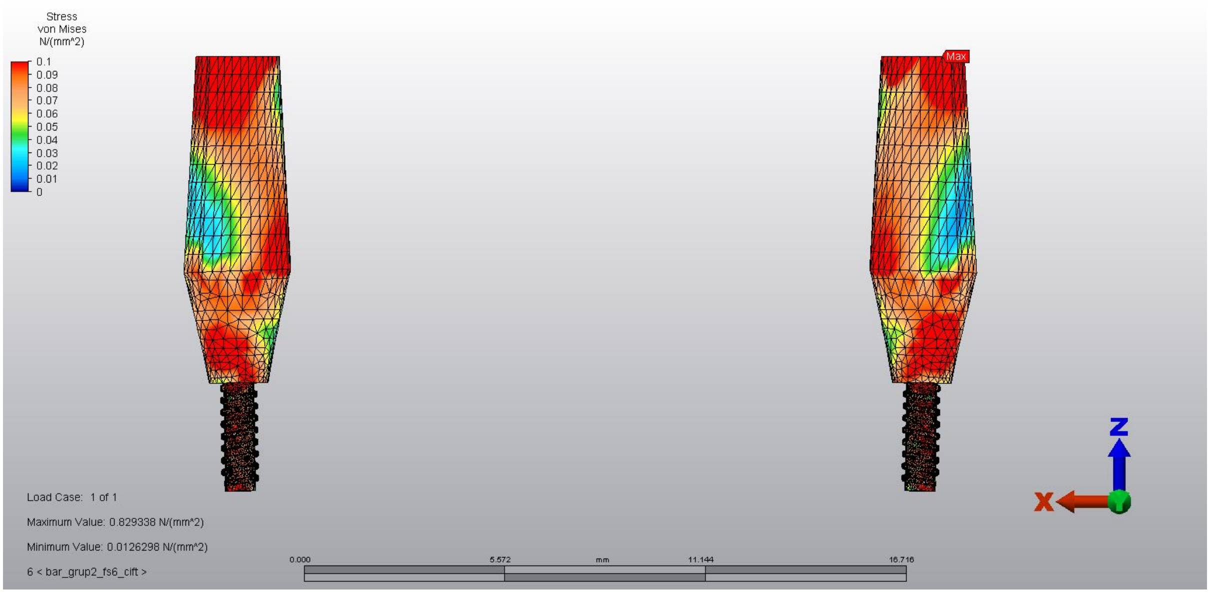
Stress
von Mises
N/(mm^2)
0.1
0.09
0.08
0.07
0.06
0.05
0.04
0.03
0.02
0.01
0
Max
Load Case: 1 of 1
Maximum Value: 0.829338 N/(mm^2)
Minimum Value: 0.0126298 N/(mm^2)
6 < bar_grup2_fs6_cift >
0.000
5.572
mm
11.144
16.716
Z
X

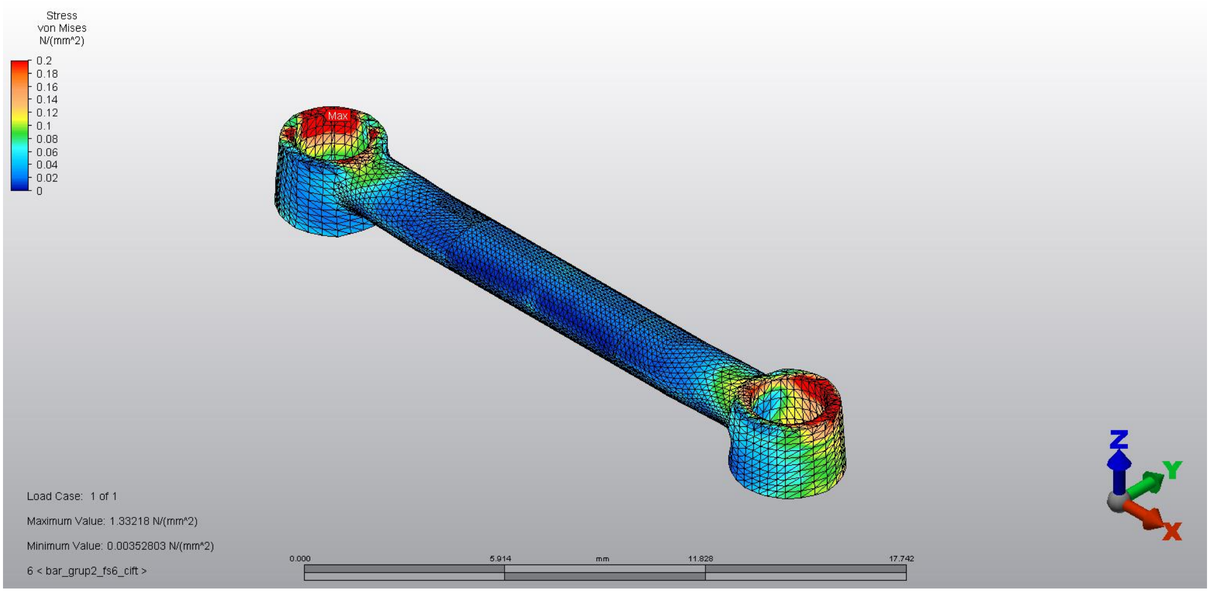
Stress
von Mises
N/(mm^2)
0.2
0.18
0.16
0.14
0.12
0.1
0.08
0.06
0.04
0.02
0
Max
Load Case: 1 of 1
Maximum Value: 1.33218 N/(mm^2)
Minimum Value: 0.00352803 N/(mm^2)
6 < bar_grup2_fs6_cift >
0.000
5.914
mm
11.828
17.742
Z
Y
X

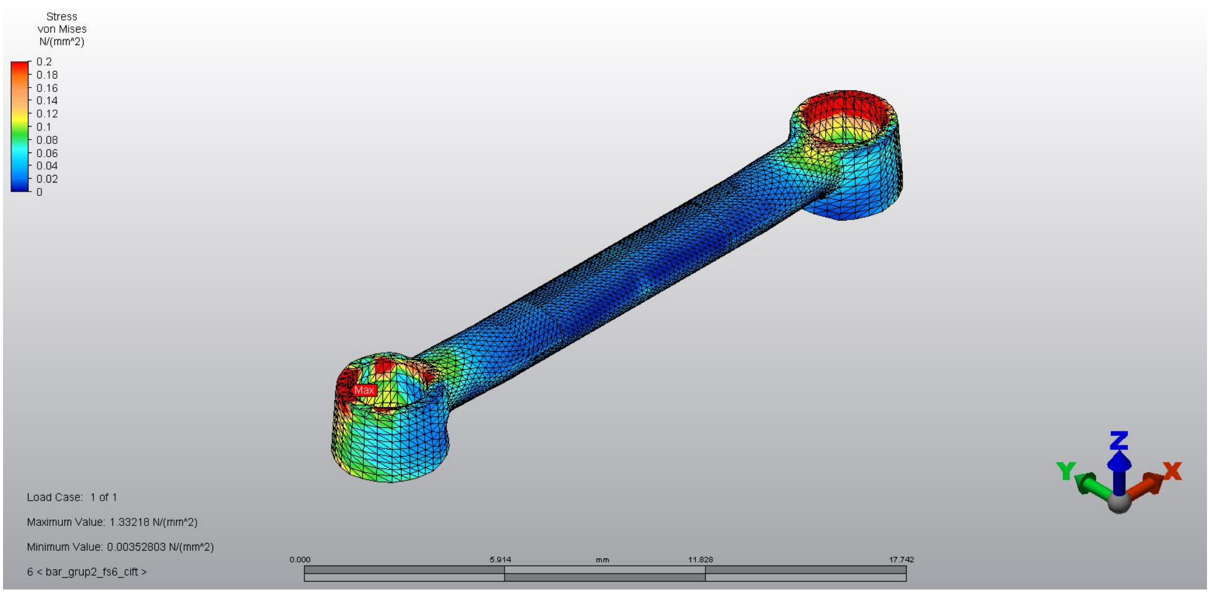
Stress
von Mises
N/(mm^2)
0.2
0.18
0.16
0.14
0.12
0.1
0.08
0.06
0.04
0.02
0
Max
Load Case: 1 of 1
Maximum Value: 1.33218 N/(mm^2)
Minimum Value: 0.00352803 N/(mm^2)
6 < bar_grup2_fs6_cift >
0.000
5.914
mm
11.828
17.742
Z
Y
X

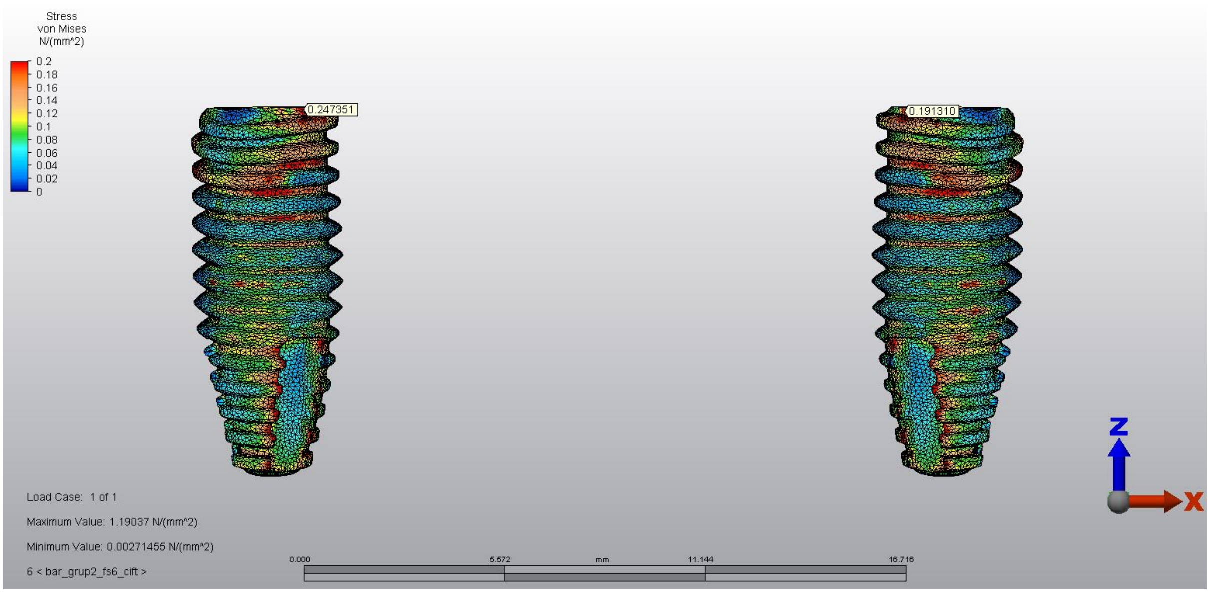
Stress
von Mises
N/(mm^2)
0.2
0.18
0.16
0.14
0.12
0.1
0.08
0.06
0.04
0.02
0
0.247351
0.191310
Load Case: 1 of 1
Maximum Value: 1.19037 N/(mm^2)
Minimum Value: 0.00271455 N/(mm^2)
6 < bar_grup2_fs6_cift >
0.000
5.572
mm
11.144
16.716
Z
X

UNILATERAL

Number of nodes = 160926

Number of elements = 792667

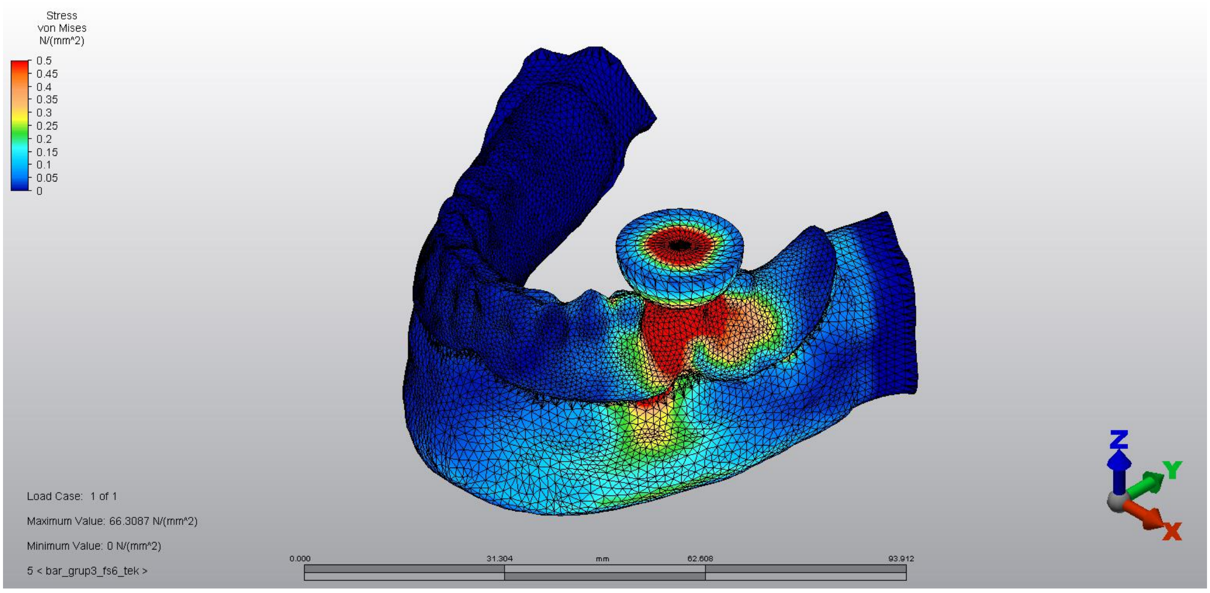

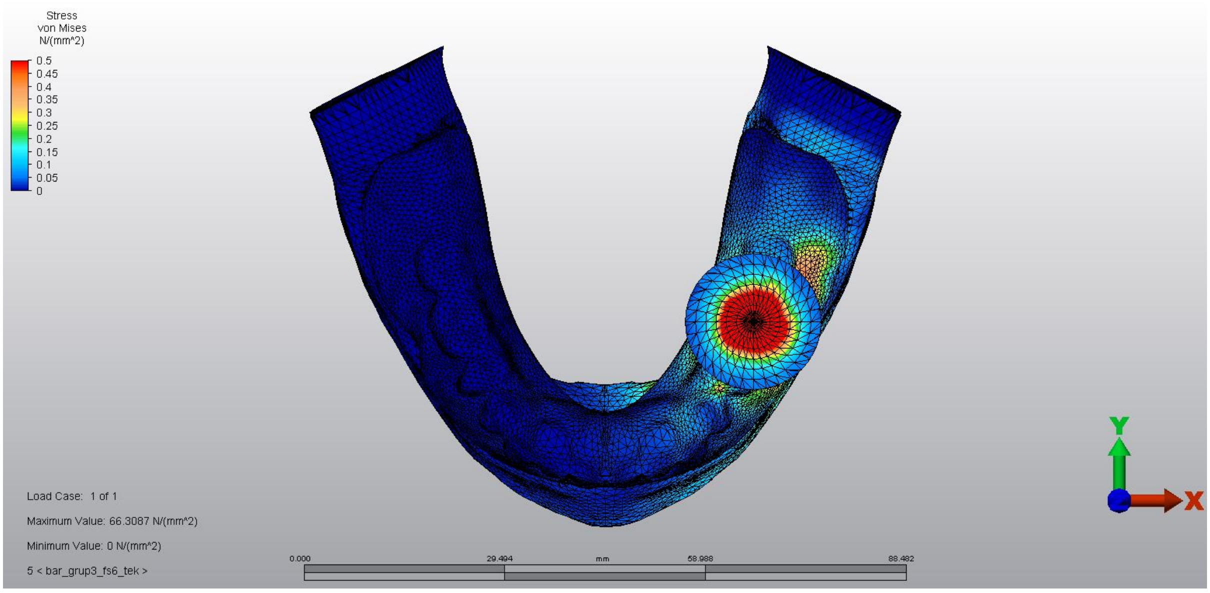

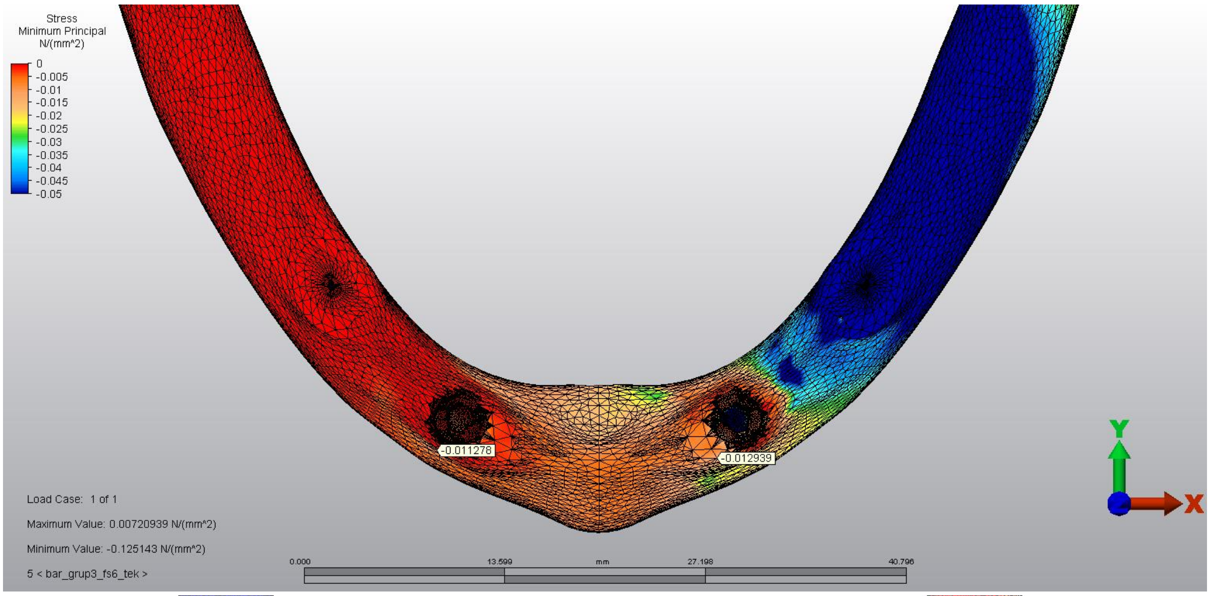
Stress
Minimum Principal
N/(mm^2)
0
-0.005
-0.01
-0.015
-0.02
-0.025
-0.03
-0.035
-0.04
-0.045
-0.05
-0.011278
-0.012939
Load Case: 1 of 1
Maximum Value: 0.00720939 N/(mm^2)
Minimum Value: -0.125143 N/(mm^2)
5 < bar_grup3_fs6_tek >
0.000
13.599
mm
27.198
40.796
Y
X

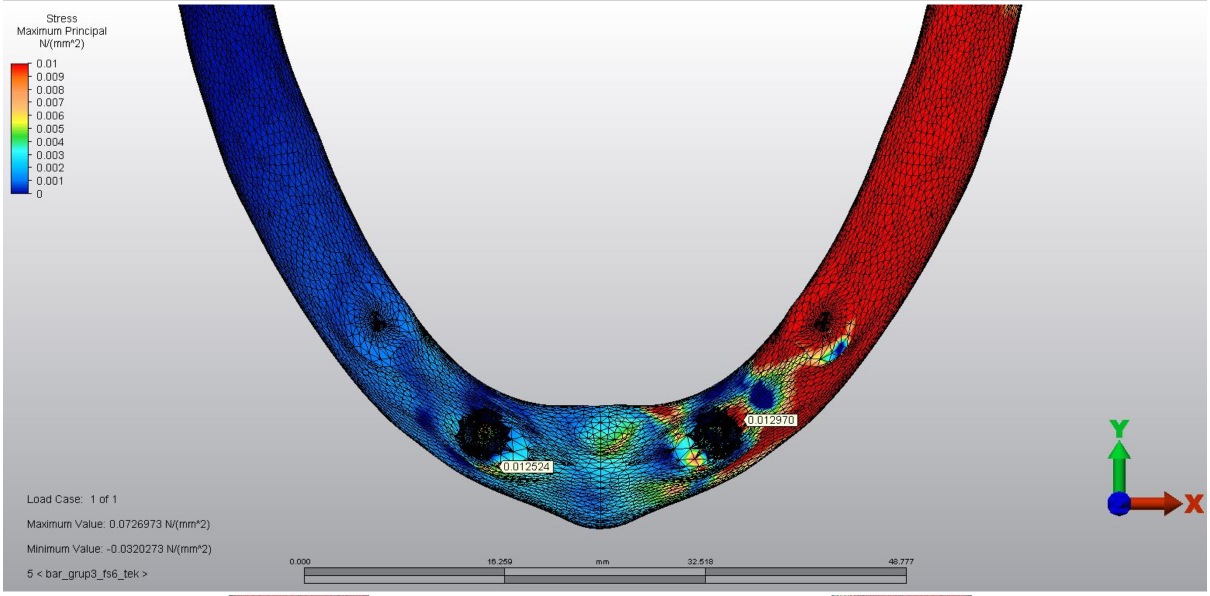
Stress
Maximum Principal
N/(mm^2)
0.01
0.009
0.008
0.007
0.006
0.005
0.004
0.003
0.002
0.001
0
0.012970
0.012524
Load Case: 1 of 1
Maximum Value: 0.0726973 N/(mm^2)
Minimum Value: -0.0320273 N/(mm^2)
5 < bar_grup3_fs6_tek >
0.000
16.259
mm
32.518
48.777
Y
X

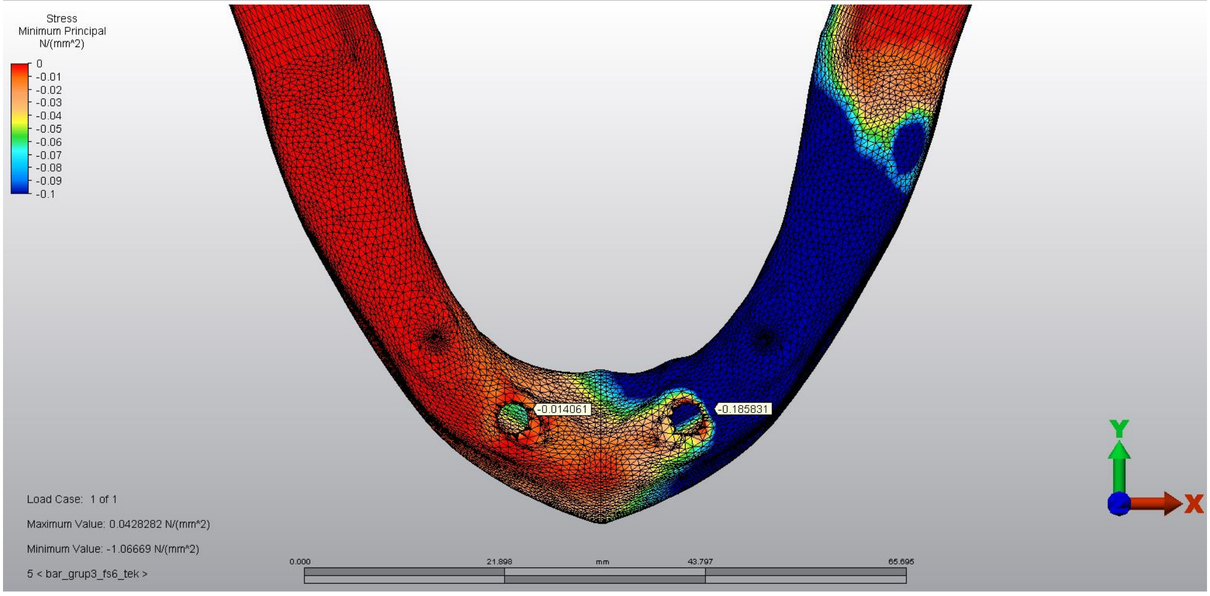
Stress
Minimum Principal
N/(mm^2)
0
-0.01
-0.02
-0.03
-0.04
-0.05
-0.06
-0.07
-0.08
-0.09
-0.1
-0.014061
-0.185831
Load Case: 1 of 1
Maximum Value: 0.0428282 N/(mm^2)
Minimum Value: -1.06669 N/(mm^2)
5 < bar_grup3_fs6_tek >
0.000
21.898
mm
43.797
65.695
Y
X

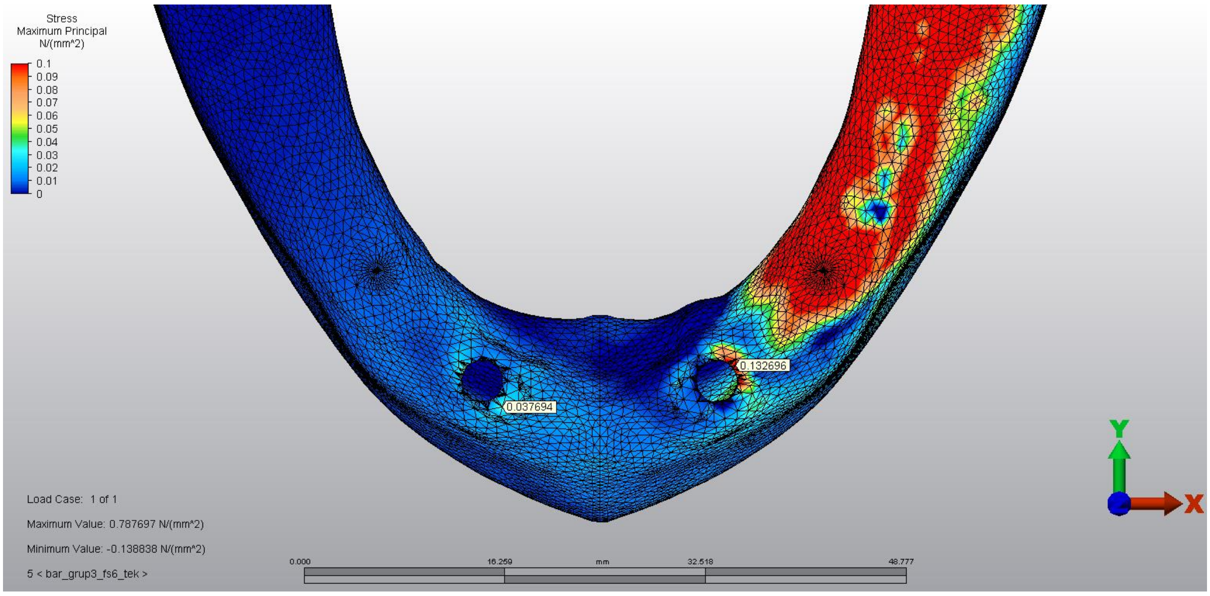
Stress
Maximum Principal
N/(mm^2)
0.1
0.09
0.08
0.07
0.06
0.05
0.04
0.03
0.02
0.01
0
0.132696
0.037694
Load Case: 1 of 1
Maximum Value: 0.787697 N/(mm^2)
Minimum Value: -0.138838 N/(mm^2)
5 < bar_grup3_fs6_tek >
0.000
16.259
mm
32.518
48.777
Y
X

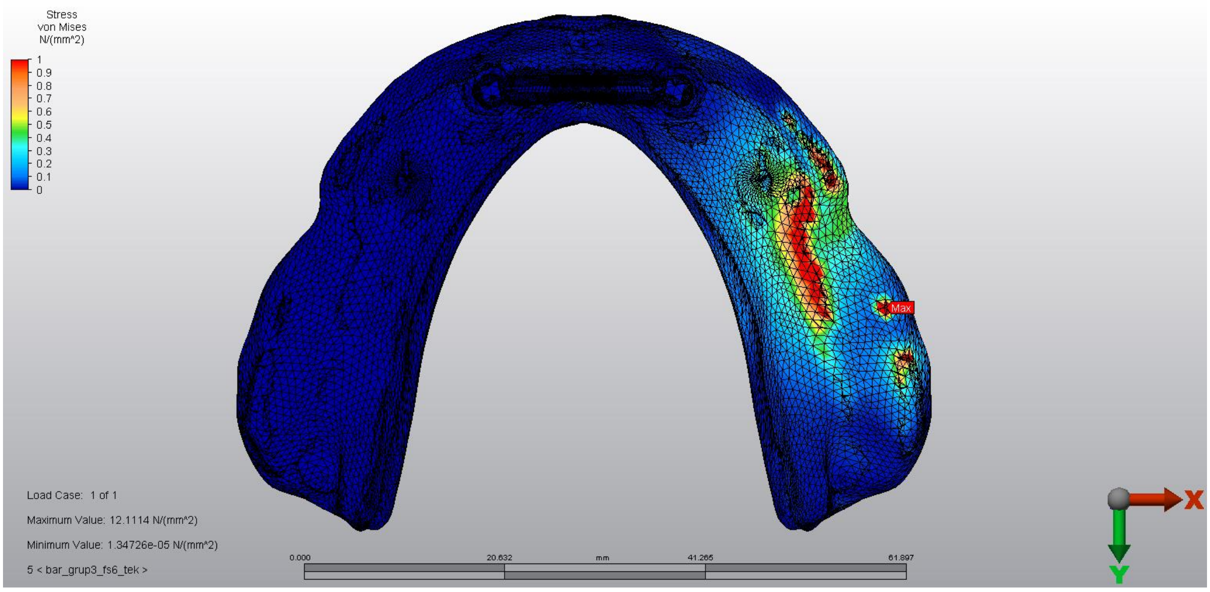
Stress
von Mises
N/(mm^2)
1
0.9
0.8
0.7
0.6
0.5
0.4
0.3
0.2
0.1
0
Max
Load Case: 1 of 1
Maximum Value: 12.1114 N/(mm^2)
Minimum Value: 1.34726e-05 N/(mm^2)
5 < bar_grup3_fs6_tek >
0.000
20.632
mm
41.265
61.897
X
Y

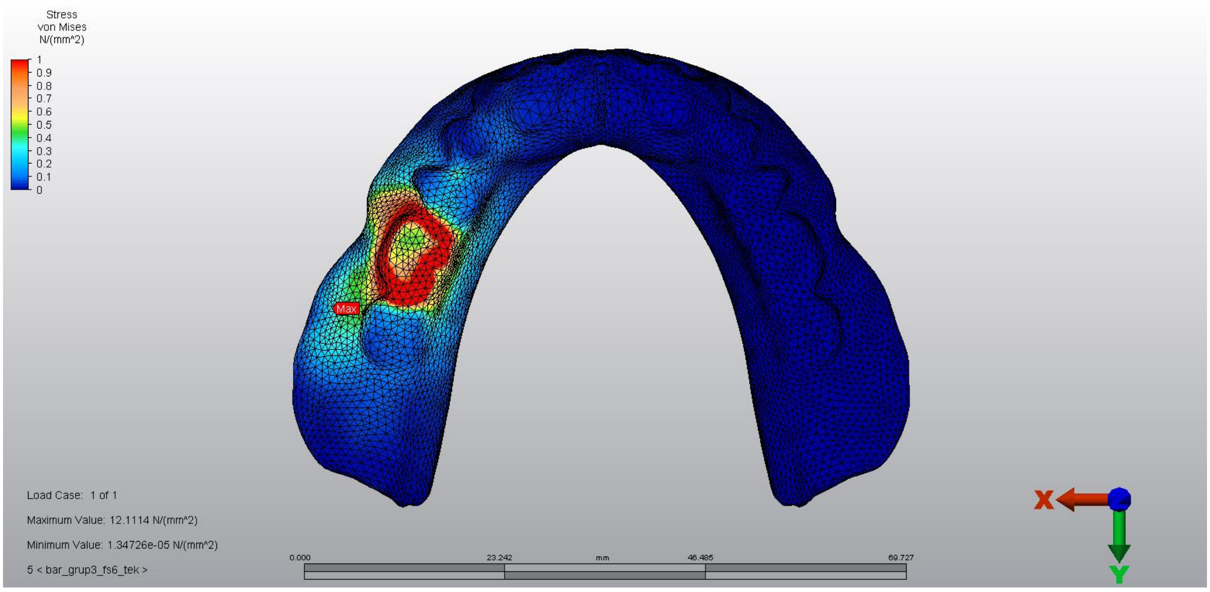
Stress
von Mises
N/(mm^2)
1
0.9
0.8
0.7
0.6
0.5
0.4
0.3
0.2
0.1
0
Max
Load Case: 1 of 1
Maximum Value: 12.1114 N/(mm^2)
Minimum Value: 1.34726e-05 N/(mm^2)
5 < bar_grup3_fs6_tek >
0.000
23.242
mm
46.485
69.727
X
Y

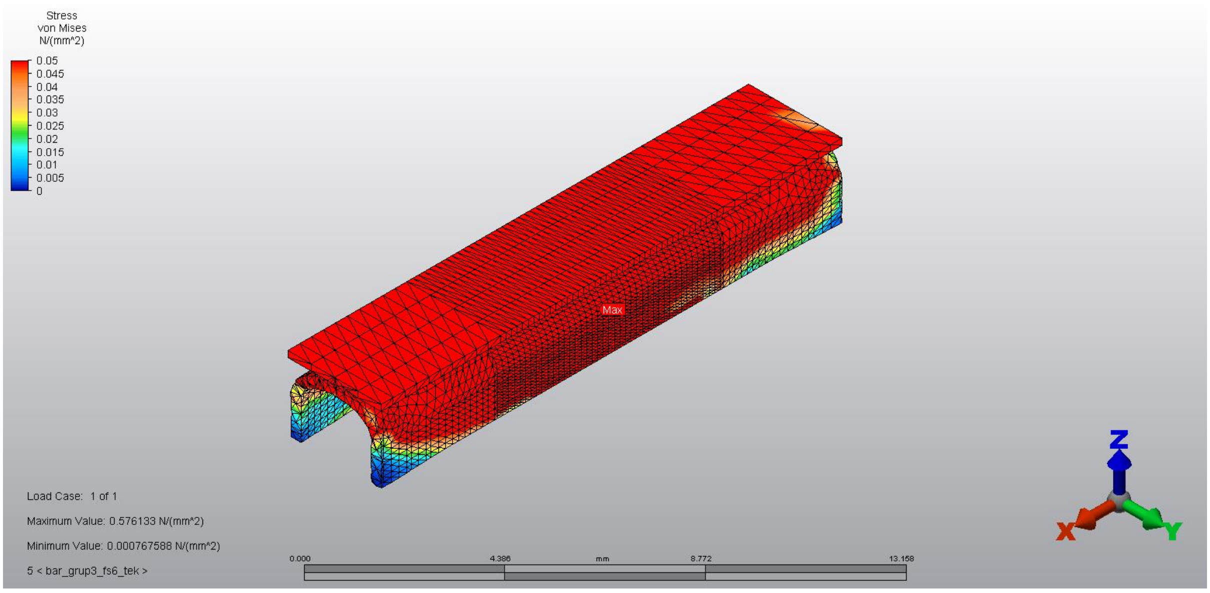
Stress
von Mises
N/(mm^2)
0.05
0.045
0.04
0.035
0.03
0.025
0.02
0.015
0.01
0.005
0
Max
Load Case: 1 of 1
Maximum Value: 0.576133 N/(mm^2)
Minimum Value: 0.000767588 N/(mm^2)
5 < bar_grup3_fs6_tek >
0.000
4.386
mm
8.772
13.158
Z
X
Y
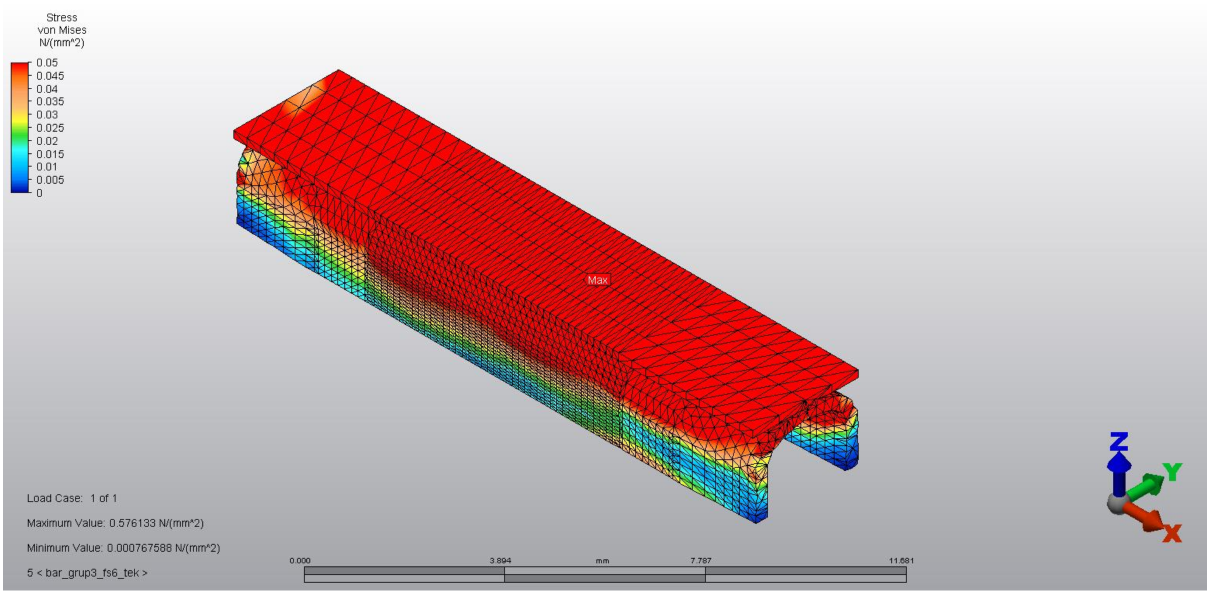
Stress
von Mises
N/(mm^2)
0.05
0.045
0.04
0.035
0.03
0.025
0.02
0.015
0.01
0.005
0
Max
Load Case: 1 of 1
Maximum Value: 0.576133 N/(mm^2)
Minimum Value: 0.000767588 N/(mm^2)
5 < bar_grup3_fs6_tek >
0.000
3.894
mm
7.787
11.681
Z
Y
X
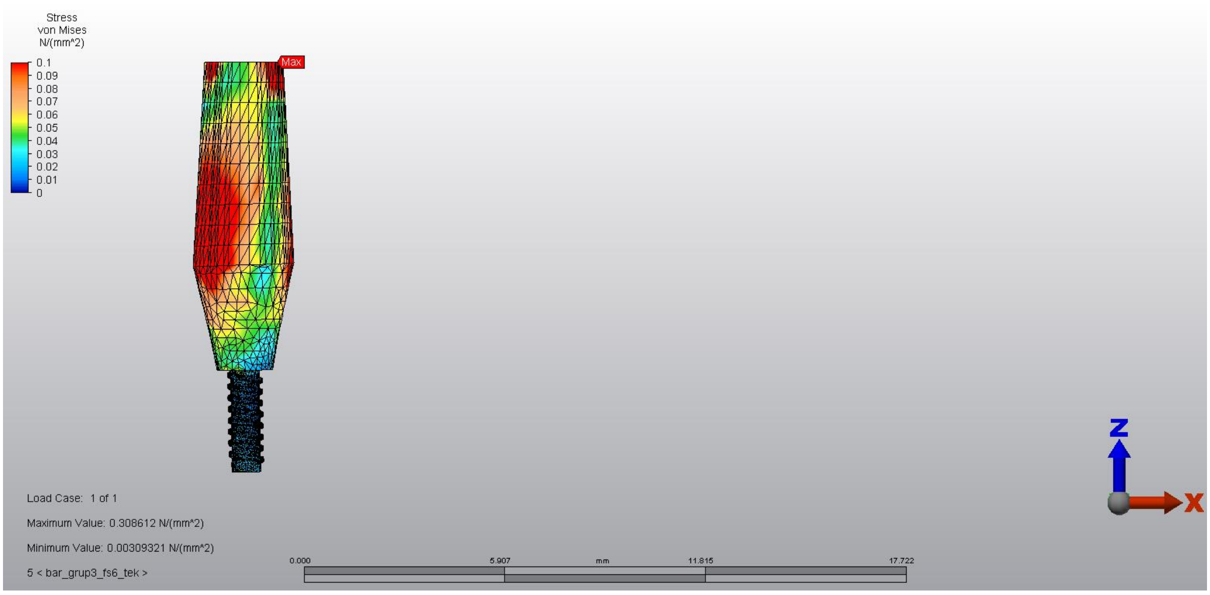
Stress
von Mises
N/(mm^2)
0.1
0.09
0.08
0.07
0.06
0.05
0.04
0.03
0.02
0.01
0
Max
Load Case: 1 of 1
Maximum Value: 0.308612 N/(mm^2)
Minimum Value: 0.00309321 N/(mm^2)
5 < bar_grup3_fs6_tek >
0.000
5.907
mm
11.815
17.722
Z
X

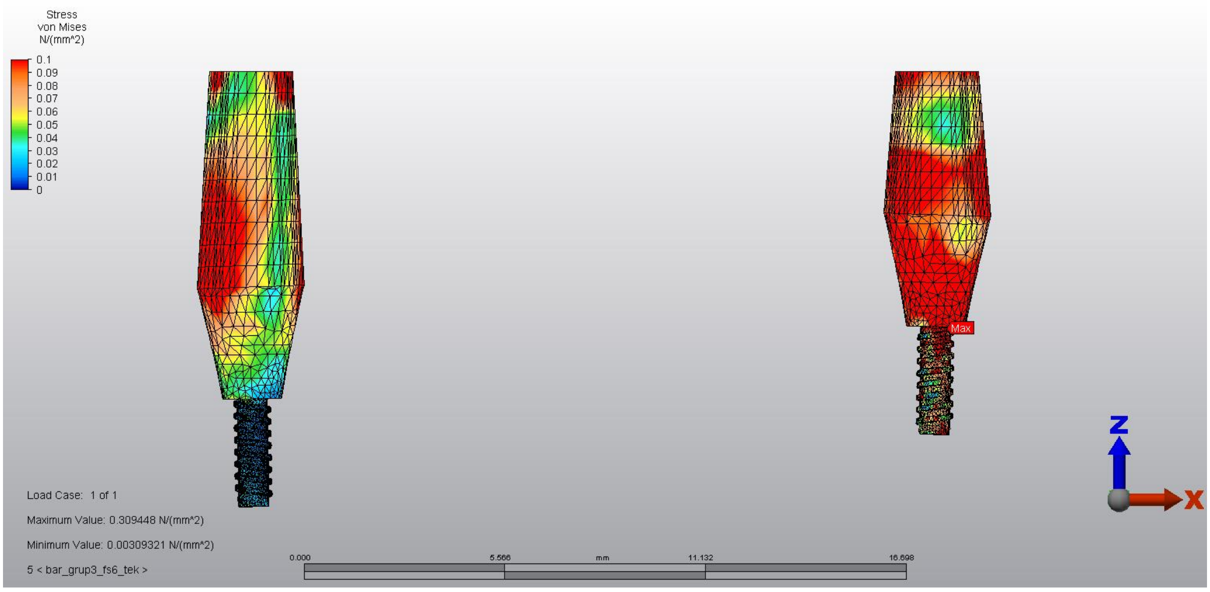
Stress
von Mises
N/(mm^2)
0.1
0.09
0.08
0.07
0.06
0.05
0.04
0.03
0.02
0.01
0
Max
Load Case: 1 of 1
Maximum Value: 0.309448 N/(mm^2)
Minimum Value: 0.00309321 N/(mm^2)
5 < bar_grup3_fs6_tek >
0.000
5.566
mm
11.132
16.698
Z
X

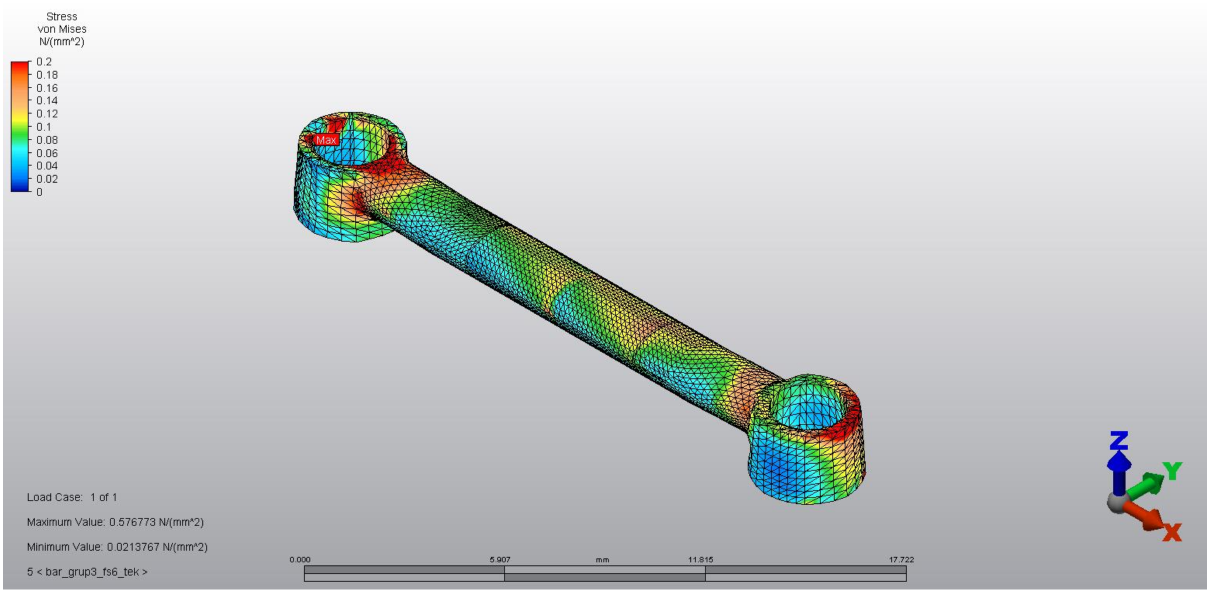
Stress
von Mises
N/(mm^2)
0.2
0.18
0.16
0.14
0.12
0.1
0.08
0.06
0.04
0.02
0
Max
Load Case: 1 of 1
Maximum Value: 0.576773 N/(mm^2)
Minimum Value: 0.0213767 N/(mm^2)
5 < bar_grup3_fs6_tek >
0.000
5.907
mm
11.815
17.722
Z
Y
X

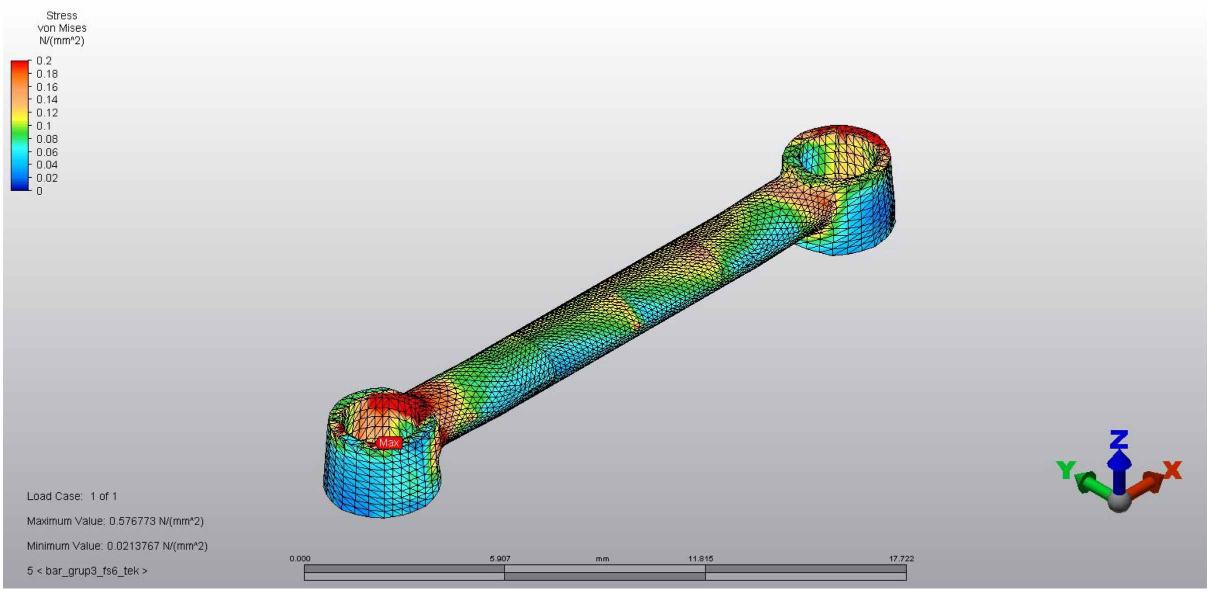

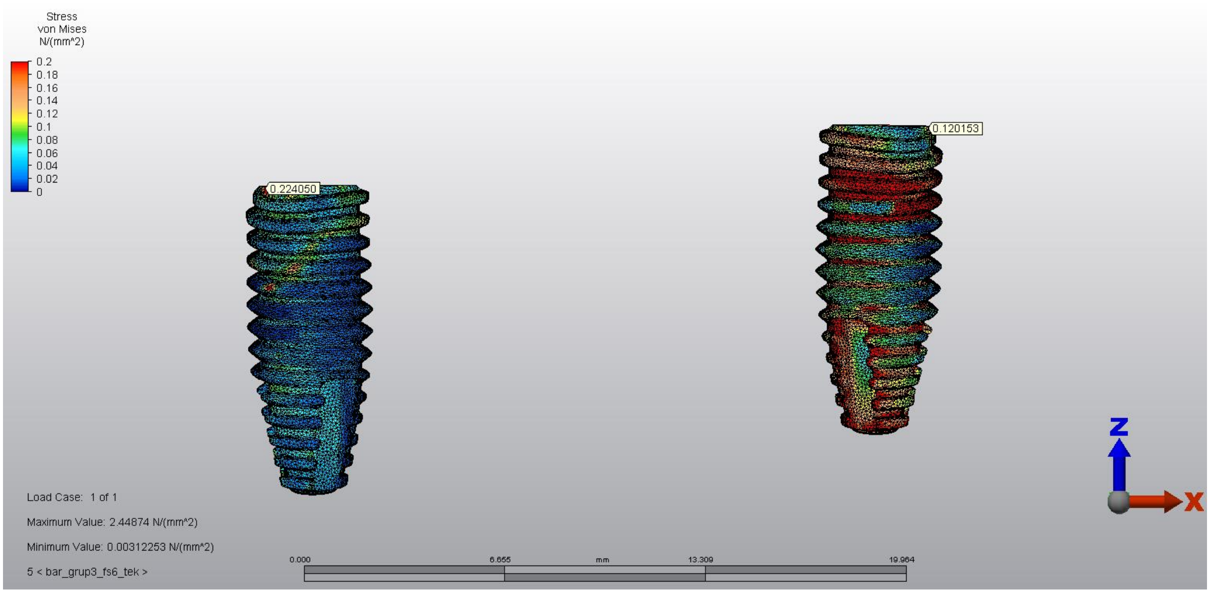

BILATERAL

Number of nodes = 161668

Number of elements = 795286

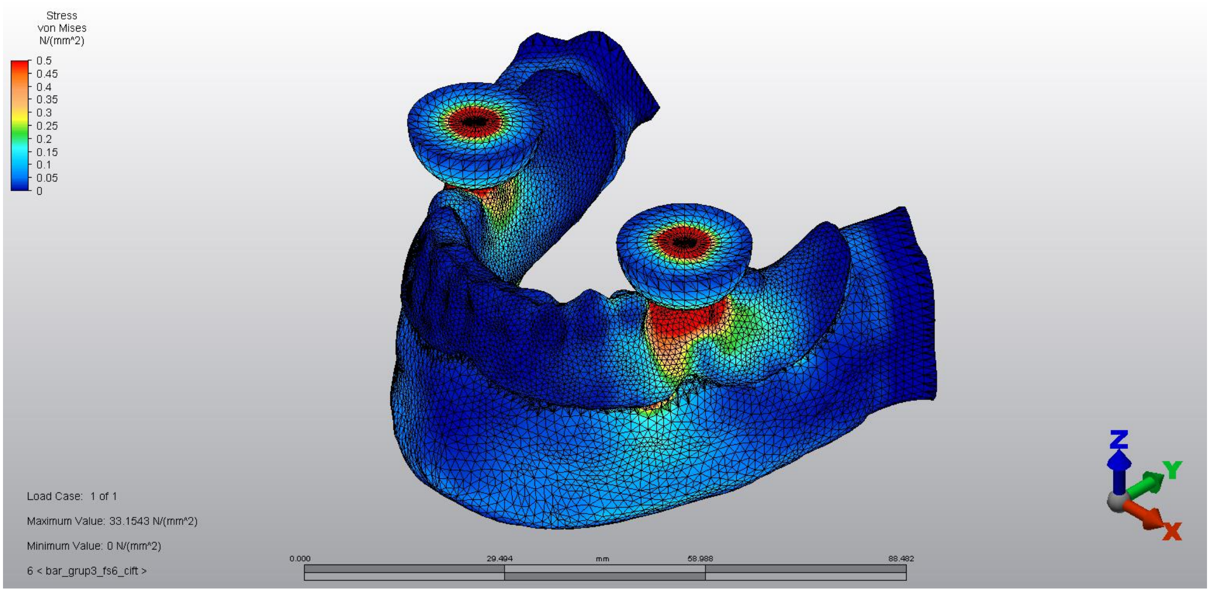
Stress
von Mises
N/(mm^2)
0.5
0.45
0.4
0.35
0.3
0.25
0.2
0.15
0.1
0.05
0
Load Case: 1 of 1
Maximum Value: 33.1543 N/(mm^2)
Minimum Value: 0 N/(mm^2)
6 < bar_grup3_fs6_cift >
0.000
29.494
mm
58.988
88.482
Z
Y
X

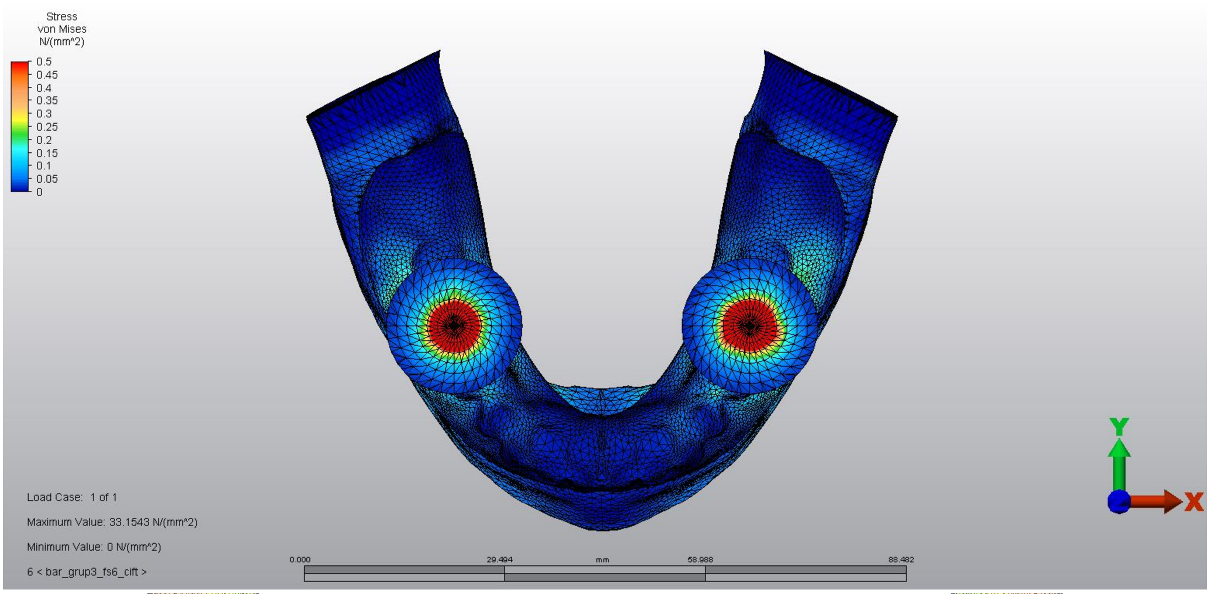
Stress
von Mises
N/(mm^2)
0.5
0.45
0.4
0.35
0.3
0.25
0.2
0.15
0.1
0.05
0
Load Case: 1 of 1
Maximum Value: 33.1543 N/(mm^2)
Minimum Value: 0 N/(mm^2)
6 < bar_grup3_fs6_cift >
0.000
29.494
mm
58.988
88.482
Y
X

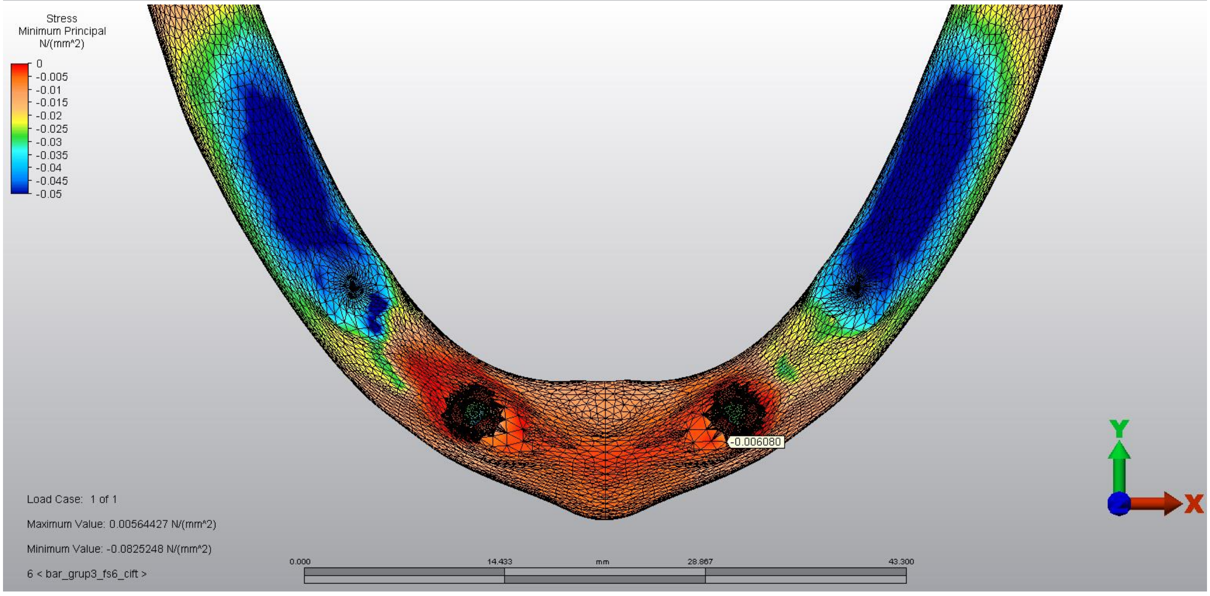
Stress
Minimum Principal
N/(mm^2)
0
-0.005
-0.01
-0.015
-0.02
-0.025
-0.03
-0.035
-0.04
-0.045
-0.05
-0.006080
Load Case: 1 of 1
Maximum Value: 0.00564427 N/(mm^2)
Minimum Value: -0.0825248 N/(mm^2)
6 < bar_grup3_fs6_cift >
0.000
14.433
mm
28.867
43.300
Y
X

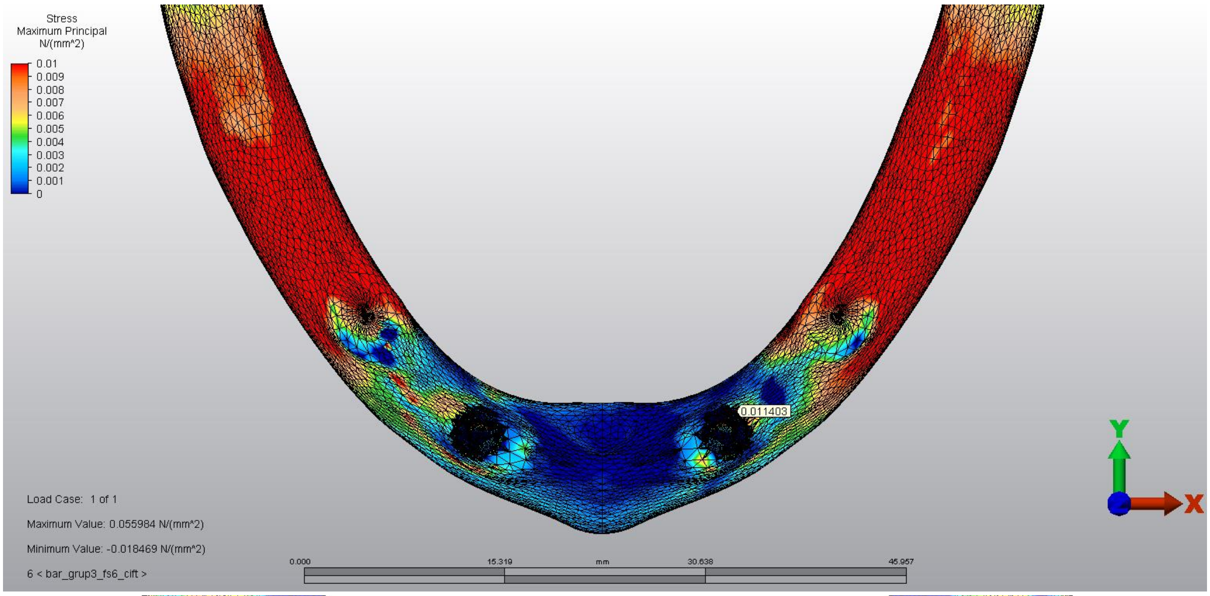
Stress
Maximum Principal
N/(mm^2)
0.01
0.009
0.008
0.007
0.006
0.005
0.004
0.003
0.002
0.001
0
0.011403
Load Case: 1 of 1
Maximum Value: 0.055984 N/(mm^2)
Minimum Value: -0.018469 N/(mm^2)
6 < bar_grup3_fs6_cift >
0.000
15.319
mm
30.638
45.957
Y
X

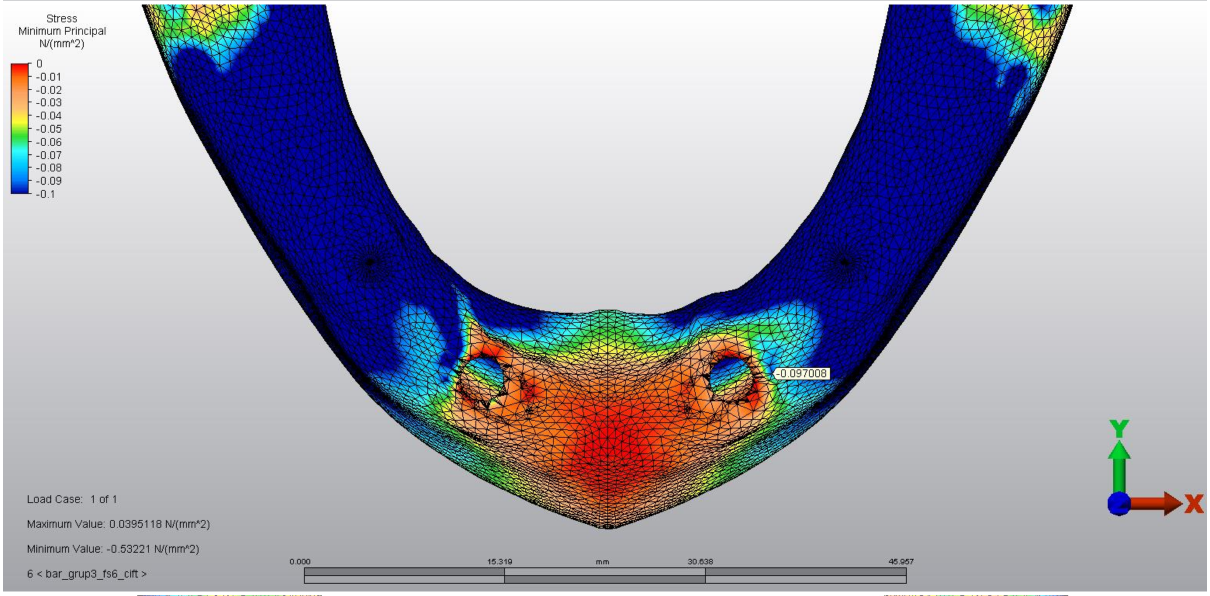
Stress
Minimum Principal
N/(mm^2)
0
-0.01
-0.02
-0.03
-0.04
-0.05
-0.06
-0.07
-0.08
-0.09
-0.1
-0.097008
Load Case: 1 of 1
Maximum Value: 0.0395118 N/(mm^2)
Minimum Value: -0.53221 N/(mm^2)
6 < bar_grup3_fs6_cift >
0.000
15.319
mm
30.638
45.957
Y
X

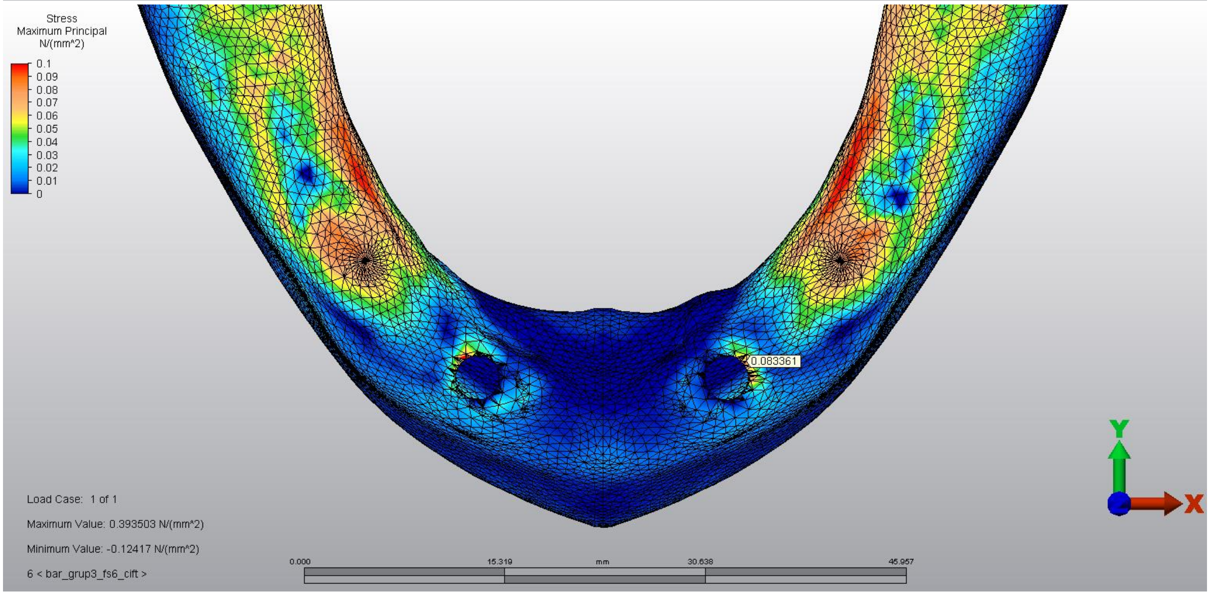
Stress
Maximum Principal
N/(mm^2)
0.1
0.09
0.08
0.07
0.06
0.05
0.04
0.03
0.02
0.01
0
0.083361
Load Case: 1 of 1
Maximum Value: 0.393503 N/(mm^2)
Minimum Value: -0.12417 N/(mm^2)
6 < bar_grup3_fs6_cift >
0.000
15.319
mm
30.638
45.957
Y
X

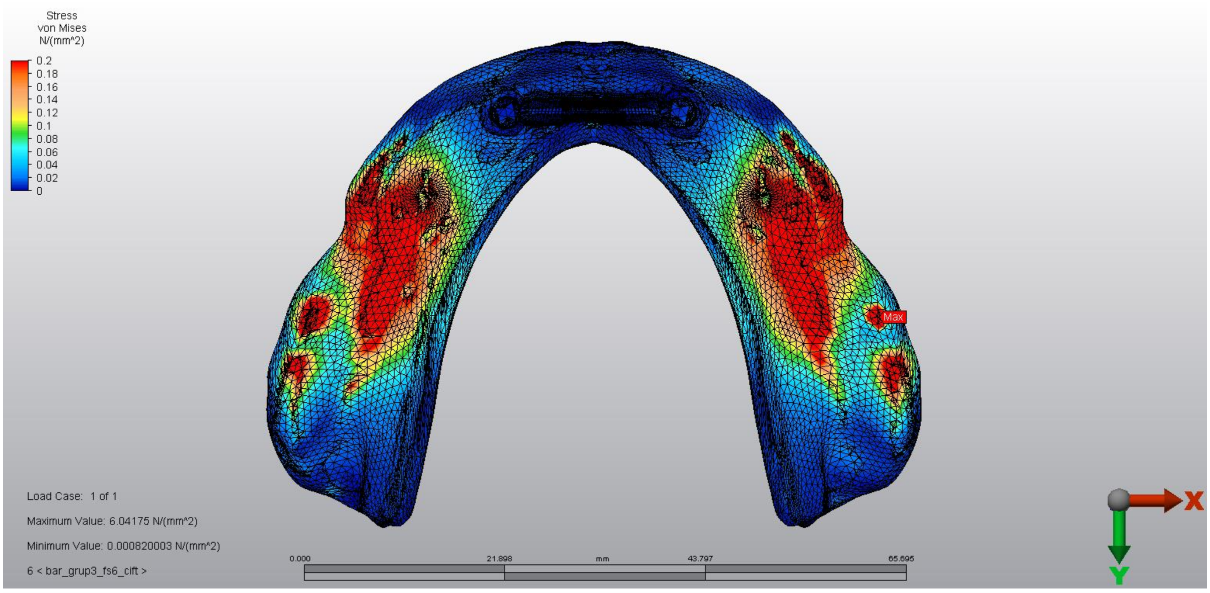
Stress
von Mises
N/(mm^2)
0.2
0.18
0.16
0.14
0.12
0.1
0.08
0.06
0.04
0.02
0
Max
Load Case: 1 of 1
Maximum Value: 6.04175 N/(mm^2)
Minimum Value: 0.000820003 N/(mm^2)
6 < bar_grup3_fs6_cift >
0.000
21.898
mm
43.797
65.695
X
Y

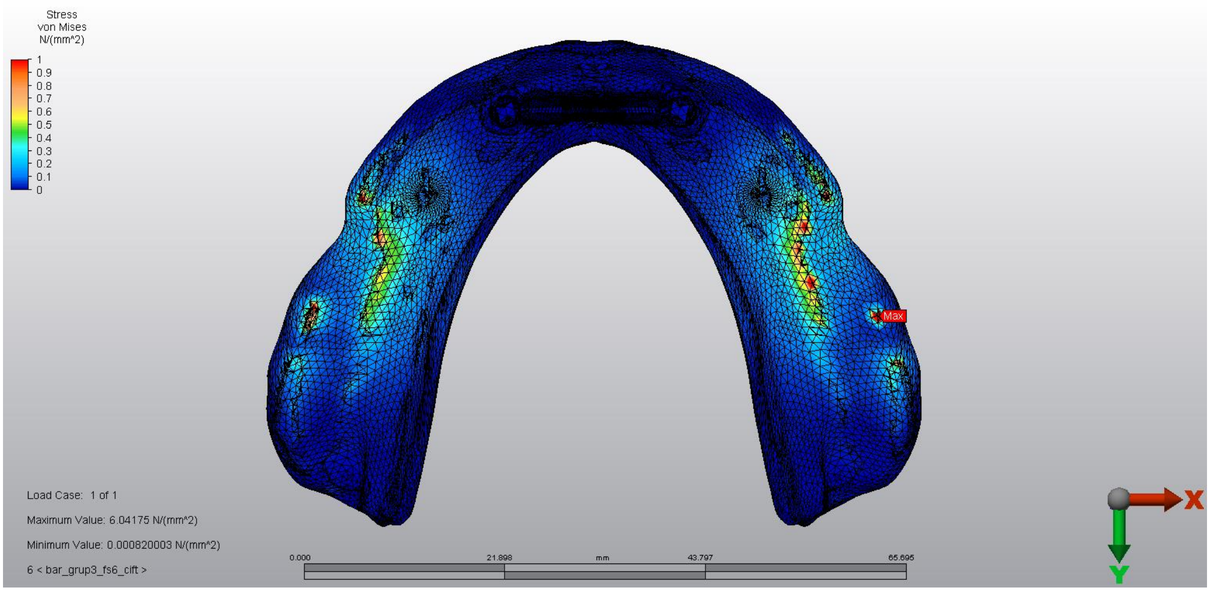
Stress
von Mises
N/(mm^2)
1
0.9
0.8
0.7
0.6
0.5
0.4
0.3
0.2
0.1
0
Max
Load Case: 1 of 1
Maximum Value: 6.04175 N/(mm^2)
Minimum Value: 0.000820003 N/(mm^2)
6 < bar_grup3_fs6_cift >
0.000
21.898
mm
43.797
65.695
X
Y

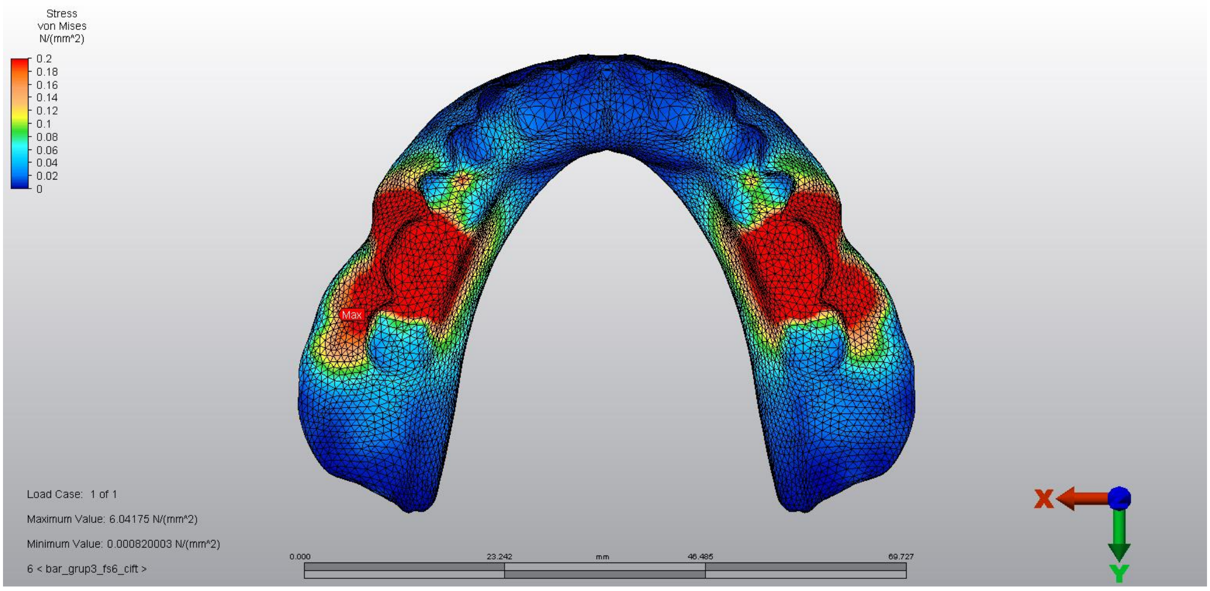
Stress
von Mises
N/(mm^2)
0.2
0.18
0.16
0.14
0.12
0.1
0.08
0.06
0.04
0.02
0
Max
Load Case: 1 of 1
Maximum Value: 6.04175 N/(mm^2)
Minimum Value: 0.000820003 N/(mm^2)
6 < bar_grup3_fs6_cift >
0.000
23.242
mm
46.485
69.727
X
Y

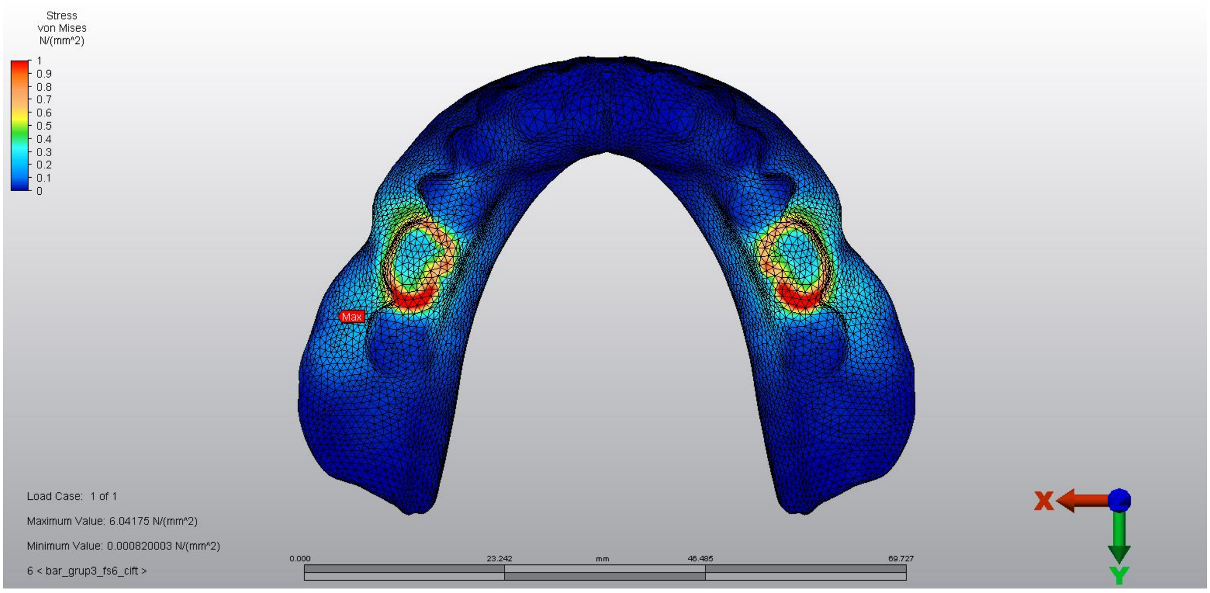
Stress
von Mises
N/(mm^2)
1
0.9
0.8
0.7
0.6
0.5
0.4
0.3
0.2
0.1
0
Max
Load Case: 1 of 1
Maximum Value: 6.04175 N/(mm^2)
Minimum Value: 0.000820003 N/(mm^2)
6 < bar_grup3_fs6_cift >
0.000
23.242
mm
46.485
69.727
X
Y

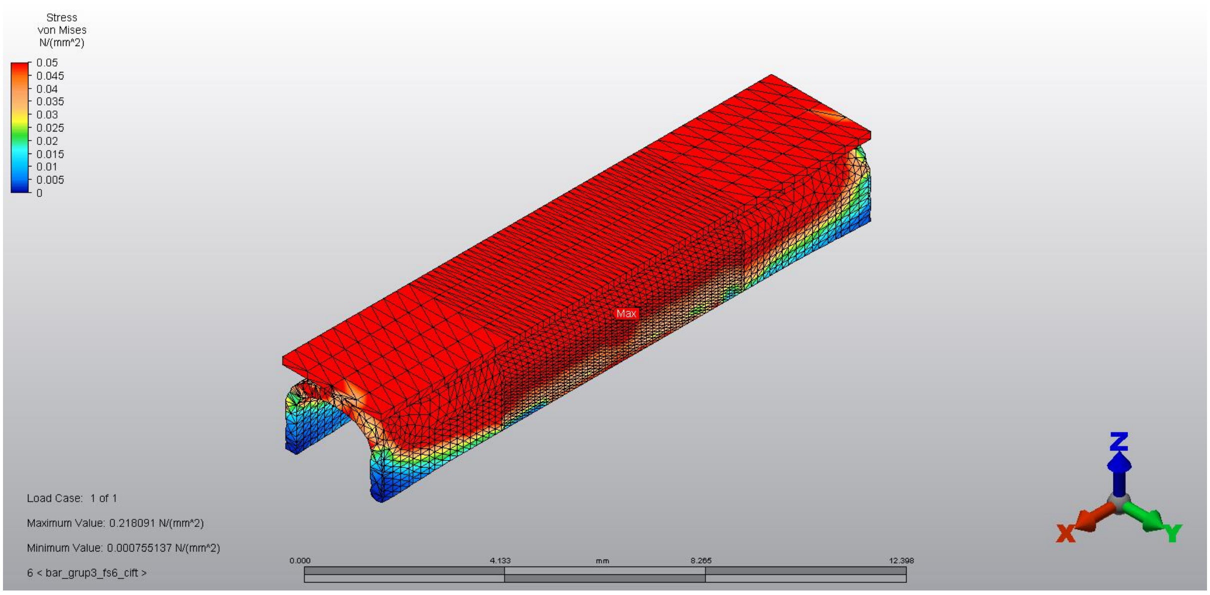
Stress
von Mises
N/(mm^2)
0.05
0.045
0.04
0.035
0.03
0.025
0.02
0.015
0.01
0.005
0
Max
Load Case: 1 of 1
Maximum Value: 0.218091 N/(mm^2)
Minimum Value: 0.000755137 N/(mm^2)
6 < bar_grup3_fs6_cift >
0.000
4.133
mm
8.265
12.398
Z
X
Y

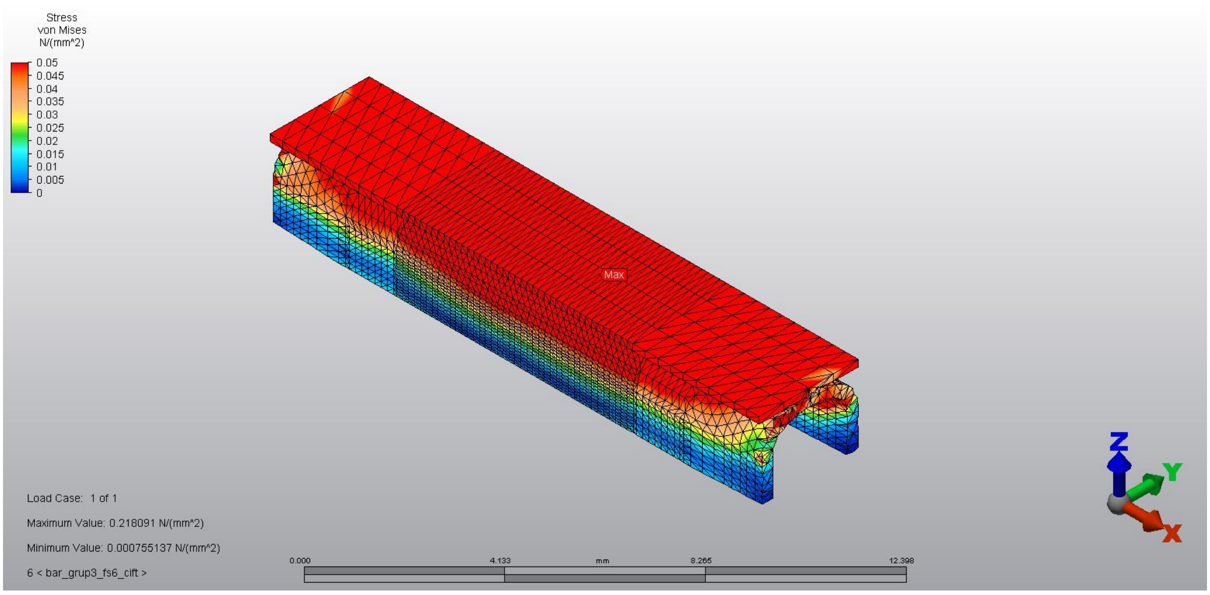
Stress
von Mises
N/(mm^2)
0.05
0.045
0.04
0.035
0.03
0.025
0.02
0.015
0.01
0.005
0
Max
Load Case: 1 of 1
Maximum Value: 0.218091 N/(mm^2)
Minimum Value: 0.000755137 N/(mm^2)
6 < bar_grup3_fs6_cift >
0.000
4.133
mm
8.265
12.398
Z
Y
X

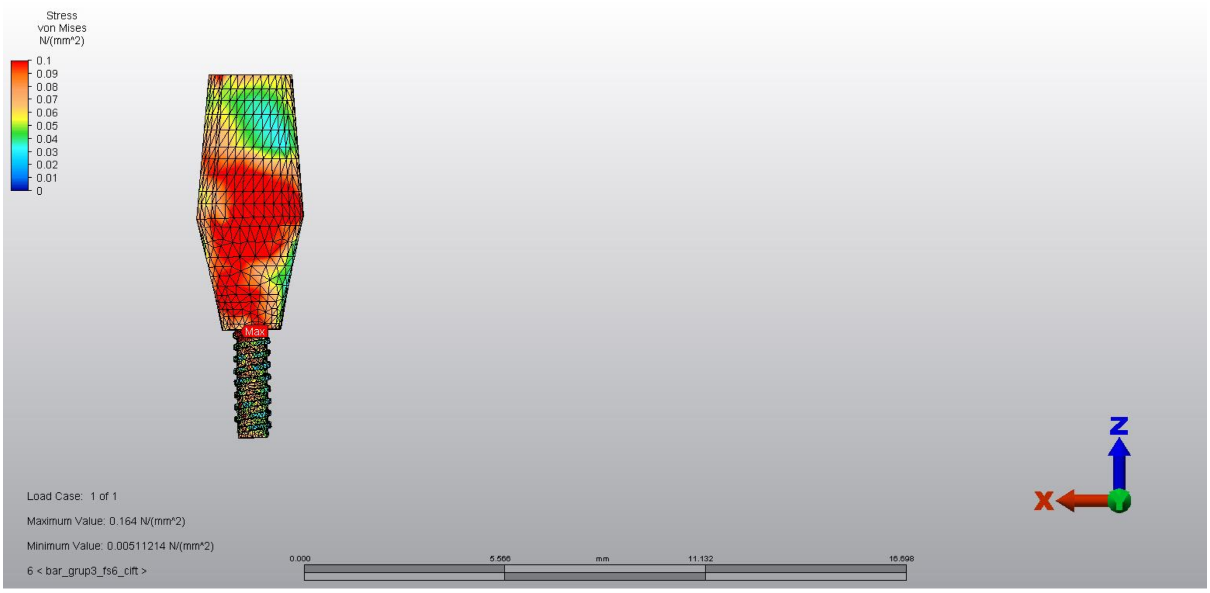
Stress
von Mises
N/(mm^2)
0.1
0.09
0.08
0.07
0.06
0.05
0.04
0.03
0.02
0.01
0
Max
Load Case: 1 of 1
Maximum Value: 0.164 N/(mm^2)
Minimum Value: 0.00511214 N/(mm^2)
6 < bar_grup3_fs6_cift >
0.000
5.566
mm
11.132
16.698
Z
X

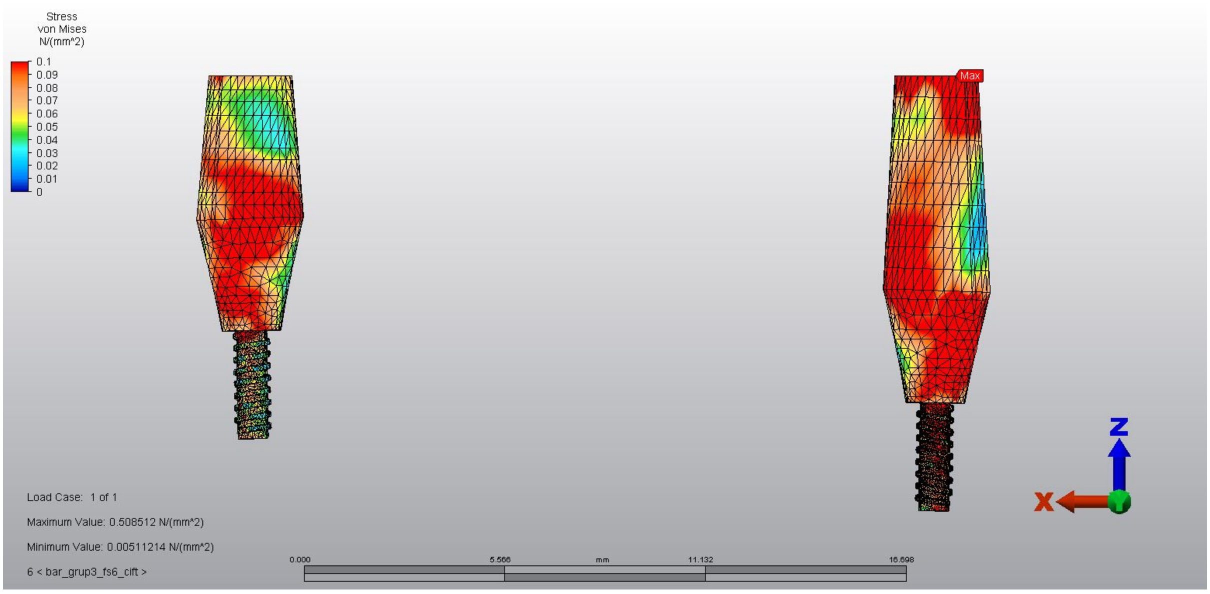
Stress
von Mises
N/(mm^2)
0.1
0.09
0.08
0.07
0.06
0.05
0.04
0.03
0.02
0.01
0
Max
Load Case: 1 of 1
Maximum Value: 0.508512 N/(mm^2)
Minimum Value: 0.00511214 N/(mm^2)
6 < bar_grup3_fs6_cift >
0.000
5.566
mm
11.132
16.698
Z
X

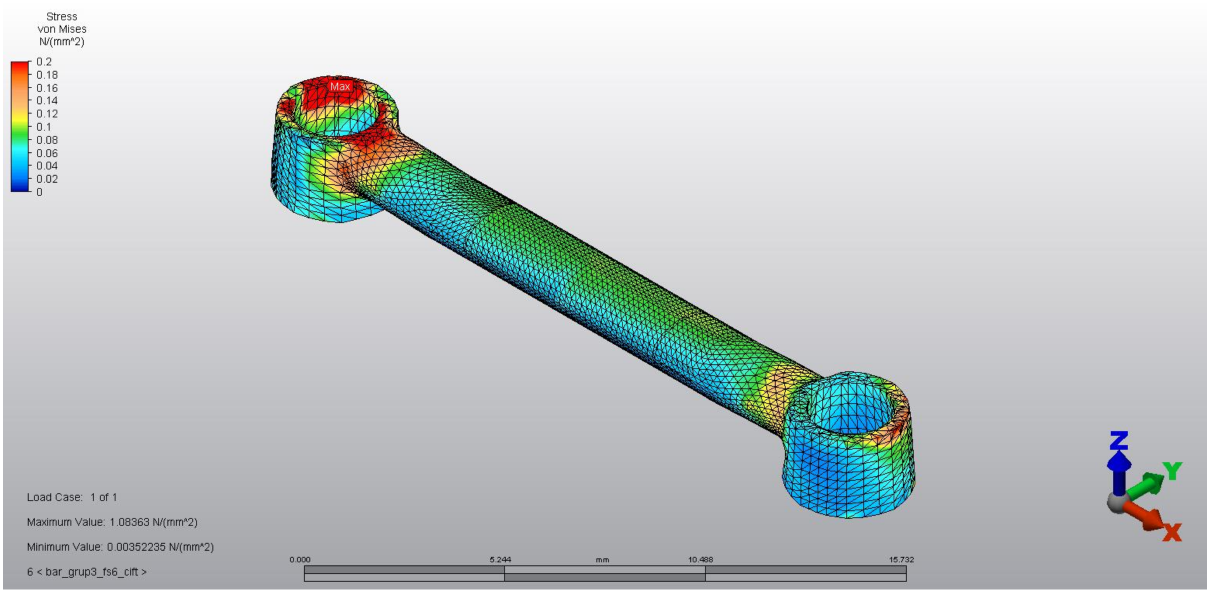
Stress
von Mises
N/(mm^2)
0.2
0.18
0.16
0.14
0.12
0.1
0.08
0.06
0.04
0.02
0
Max
Load Case: 1 of 1
Maximum Value: 1.08363 N/(mm^2)
Minimum Value: 0.00352235 N/(mm^2)
6 < bar_grup3_fs6_cift >
0.000
5.244
mm
10.488
15.732
Z
Y
X

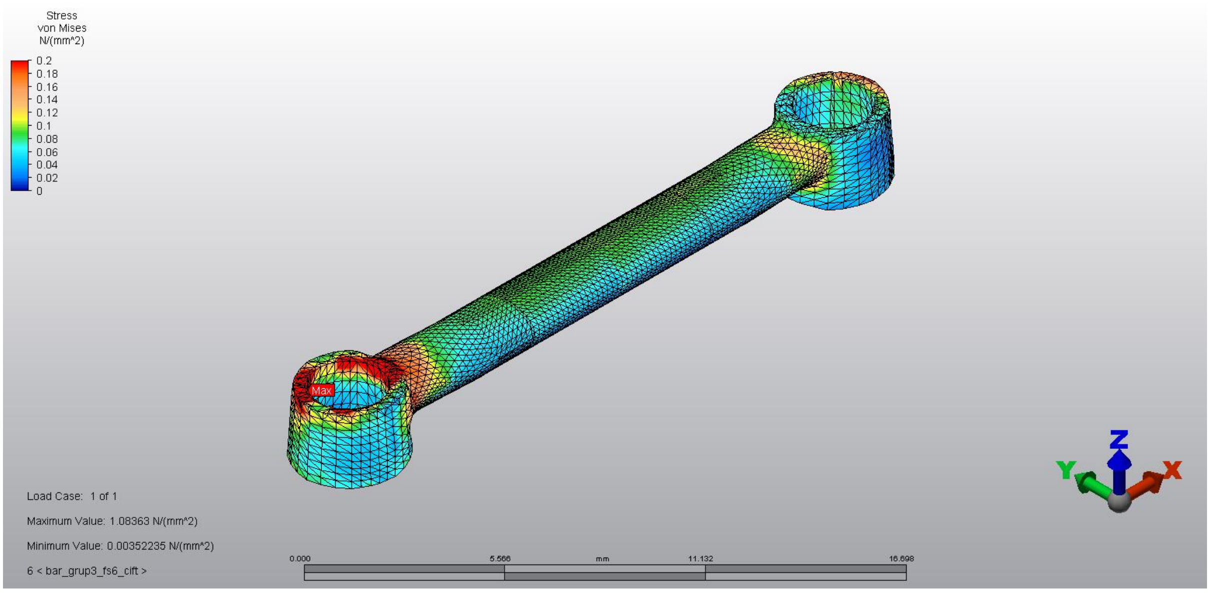
Stress
von Mises
N/(mm^2)
0.2
0.18
0.16
0.14
0.12
0.1
0.08
0.06
0.04
0.02
0
Max
Load Case: 1 of 1
Maximum Value: 1.08363 N/(mm^2)
Minimum Value: 0.00352235 N/(mm^2)
6 < bar_grup3_fs6_cift >
0.000
5.566
mm
11.132
16.698
Z
Y
X

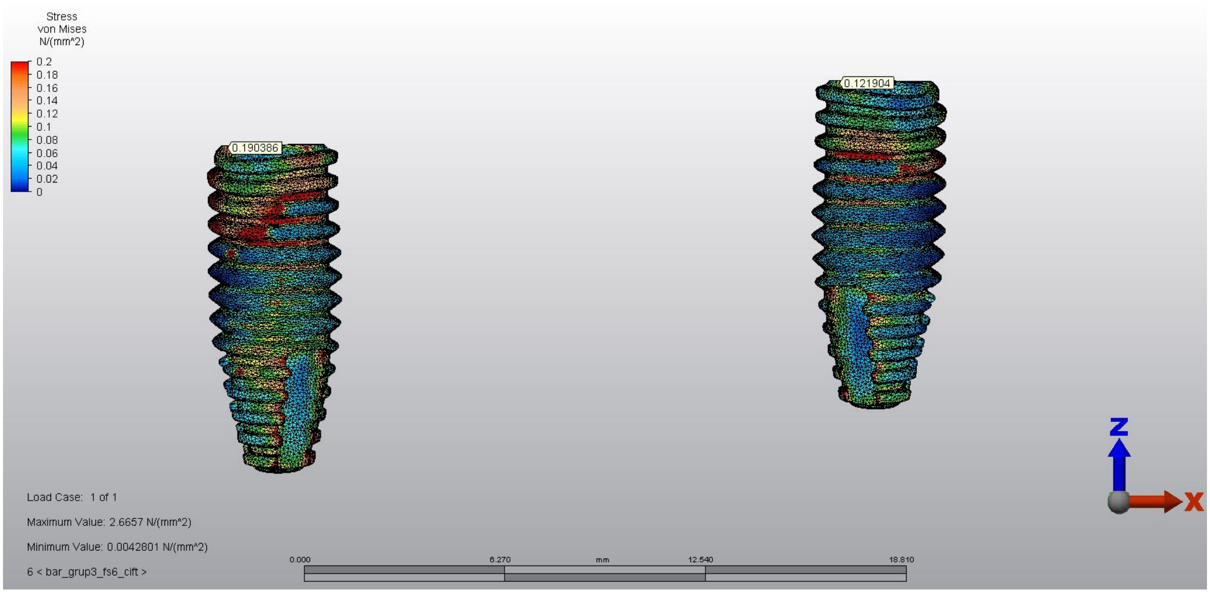
Stress
von Mises
N/(mm^2)
0.2
0.18
0.16
0.14
0.12
0.1
0.08
0.06
0.04
0.02
0
0.190386
0.121904
Load Case: 1 of 1
Maximum Value: 2.6657 N/(mm^2)
Minimum Value: 0.0042801 N/(mm^2)
6 < bar_grup3_fs6_cift >
0.000
6.270
mm
12.540
18.810
Z
X
