# Supplementary material for: The role of attachment type and bone height in modulating stress distribution in mandibular overdentures: Insights from finite element analysis
Source: PLoS One. 2026 Jun 16;21(6):e0351498. doi: 10.1371/journal.pone.0351498 (PMC13271450; doi:10.1371/journal.pone.0351498)
Supplement: S2 File — This PDF file contains stress distribution images of implants, attachment components, prosthesis, tissue layers and loading conditions of all locator attachment groups. (PDF) [file pone.0351498.s002.pdf]

LOCATOR

GRUP 01

INCISAL

Number of nodes = 135800

Number of elements = 713589

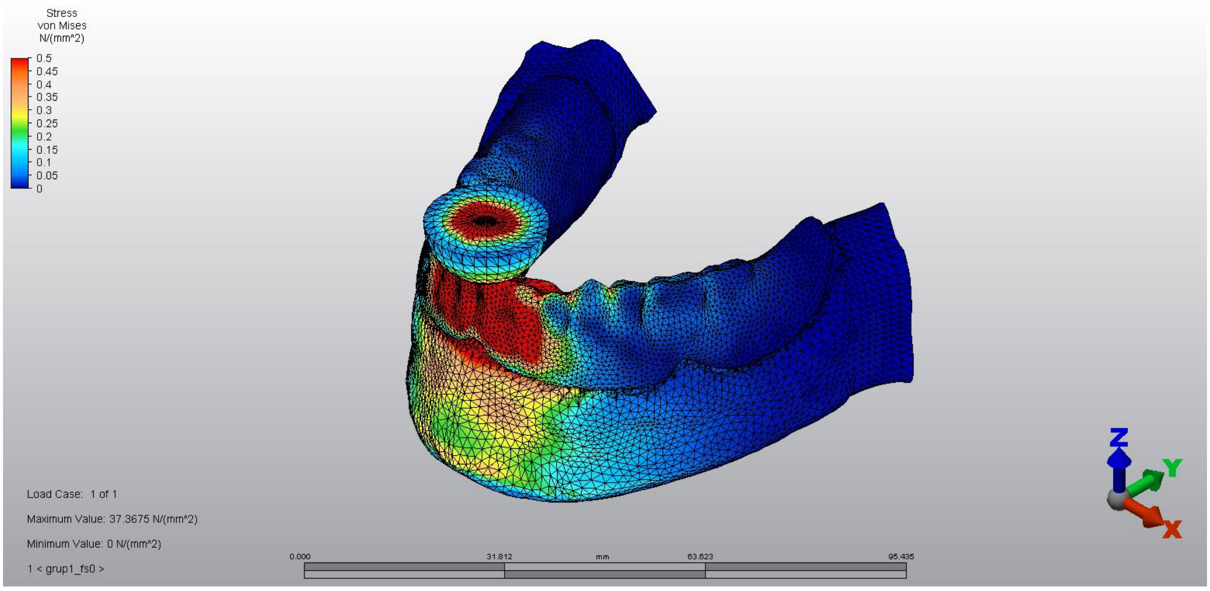

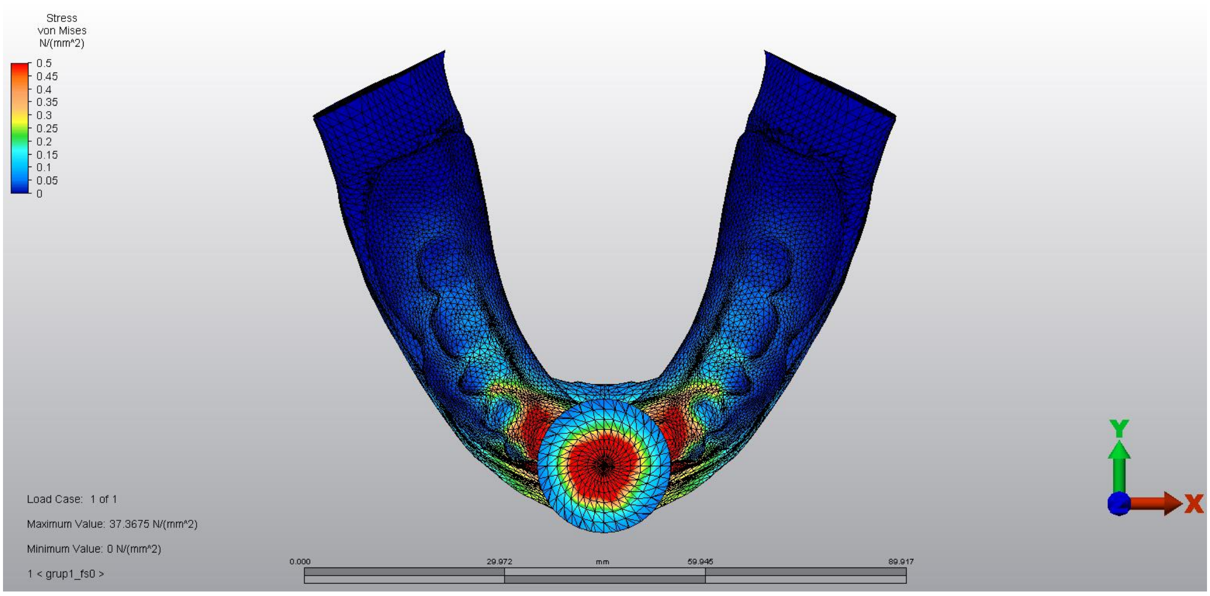

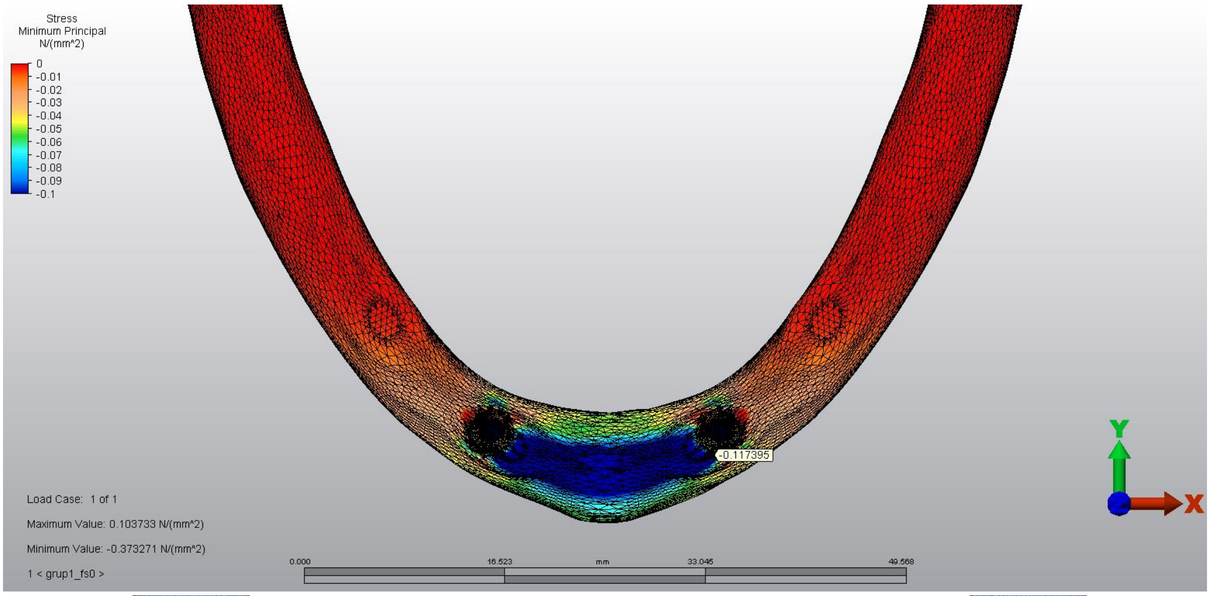
Stress
Minimum Principal
N/(mm^2)
0
-0.01
-0.02
-0.03
-0.04
-0.05
-0.06
-0.07
-0.08
-0.09
-0.1
-0.117395
Load Case: 1 of 1
Maximum Value: 0.103733 N/(mm^2)
Minimum Value: -0.373271 N/(mm^2)
1 < grup1_fs0 >
0.000
16.523
mm
33.045
49.568
Y
X

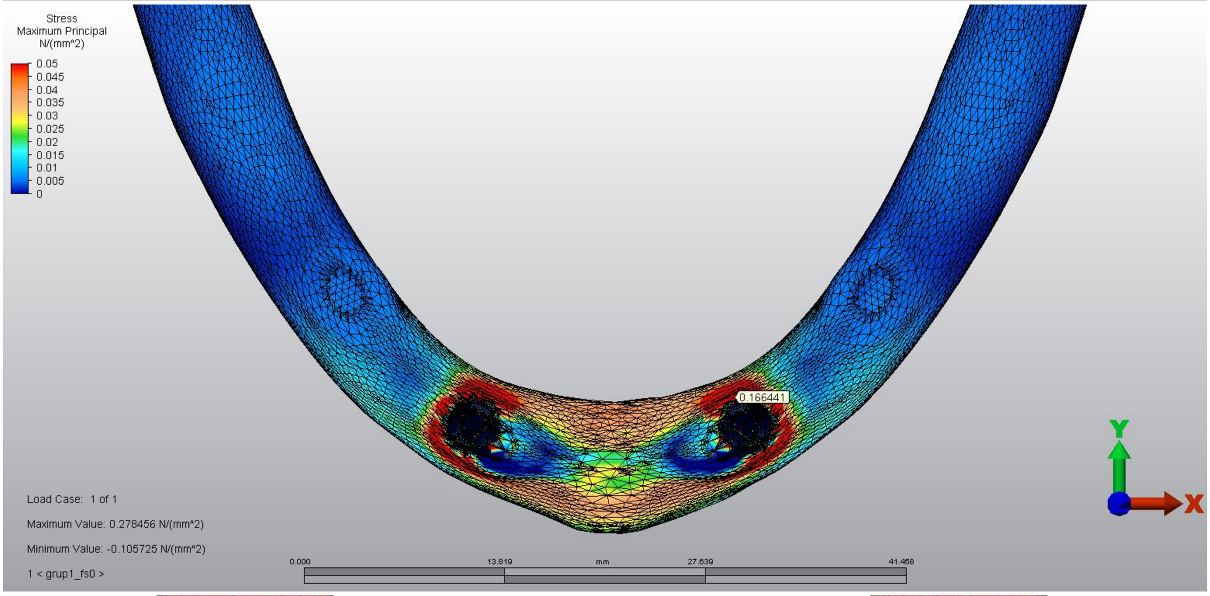
Stress
Maximum Principal
N/(mm^2)
0.05
0.045
0.04
0.035
0.03
0.025
0.02
0.015
0.01
0.005
0
0.166441
Load Case: 1 of 1
Maximum Value: 0.278456 N/(mm^2)
Minimum Value: -0.105725 N/(mm^2)
1 < grup1_fs0 >
0.000
13.819
mm
27.639
41.458
Y
X

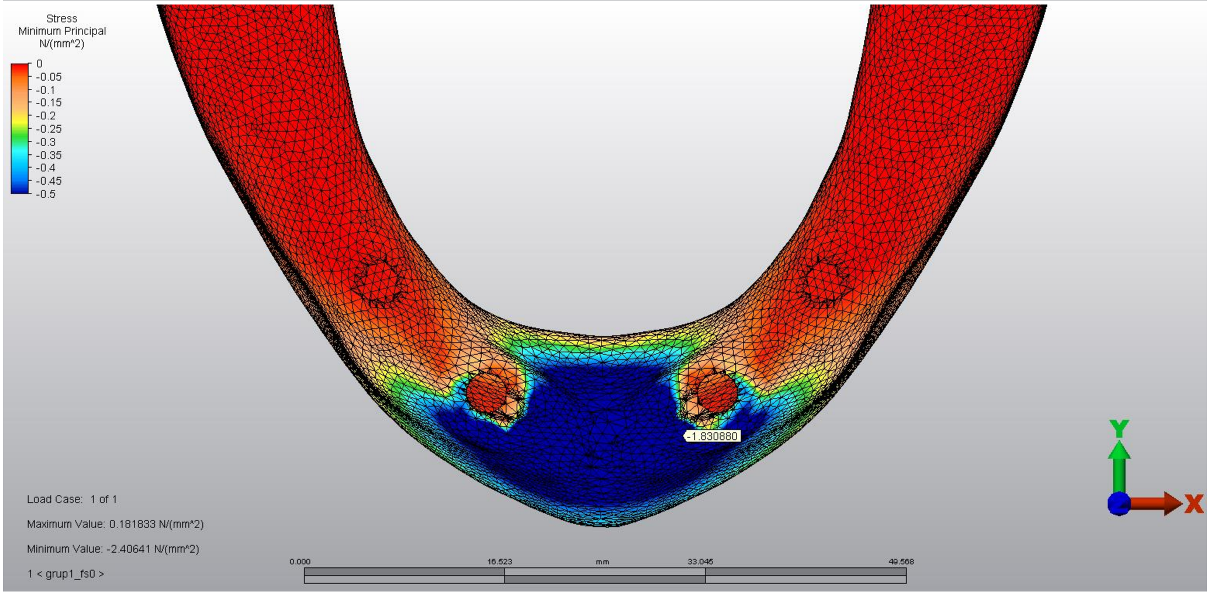
Stress
Minimum Principal
N/(mm^2)
0
-0.05
-0.1
-0.15
-0.2
-0.25
-0.3
-0.35
-0.4
-0.45
-0.5
-1.830880
Load Case: 1 of 1
Maximum Value: 0.181833 N/(mm^2)
Minimum Value: -2.40641 N/(mm^2)
1 < grup1_fs0 >
0.000
16.523
mm
33.045
49.568
Y
X

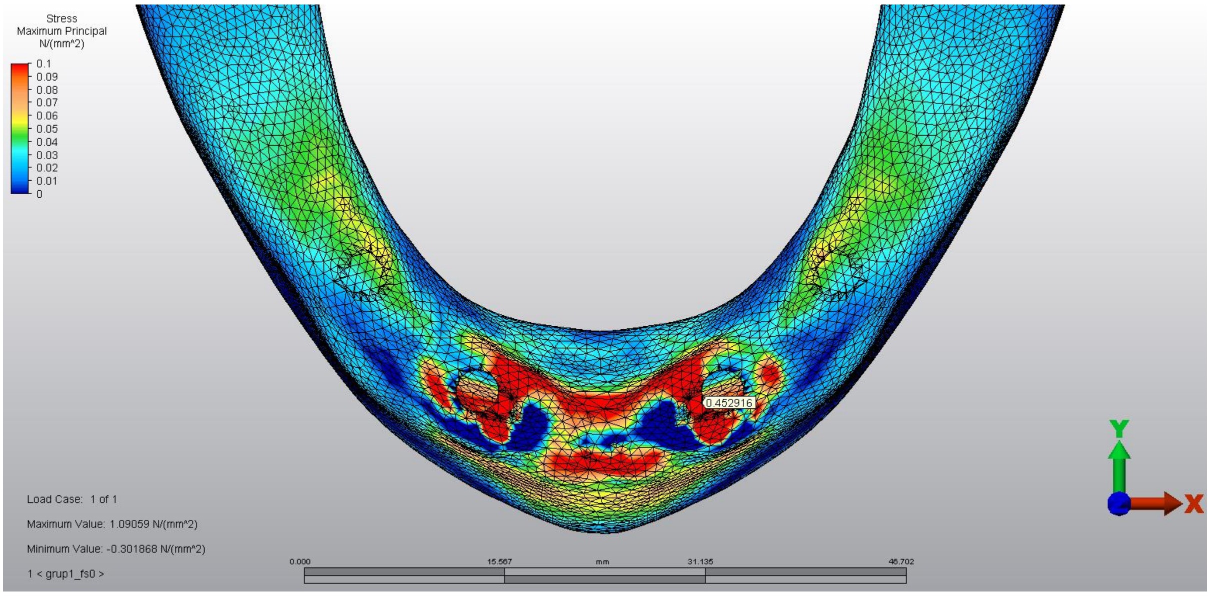
Stress
Maximum Principal
N/(mm^2)
0.1
0.09
0.08
0.07
0.06
0.05
0.04
0.03
0.02
0.01
0
0.452916
Load Case: 1 of 1
Maximum Value: 1.09059 N/(mm^2)
Minimum Value: -0.301868 N/(mm^2)
1 < grup1_fs0 >
0.000
15.567
mm
31.135
46.702
Y
X

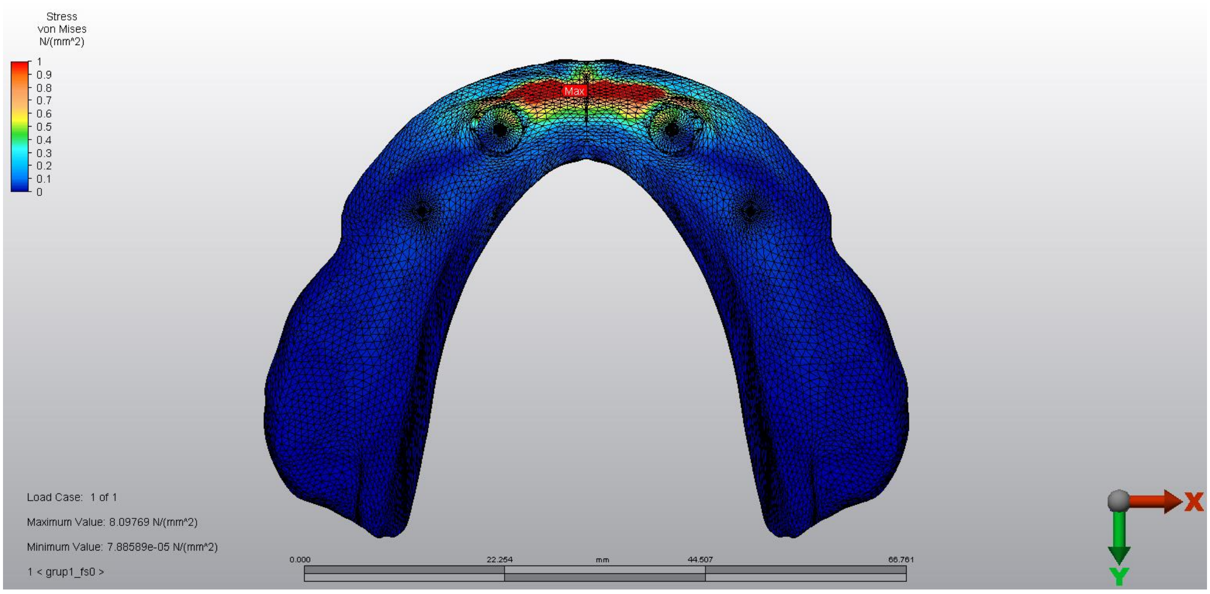
Stress
von Mises
N/(mm^2)
1
0.9
0.8
0.7
0.6
0.5
0.4
0.3
0.2
0.1
0
Max
Load Case: 1 of 1
Maximum Value: 8.09769 N/(mm^2)
Minimum Value: 7.88589e-05 N/(mm^2)
1 < grup1_fs0 >
0.000
22.254
mm
44.507
66.761
X
Y

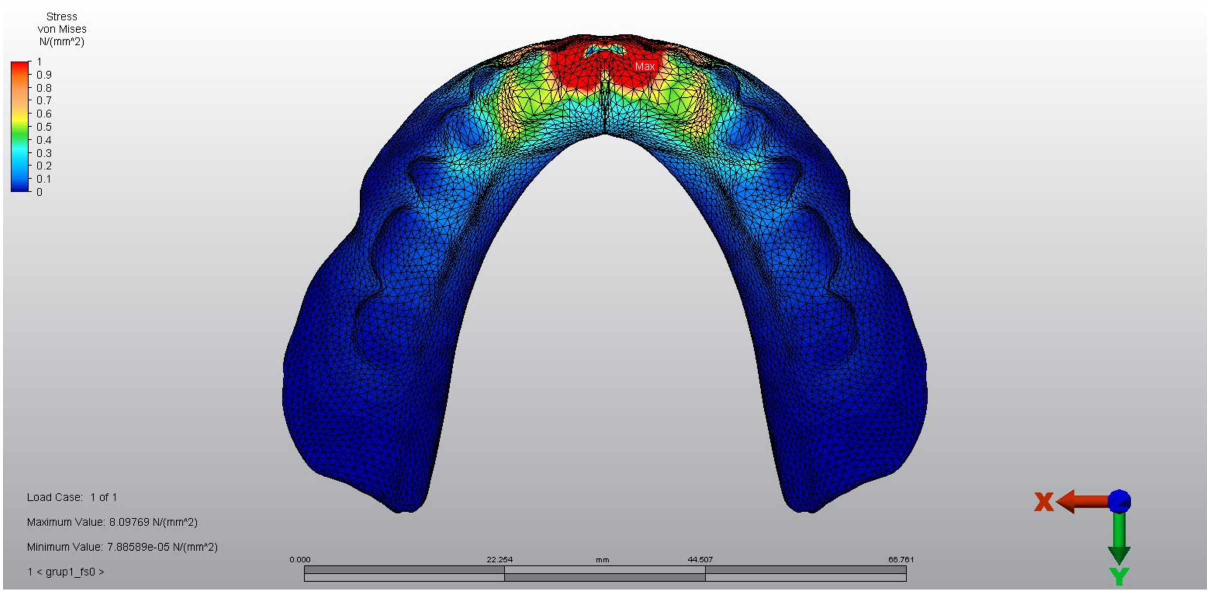
Stress
von Mises
N/(mm^2)
1
0.9
0.8
0.7
0.6
0.5
0.4
0.3
0.2
0.1
0
Max
Load Case: 1 of 1
Maximum Value: 8.09769 N/(mm^2)
Minimum Value: 7.88589e-05 N/(mm^2)
1 < grup1_fs0 >
0.000
22.254
mm
44.507
66.761
X
Y

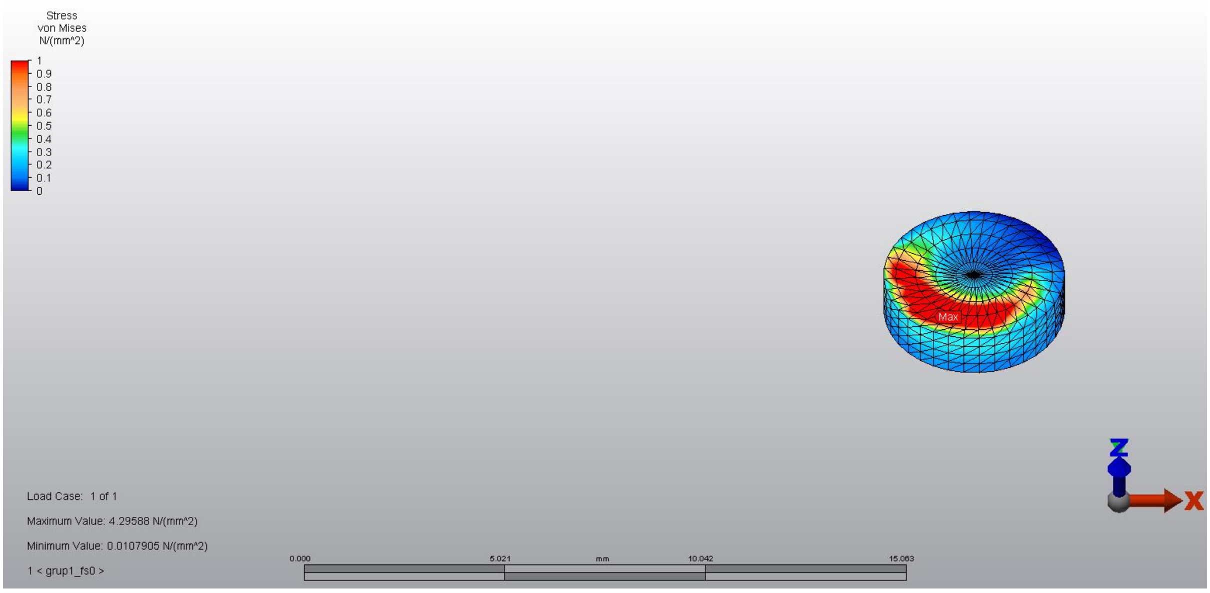
Stress
von Mises
N/(mm^2)
1
0.9
0.8
0.7
0.6
0.5
0.4
0.3
0.2
0.1
0
Max
Load Case: 1 of 1
Maximum Value: 4.29588 N/(mm^2)
Minimum Value: 0.0107905 N/(mm^2)
1 < grup1_fs0 >
0.000
5.021
mm
10.042
15.063
Z
X

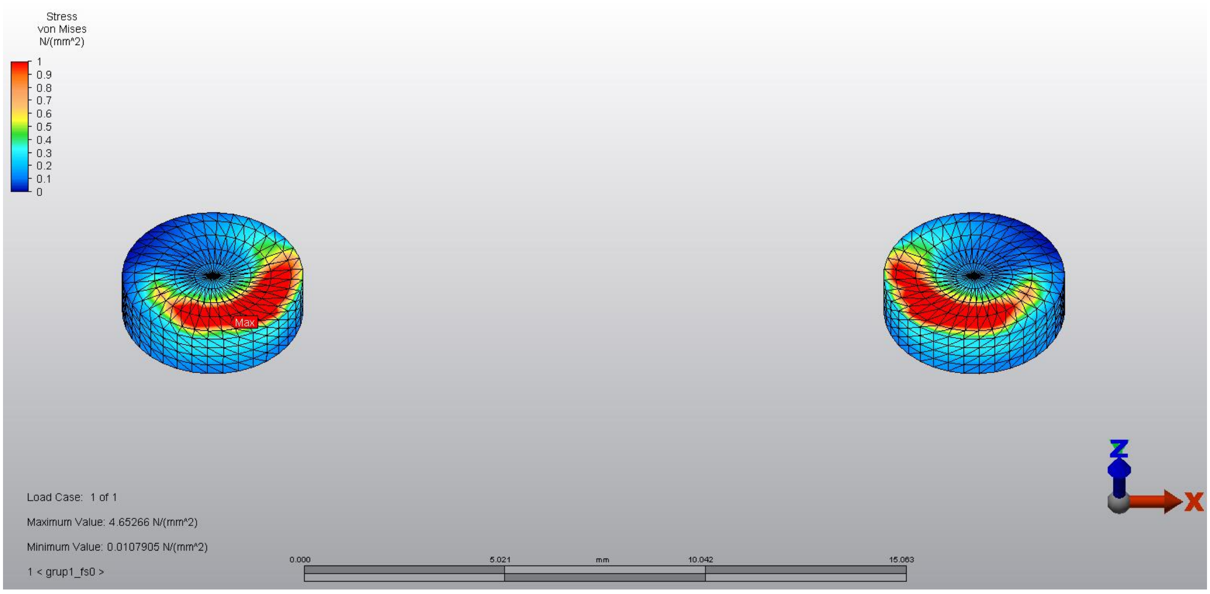
Stress
von Mises
N/(mm^2)
1
0.9
0.8
0.7
0.6
0.5
0.4
0.3
0.2
0.1
0
Max
Load Case: 1 of 1
Maximum Value: 4.65266 N/(mm^2)
Minimum Value: 0.0107905 N/(mm^2)
1 < grup1_fs0 >
0.000
5.021
mm
10.042
15.063
Z
X

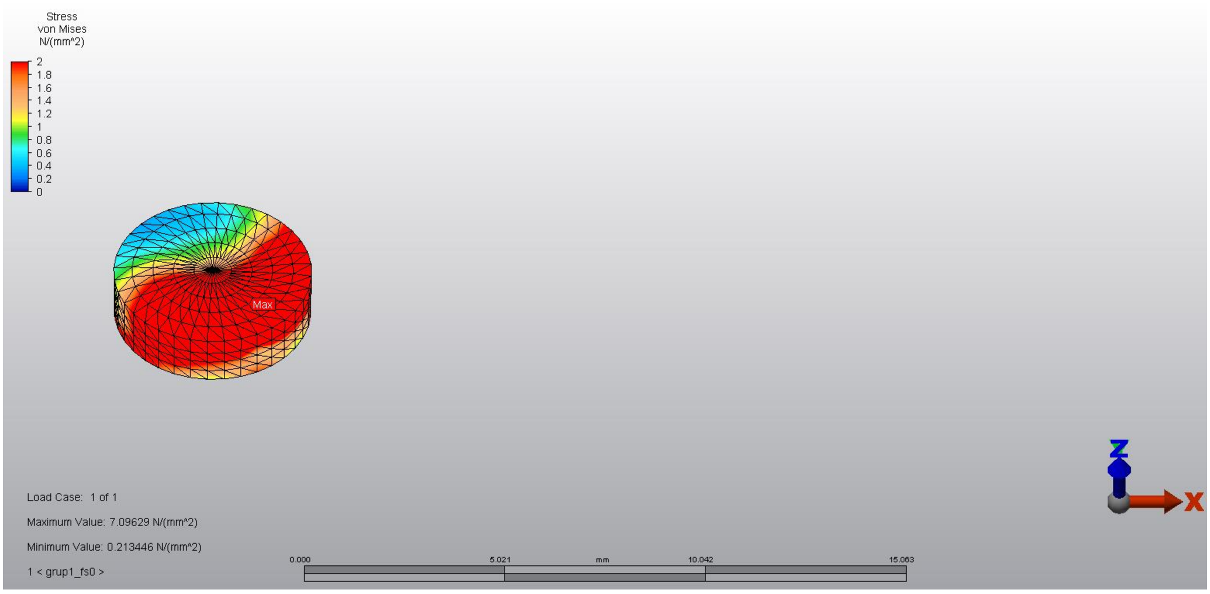
Stress
von Mises
N/(mm^2)
2
1.8
1.6
1.4
1.2
1
0.8
0.6
0.4
0.2
0
Max
Load Case: 1 of 1
Maximum Value: 7.09629 N/(mm^2)
Minimum Value: 0.213446 N/(mm^2)
1 < grup1_fs0 >
0.000
5.021
mm
10.042
15.063
Z
X

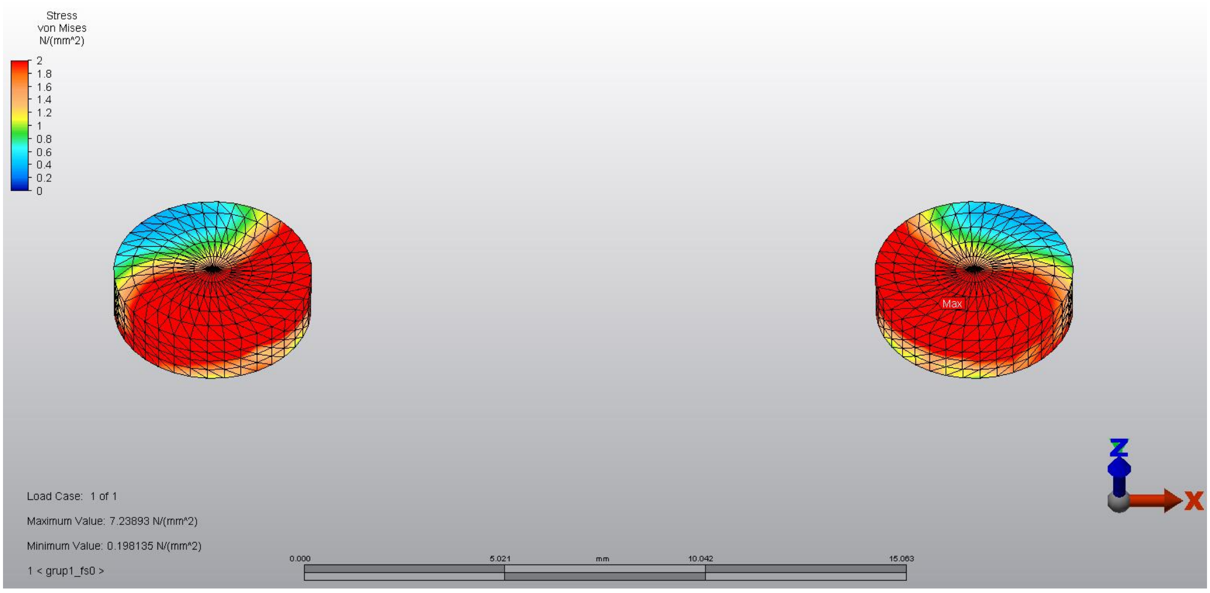
Stress
von Mises
N/(mm^2)
2
1.8
1.6
1.4
1.2
1
0.8
0.6
0.4
0.2
0
Max
Load Case: 1 of 1
Maximum Value: 7.23893 N/(mm^2)
Minimum Value: 0.198135 N/(mm^2)
1 < grup1_fs0 >
0.000
5.021
mm
10.042
15.063
Z
X

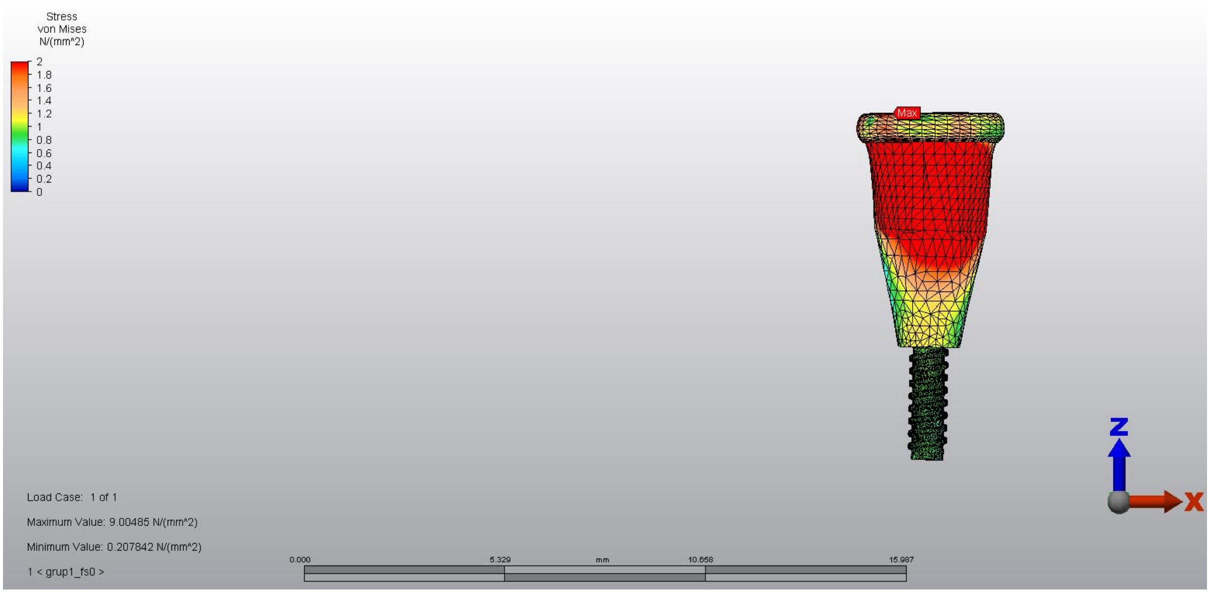
Stress
von Mises
N/(mm^2)
2
1.8
1.6
1.4
1.2
1
0.8
0.6
0.4
0.2
0
Max
Load Case: 1 of 1
Maximum Value: 9.00485 N/(mm^2)
Minimum Value: 0.207842 N/(mm^2)
1 < grup1_fs0 >
0.000
5.329
mm
10.658
15.987
Z
X

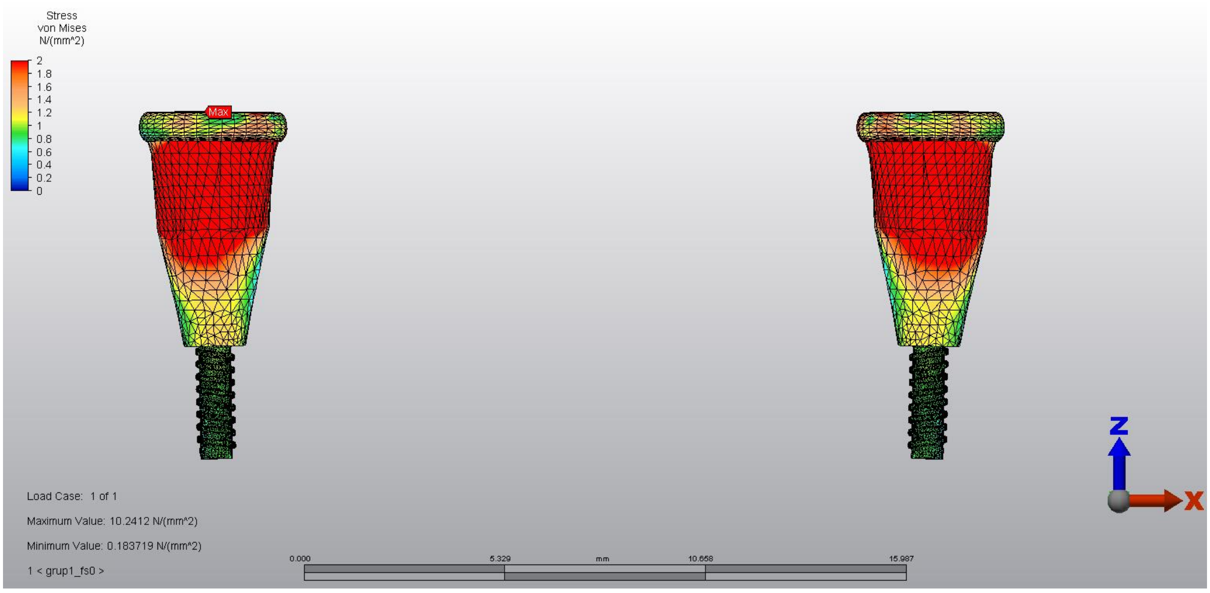

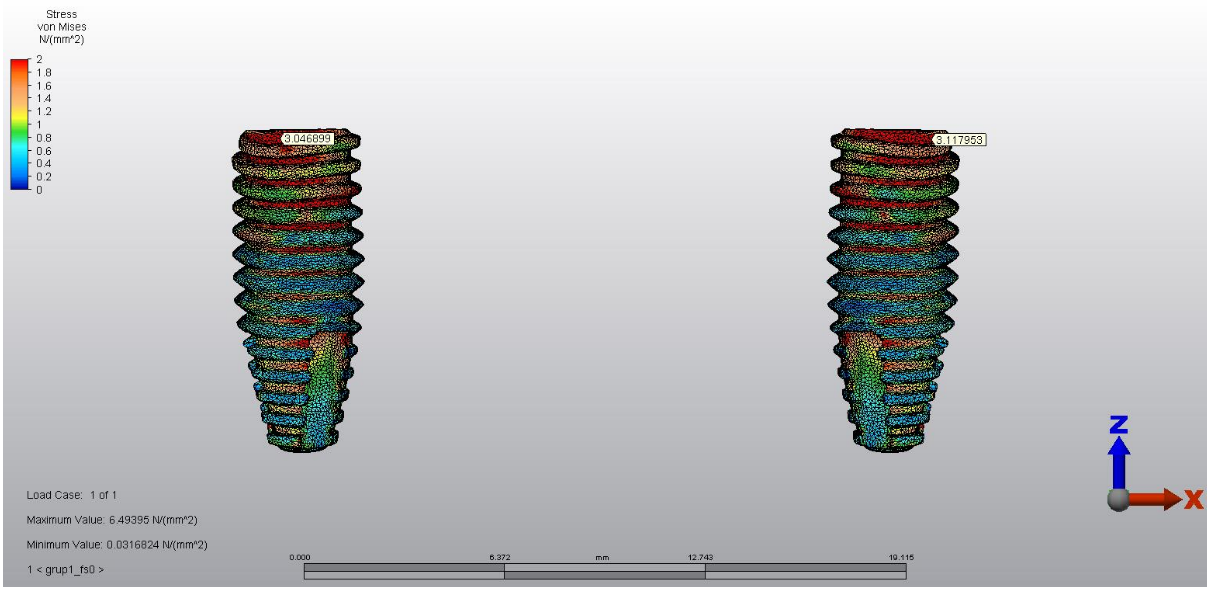

UNILATERAL

Number of nodes = 135767

Number of elements = 713569

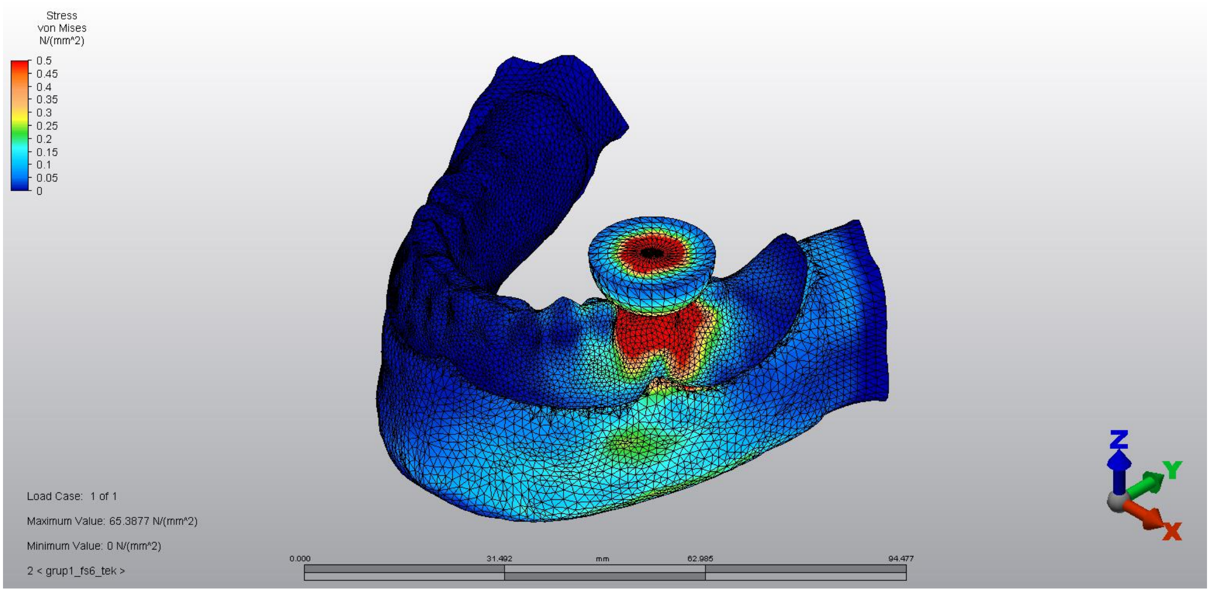
Stress
von Mises
N/(mm^2)
0.5
0.45
0.4
0.35
0.3
0.25
0.2
0.15
0.1
0.05
0
Load Case: 1 of 1
Maximum Value: 65.3877 N/(mm^2)
Minimum Value: 0 N/(mm^2)
2 < grup1_fs6_tek >
0.000
31.492
mm
62.985
94.477
Z
Y
X

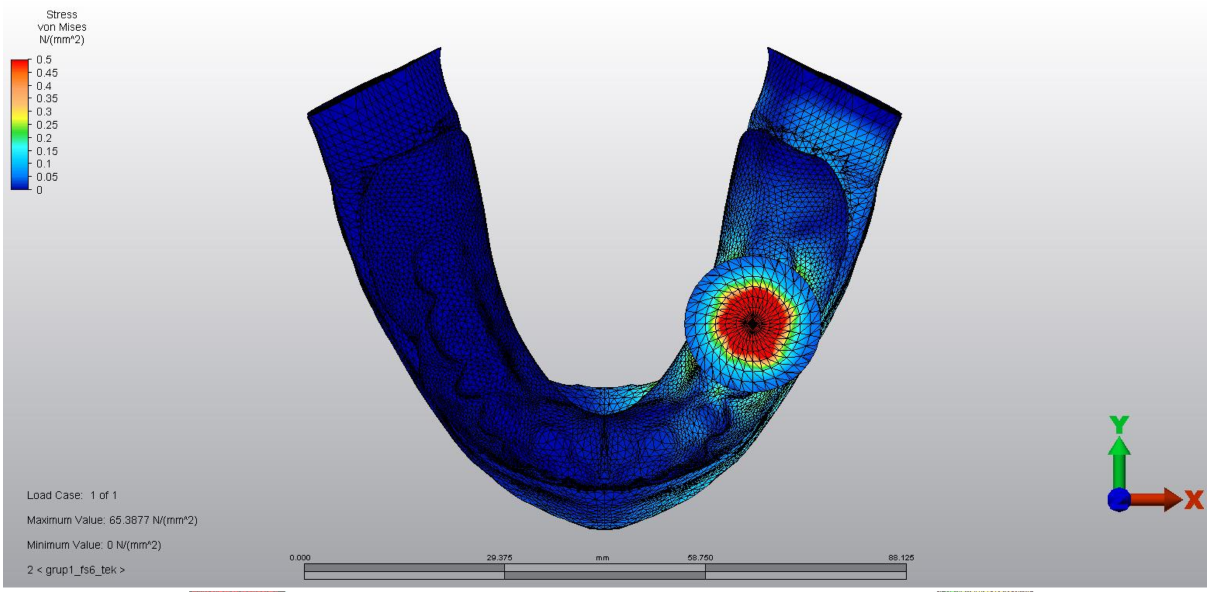
Stress
von Mises
N/(mm^2)
0.5
0.45
0.4
0.35
0.3
0.25
0.2
0.15
0.1
0.05
0
Load Case: 1 of 1
Maximum Value: 65.3877 N/(mm^2)
Minimum Value: 0 N/(mm^2)
2 < grup1_fs6_tek >
0.000
29.375
mm
58.750
88.125
Y
X

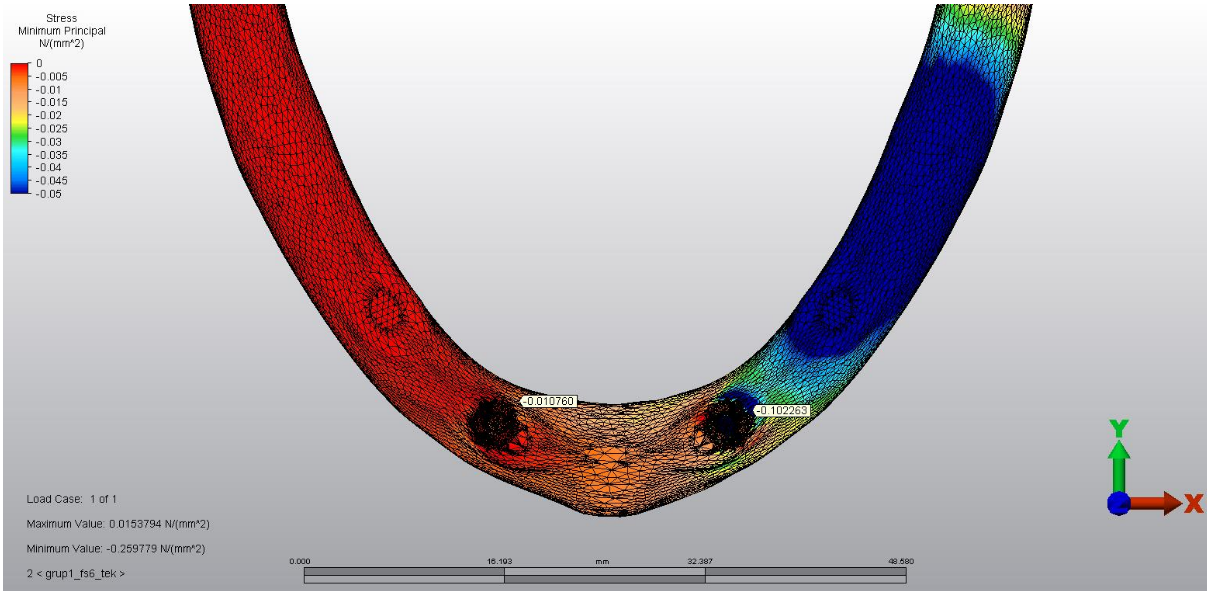
Stress
Minimum Principal
N/(mm^2)
0
-0.005
-0.01
-0.015
-0.02
-0.025
-0.03
-0.035
-0.04
-0.045
-0.05
-0.010760
-0.102263
Load Case: 1 of 1
Maximum Value: 0.0153794 N/(mm^2)
Minimum Value: -0.259779 N/(mm^2)
2 < grup1_fs6_tek >
0.000
16.193
mm
32.387
48.580
Y
X

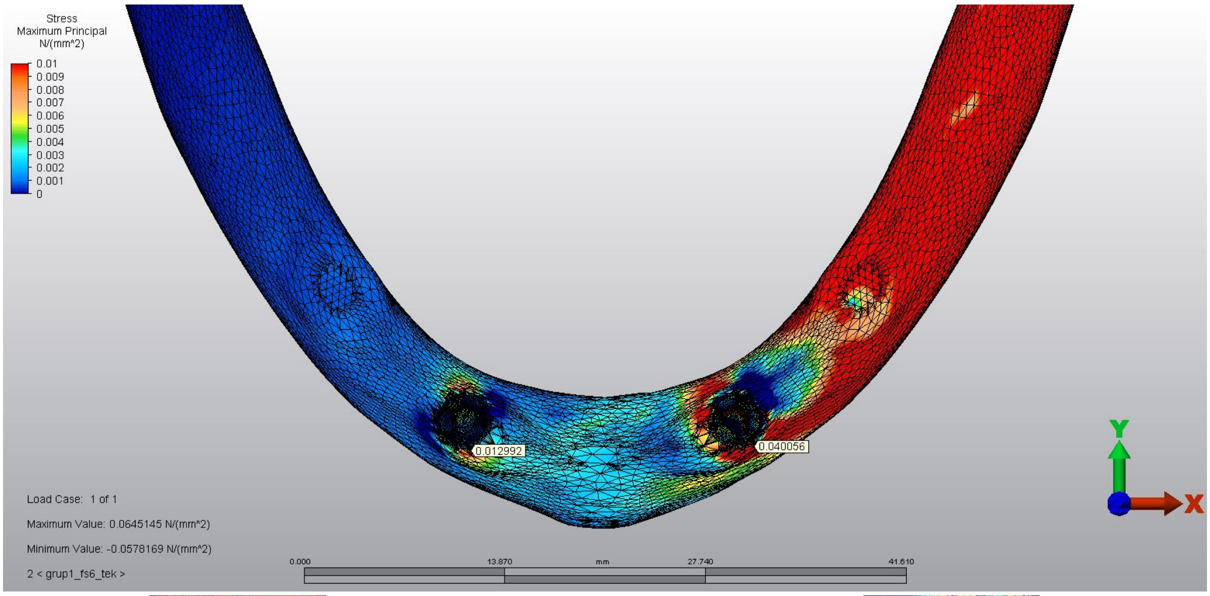
Stress
Maximum Principal
N/(mm^2)
0.01
0.009
0.008
0.007
0.006
0.005
0.004
0.003
0.002
0.001
0
0.012992
0.040056
Load Case: 1 of 1
Maximum Value: 0.0645145 N/(mm^2)
Minimum Value: -0.0578169 N/(mm^2)
2 < grup1_fs6_tek >
0.000
13.870
mm
27.740
41.610
Y
X

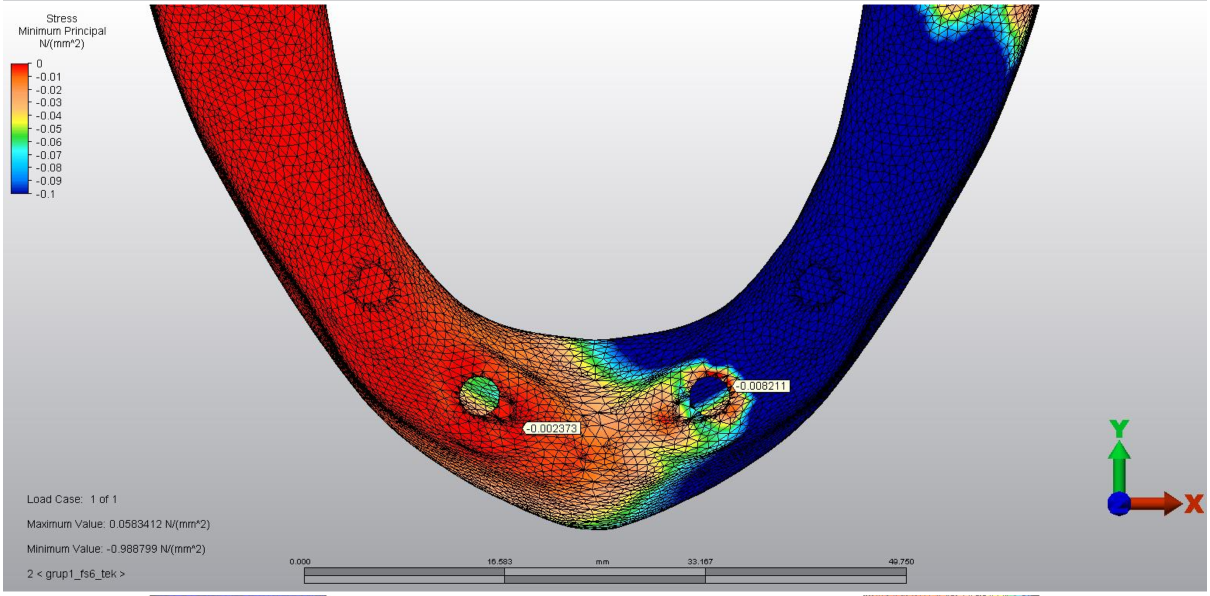
Stress
Minimum Principal
N/(mm^2)
0
-0.01
-0.02
-0.03
-0.04
-0.05
-0.06
-0.07
-0.08
-0.09
-0.1
-0.008211
-0.002373
Load Case: 1 of 1
Maximum Value: 0.0583412 N/(mm^2)
Minimum Value: -0.988799 N/(mm^2)
2 < grup1_fs6_tek >
0.000
16.583
mm
33.167
49.750
Y
X

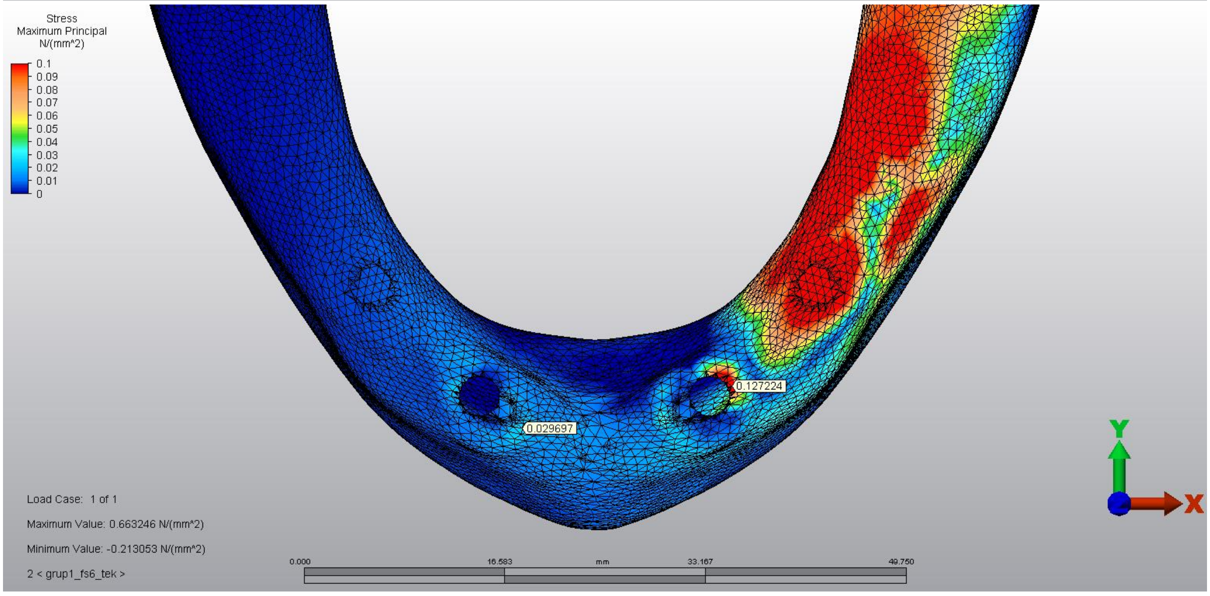
Stress
Maximum Principal
N/(mm^2)
0.1
0.09
0.08
0.07
0.06
0.05
0.04
0.03
0.02
0.01
0
0.127224
0.029697
Load Case: 1 of 1
Maximum Value: 0.663246 N/(mm^2)
Minimum Value: -0.213053 N/(mm^2)
2 < grup1_fs6_tek >
0.000
16.583
mm
33.167
49.750
Y
X

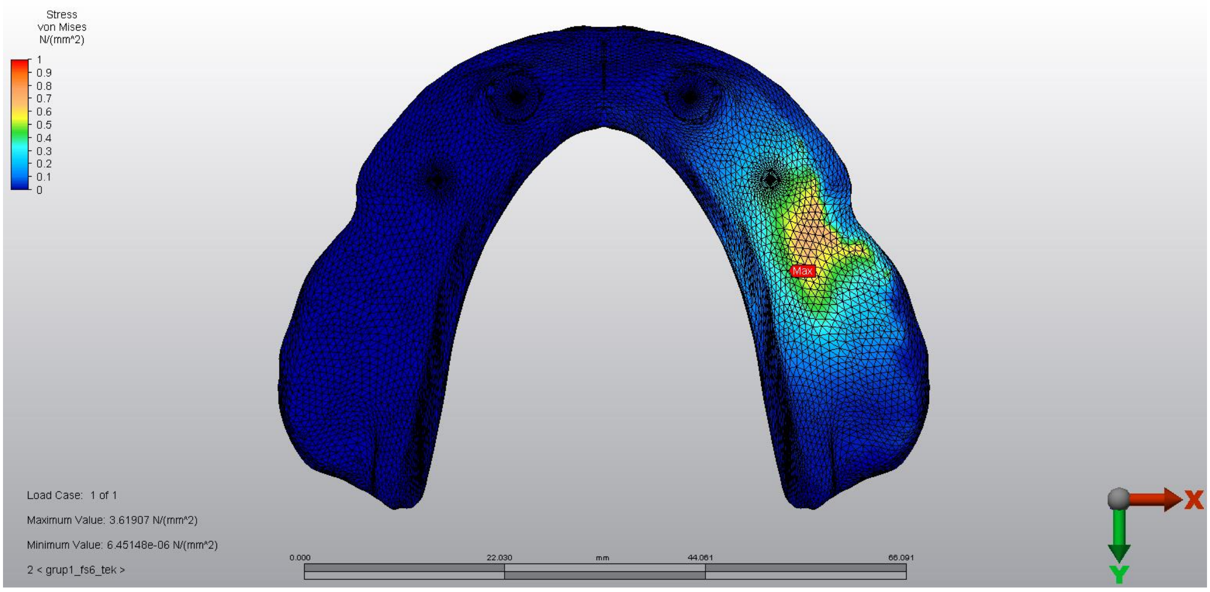
Stress
von Mises
N/(mm^2)
1
0.9
0.8
0.7
0.6
0.5
0.4
0.3
0.2
0.1
0
Max
Load Case: 1 of 1
Maximum Value: 3.61907 N/(mm^2)
Minimum Value: 6.45148e-06 N/(mm^2)
2 < grup1_fs6_tek >
0.000
22.030
mm
44.061
66.091
X
Y

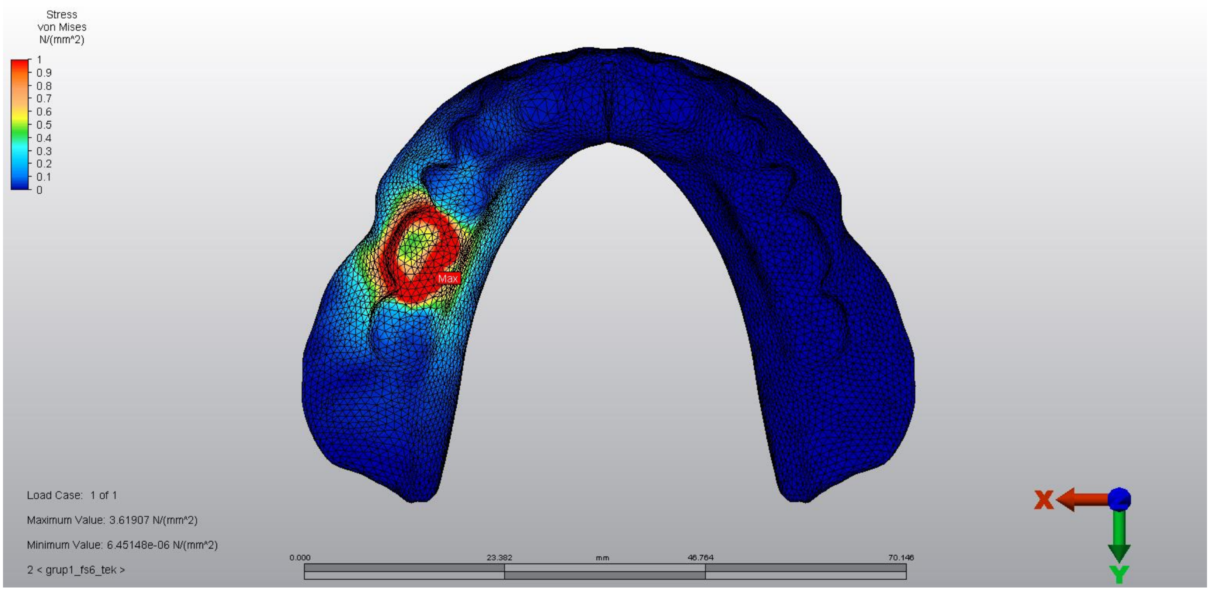
Stress
von Mises
N/(mm^2)
1
0.9
0.8
0.7
0.6
0.5
0.4
0.3
0.2
0.1
0
Max
Load Case: 1 of 1
Maximum Value: 3.61907 N/(mm^2)
Minimum Value: 6.45148e-06 N/(mm^2)
2 < grup1_fs6_tek >
0.000
23.382
mm
46.764
70.146
X
Y

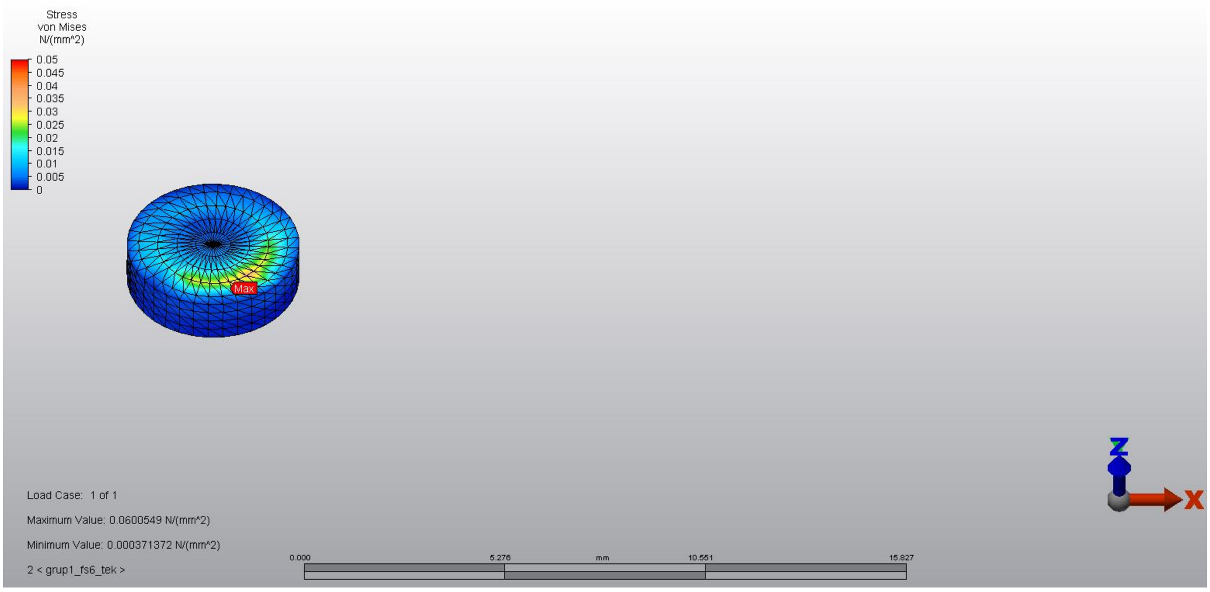
Stress
von Mises
N/(mm^2)
0.05
0.045
0.04
0.035
0.03
0.025
0.02
0.015
0.01
0.005
0
Max
Load Case: 1 of 1
Maximum Value: 0.0600549 N/(mm^2)
Minimum Value: 0.000371372 N/(mm^2)
2 < grup1_fs6_tek >
0.000
5.276
mm
10.551
15.827
Z
X

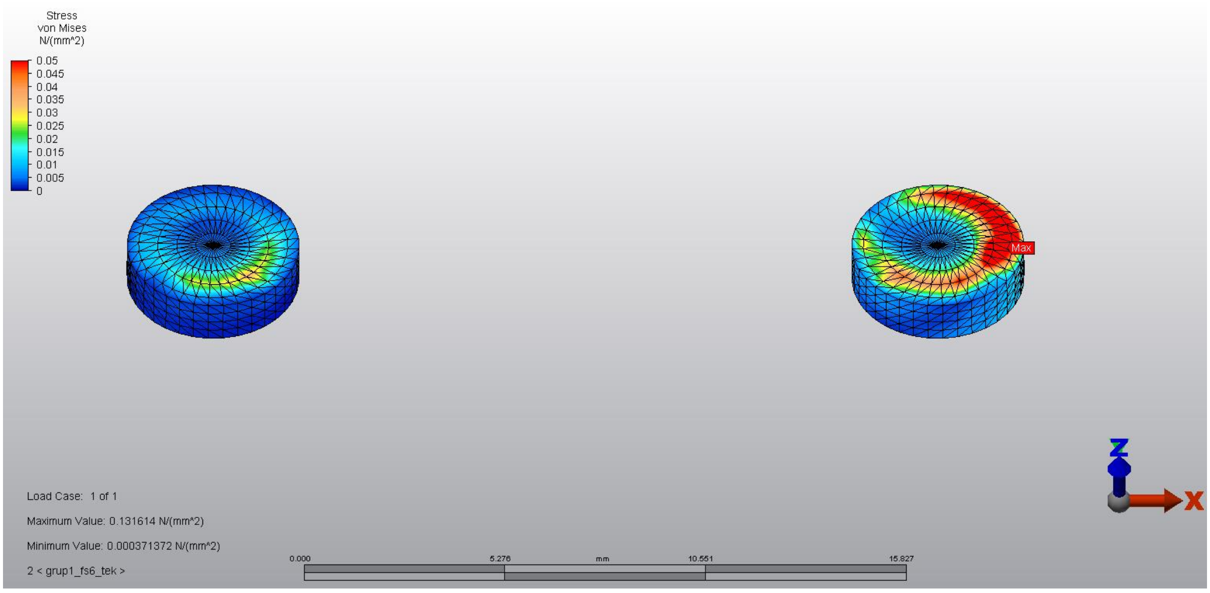
Stress
von Mises
N/(mm^2)
0.05
0.045
0.04
0.035
0.03
0.025
0.02
0.015
0.01
0.005
0
Max
Z
X
Load Case: 1 of 1
Maximum Value: 0.131614 N/(mm^2)
Minimum Value: 0.000371372 N/(mm^2)
2 < grup1_fs6_tek >
0.000
5.276
mm
10.551
15.827

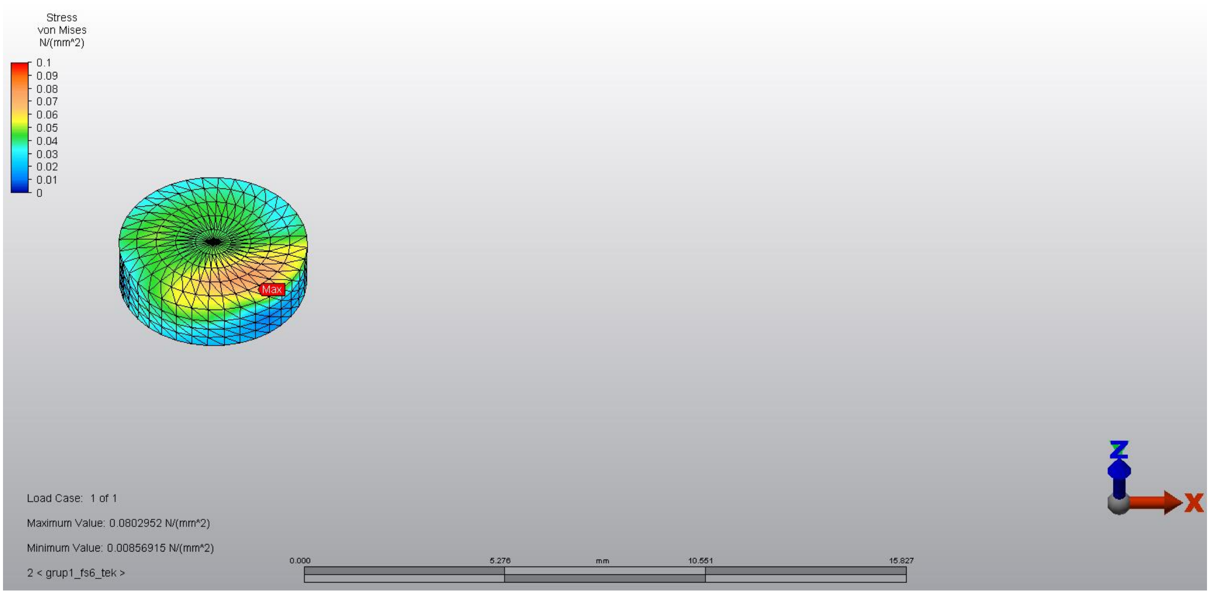
Stress
von Mises
N/(mm^2)
0.1
0.09
0.08
0.07
0.06
0.05
0.04
0.03
0.02
0.01
0
Max
Z
X
Load Case: 1 of 1
Maximum Value: 0.0802952 N/(mm^2)
Minimum Value: 0.00856915 N/(mm^2)
2 < grup1_fs6_tek >
0.000
5.276
mm
10.551
15.827

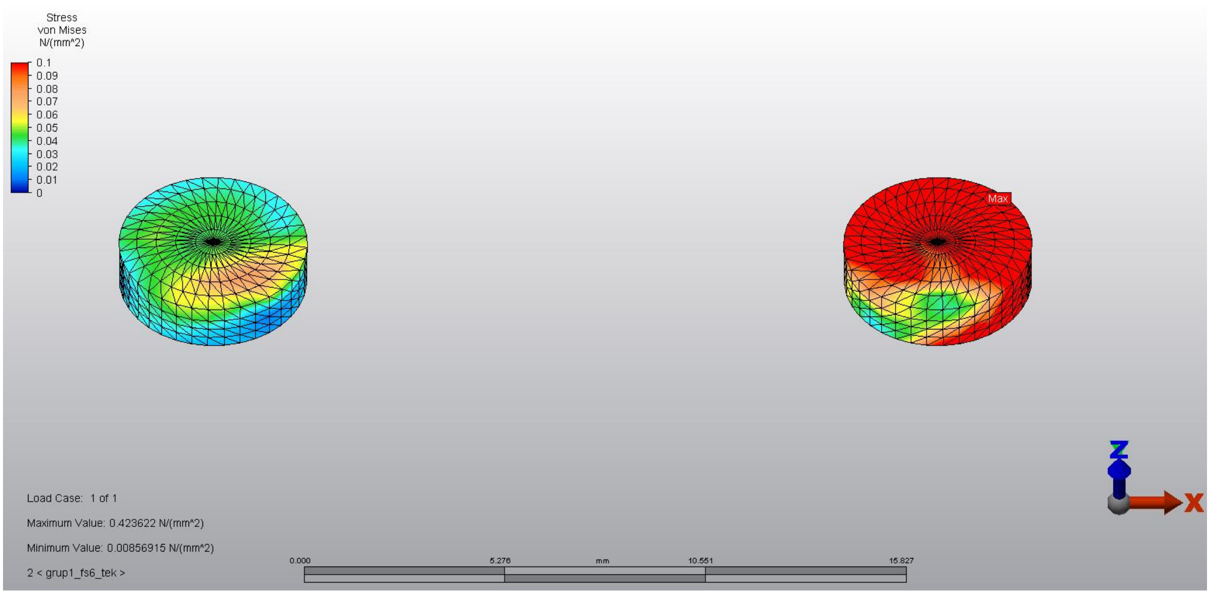
Stress
von Mises
N/(mm^2)
0.1
0.09
0.08
0.07
0.06
0.05
0.04
0.03
0.02
0.01
0
Max
Z
X
Load Case: 1 of 1
Maximum Value: 0.423622 N/(mm^2)
Minimum Value: 0.00856915 N/(mm^2)
2 < grup1_fs6_tek >
0.000
5.276
mm
10.551
15.827

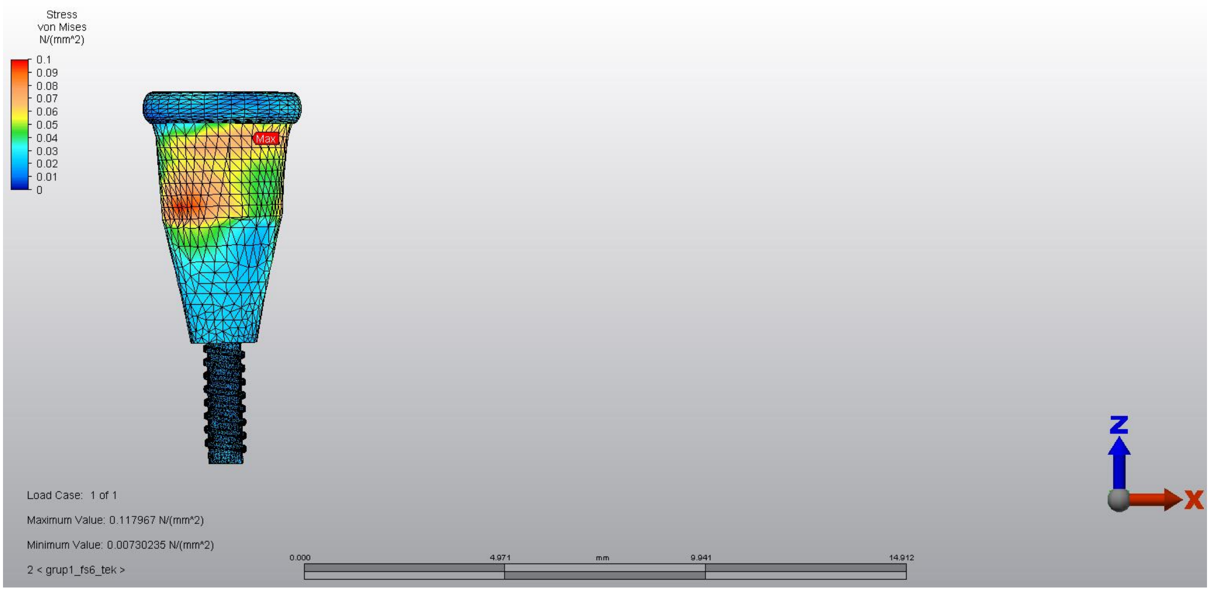
Stress
von Mises
N/(mm^2)
0.1
0.09
0.08
0.07
0.06
0.05
0.04
0.03
0.02
0.01
0
Max
Z
X
Load Case: 1 of 1
Maximum Value: 0.117967 N/(mm^2)
Minimum Value: 0.00730235 N/(mm^2)
2 < grup1_fs6_tek >
0.000
4.971
mm
9.941
14.912

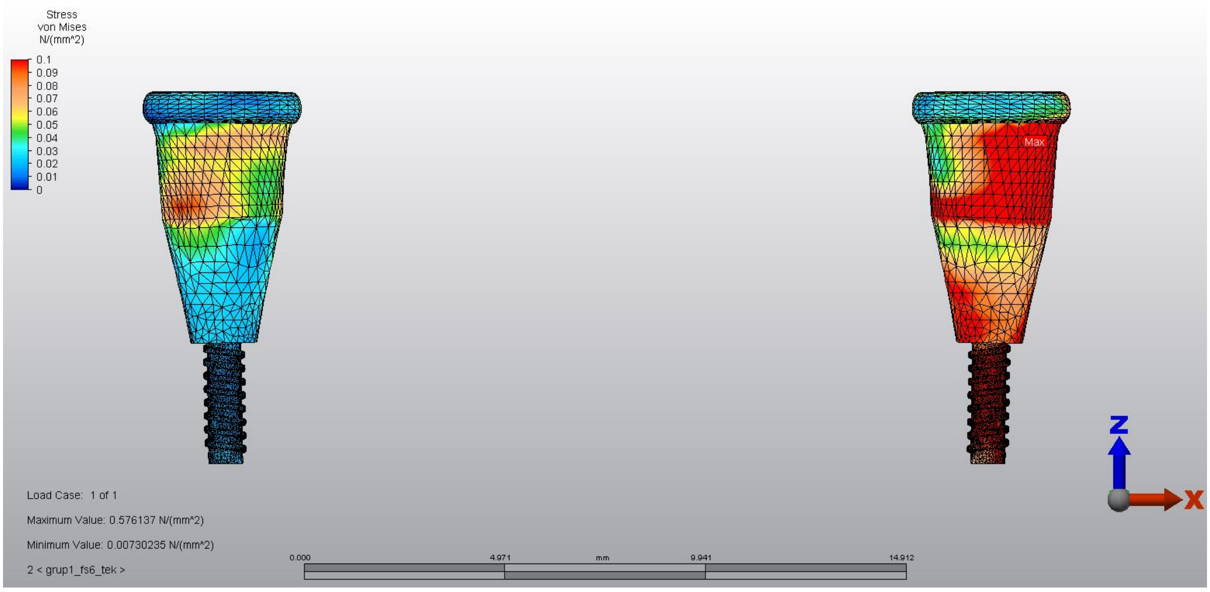
Stress
von Mises
N/(mm^2)
0.1
0.09
0.08
0.07
0.06
0.05
0.04
0.03
0.02
0.01
0
Max
Z
X
Load Case: 1 of 1
Maximum Value: 0.576137 N/(mm^2)
Minimum Value: 0.00730235 N/(mm^2)
2 < grup1_fs6_tek >
0.000
4.971
mm
9.941
14.912

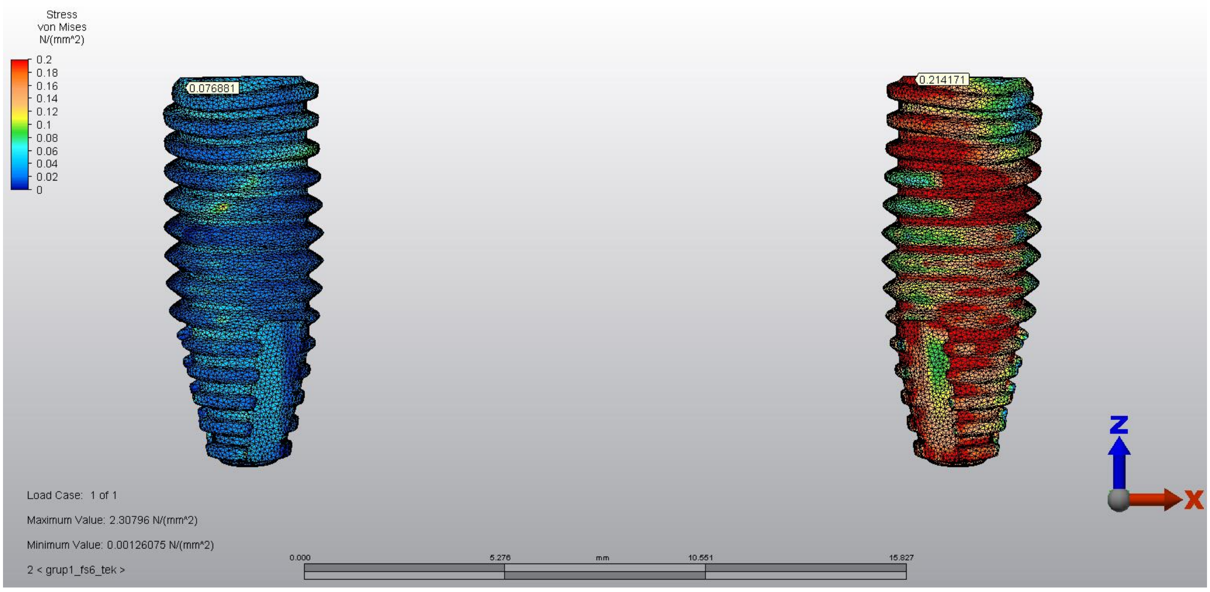
Stress
von Mises
N/(mm^2)
0.2
0.18
0.16
0.14
0.12
0.1
0.08
0.06
0.04
0.02
0
0.076881
0.214171
Z
X
Load Case: 1 of 1
Maximum Value: 2.30796 N/(mm^2)
Minimum Value: 0.00126075 N/(mm^2)
2 < grup1_fs6_tek >
0.000
5.276
mm
10.551
15.827

BILATERAL

Number of nodes = 136479

Number of elements = 716117

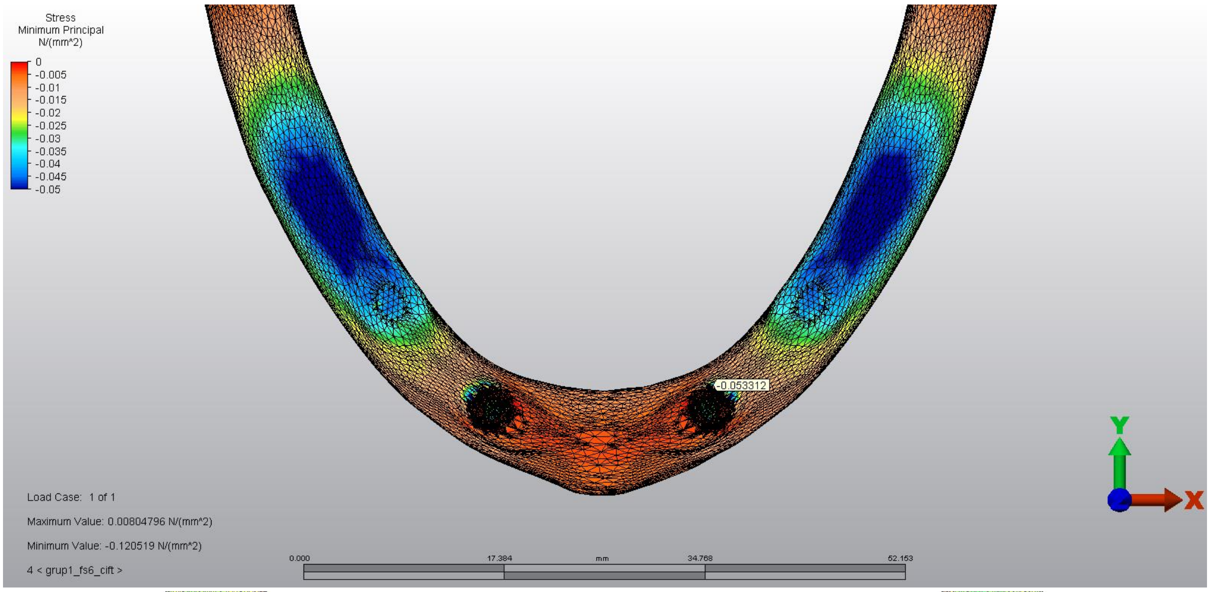

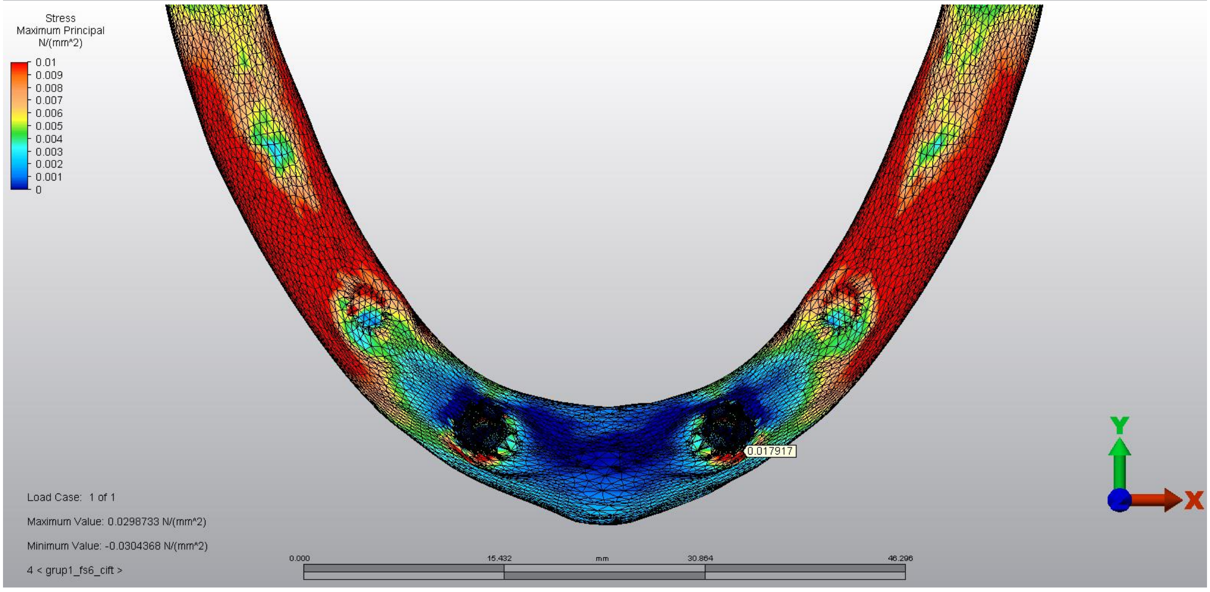

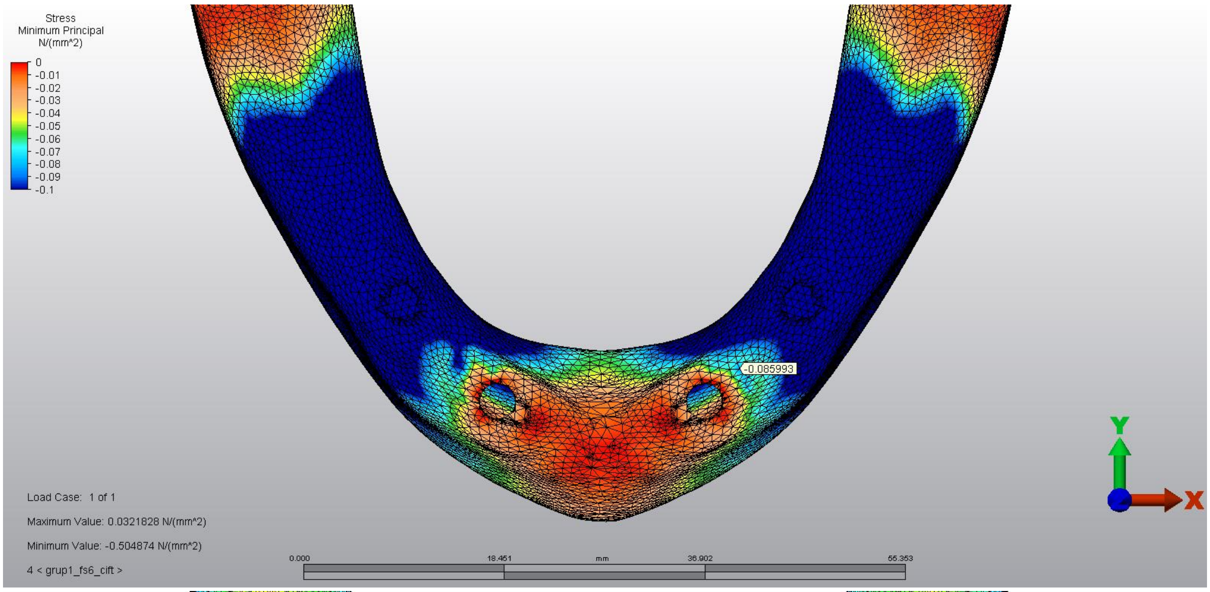
Stress
Minimum Principal
N/(mm^2)
0
-0.01
-0.02
-0.03
-0.04
-0.05
-0.06
-0.07
-0.08
-0.09
-0.1
-0.085993
Load Case: 1 of 1
Maximum Value: 0.0321828 N/(mm^2)
Minimum Value: -0.504874 N/(mm^2)
4 < grup1_fs6_cift >
0.000
18.451
mm
36.902
55.353
Y
X

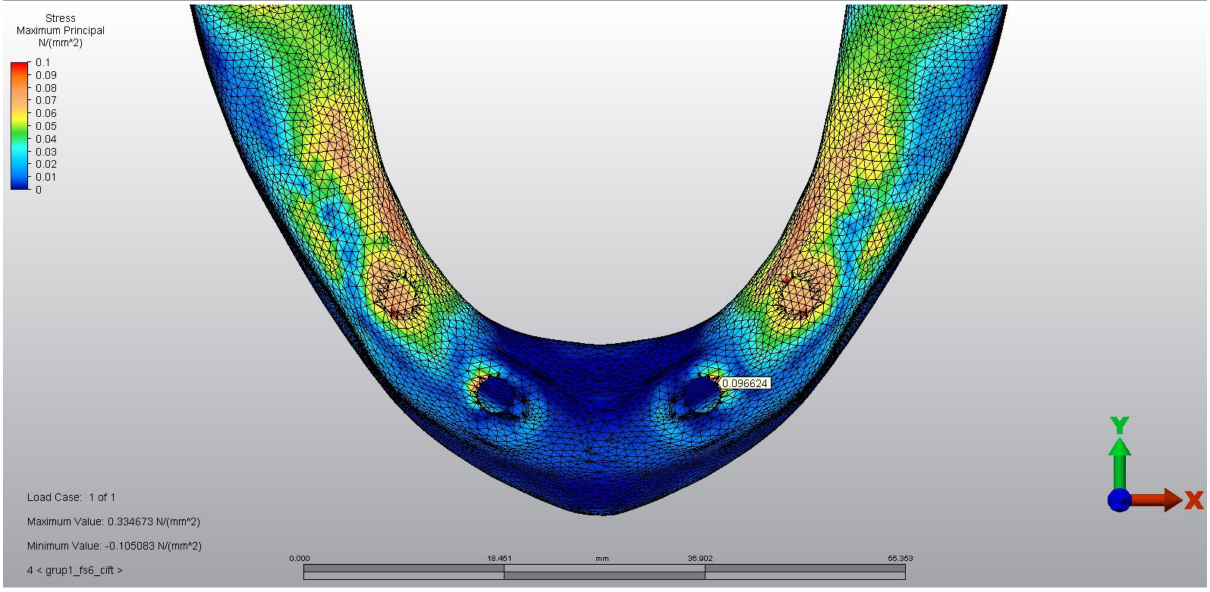
Stress
Maximum Principal
N/(mm^2)
0.1
0.09
0.08
0.07
0.06
0.05
0.04
0.03
0.02
0.01
0
0.096624
Load Case: 1 of 1
Maximum Value: 0.334673 N/(mm^2)
Minimum Value: -0.105083 N/(mm^2)
4 < grup1_fs6_cift >
0.000
18.451
mm
36.902
55.353
Y
X

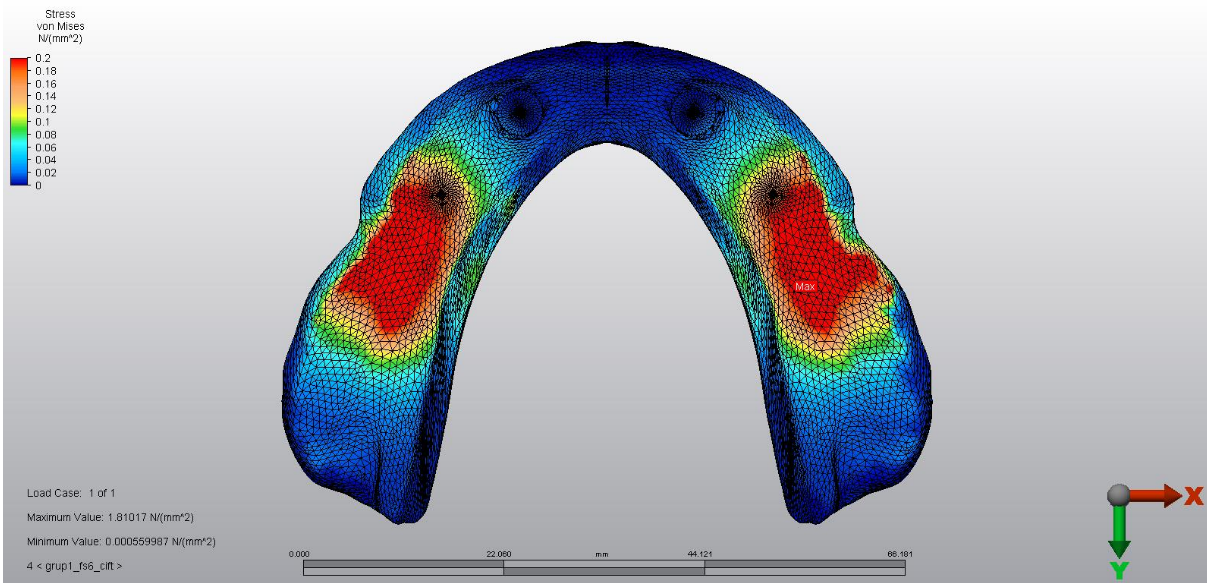
Stress
von Mises
N/(mm^2)
0.2
0.18
0.16
0.14
0.12
0.1
0.08
0.06
0.04
0.02
0
Max
Load Case: 1 of 1
Maximum Value: 1.81017 N/(mm^2)
Minimum Value: 0.000559987 N/(mm^2)
4 < grup1_fs6_cift >
0.000
22.060
mm
44.121
66.181
X
Y

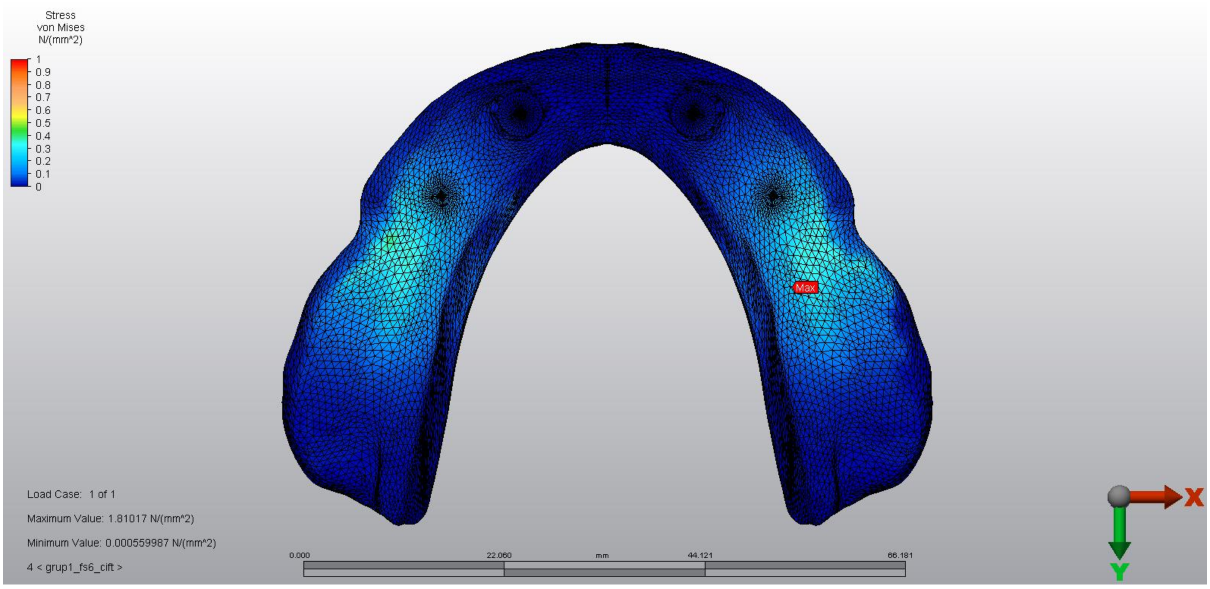
Stress
von Mises
N/(mm^2)
1
0.9
0.8
0.7
0.6
0.5
0.4
0.3
0.2
0.1
0
Max
Load Case: 1 of 1
Maximum Value: 1.81017 N/(mm^2)
Minimum Value: 0.000559987 N/(mm^2)
4 < grup1_fs6_cift >
0.000
22.060
mm
44.121
66.181
X
Y

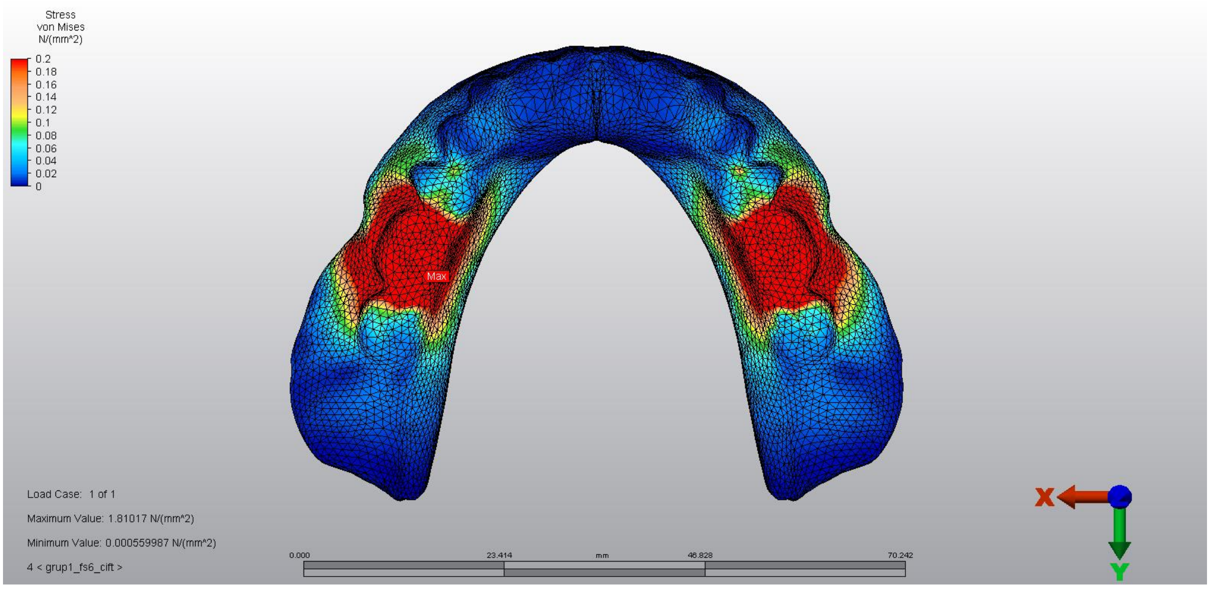
Stress
von Mises
N/(mm^2)
0.2
0.18
0.16
0.14
0.12
0.1
0.08
0.06
0.04
0.02
0
Max
Load Case: 1 of 1
Maximum Value: 1.81017 N/(mm^2)
Minimum Value: 0.000559987 N/(mm^2)
4 < grup1_fs6_cift >
0.000
23.414
mm
46.828
70.242
X
Y

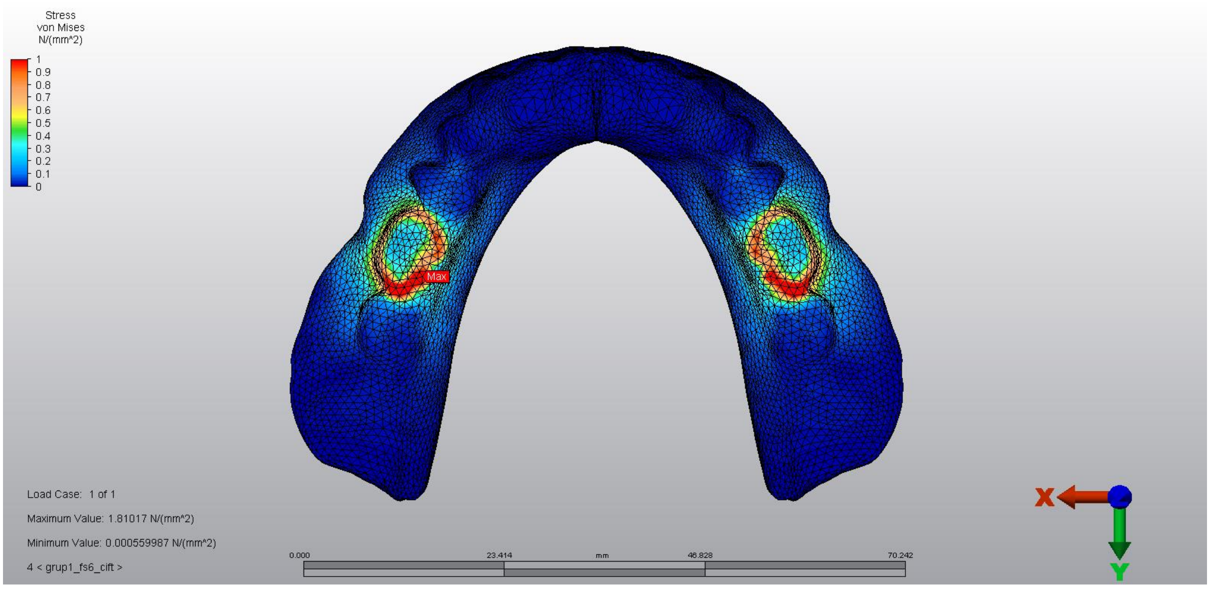
Stress
von Mises
N/(mm^2)
1
0.9
0.8
0.7
0.6
0.5
0.4
0.3
0.2
0.1
0
Max
Load Case: 1 of 1
Maximum Value: 1.81017 N/(mm^2)
Minimum Value: 0.000559987 N/(mm^2)
4 < grup1_fs6_cift >
0.000
23.414
mm
46.828
70.242
X
Y

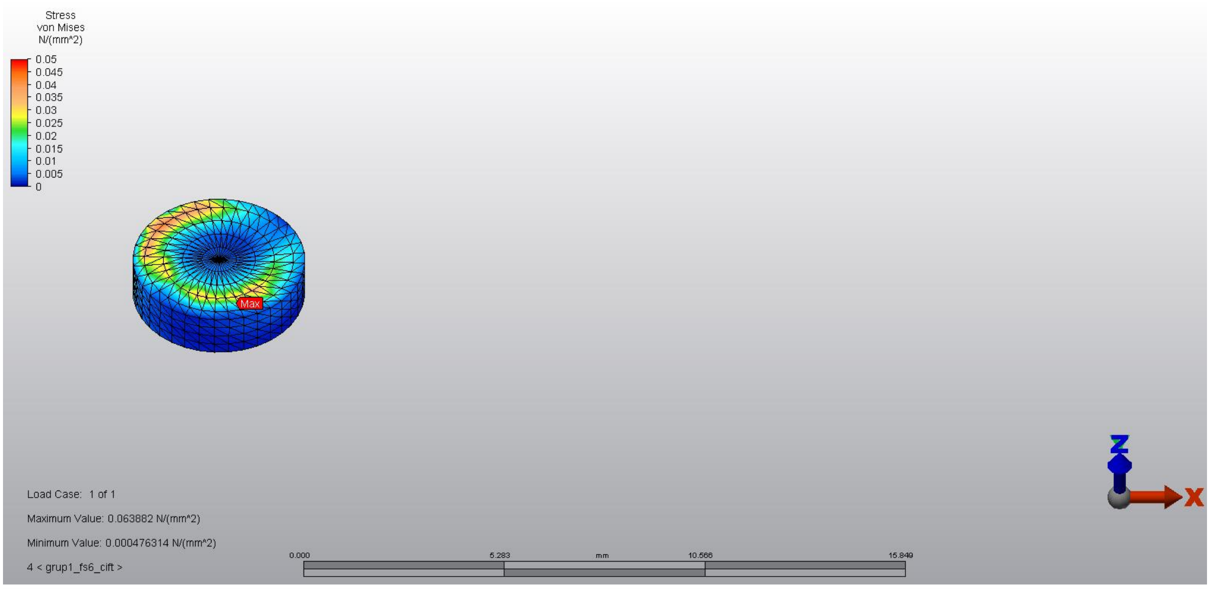
Stress
von Mises
N/(mm^2)
0.05
0.045
0.04
0.035
0.03
0.025
0.02
0.015
0.01
0.005
0
Max
Load Case: 1 of 1
Maximum Value: 0.063882 N/(mm^2)
Minimum Value: 0.000476314 N/(mm^2)
4 < grup1_fs6_cift >
0.000
5.283
mm
10.566
15.849
Z
X

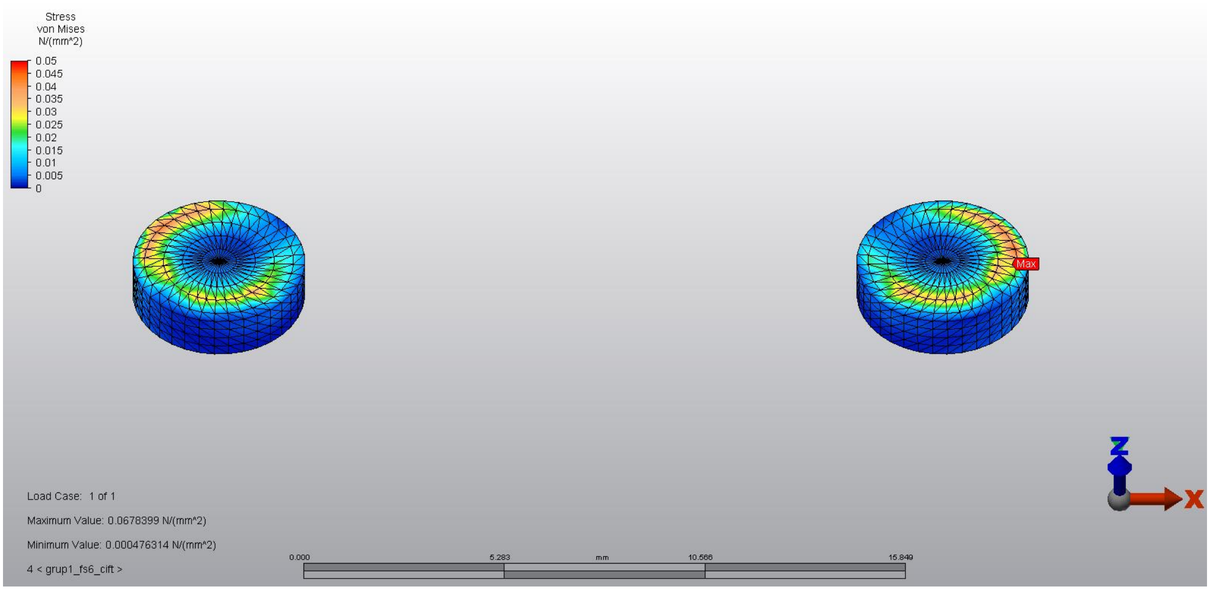
Stress
von Mises
N/(mm^2)
0.05
0.045
0.04
0.035
0.03
0.025
0.02
0.015
0.01
0.005
0
Max
Load Case: 1 of 1
Maximum Value: 0.0678399 N/(mm^2)
Minimum Value: 0.000476314 N/(mm^2)
4 < grup1_fs6_cift >
0.000
5.283
mm
10.566
15.849
Z
X

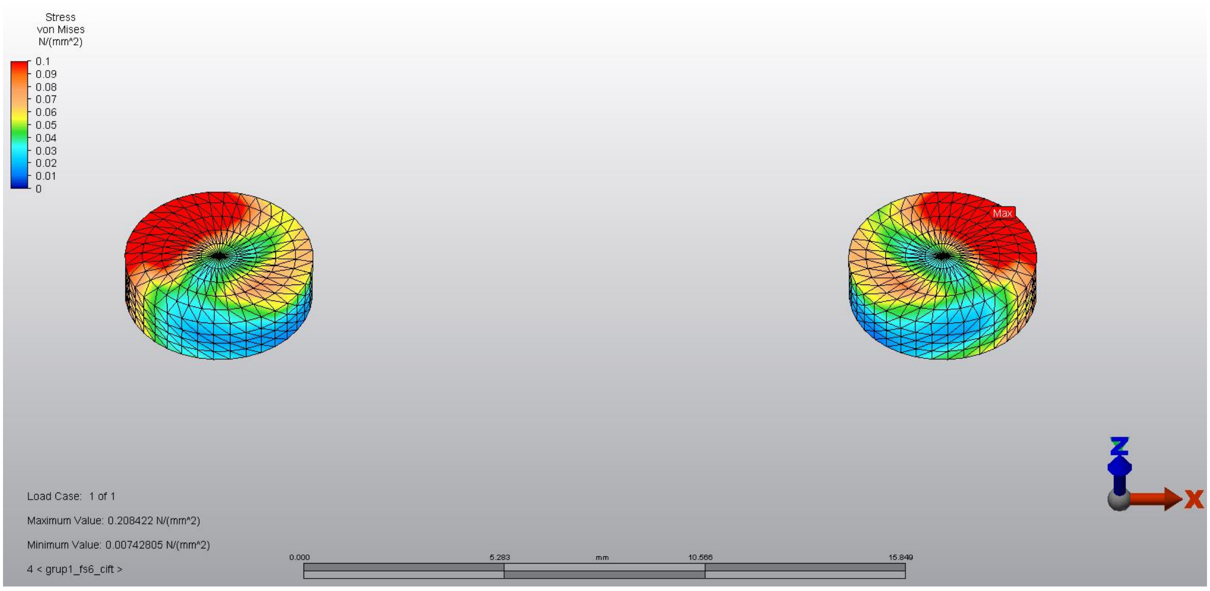
Stress
von Mises
N/(mm^2)
0.1
0.09
0.08
0.07
0.06
0.05
0.04
0.03
0.02
0.01
0
Max
Load Case: 1 of 1
Maximum Value: 0.208422 N/(mm^2)
Minimum Value: 0.00742805 N/(mm^2)
4 < grup1_fs6_cift >
0.000
5.283
mm
10.566
15.849
Z
X

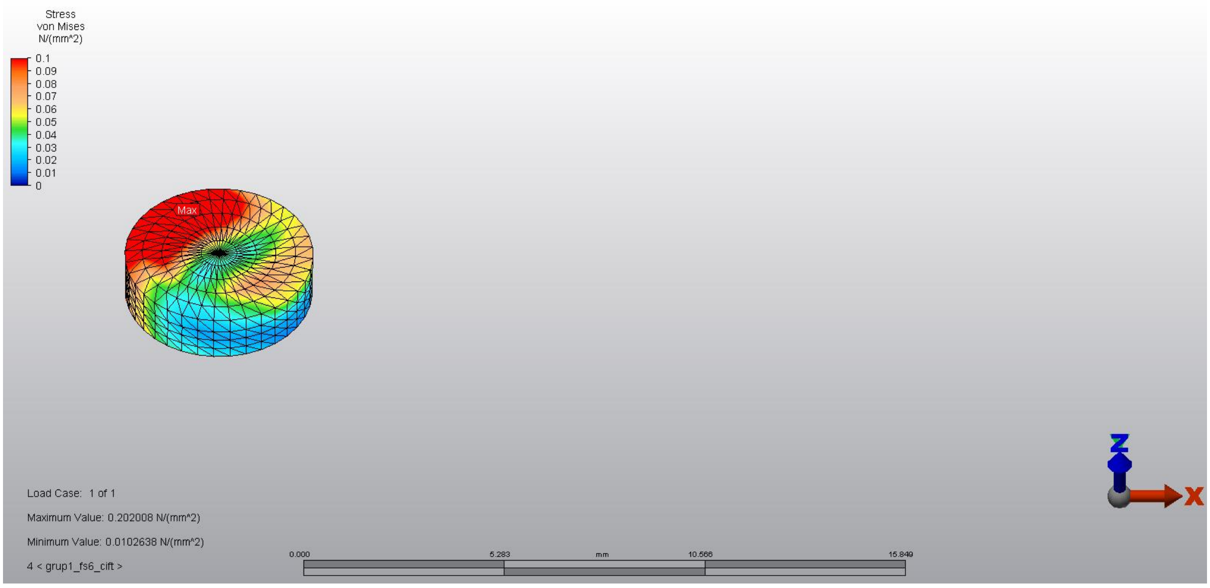
Stress
von Mises
N/(mm^2)
0.1
0.09
0.08
0.07
0.06
0.05
0.04
0.03
0.02
0.01
0
Max
Load Case: 1 of 1
Maximum Value: 0.202008 N/(mm^2)
Minimum Value: 0.0102638 N/(mm^2)
4 < grup1_fs6_cift >
0.000
5.283
mm
10.566
15.849
Z
X

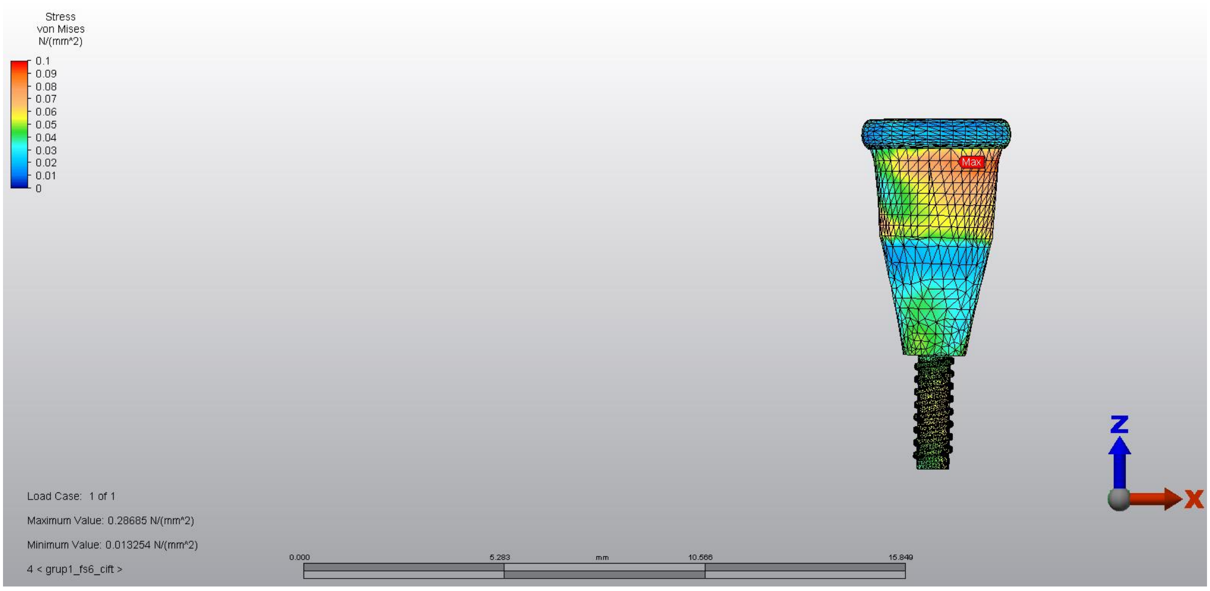
Stress
von Mises
N/(mm^2)
0.1
0.09
0.08
0.07
0.06
0.05
0.04
0.03
0.02
0.01
0
Max
Load Case: 1 of 1
Maximum Value: 0.28685 N/(mm^2)
Minimum Value: 0.013254 N/(mm^2)
4 < grup1_fs6_cift >
0.000
5.283
mm
10.566
15.849
Z
X

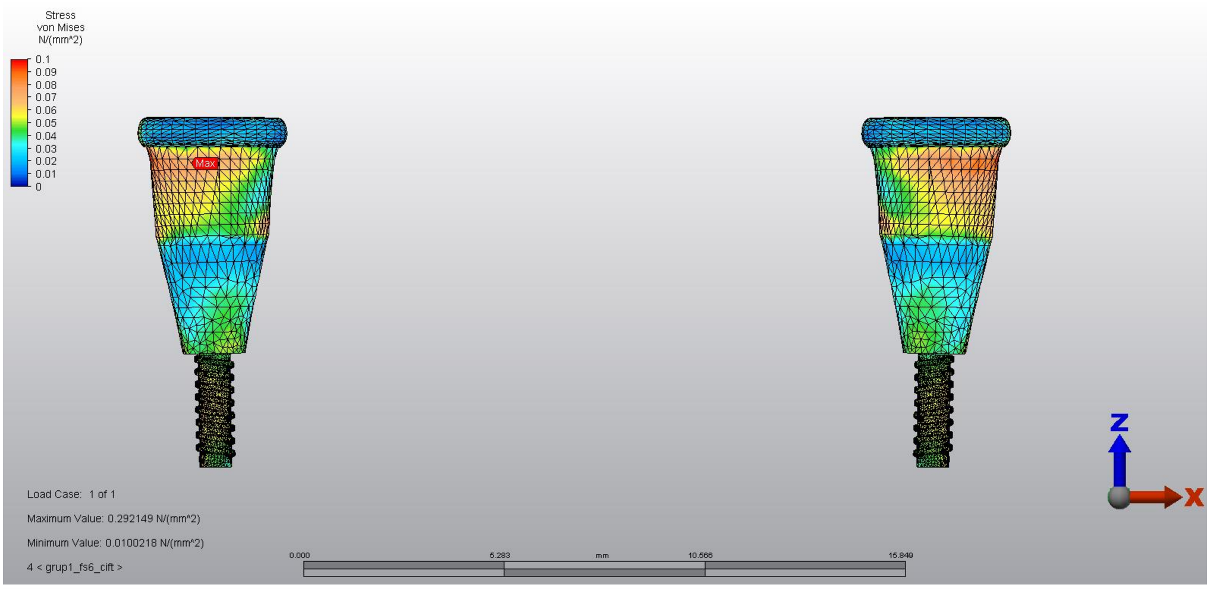

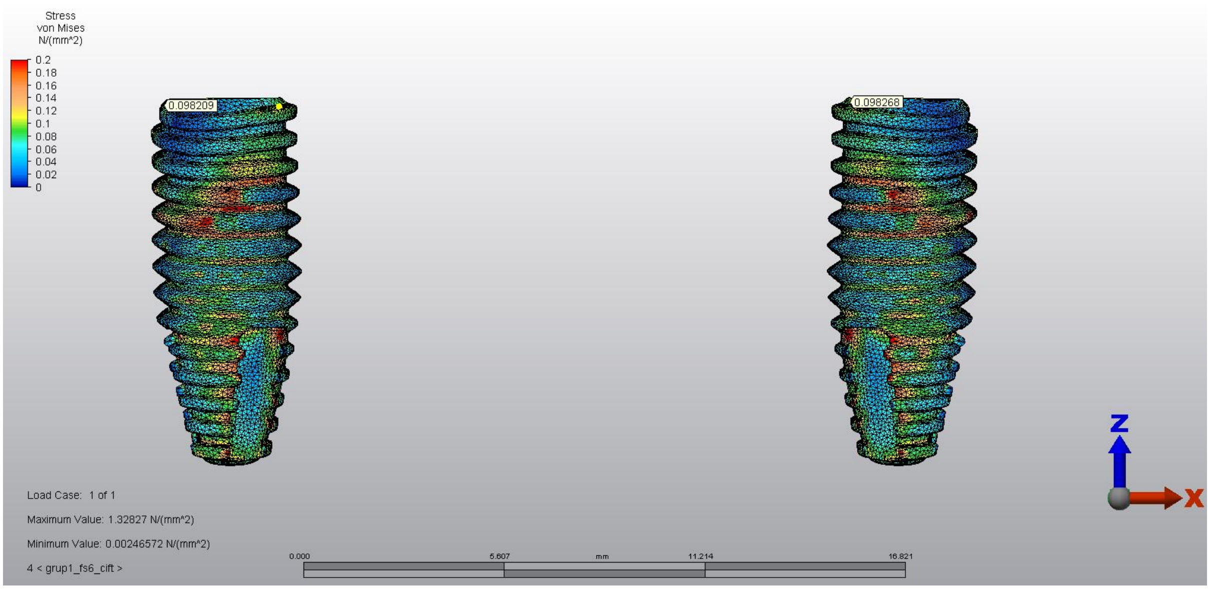

GRUP 02

Number of nodes = 136173

Number of elements = 718316

INCISAL

Number of nodes = 136206

Number of elements = 718322

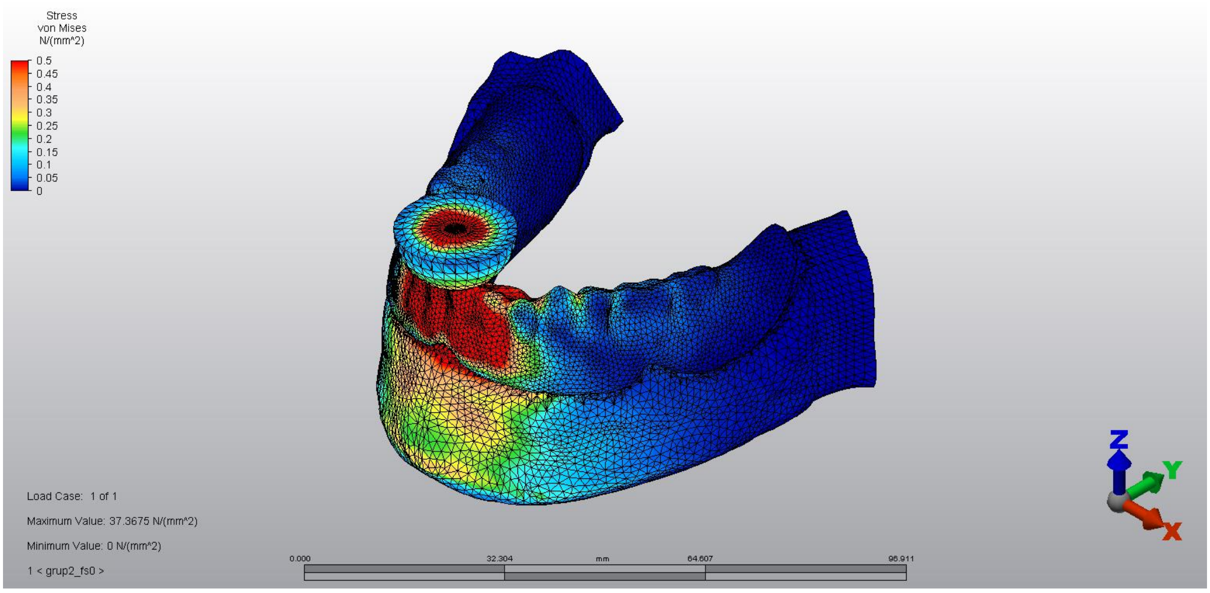
Stress
von Mises
N/(mm^2)
0.5
0.45
0.4
0.35
0.3
0.25
0.2
0.15
0.1
0.05
0
Load Case: 1 of 1
Maximum Value: 37.3675 N/(mm^2)
Minimum Value: 0 N/(mm^2)
1 < grup2_fs0 >
0.000
32.304
mm
64.607
96.911
Z
Y
X

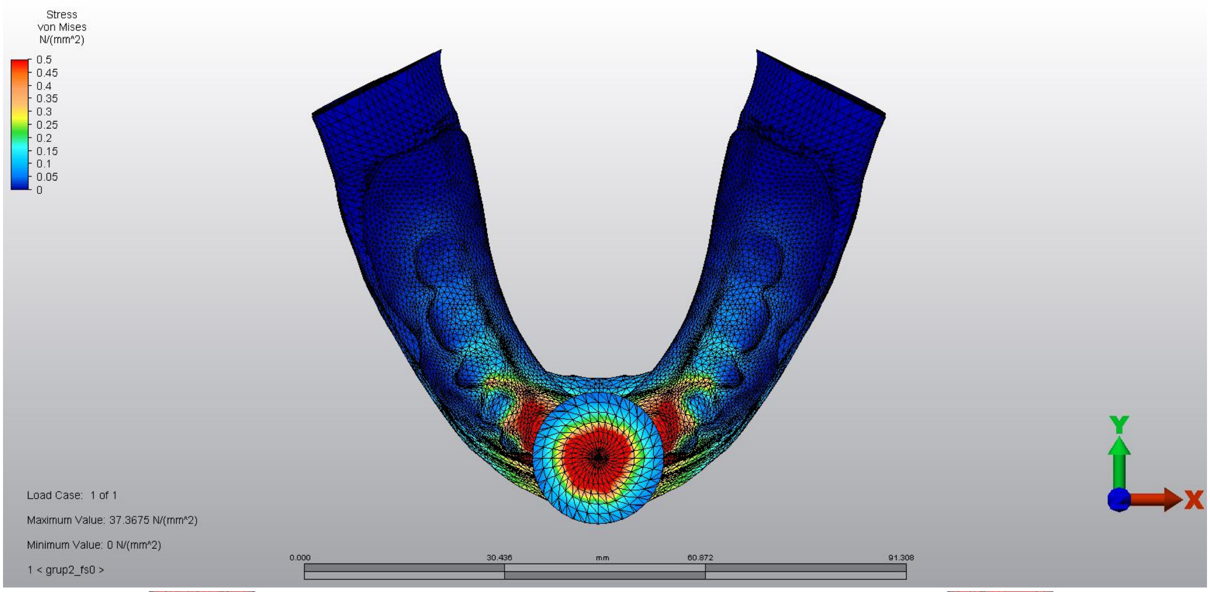
Stress
von Mises
N/(mm^2)
0.5
0.45
0.4
0.35
0.3
0.25
0.2
0.15
0.1
0.05
0
Load Case: 1 of 1
Maximum Value: 37.3675 N/(mm^2)
Minimum Value: 0 N/(mm^2)
1 < grup2_fs0 >
0.000
30.436
mm
60.872
91.308
Y
X

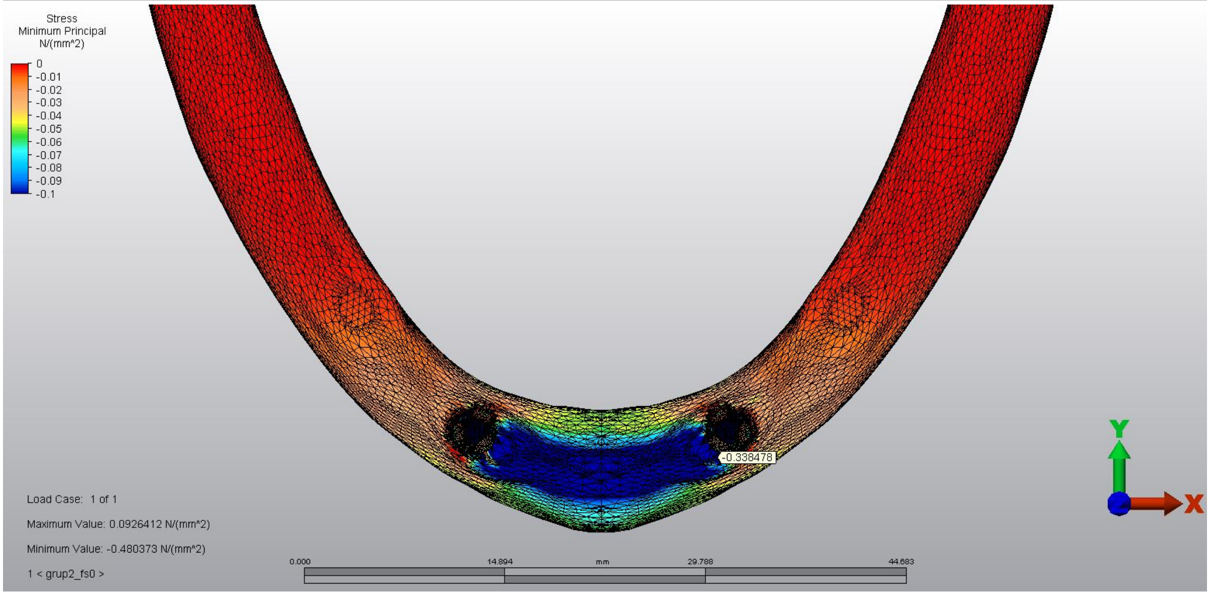
Stress
Minimum Principal
N/(mm^2)
0
-0.01
-0.02
-0.03
-0.04
-0.05
-0.06
-0.07
-0.08
-0.09
-0.1
-0.338478
Load Case: 1 of 1
Maximum Value: 0.0926412 N/(mm^2)
Minimum Value: -0.480373 N/(mm^2)
1 < grup2_fs0 >
0.000
14.894
mm
29.788
44.683
Y
X

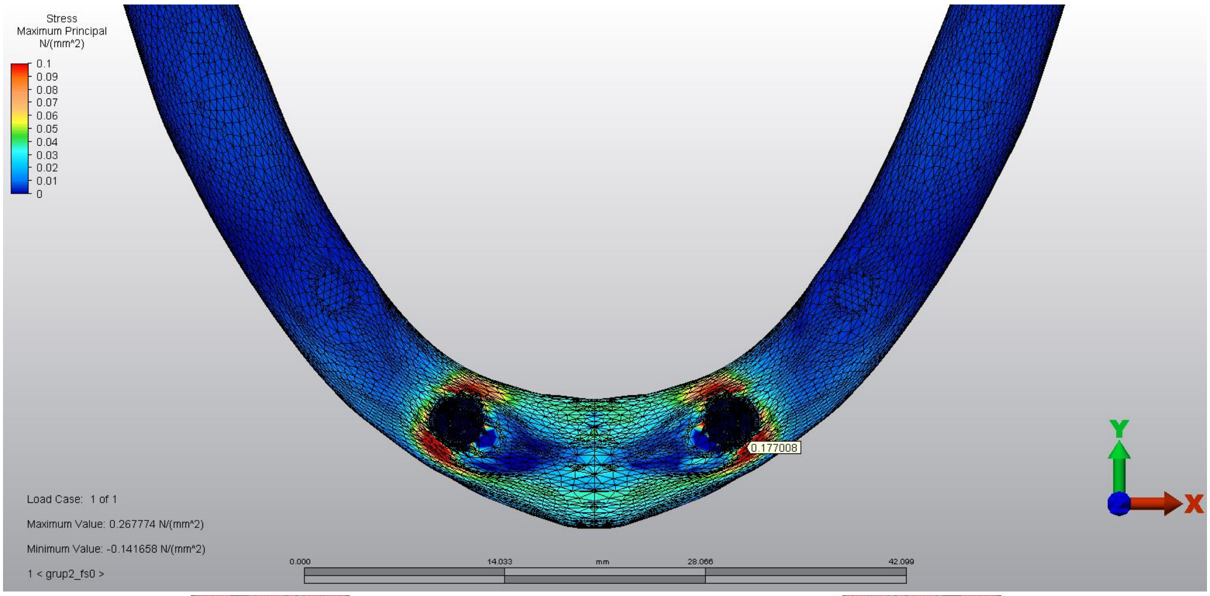
Stress
Maximum Principal
N/(mm^2)
0.1
0.09
0.08
0.07
0.06
0.05
0.04
0.03
0.02
0.01
0
0.177008
Load Case: 1 of 1
Maximum Value: 0.267774 N/(mm^2)
Minimum Value: -0.141658 N/(mm^2)
1 < grup2_fs0 >
0.000
14.033
mm
28.066
42.099
Y
X

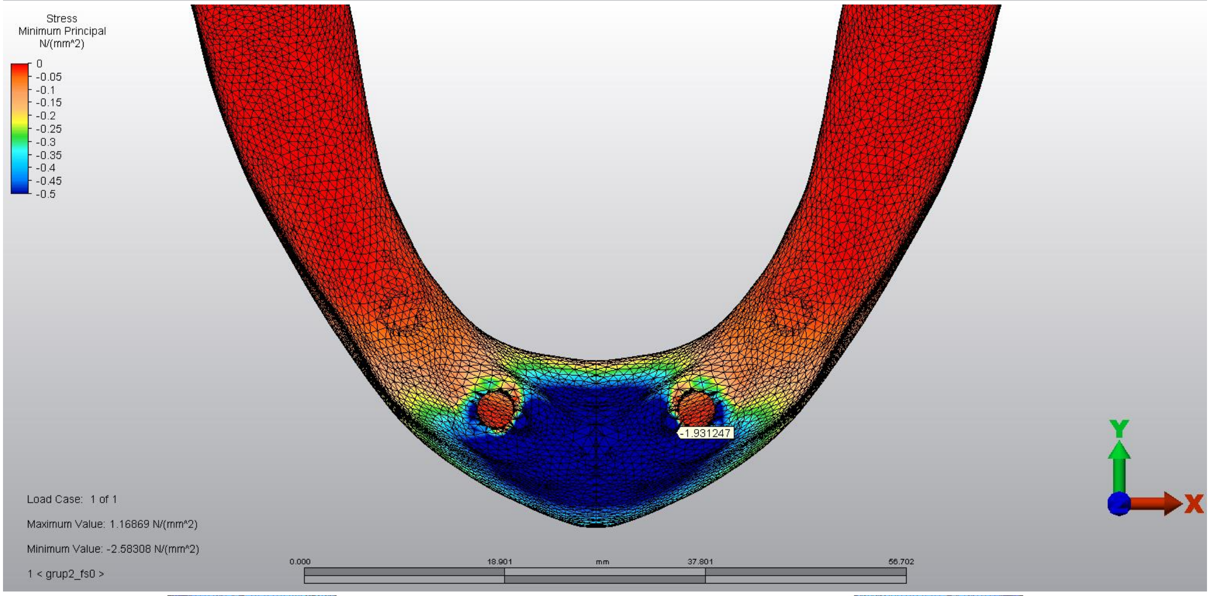
Stress
Minimum Principal
N/(mm^2)
0
-0.05
-0.1
-0.15
-0.2
-0.25
-0.3
-0.35
-0.4
-0.45
-0.5
-1.931247
Load Case: 1 of 1
Maximum Value: 1.16869 N/(mm^2)
Minimum Value: -2.58308 N/(mm^2)
1 < grup2_fs0 >
0.000
18.901
mm
37.801
56.702
Y
X

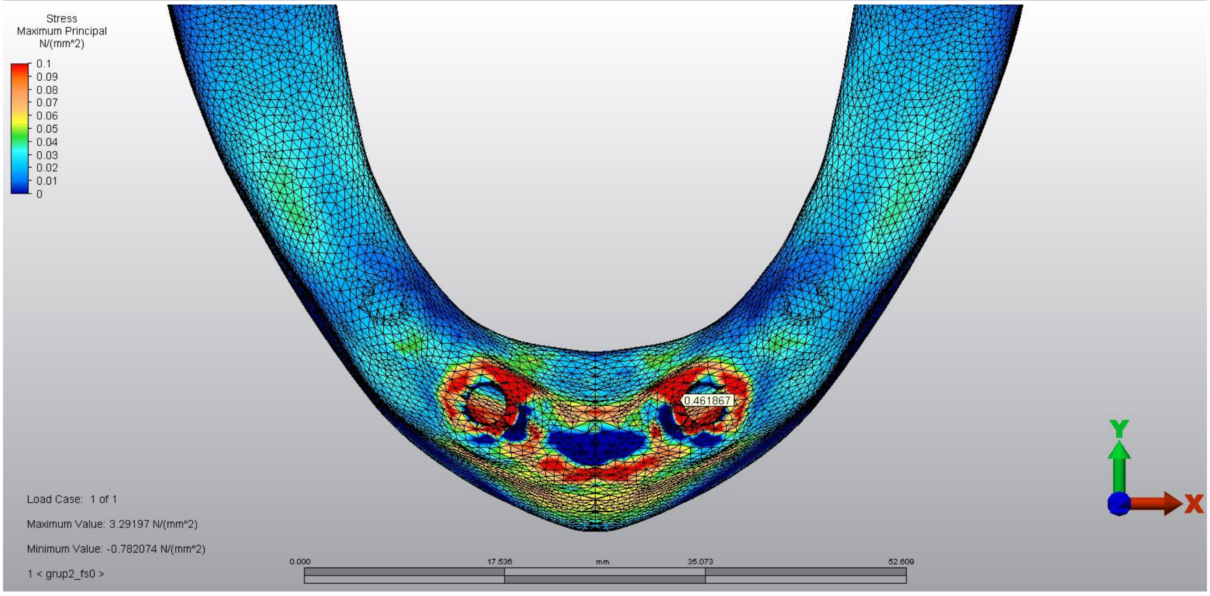
Stress
Maximum Principal
N/(mm^2)
0.1
0.09
0.08
0.07
0.06
0.05
0.04
0.03
0.02
0.01
0
0.461867
Load Case: 1 of 1
Maximum Value: 3.29197 N/(mm^2)
Minimum Value: -0.782074 N/(mm^2)
1 < grup2_fs0 >
0.000
17.536
mm
35.073
52.609
Y
X

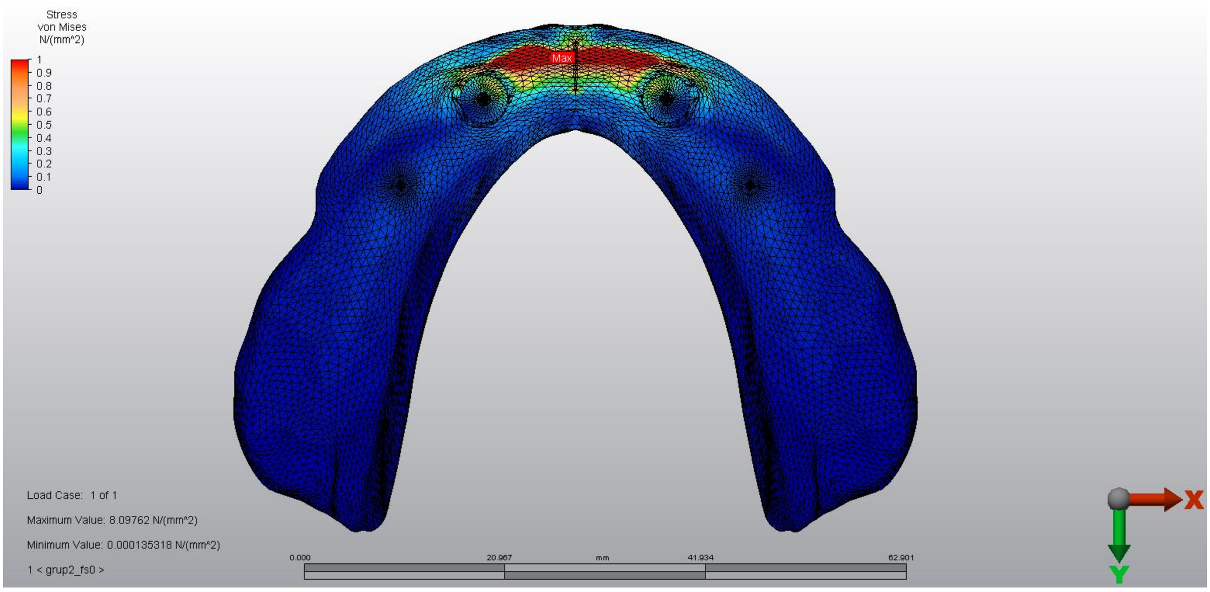
Stress
von Mises
N/(mm^2)
1
0.9
0.8
0.7
0.6
0.5
0.4
0.3
0.2
0.1
0
Max
Load Case: 1 of 1
Maximum Value: 8.09762 N/(mm^2)
Minimum Value: 0.000135318 N/(mm^2)
1 < grup2_fs0 >
0.000
20.967
mm
41.934
62.901
X
Y

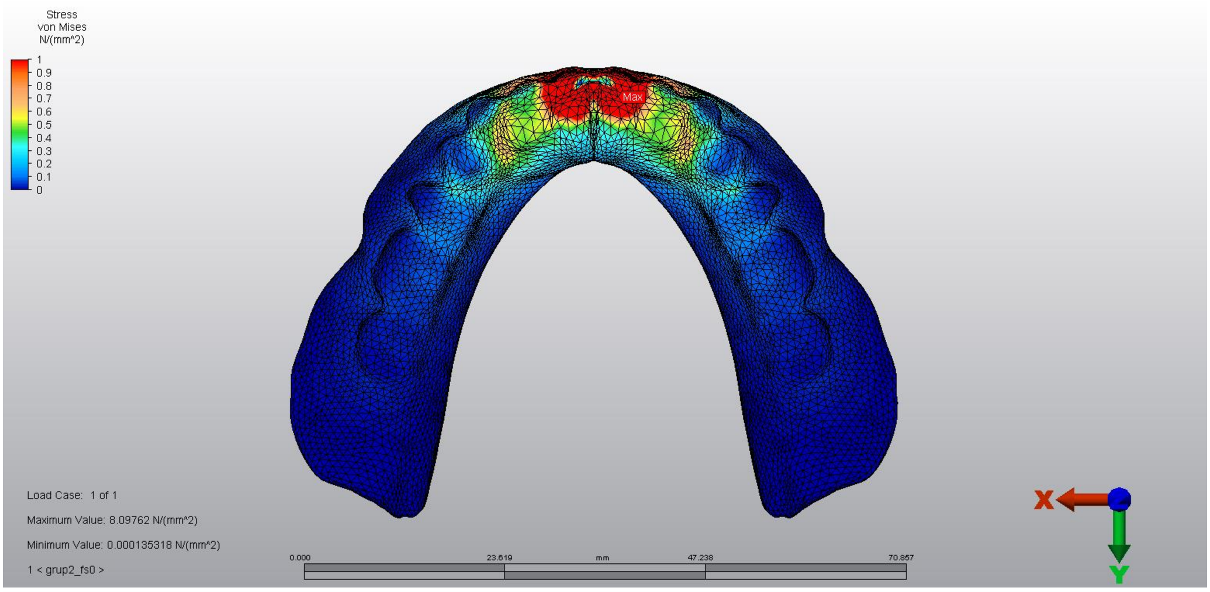
Stress
von Mises
N/(mm^2)
1
0.9
0.8
0.7
0.6
0.5
0.4
0.3
0.2
0.1
0
Max
Load Case: 1 of 1
Maximum Value: 8.09762 N/(mm^2)
Minimum Value: 0.000135318 N/(mm^2)
1 < grup2_fs0 >
0.000
23.619
mm
47.238
70.857
X
Y

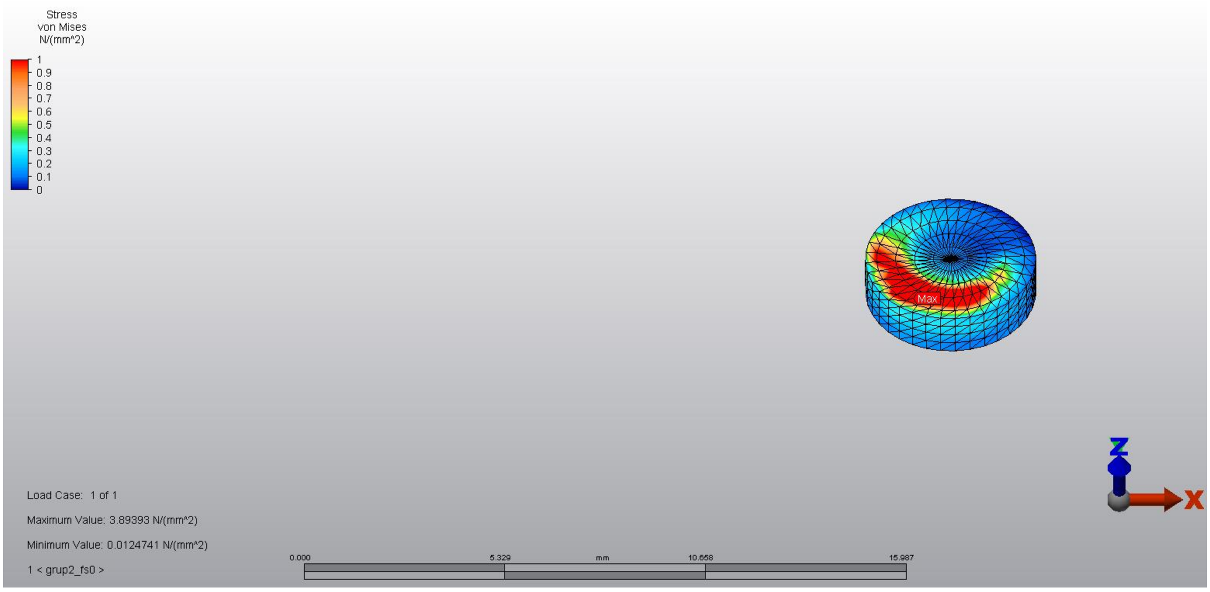
Stress
von Mises
N/(mm^2)
1
0.9
0.8
0.7
0.6
0.5
0.4
0.3
0.2
0.1
0
Max
Load Case: 1 of 1
Maximum Value: 3.89393 N/(mm^2)
Minimum Value: 0.0124741 N/(mm^2)
1 < grup2_fs0 >
0.000
5.329
mm
10.658
15.987
Z
X

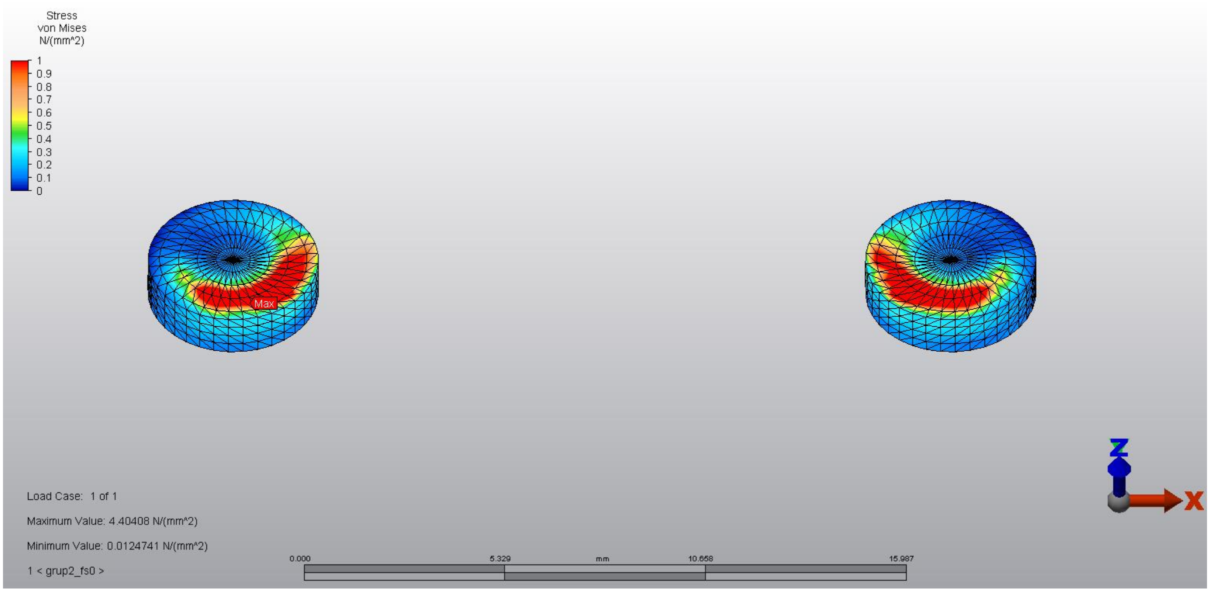
Stress
von Mises
N/(mm^2)
1
0.9
0.8
0.7
0.6
0.5
0.4
0.3
0.2
0.1
0
Max
Load Case: 1 of 1
Maximum Value: 4.40408 N/(mm^2)
Minimum Value: 0.0124741 N/(mm^2)
1 < grup2_fs0 >
0.000
5.329
mm
10.658
15.987
Z
X

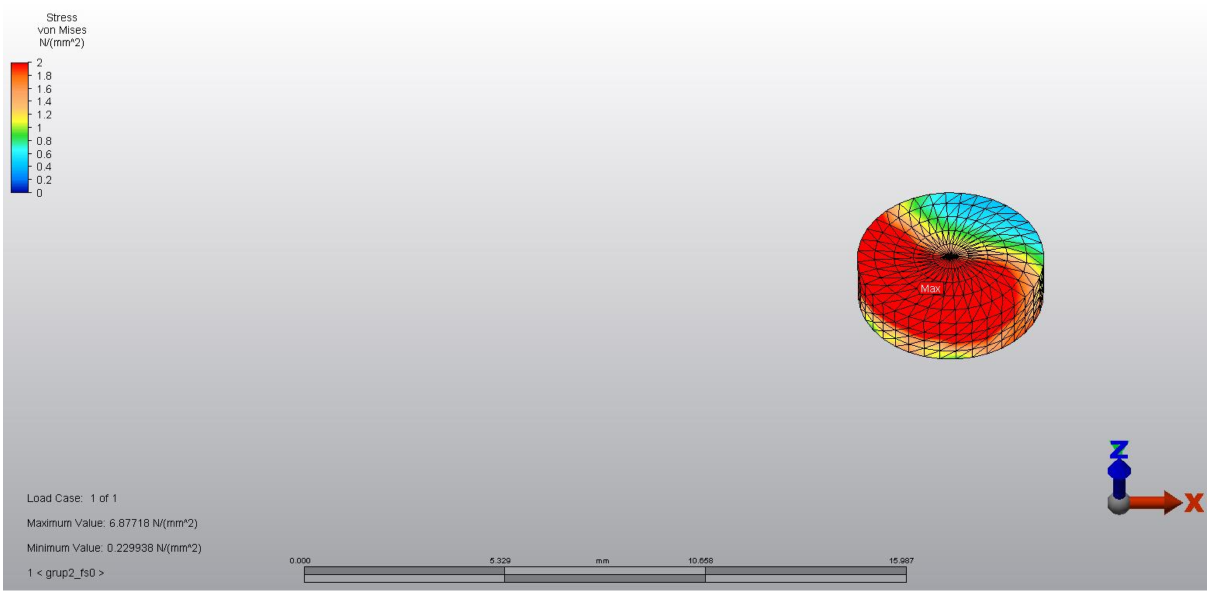
Stress
von Mises
N/(mm^2)
2
1.8
1.6
1.4
1.2
1
0.8
0.6
0.4
0.2
0
Max
Load Case: 1 of 1
Maximum Value: 6.87718 N/(mm^2)
Minimum Value: 0.229938 N/(mm^2)
1 < grup2_fs0 >
0.000
5.329
mm
10.658
15.987
Z
X

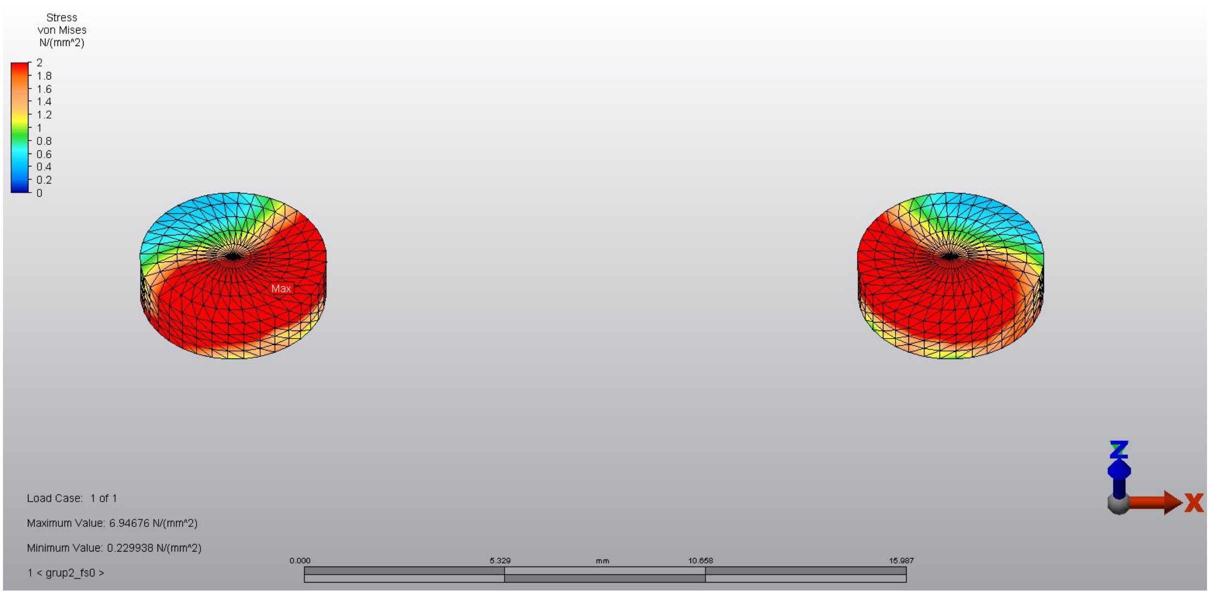
Stress
von Mises
N/(mm^2)
2
1.8
1.6
1.4
1.2
1
0.8
0.6
0.4
0.2
0
Max
Load Case: 1 of 1
Maximum Value: 6.94676 N/(mm^2)
Minimum Value: 0.229938 N/(mm^2)
1 < grup2_fs0 >
0.000
5.329
mm
10.658
15.987
Z
X

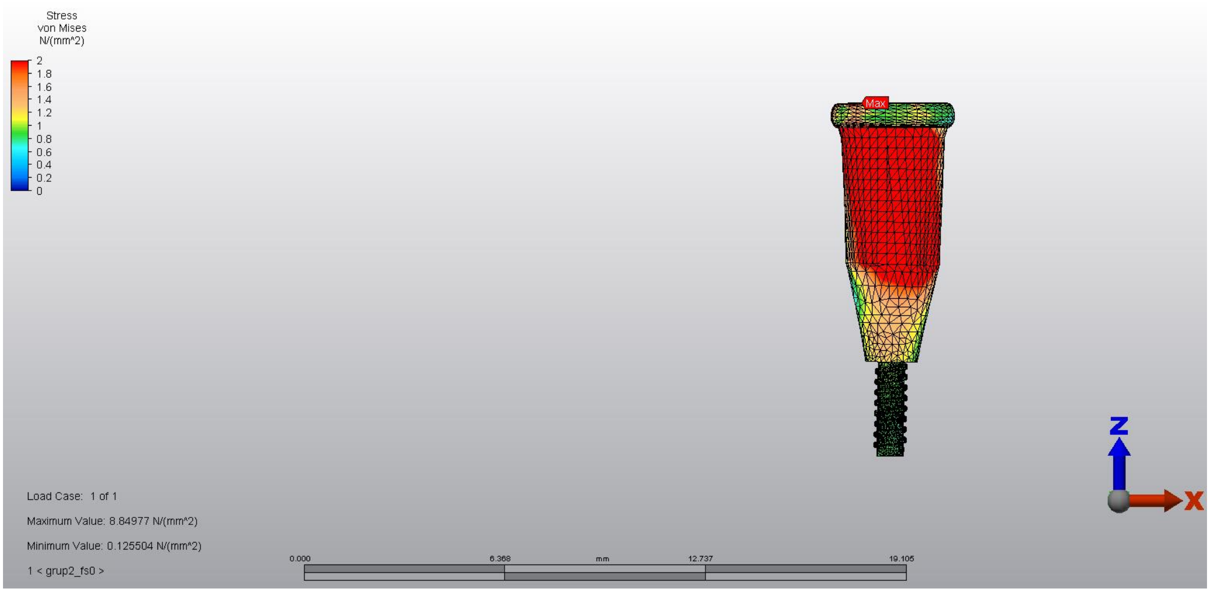
Stress
von Mises
N/(mm^2)
2
1.8
1.6
1.4
1.2
1
0.8
0.6
0.4
0.2
0
Max
Load Case: 1 of 1
Maximum Value: 8.84977 N/(mm^2)
Minimum Value: 0.125504 N/(mm^2)
1 < grup2_fs0 >
0.000
6.368
mm
12.737
19.105
Z
X

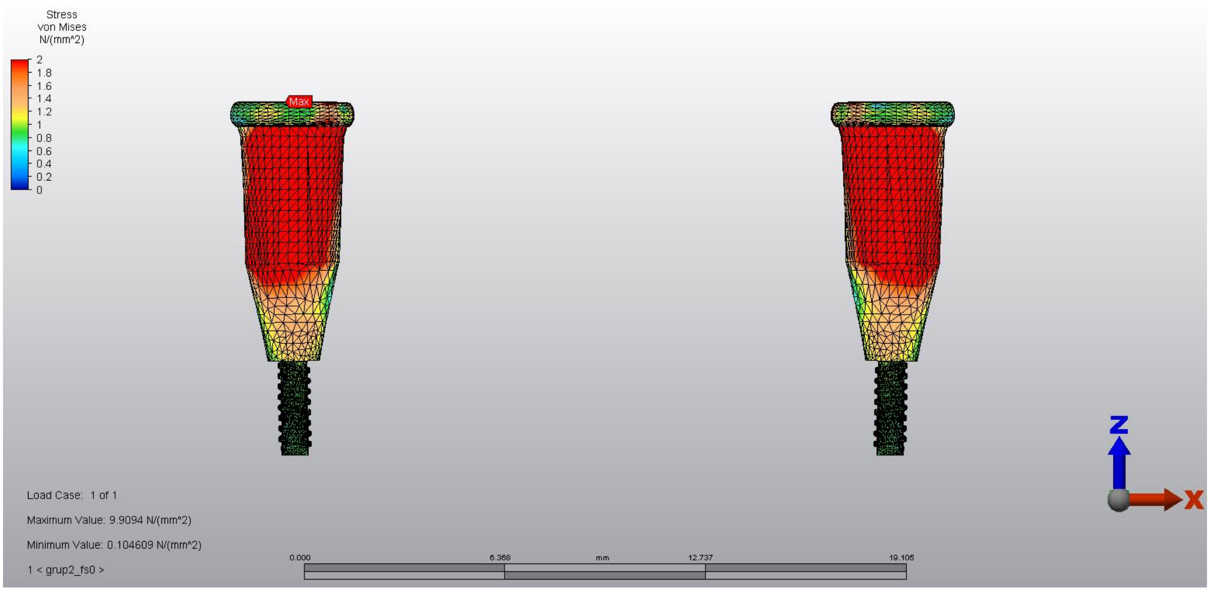
Stress
von Mises
N/(mm^2)
2
1.8
1.6
1.4
1.2
1
0.8
0.6
0.4
0.2
0
Max
Load Case: 1 of 1
Maximum Value: 9.9094 N/(mm^2)
Minimum Value: 0.104609 N/(mm^2)
1 < grup2_fs0 >
0.000
6.368
mm
12.737
19.105
Z
X

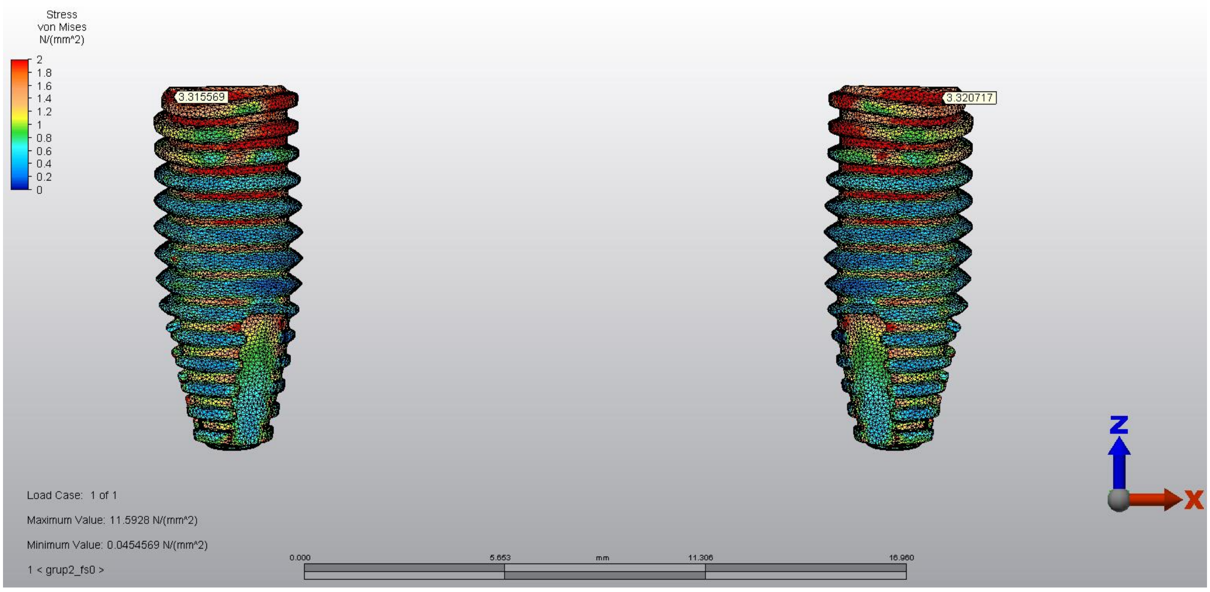
Stress
von Mises
N/(mm^2)
2
1.8
1.6
1.4
1.2
1
0.8
0.6
0.4
0.2
0
3.315569
3.320717
Load Case: 1 of 1
Maximum Value: 11.5928 N/(mm^2)
Minimum Value: 0.0454569 N/(mm^2)
1 < grup2_fs0 >
0.000
5.653
mm
11.306
16.960
Z
X

UNILATERAL

Number of nodes = 136173

Number of elements = 718316

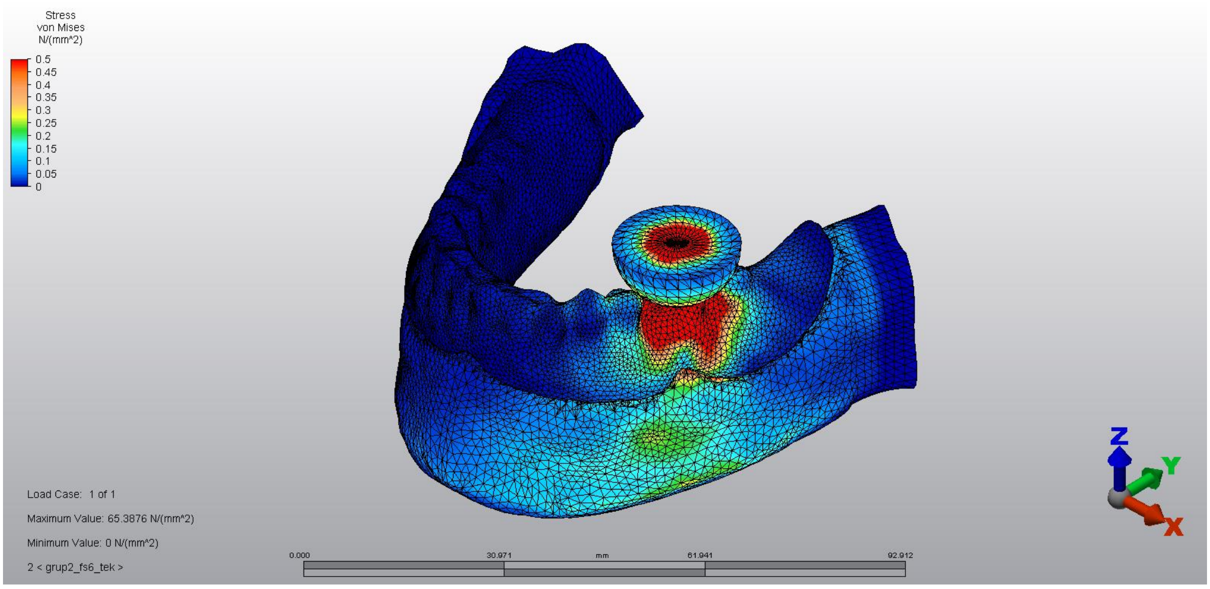

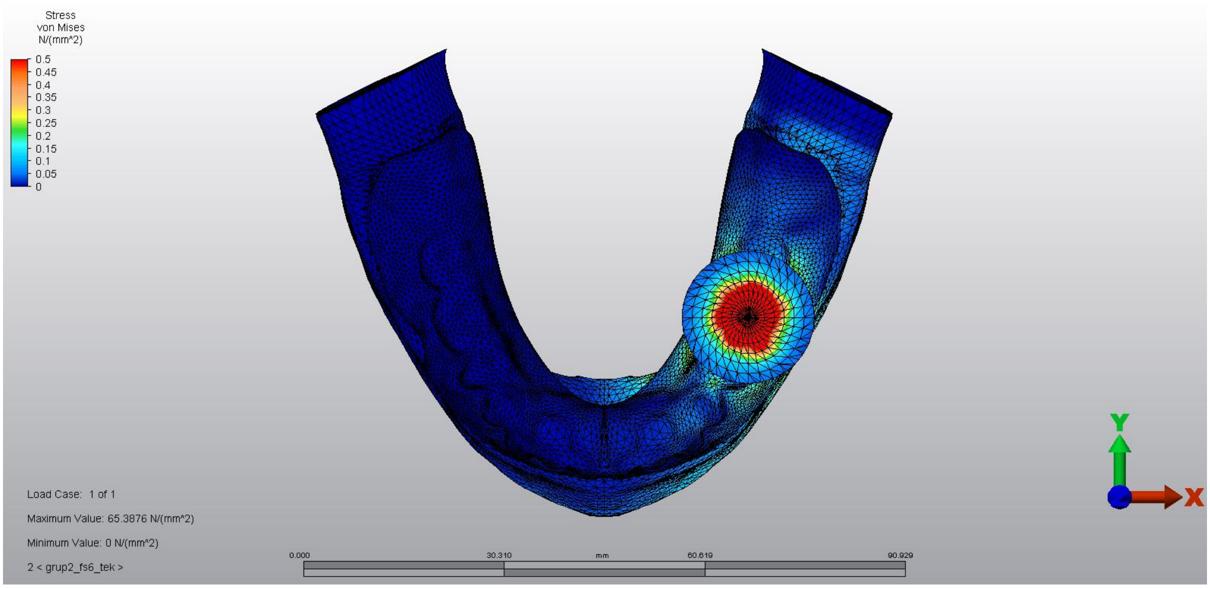

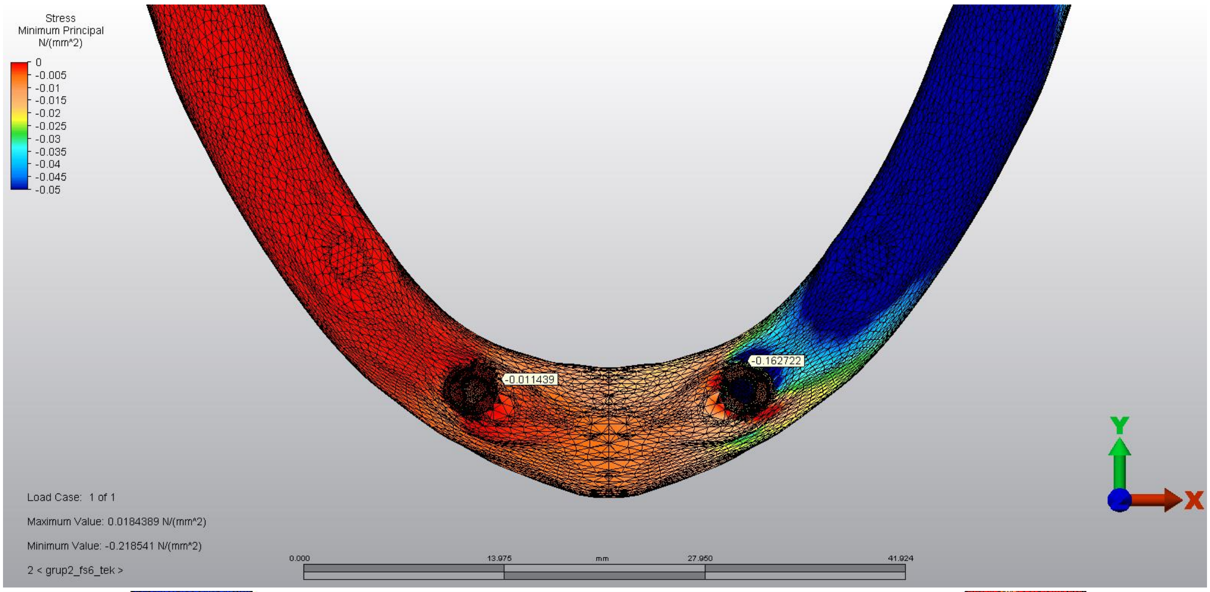

Stress
Minimum Principal
N/(mm^2)
0
-0.005
-0.01
-0.015
-0.02
-0.025
-0.03
-0.035
-0.04
-0.045
-0.05
-0.011439
-0.162722
Load Case: 1 of 1
Maximum Value: 0.0184389 N/(mm^2)
Minimum Value: -0.218541 N/(mm^2)
2 < grup2_fs6_tek >
0.000
13.975
mm
27.950
41.924

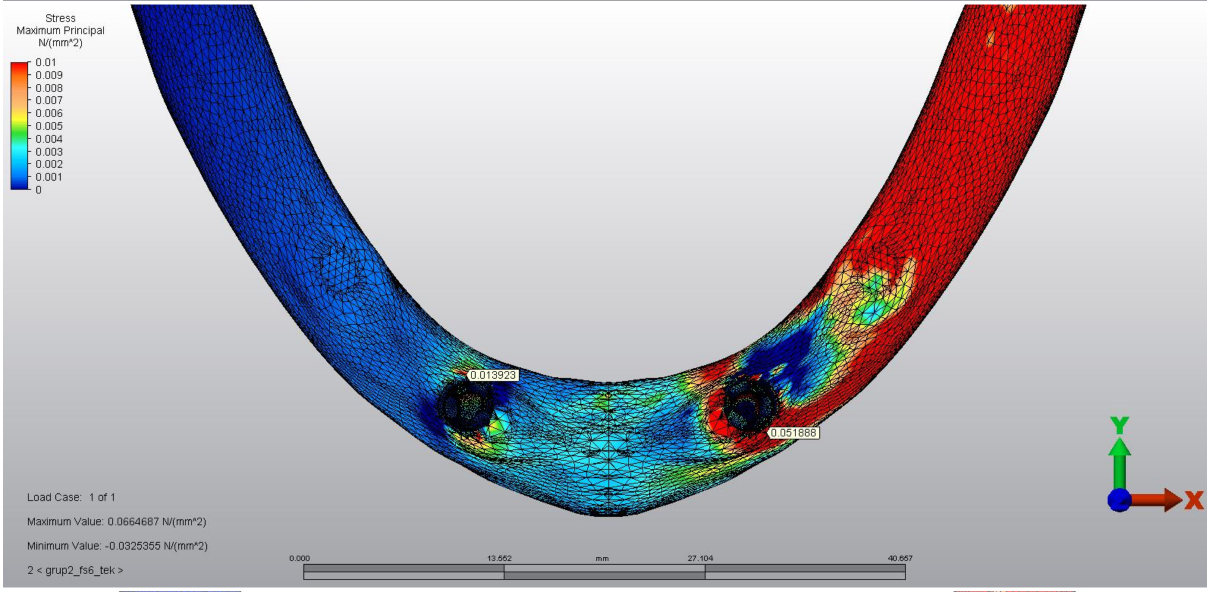

Stress
Maximum Principal
N/(mm^2)
0.01
0.009
0.008
0.007
0.006
0.005
0.004
0.003
0.002
0.001
0
0.013923
0.051888
Load Case: 1 of 1
Maximum Value: 0.0664687 N/(mm^2)
Minimum Value: -0.0325355 N/(mm^2)
2 < grup2_fs6_tek >
0.000
13.552
mm
27.104
40.657

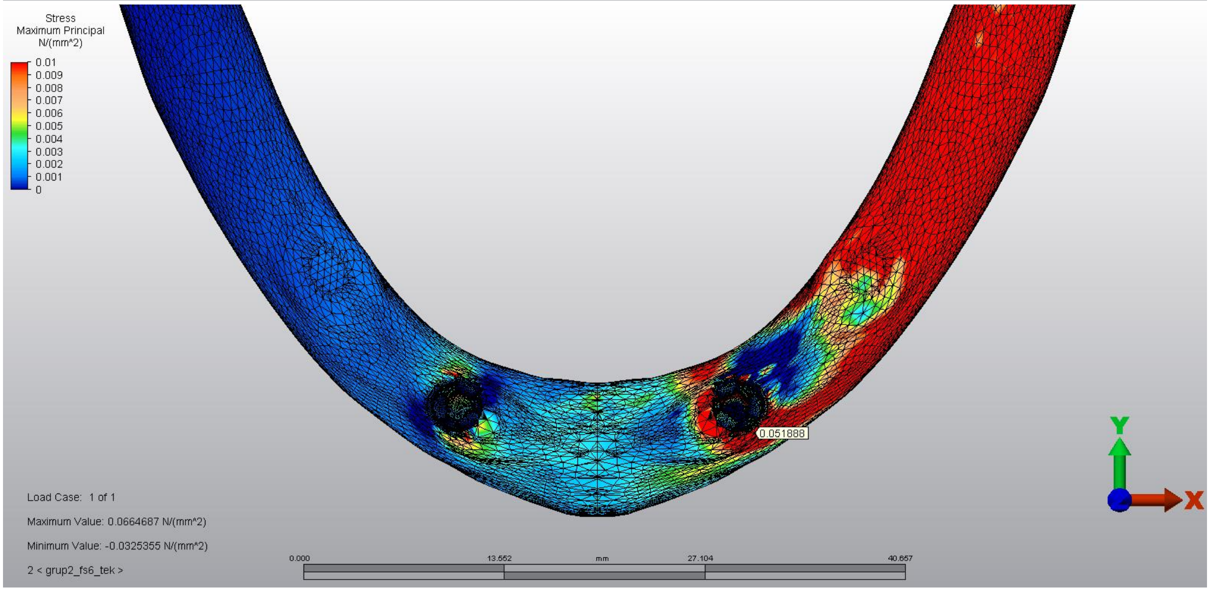

Stress
Maximum Principal
N/(mm^2)
0.01
0.009
0.008
0.007
0.006
0.005
0.004
0.003
0.002
0.001
0
0.051888
Load Case: 1 of 1
Maximum Value: 0.0664687 N/(mm^2)
Minimum Value: -0.0325355 N/(mm^2)
2 < grup2_fs6_tek >
0.000
13.552
mm
27.104
40.657

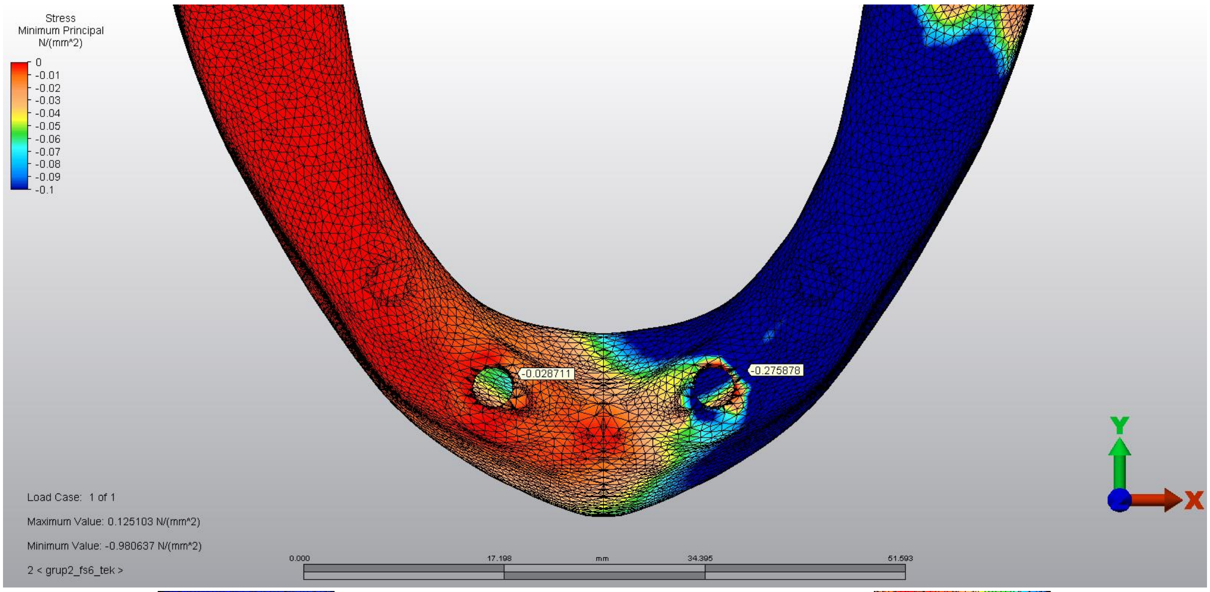
Stress
Minimum Principal
N/(mm^2)
0
-0.01
-0.02
-0.03
-0.04
-0.05
-0.06
-0.07
-0.08
-0.09
-0.1
-0.028711
-0.275878
Load Case: 1 of 1
Maximum Value: 0.125103 N/(mm^2)
Minimum Value: -0.980637 N/(mm^2)
2 < grup2_fs6_tek >
0.000
17.198
mm
34.395
51.593
Y
X

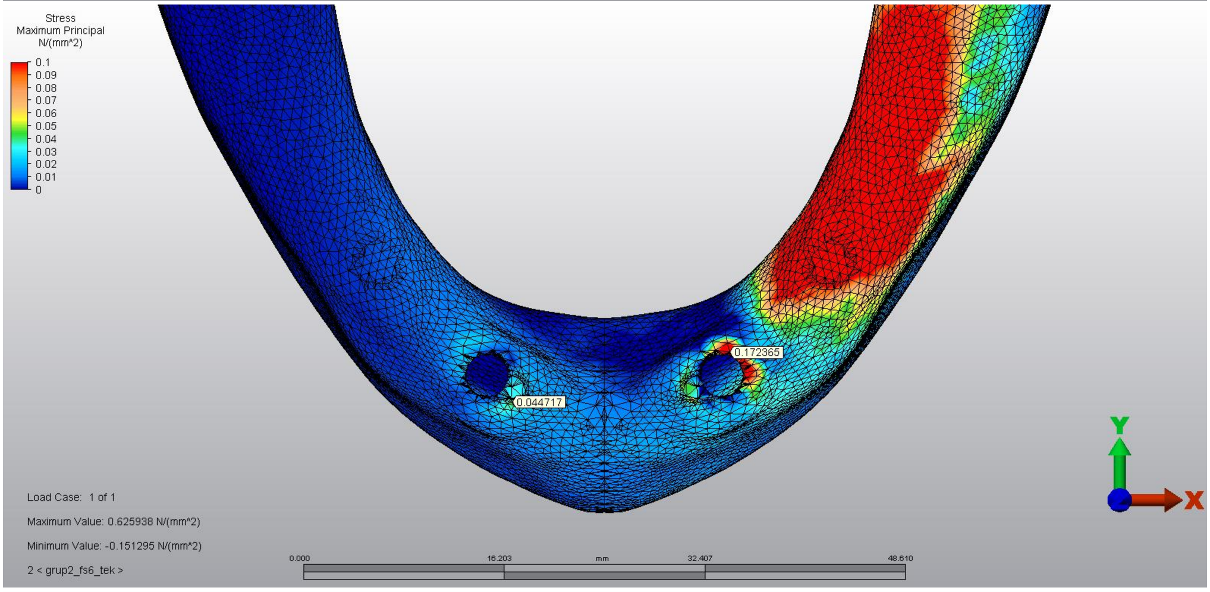
Stress
Maximum Principal
N/(mm^2)
0.1
0.09
0.08
0.07
0.06
0.05
0.04
0.03
0.02
0.01
0
0.172365
0.044717
Load Case: 1 of 1
Maximum Value: 0.625938 N/(mm^2)
Minimum Value: -0.151295 N/(mm^2)
2 < grup2_fs6_tek >
0.000
16.203
mm
32.407
48.610
Y
X

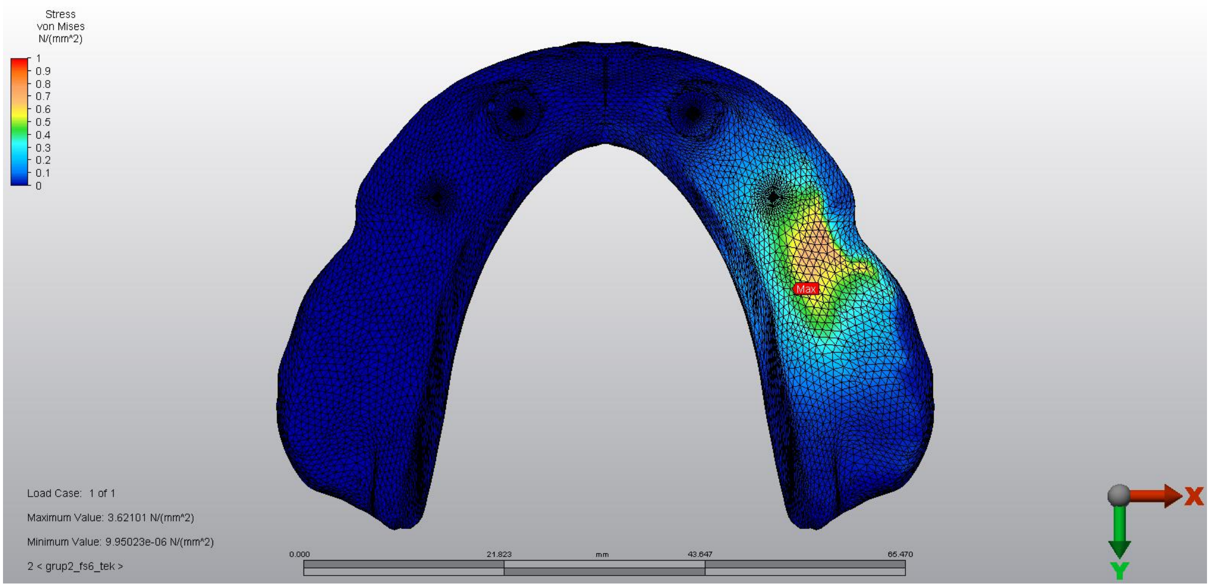
Stress
von Mises
N/(mm^2)
1
0.9
0.8
0.7
0.6
0.5
0.4
0.3
0.2
0.1
0
Max
Load Case: 1 of 1
Maximum Value: 3.62101 N/(mm^2)
Minimum Value: 9.95023e-06 N/(mm^2)
2 < grup2_fs6_tek >
0.000
21.823
mm
43.647
65.470
X
Y

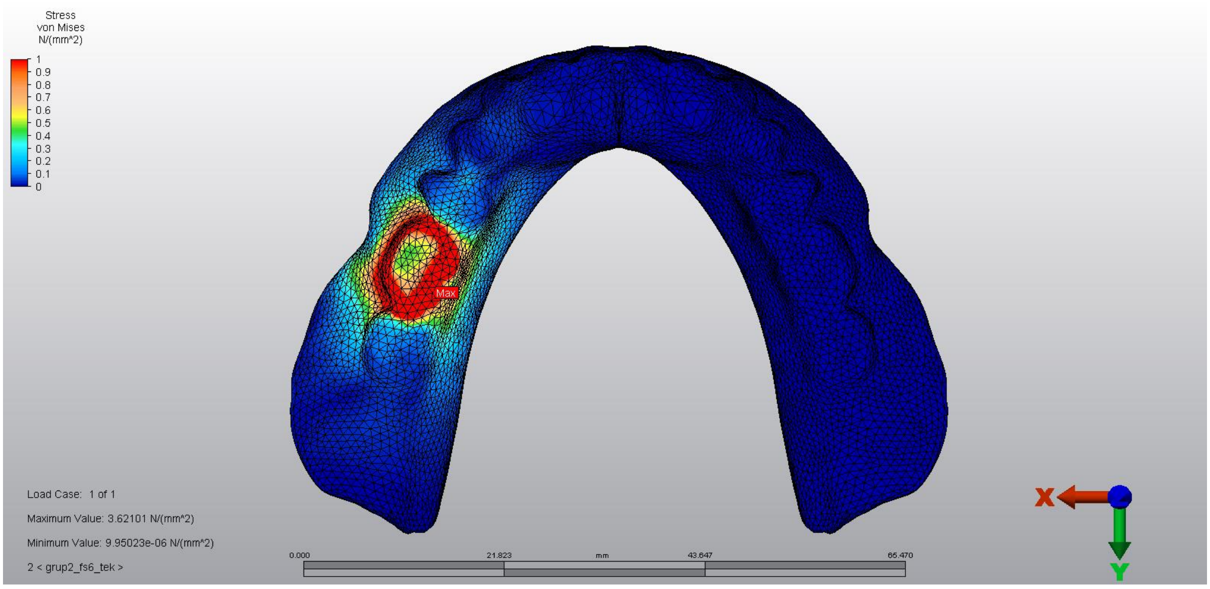
Stress
von Mises
N/(mm^2)
1
0.9
0.8
0.7
0.6
0.5
0.4
0.3
0.2
0.1
0
Max
Load Case: 1 of 1
Maximum Value: 3.62101 N/(mm^2)
Minimum Value: 9.95023e-06 N/(mm^2)
2 < grup2_fs6_tek >
0.000
21.823
mm
43.647
65.470
X
Y

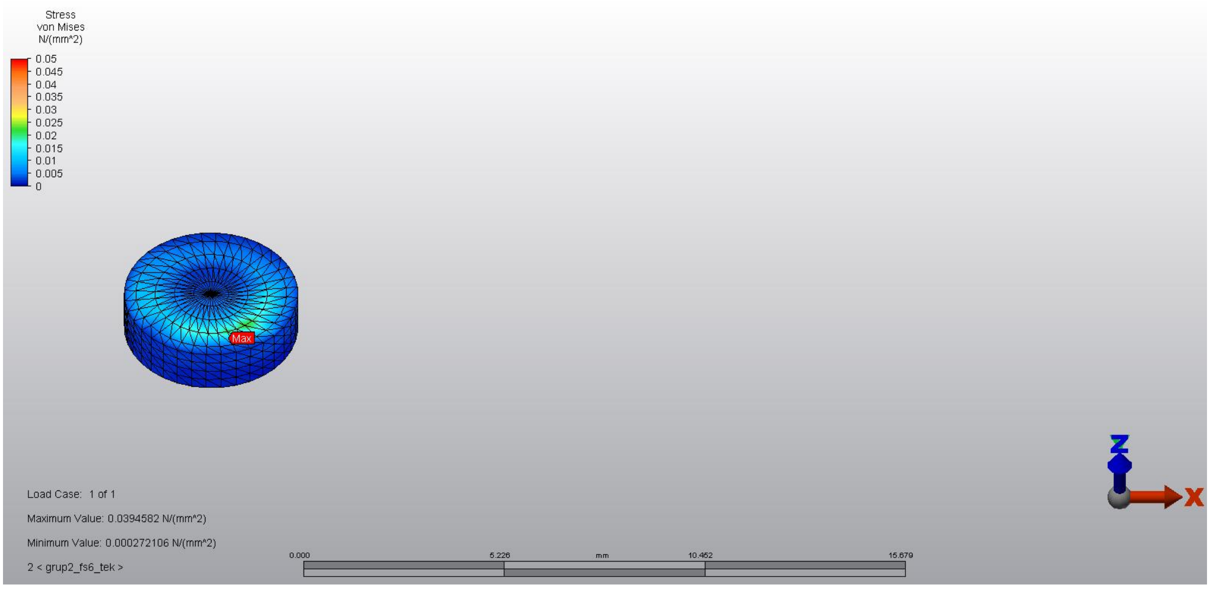
Stress
von Mises
N/(mm^2)
0.05
0.045
0.04
0.035
0.03
0.025
0.02
0.015
0.01
0.005
0
Max
Load Case: 1 of 1
Maximum Value: 0.0394582 N/(mm^2)
Minimum Value: 0.000272106 N/(mm^2)
2 < grup2_fs6_tek >
0.000
5.226
mm
10.452
15.679
Z
X

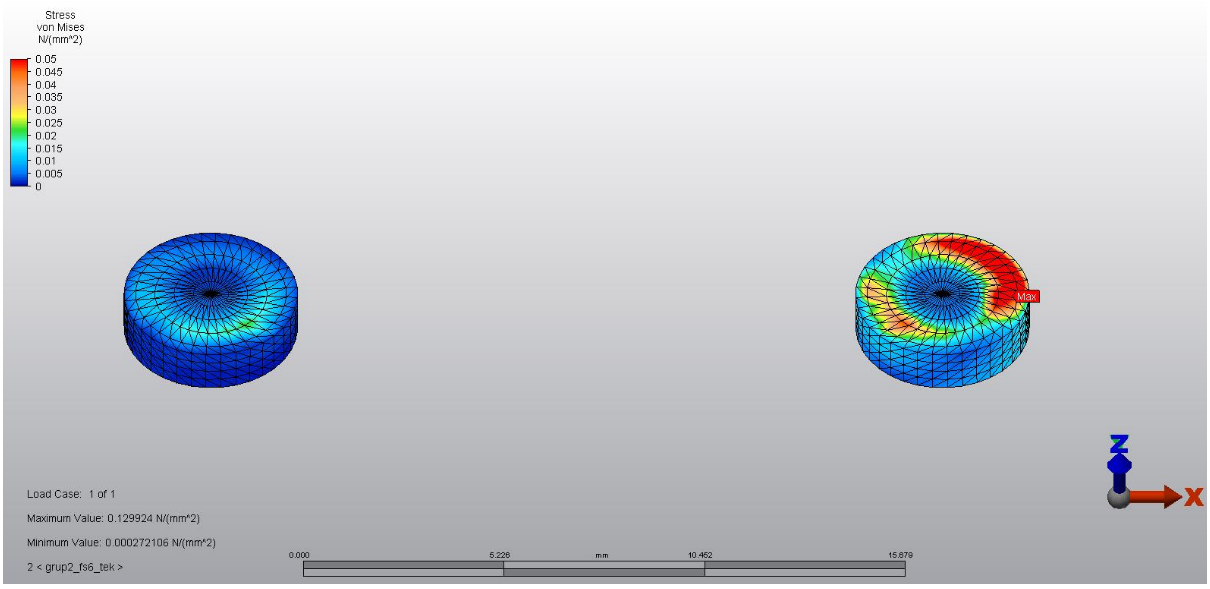
Stress
von Mises
N/(mm^2)
0.05
0.045
0.04
0.035
0.03
0.025
0.02
0.015
0.01
0.005
0
Max
Load Case: 1 of 1
Maximum Value: 0.129924 N/(mm^2)
Minimum Value: 0.000272106 N/(mm^2)
2 < grup2_fs6_tek >
0.000
5.226
mm
10.452
15.679
Z
X

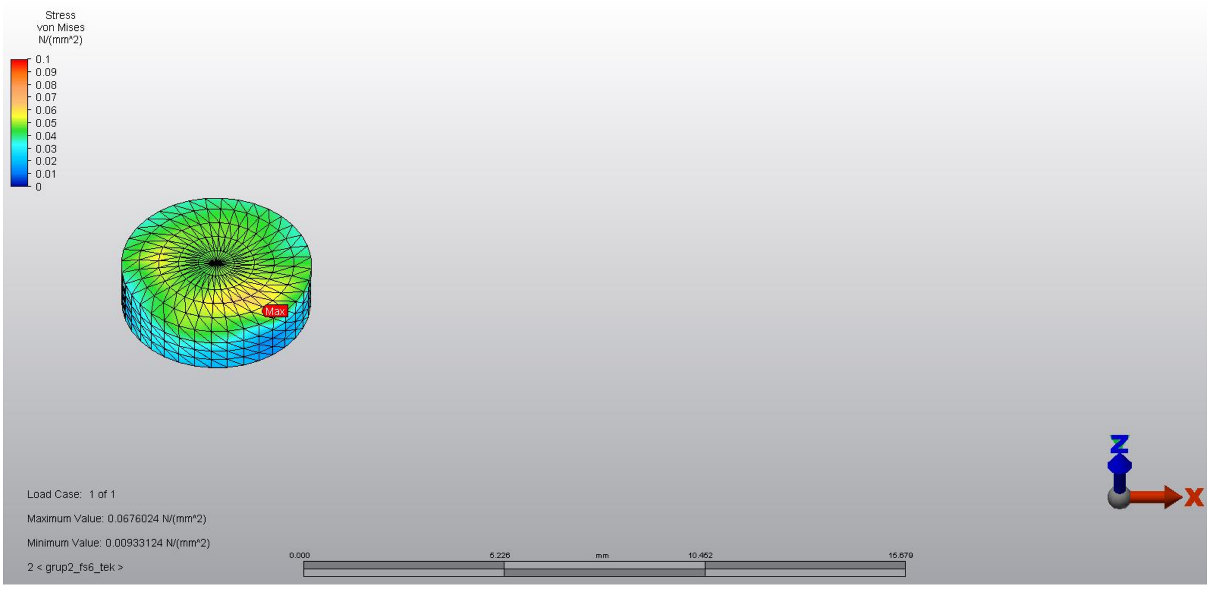
Stress
von Mises
N/(mm^2)
0.1
0.09
0.08
0.07
0.06
0.05
0.04
0.03
0.02
0.01
0
Max
Load Case: 1 of 1
Maximum Value: 0.0676024 N/(mm^2)
Minimum Value: 0.00933124 N/(mm^2)
2 < grup2_fs6_tek >
0.000
5.226
mm
10.452
15.679
Z
X

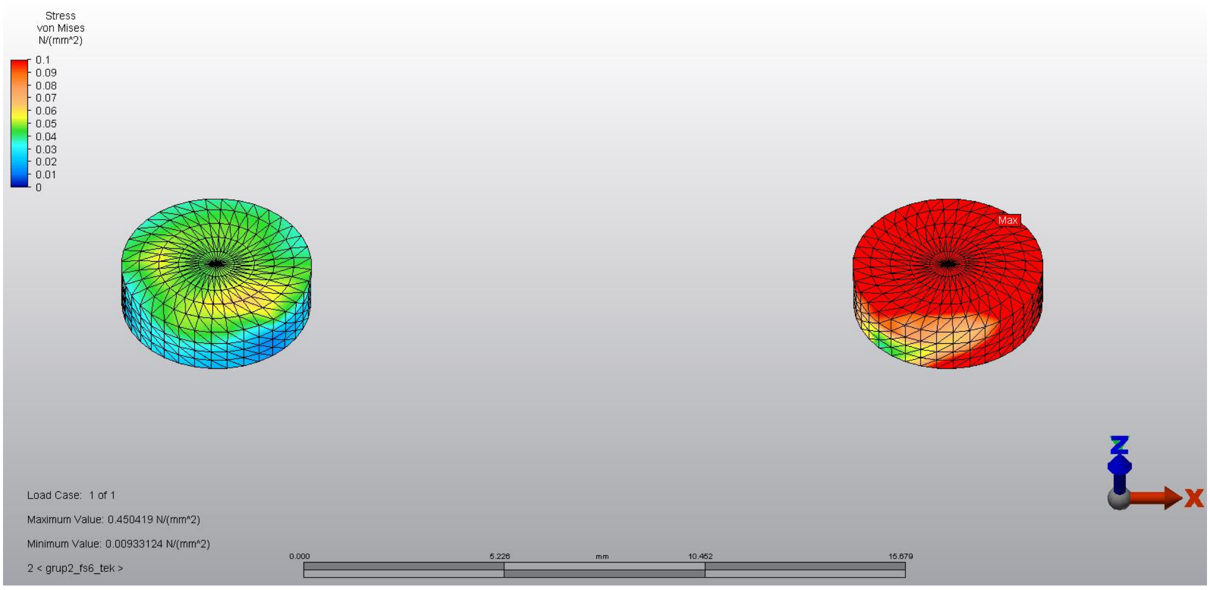
Stress
von Mises
N/(mm^2)
0.1
0.09
0.08
0.07
0.06
0.05
0.04
0.03
0.02
0.01
0
Max
Load Case: 1 of 1
Maximum Value: 0.450419 N/(mm^2)
Minimum Value: 0.00933124 N/(mm^2)
2 < grup2_fs6_tek >
0.000
5.226
mm
10.452
15.679
Z
X

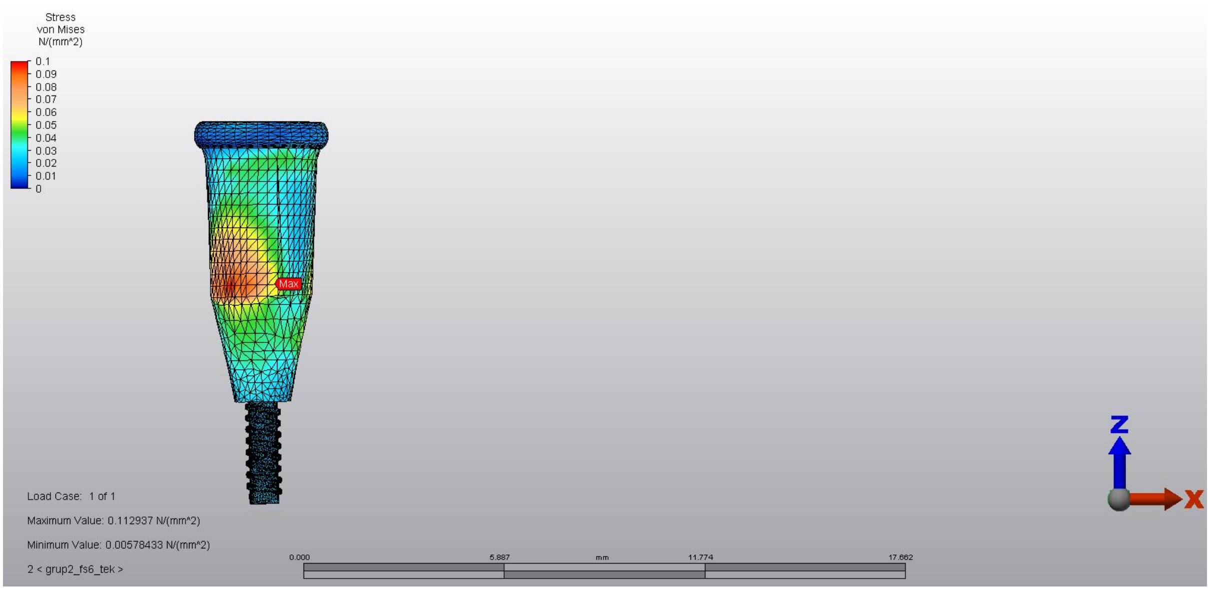
Stress
von Mises
N/(mm^2)
0.1
0.09
0.08
0.07
0.06
0.05
0.04
0.03
0.02
0.01
0
Max
Load Case: 1 of 1
Maximum Value: 0.112937 N/(mm^2)
Minimum Value: 0.00578433 N/(mm^2)
2 < grup2_fs6_tek >
0.000
5.887
mm
11.774
17.662
Z
X

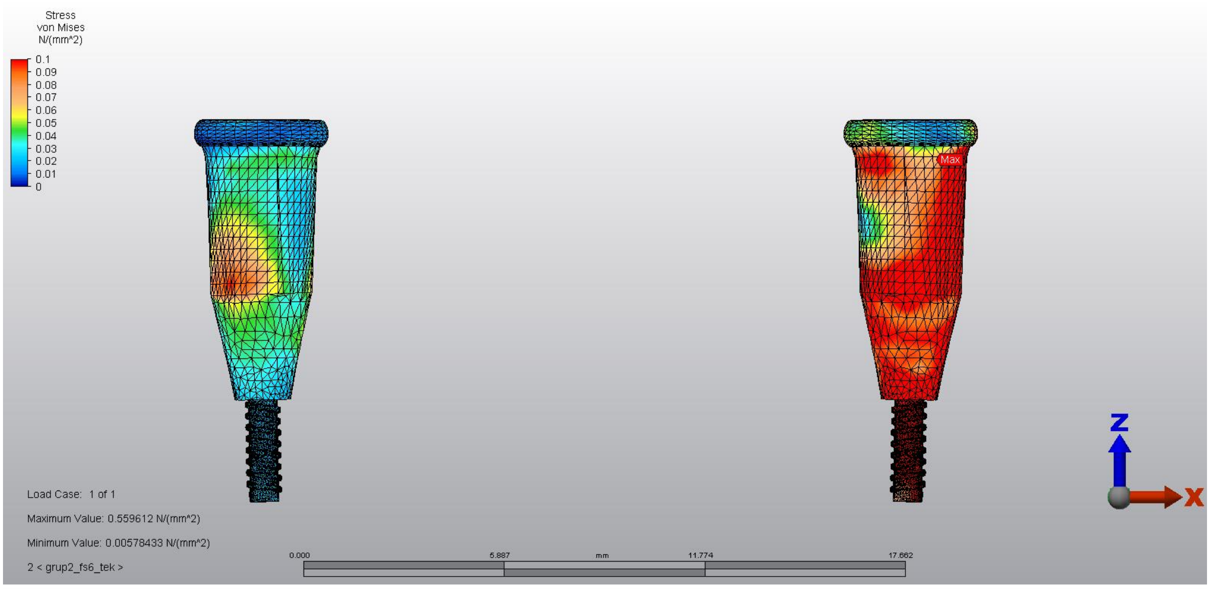

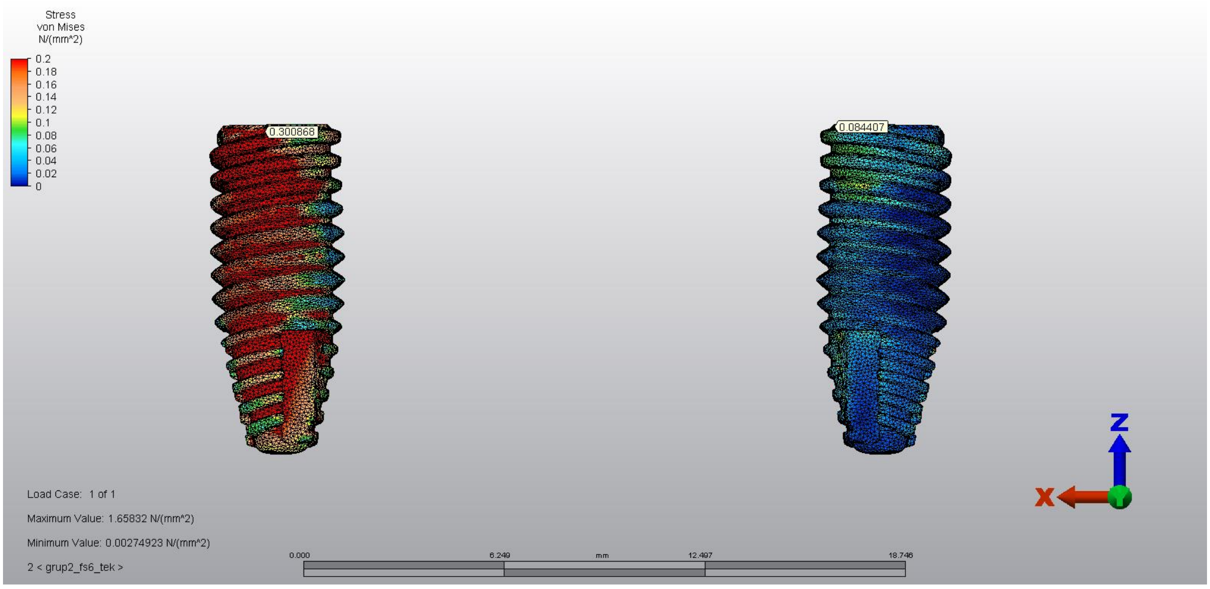

BILATERAL

Number of nodes = 136885

Number of elements = 720859

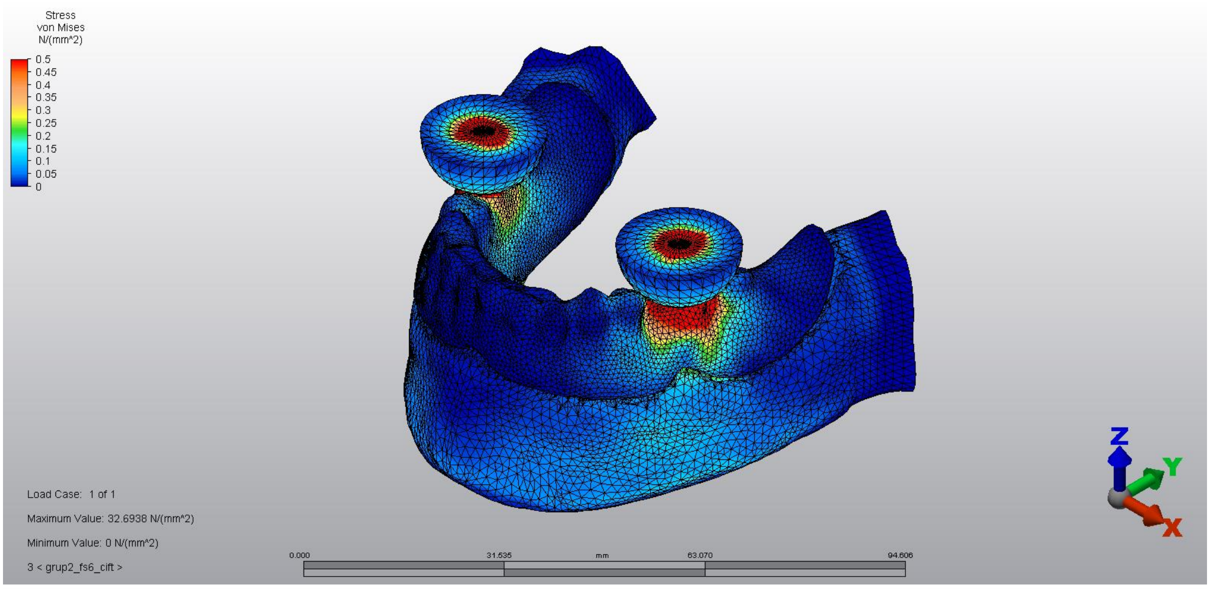
Stress
von Mises
N/(mm^2)
0.5
0.45
0.4
0.35
0.3
0.25
0.2
0.15
0.1
0.05
0
Load Case: 1 of 1
Maximum Value: 32.6938 N/(mm^2)
Minimum Value: 0 N/(mm^2)
3 < grup2_fs6_cift >
0.000
31.535
mm
63.070
94.606
Z
Y
X

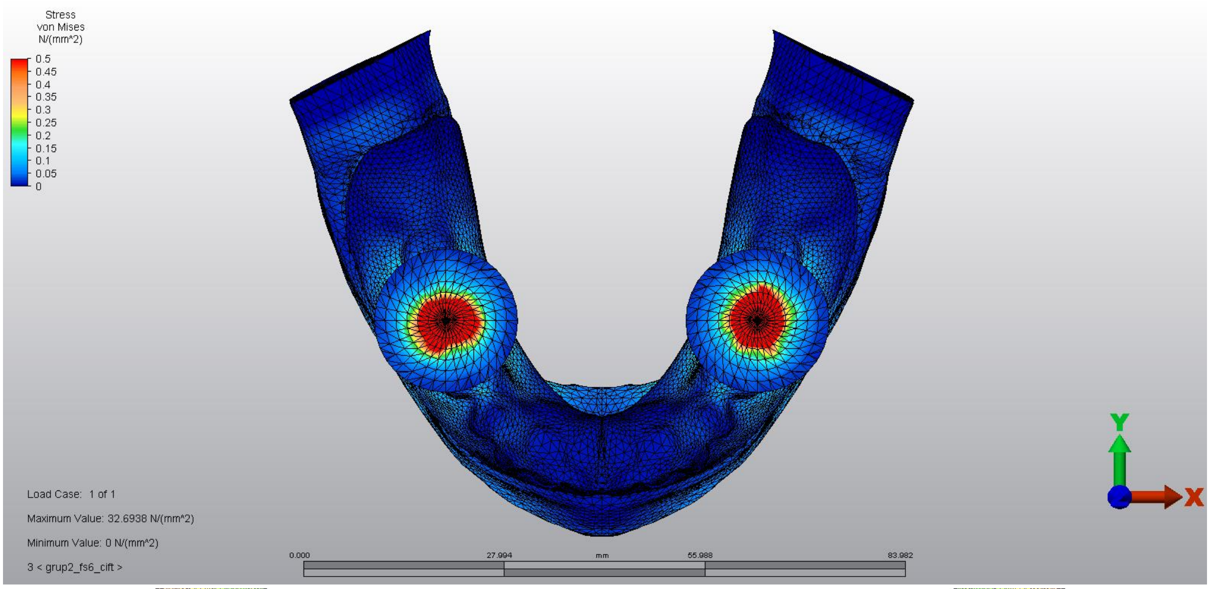
Stress
von Mises
N/(mm^2)
0.5
0.45
0.4
0.35
0.3
0.25
0.2
0.15
0.1
0.05
0
Load Case: 1 of 1
Maximum Value: 32.6938 N/(mm^2)
Minimum Value: 0 N/(mm^2)
3 < grup2_fs6_cift >
0.000
27.994
mm
55.988
83.982
Y
X

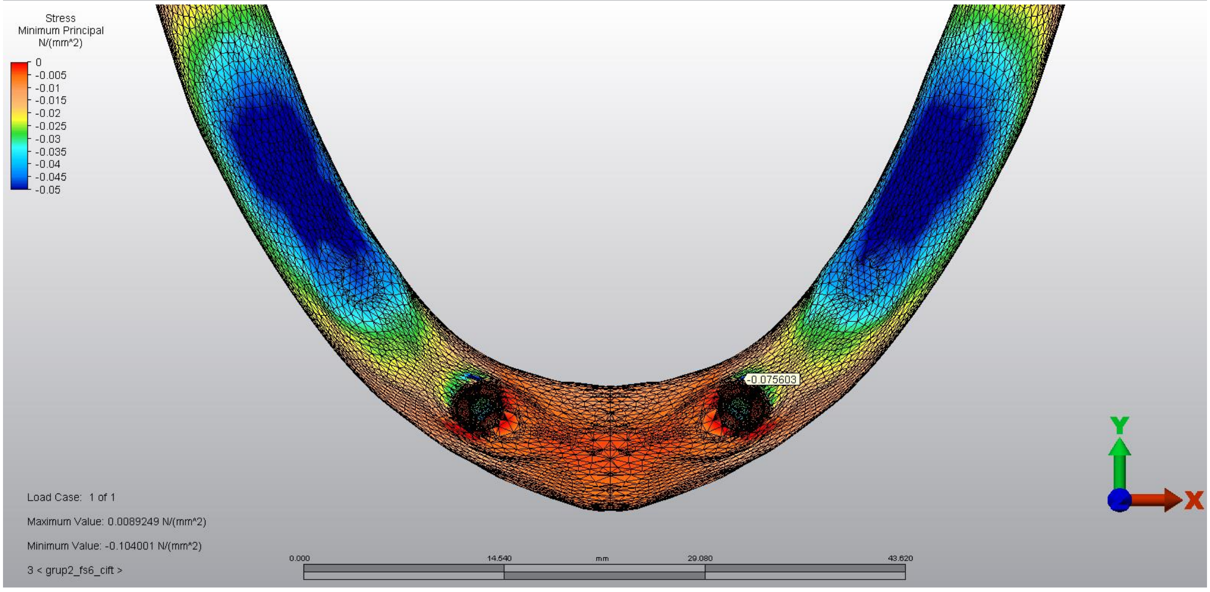
Stress
Minimum Principal
N/(mm^2)
0
-0.005
-0.01
-0.015
-0.02
-0.025
-0.03
-0.035
-0.04
-0.045
-0.05
-0.075603
Load Case: 1 of 1
Maximum Value: 0.0089249 N/(mm^2)
Minimum Value: -0.104001 N/(mm^2)
3 < grup2_fs6_cift >
0.000
14.540
mm
29.080
43.620
Y
X

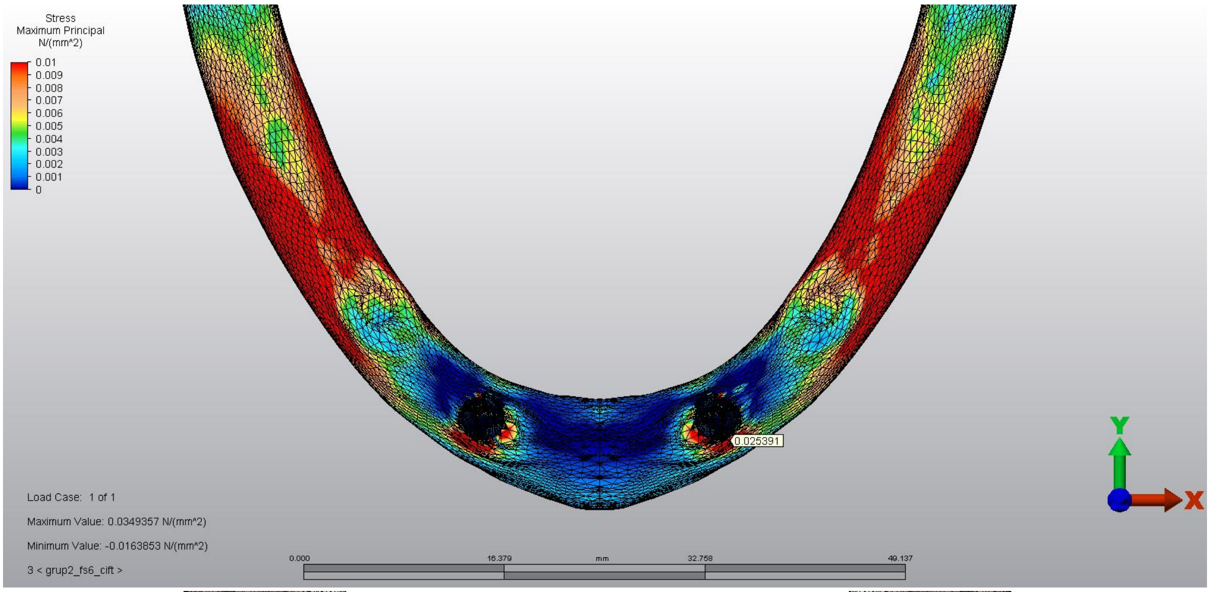
Stress
Maximum Principal
N/(mm^2)
0.01
0.009
0.008
0.007
0.006
0.005
0.004
0.003
0.002
0.001
0
0.025391
Load Case: 1 of 1
Maximum Value: 0.0349357 N/(mm^2)
Minimum Value: -0.0163853 N/(mm^2)
3 < grup2_fs6_cift >
0.000
16.379
mm
32.758
49.137
Y
X

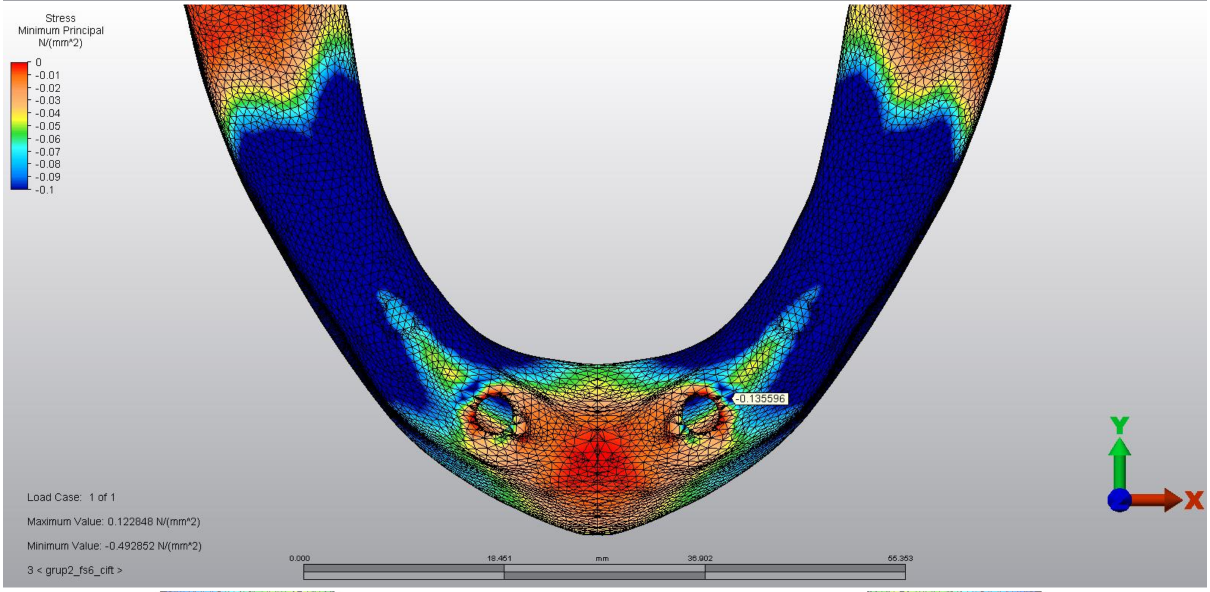
Stress
Minimum Principal
N/(mm^2)
0
-0.01
-0.02
-0.03
-0.04
-0.05
-0.06
-0.07
-0.08
-0.09
-0.1
-0.135596
Load Case: 1 of 1
Maximum Value: 0.122848 N/(mm^2)
Minimum Value: -0.492852 N/(mm^2)
3 < grup2_fs6_cift >
0.000
18.451
mm
36.902
55.353
Y
X

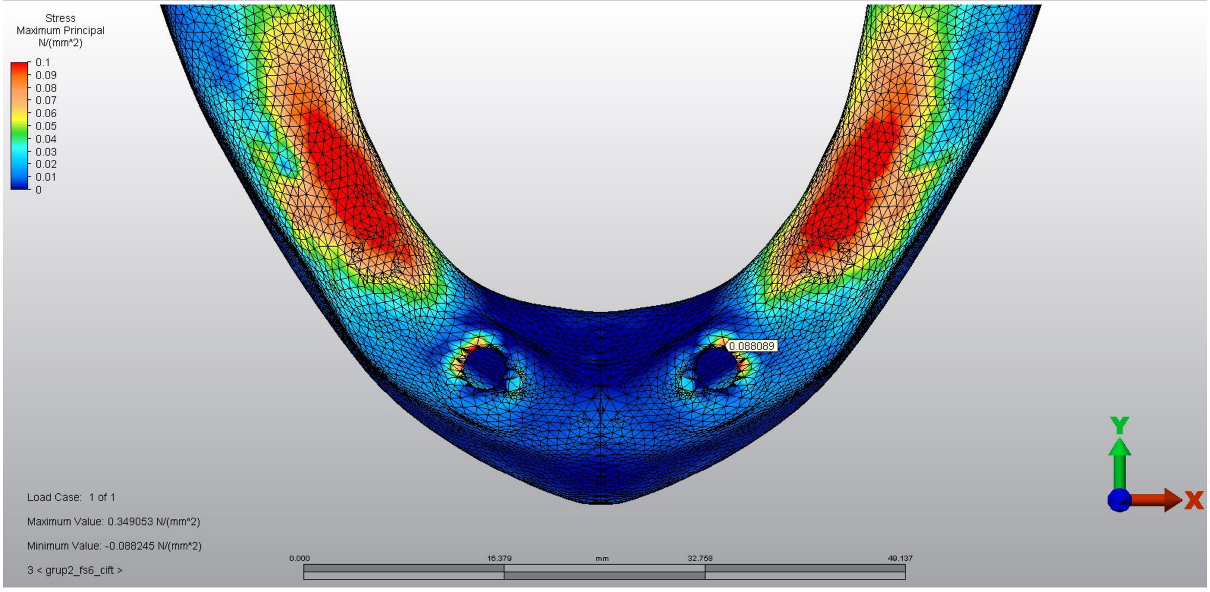
Stress
Maximum Principal
N/(mm^2)
0.1
0.09
0.08
0.07
0.06
0.05
0.04
0.03
0.02
0.01
0
0.088089
Load Case: 1 of 1
Maximum Value: 0.349053 N/(mm^2)
Minimum Value: -0.088245 N/(mm^2)
3 < grup2_fs6_cift >
0.000
16.379
mm
32.758
49.137
Y
X

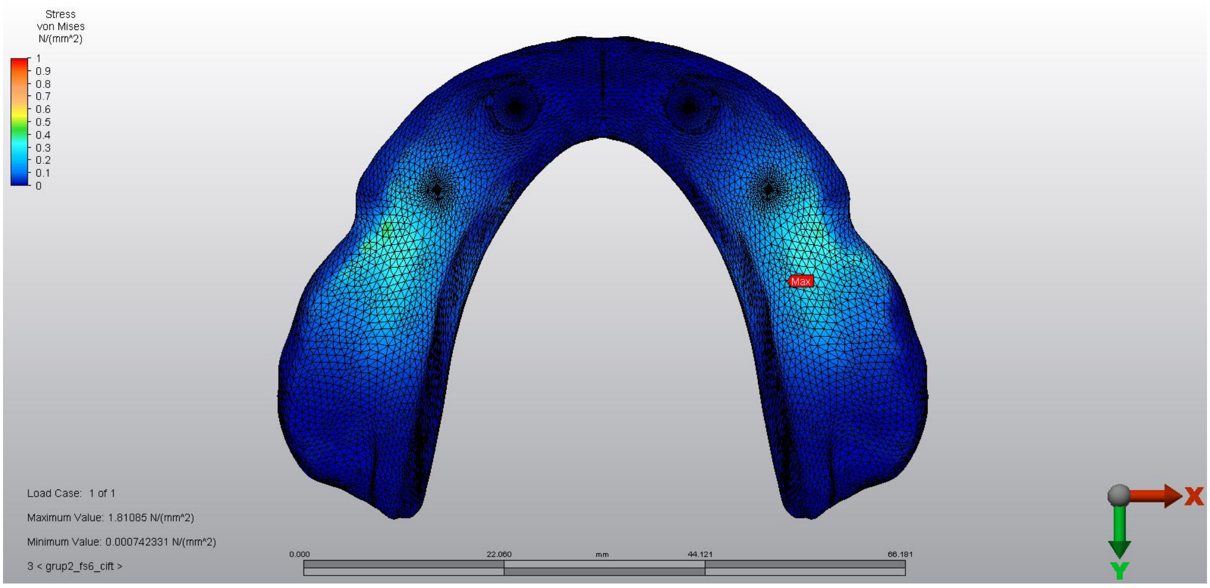
Stress
von Mises
N/(mm^2)
1
0.9
0.8
0.7
0.6
0.5
0.4
0.3
0.2
0.1
0
Max
Load Case: 1 of 1
Maximum Value: 1.81085 N/(mm^2)
Minimum Value: 0.000742331 N/(mm^2)
3 < grup2_fs6_cift >
0.000
22.060
mm
44.121
66.181
X
Y

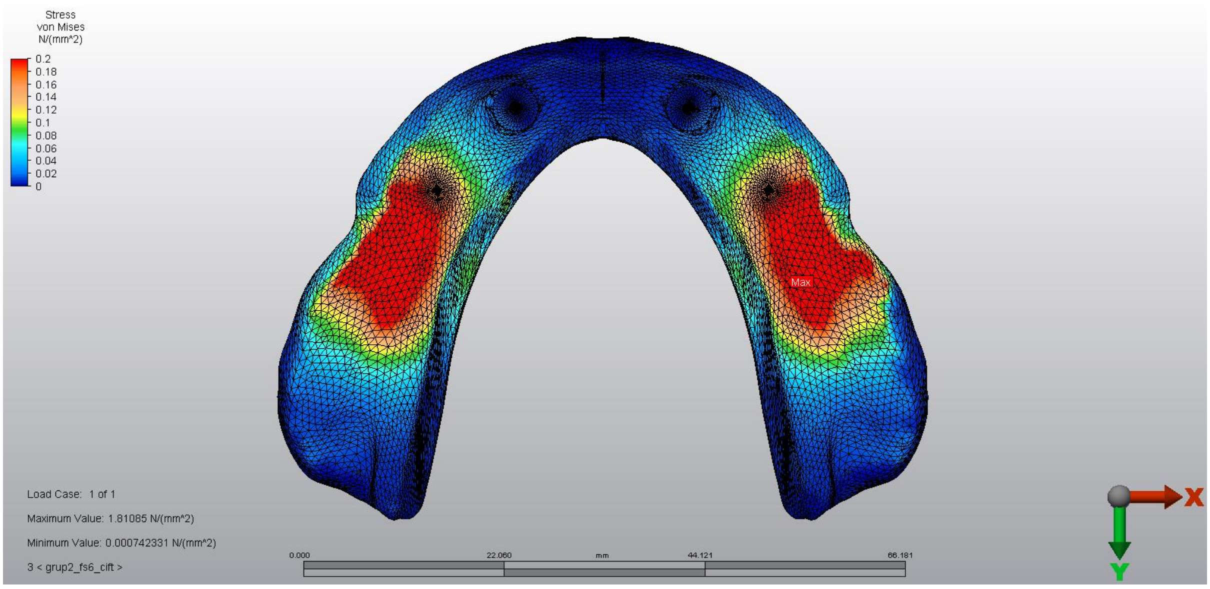
Stress
von Mises
N/(mm^2)
0.2
0.18
0.16
0.14
0.12
0.1
0.08
0.06
0.04
0.02
0
Max
Load Case: 1 of 1
Maximum Value: 1.81085 N/(mm^2)
Minimum Value: 0.000742331 N/(mm^2)
3 < grup2_fs6_cift >
0.000
22.060
mm
44.121
66.181
X
Y

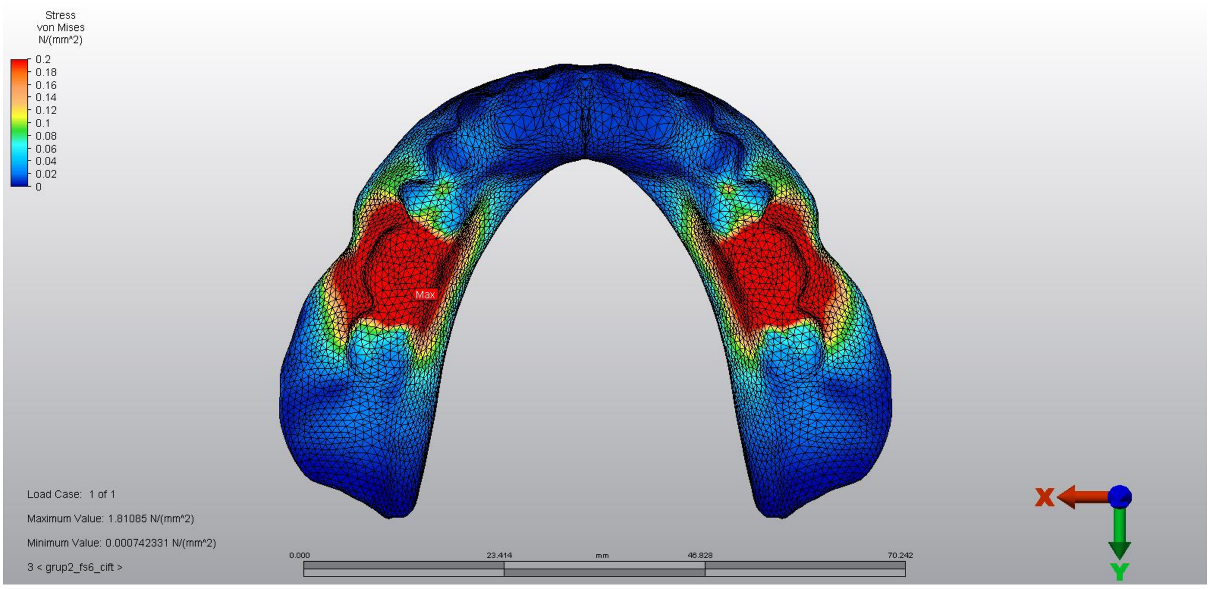
Stress
von Mises
N/(mm^2)
0.2
0.18
0.16
0.14
0.12
0.1
0.08
0.06
0.04
0.02
0
Max
Load Case: 1 of 1
Maximum Value: 1.81085 N/(mm^2)
Minimum Value: 0.000742331 N/(mm^2)
3 < grup2_fs6_cift >
0.000
23.414
mm
46.828
70.242
X
Y

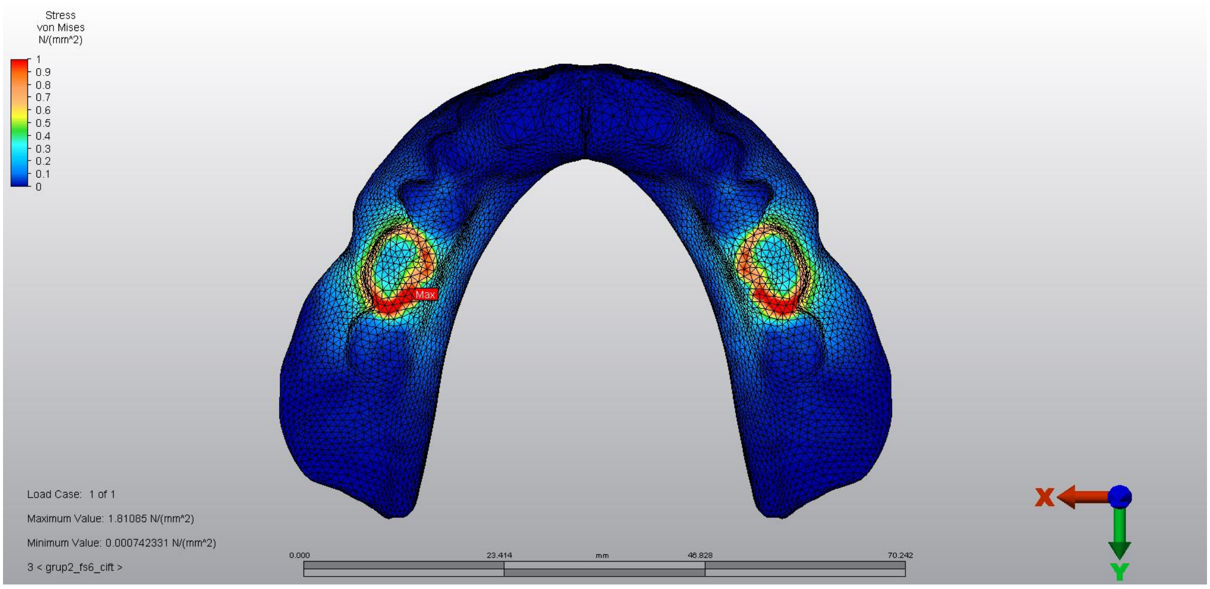
Stress
von Mises
N/(mm^2)
1
0.9
0.8
0.7
0.6
0.5
0.4
0.3
0.2
0.1
0
Max
Load Case: 1 of 1
Maximum Value: 1.81085 N/(mm^2)
Minimum Value: 0.000742331 N/(mm^2)
3 < grup2_fs6_cift >
0.000
23.414
mm
46.828
70.242
X
Y

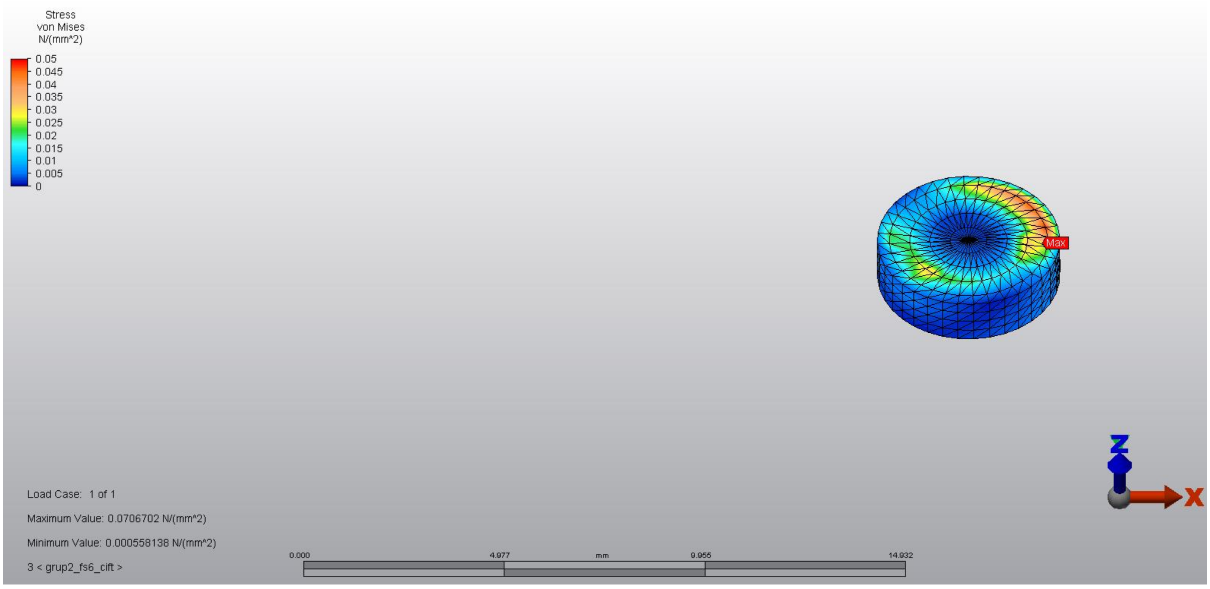
Stress
von Mises
N/(mm^2)
0.05
0.045
0.04
0.035
0.03
0.025
0.02
0.015
0.01
0.005
0
Max
Load Case: 1 of 1
Maximum Value: 0.0706702 N/(mm^2)
Minimum Value: 0.000558138 N/(mm^2)
3 < grup2_fs6_cift >
0.000
4.977
mm
9.955
14.932
Z
X

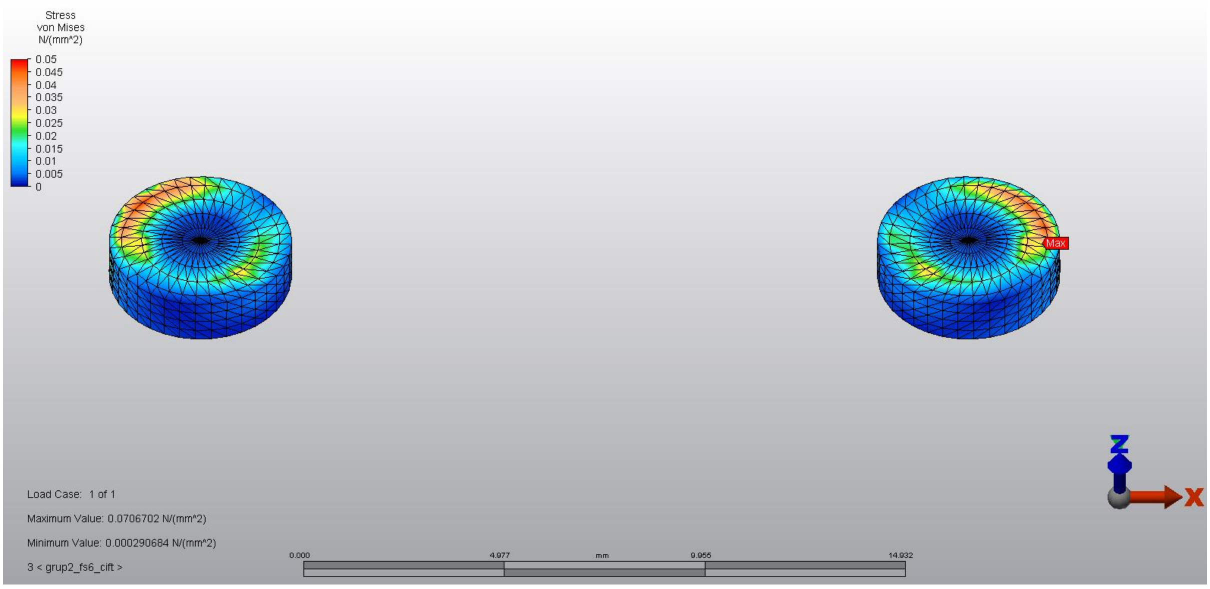
Stress
von Mises
N/(mm^2)
0.05
0.045
0.04
0.035
0.03
0.025
0.02
0.015
0.01
0.005
0
Max
Load Case: 1 of 1
Maximum Value: 0.0706702 N/(mm^2)
Minimum Value: 0.000290684 N/(mm^2)
3 < grup2_fs6_cift >
0.000
4.977
mm
9.955
14.932
Z
X

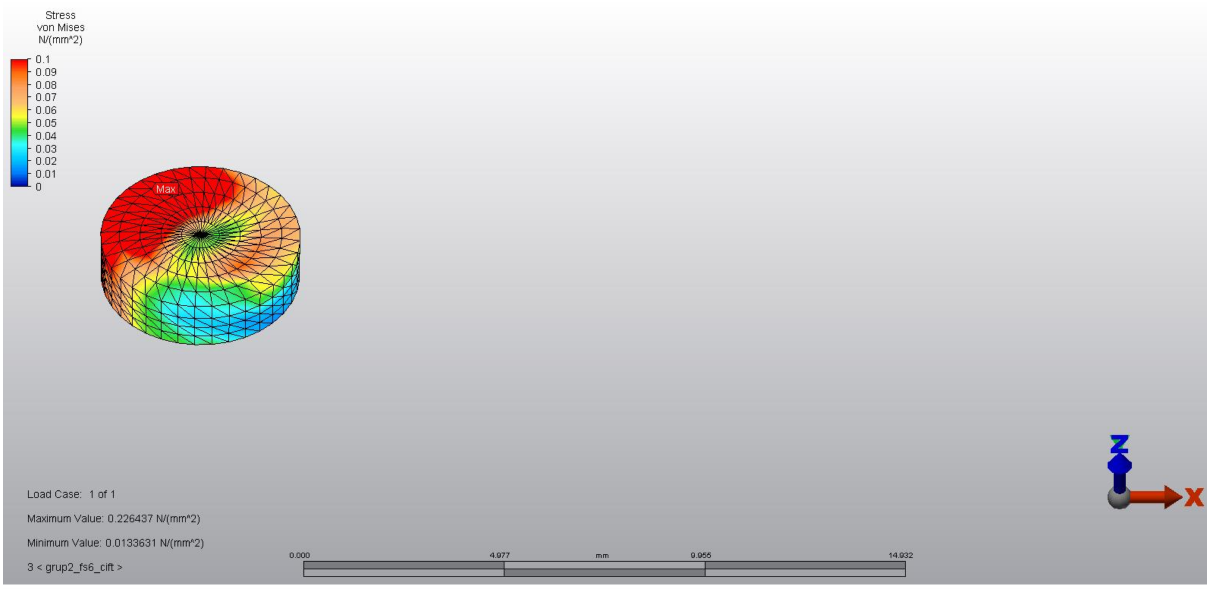
Stress
von Mises
N/(mm^2)
0.1
0.09
0.08
0.07
0.06
0.05
0.04
0.03
0.02
0.01
0
Max
Load Case: 1 of 1
Maximum Value: 0.226437 N/(mm^2)
Minimum Value: 0.0133631 N/(mm^2)
3 < grup2_fs6_cift >
0.000
4.977
mm
9.955
14.932
Z
X

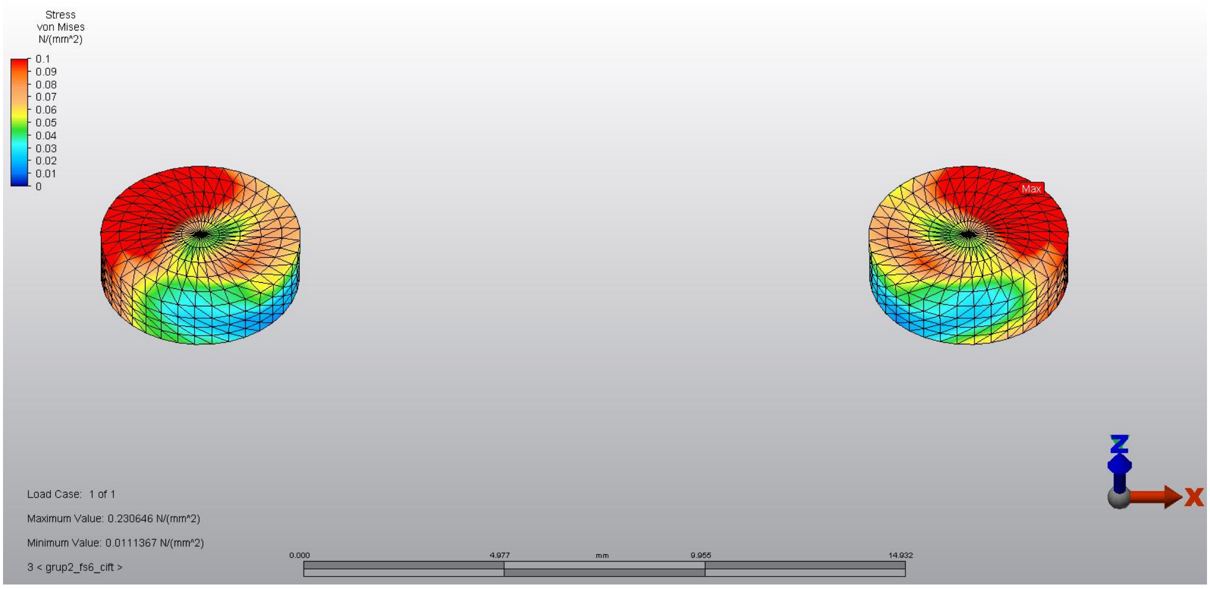
Stress
von Mises
N/(mm^2)
0.1
0.09
0.08
0.07
0.06
0.05
0.04
0.03
0.02
0.01
0
Max
Load Case: 1 of 1
Maximum Value: 0.230646 N/(mm^2)
Minimum Value: 0.0111367 N/(mm^2)
3 < grup2_fs6_cift >
0.000
4.977
mm
9.955
14.932
Z
X

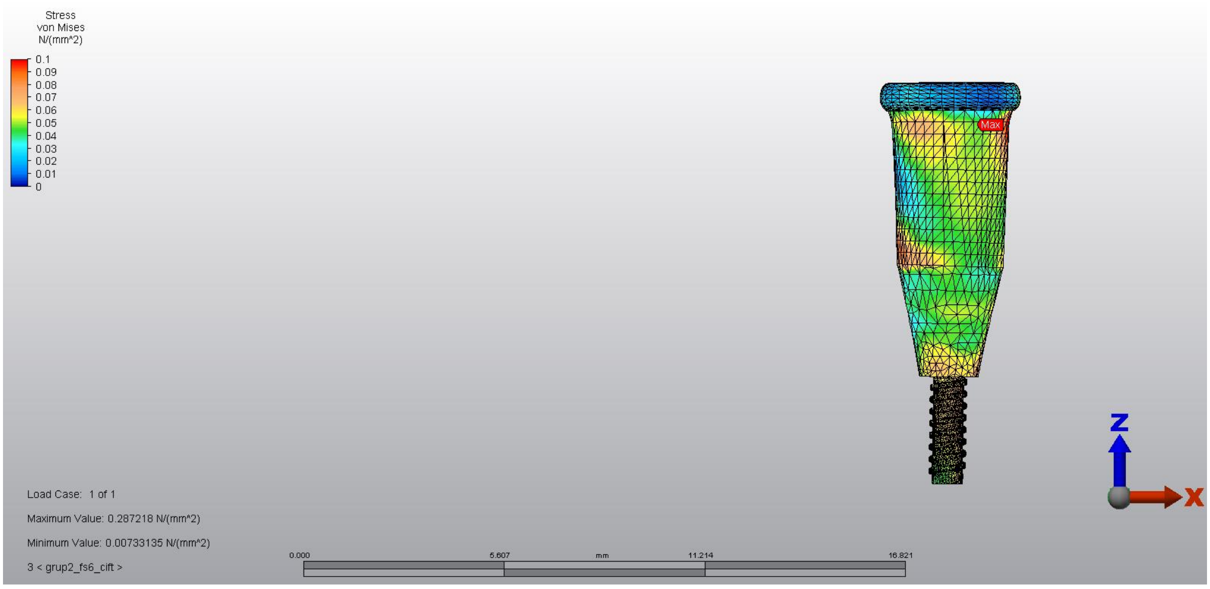
Stress
von Mises
N/(mm^2)
0.1
0.09
0.08
0.07
0.06
0.05
0.04
0.03
0.02
0.01
0
Max
Load Case: 1 of 1
Maximum Value: 0.287218 N/(mm^2)
Minimum Value: 0.00733135 N/(mm^2)
3 < grup2_fs6_cift >
0.000
5.607
mm
11.214
16.821
Z
X

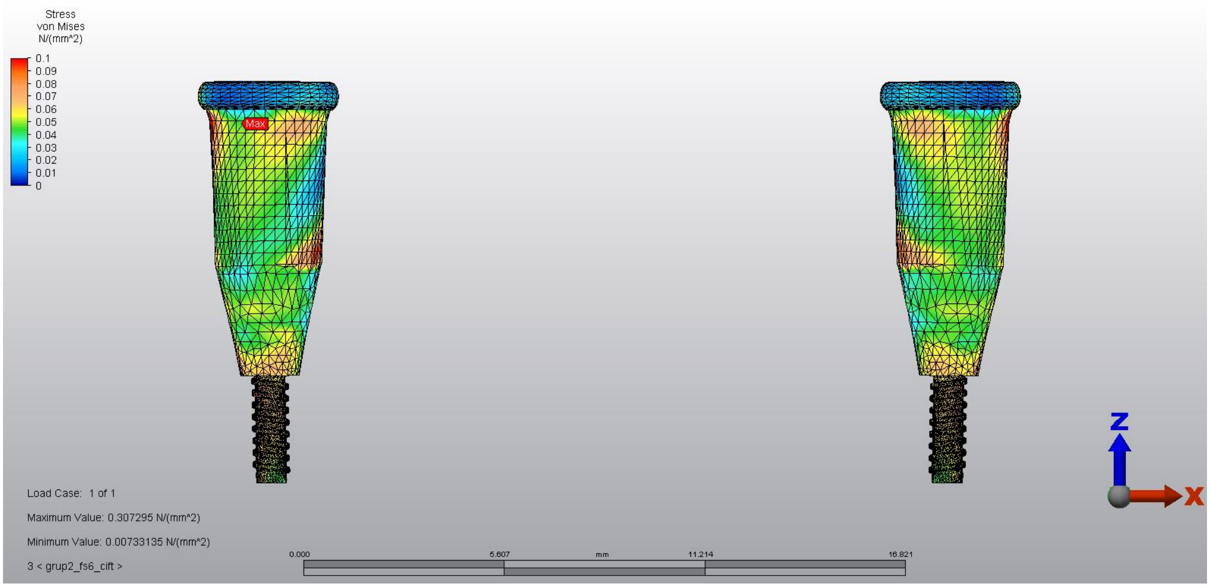

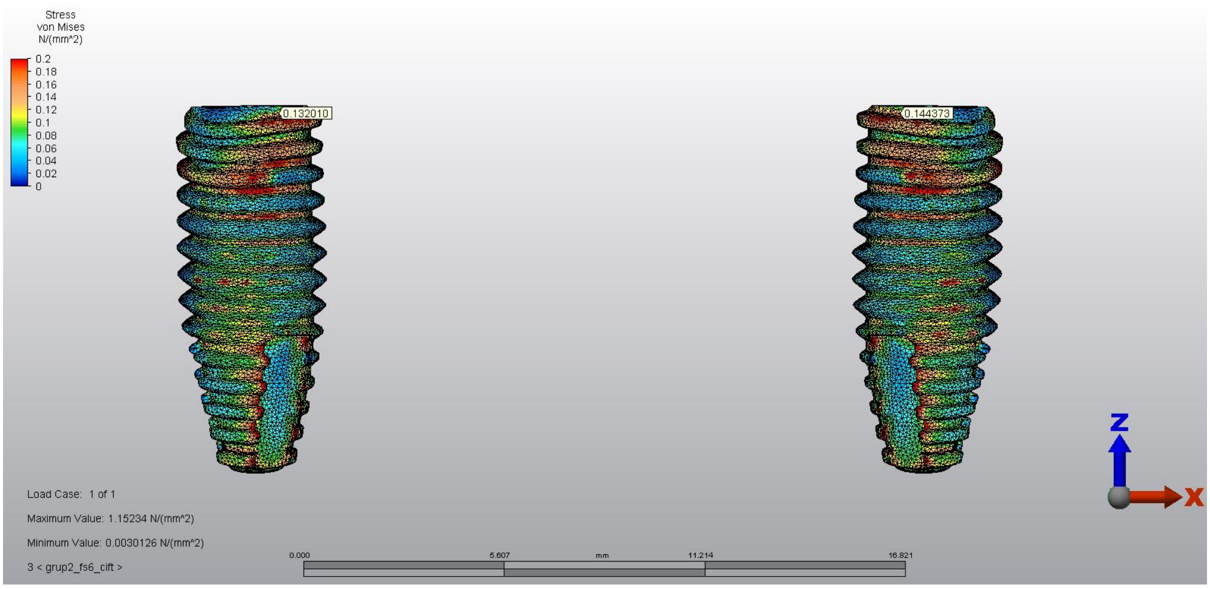

GRUP 03

INCISAL

Number of nodes = 143790

Number of elements = 722586

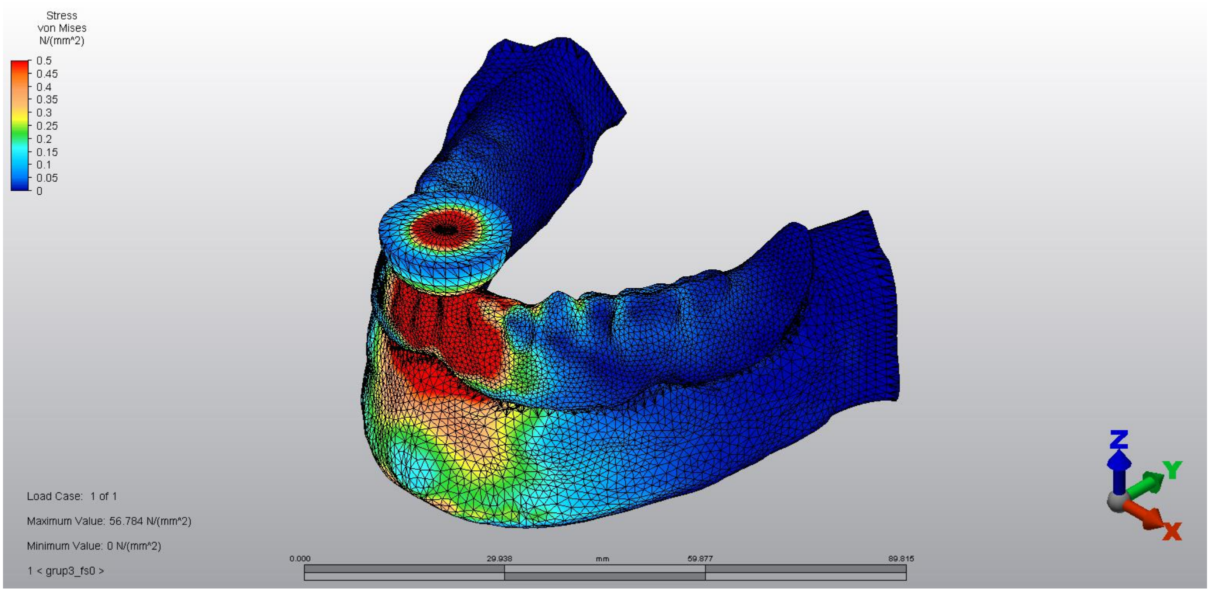
Stress
von Mises
N/(mm^2)
0.5
0.45
0.4
0.35
0.3
0.25
0.2
0.15
0.1
0.05
0
Load Case: 1 of 1
Maximum Value: 56.784 N/(mm^2)
Minimum Value: 0 N/(mm^2)
1 < grup3_fs0 >
0.000
29.938
mm
59.877
89.815
Z
Y
X

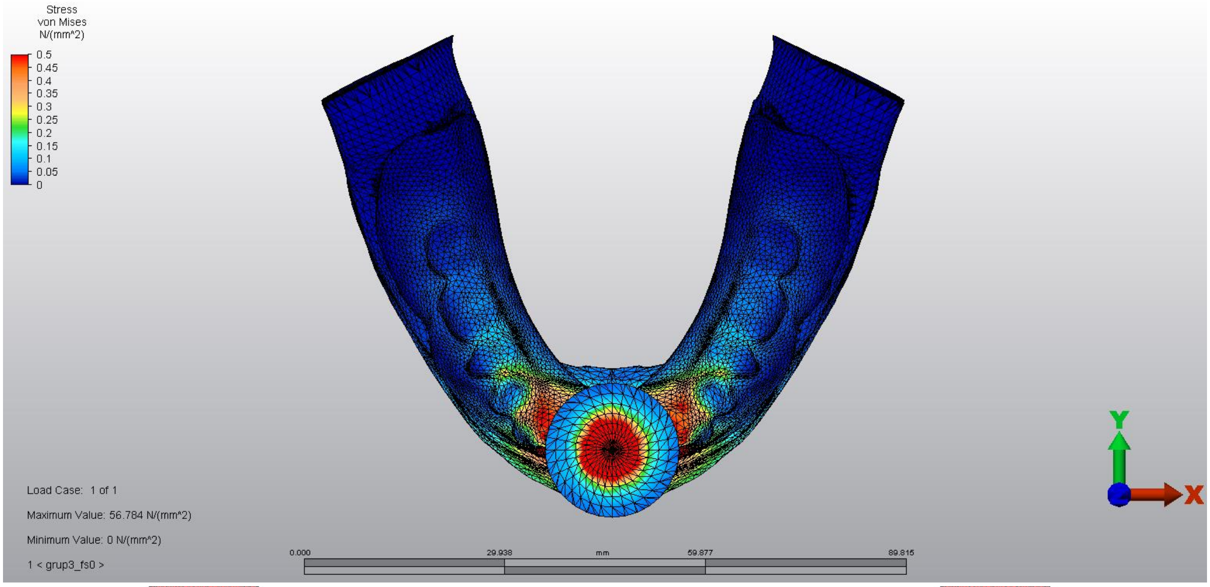
Stress
von Mises
N/(mm^2)
0.5
0.45
0.4
0.35
0.3
0.25
0.2
0.15
0.1
0.05
0
Load Case: 1 of 1
Maximum Value: 56.784 N/(mm^2)
Minimum Value: 0 N/(mm^2)
1 < grup3_fs0 >
0.000
29.938
mm
59.877
89.815
Y
X

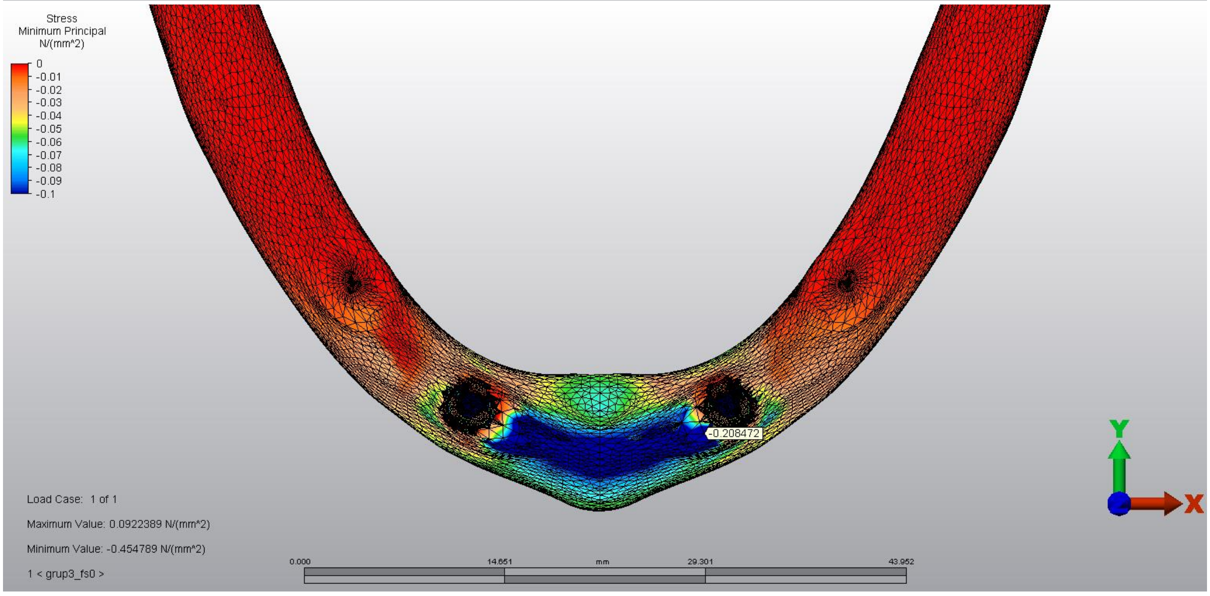
Stress
Minimum Principal
N/(mm^2)
0
-0.01
-0.02
-0.03
-0.04
-0.05
-0.06
-0.07
-0.08
-0.09
-0.1
-0.208472
Load Case: 1 of 1
Maximum Value: 0.0922389 N/(mm^2)
Minimum Value: -0.454789 N/(mm^2)
1 < grup3_fs0 >
0.000
14.651
mm
29.301
43.952
Y
X

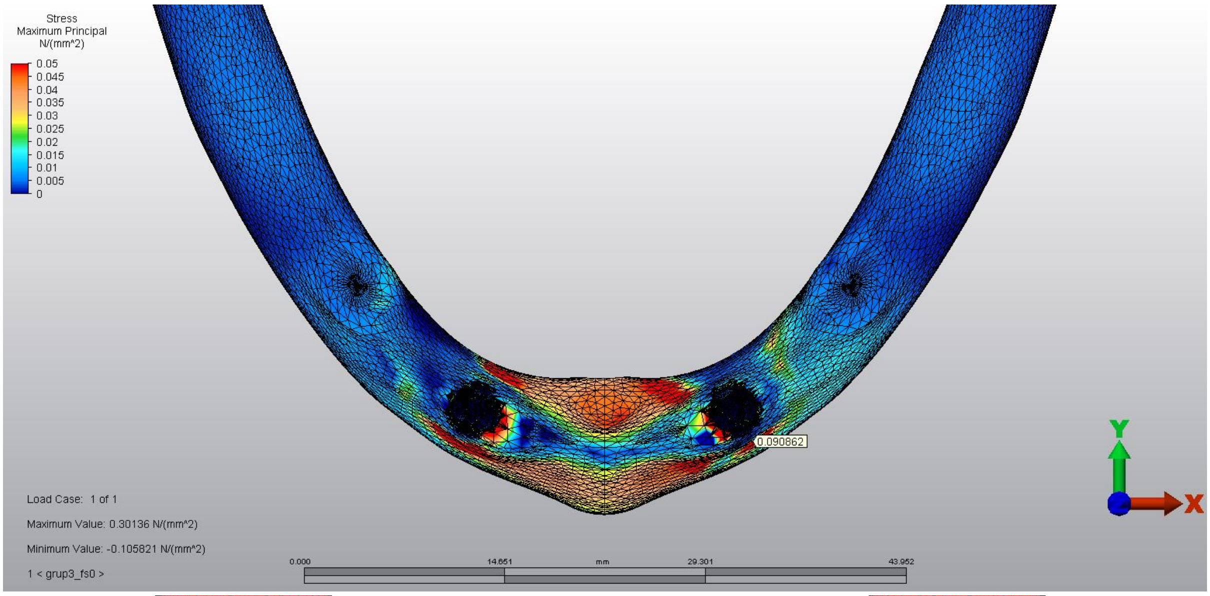
Stress
Maximum Principal
N/(mm^2)
0.05
0.045
0.04
0.035
0.03
0.025
0.02
0.015
0.01
0.005
0
0.090862
Load Case: 1 of 1
Maximum Value: 0.30136 N/(mm^2)
Minimum Value: -0.105821 N/(mm^2)
1 < grup3_fs0 >
0.000
14.651
mm
29.301
43.952
Y
X

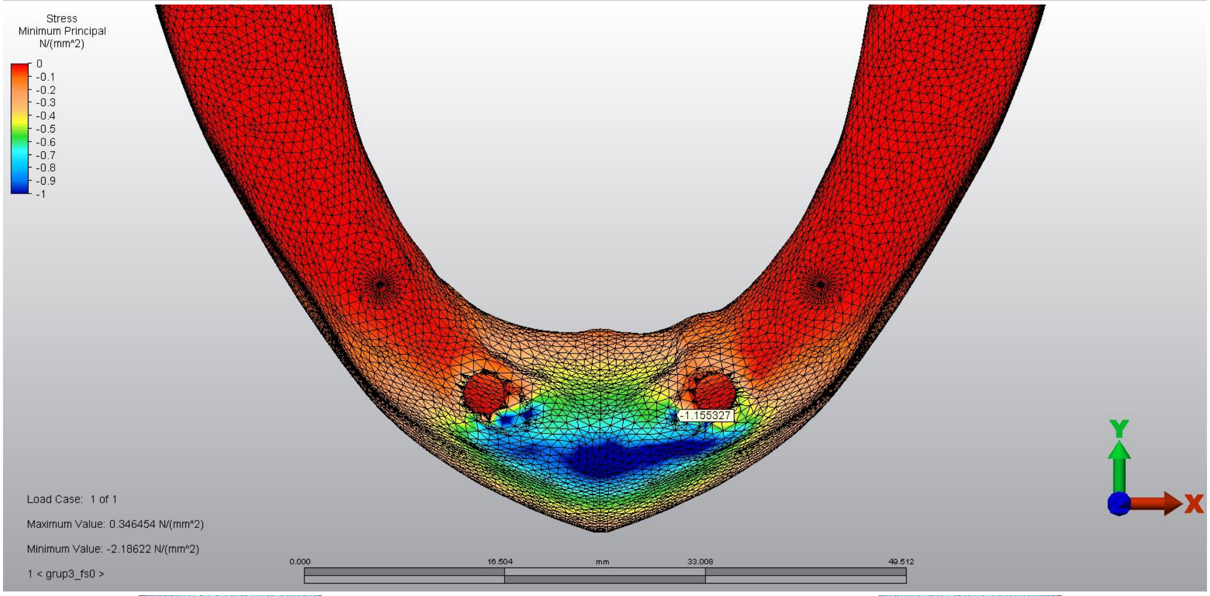
Stress
Minimum Principal
N/(mm^2)
0
-0.1
-0.2
-0.3
-0.4
-0.5
-0.6
-0.7
-0.8
-0.9
-1
-1.155327
Load Case: 1 of 1
Maximum Value: 0.346454 N/(mm^2)
Minimum Value: -2.18622 N/(mm^2)
1 < grup3_fs0 >
0.000
16.504
mm
33.008
49.512
Y
X

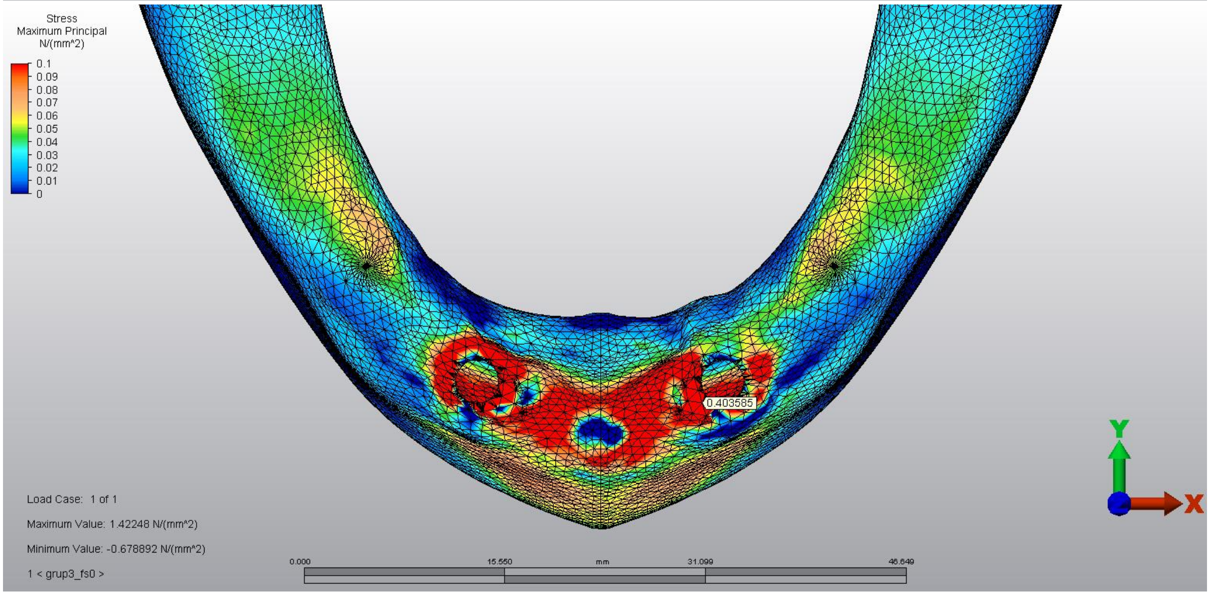
Stress
Maximum Principal
N/(mm^2)
0.1
0.09
0.08
0.07
0.06
0.05
0.04
0.03
0.02
0.01
0
0.403585
Load Case: 1 of 1
Maximum Value: 1.42248 N/(mm^2)
Minimum Value: -0.678892 N/(mm^2)
1 < grup3_fs0 >
0.000
15.550
mm
31.099
46.649
Y
X

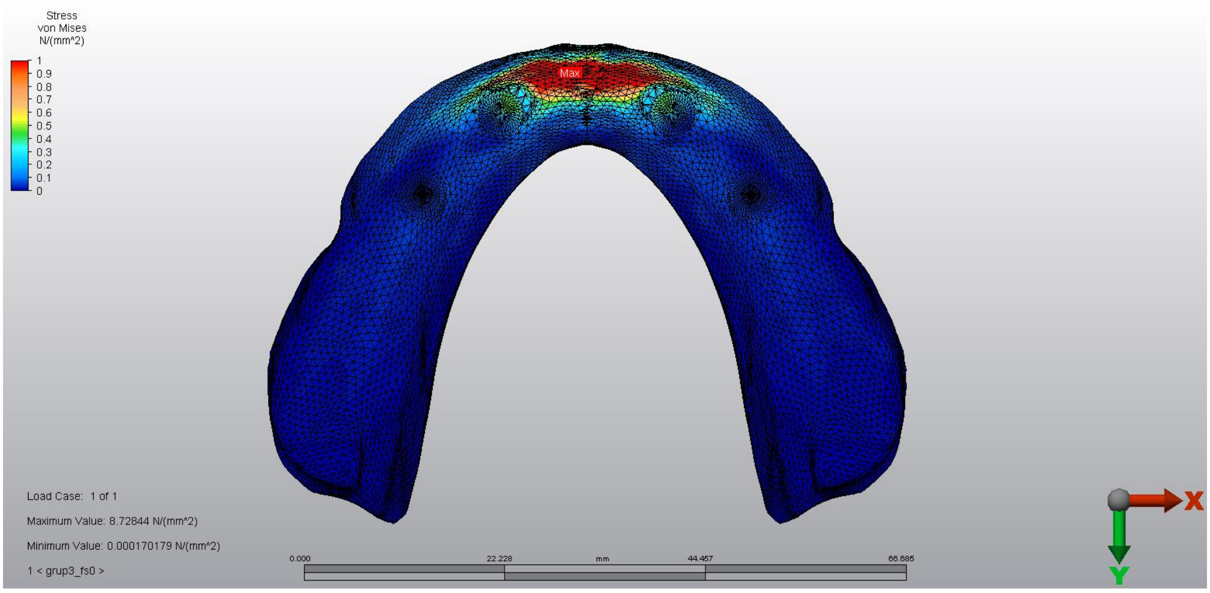
Stress
von Mises
N/(mm^2)
1
0.9
0.8
0.7
0.6
0.5
0.4
0.3
0.2
0.1
0
Max
Load Case: 1 of 1
Maximum Value: 8.72844 N/(mm^2)
Minimum Value: 0.000170179 N/(mm^2)
1 < grup3_fs0 >
0.000
22.228
mm
44.457
66.685
X
Y

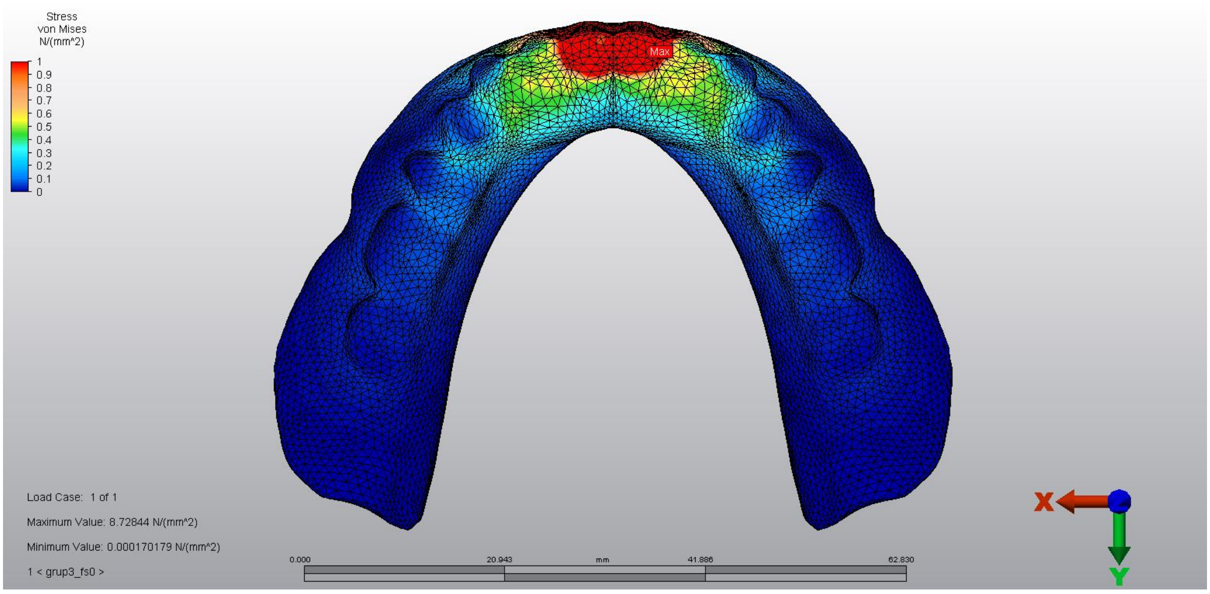
Stress
von Mises
N/(mm^2)
1
0.9
0.8
0.7
0.6
0.5
0.4
0.3
0.2
0.1
0
Max
Load Case: 1 of 1
Maximum Value: 8.72844 N/(mm^2)
Minimum Value: 0.000170179 N/(mm^2)
1 < grup3_fs0 >
0.000
20.943
mm
41.886
62.830
X
Y

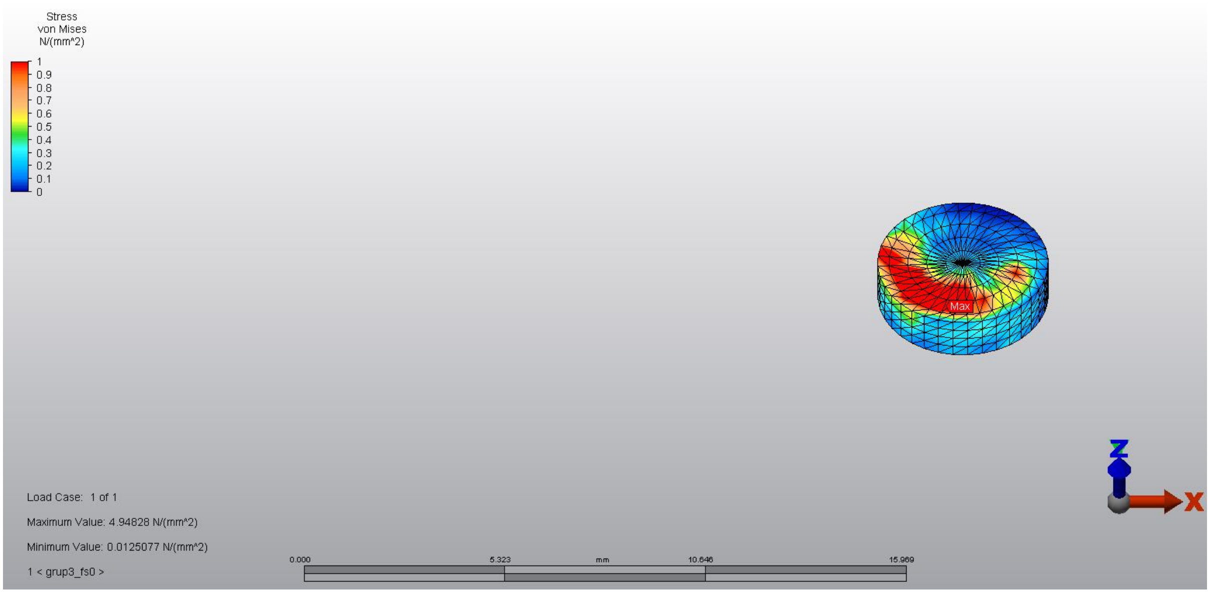
Stress
von Mises
N/(mm^2)
1
0.9
0.8
0.7
0.6
0.5
0.4
0.3
0.2
0.1
0
Max
Load Case: 1 of 1
Maximum Value: 4.94828 N/(mm^2)
Minimum Value: 0.0125077 N/(mm^2)
1 < grup3_fs0 >
0.000
5.323
mm
10.646
15.969
Z
X

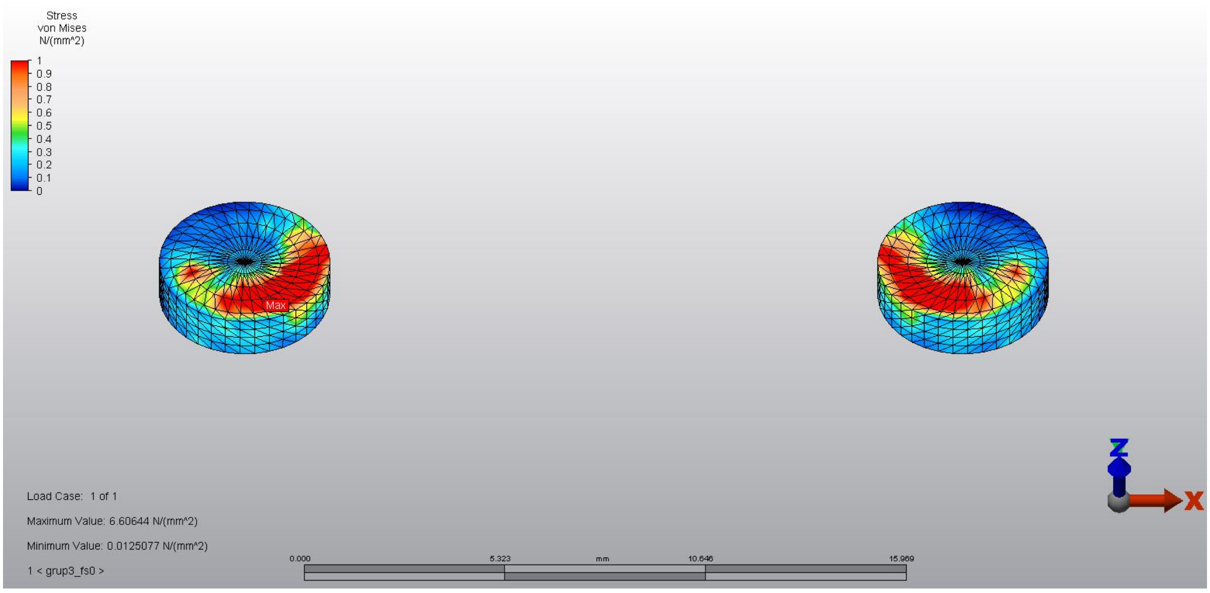
Stress
von Mises
N/(mm^2)
1
0.9
0.8
0.7
0.6
0.5
0.4
0.3
0.2
0.1
0
Max
Z
X
Load Case: 1 of 1
Maximum Value: 6.60644 N/(mm^2)
Minimum Value: 0.0125077 N/(mm^2)
1 < grup3_fs0 >
0.000
5.323
mm
10.646
15.969

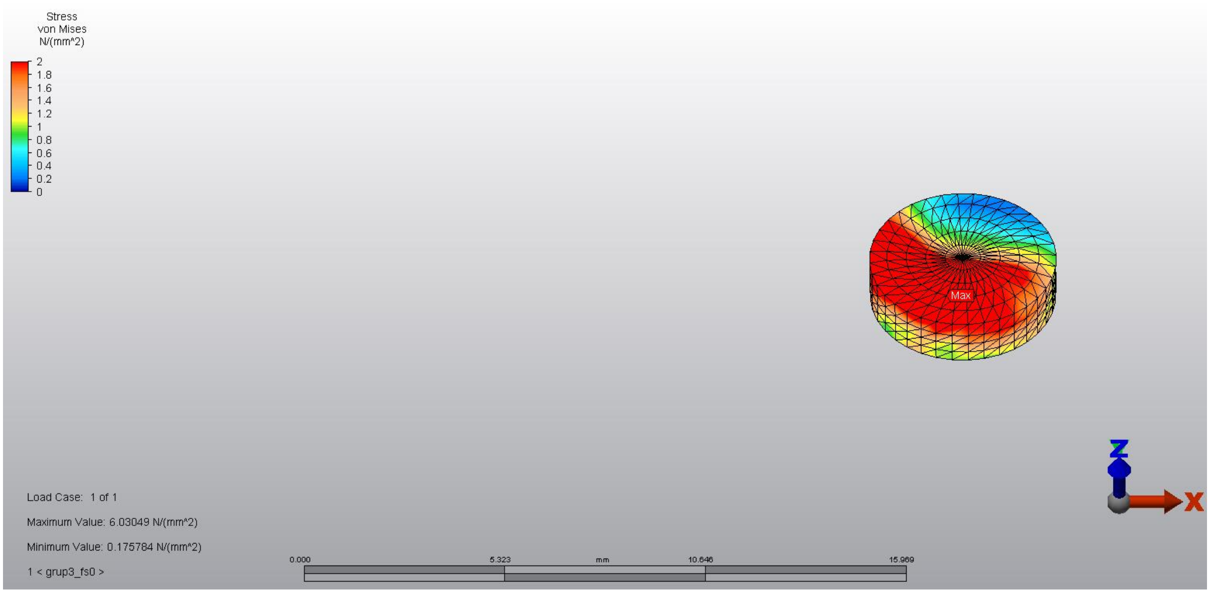
Stress
von Mises
N/(mm^2)
2
1.8
1.6
1.4
1.2
1
0.8
0.6
0.4
0.2
0
Max
Z
X
Load Case: 1 of 1
Maximum Value: 6.03049 N/(mm^2)
Minimum Value: 0.175784 N/(mm^2)
1 < grup3_fs0 >
0.000
5.323
mm
10.646
15.969

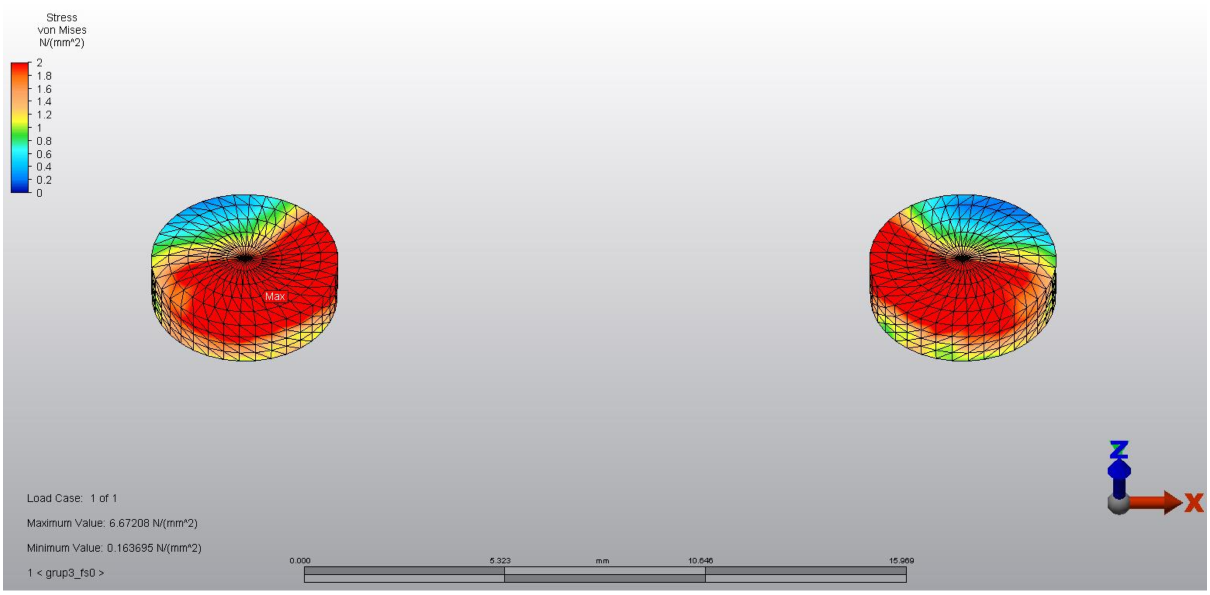
Stress
von Mises
N/(mm^2)
2
1.8
1.6
1.4
1.2
1
0.8
0.6
0.4
0.2
0
Max
Z
X
Load Case: 1 of 1
Maximum Value: 6.67208 N/(mm^2)
Minimum Value: 0.163695 N/(mm^2)
1 < grup3_fs0 >
0.000
5.323
mm
10.646
15.969

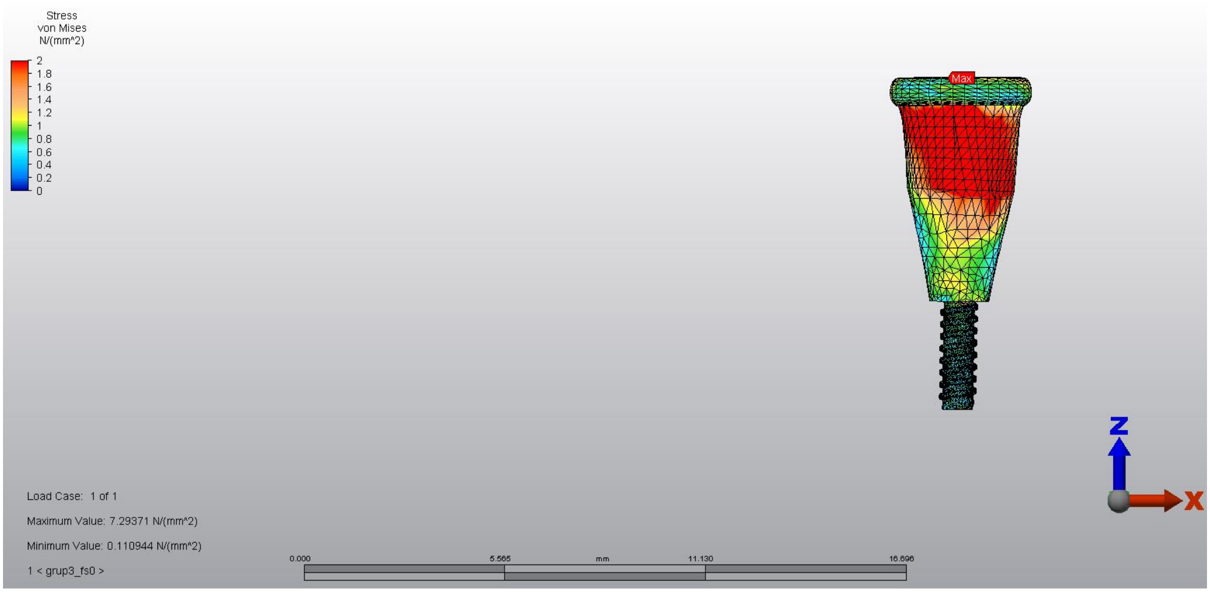
Stress
von Mises
N/(mm^2)
2
1.8
1.6
1.4
1.2
1
0.8
0.6
0.4
0.2
0
Max
Load Case: 1 of 1
Maximum Value: 7.29371 N/(mm^2)
Minimum Value: 0.110944 N/(mm^2)
1 < grup3_fs0 >
0.000
5.565
mm
11.130
16.696
Z
X

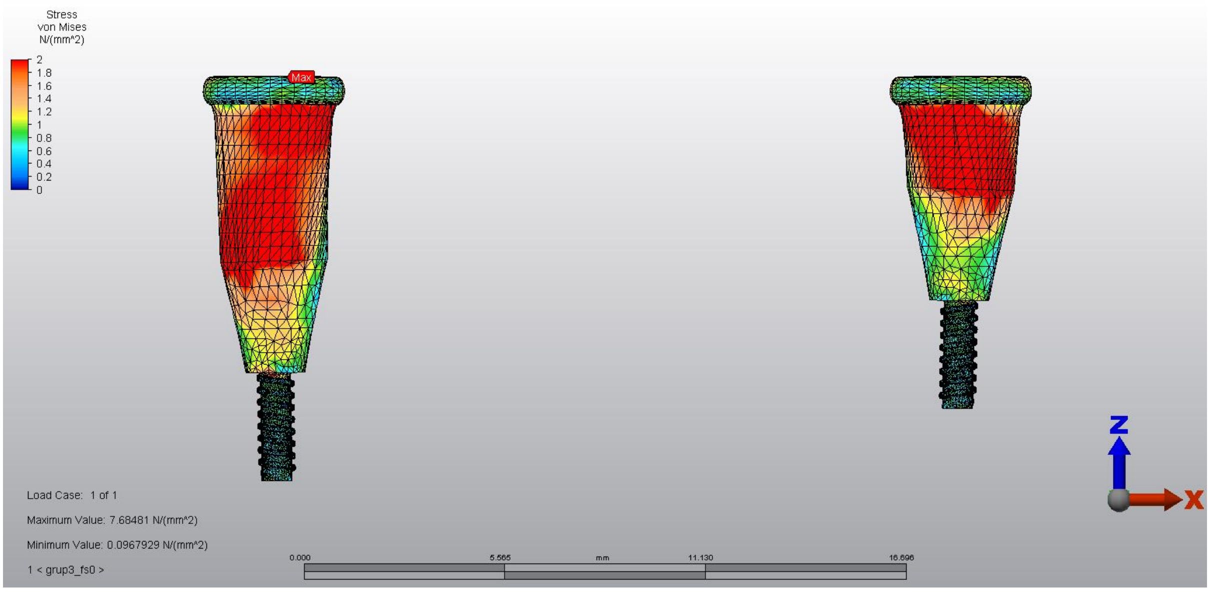
Stress
von Mises
N/(mm^2)
2
1.8
1.6
1.4
1.2
1
0.8
0.6
0.4
0.2
0
Max
Load Case: 1 of 1
Maximum Value: 7.68481 N/(mm^2)
Minimum Value: 0.0967929 N/(mm^2)
1 < grup3_fs0 >
0.000
5.565
mm
11.130
16.696
Z
X

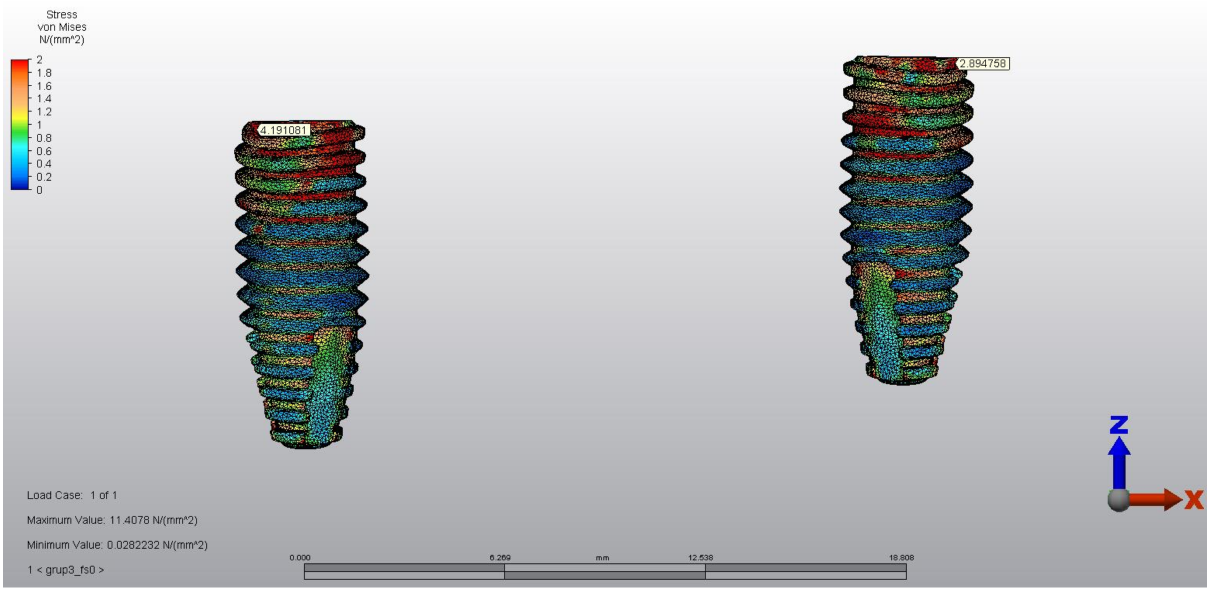
Stress
von Mises
N/(mm^2)
2
1.8
1.6
1.4
1.2
1
0.8
0.6
0.4
0.2
0
4.191081
2.894758
Load Case: 1 of 1
Maximum Value: 11.4078 N/(mm^2)
Minimum Value: 0.0282232 N/(mm^2)
1 < grup3_fs0 >
0.000
6.269
mm
12.538
18.808
Z
X

UNILATERAL

Number of nodes = 143790

Number of elements = 722734

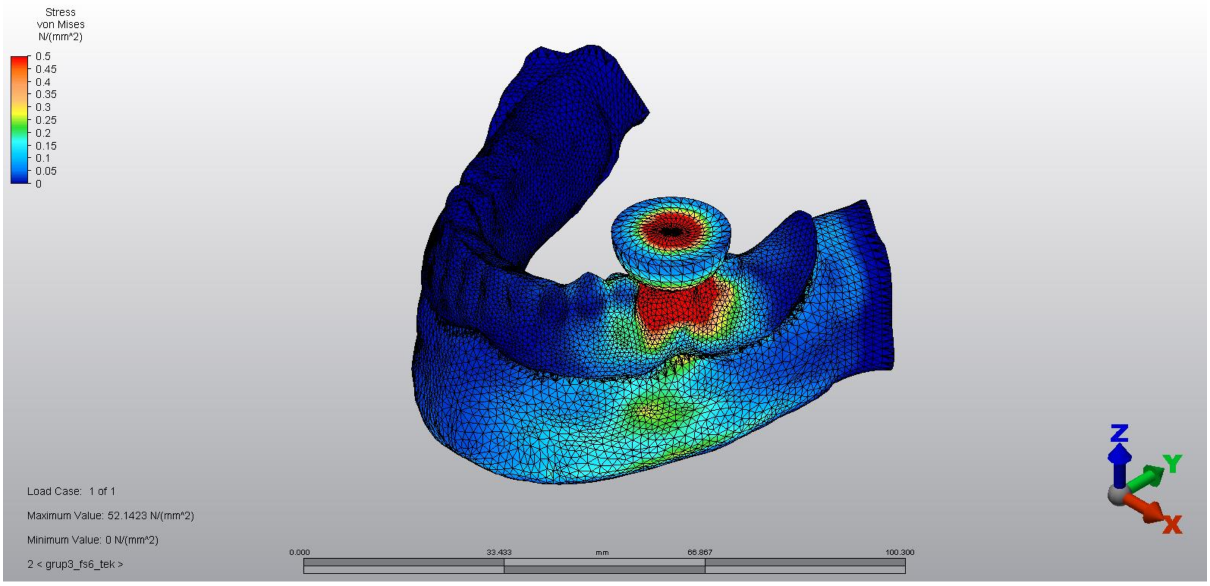

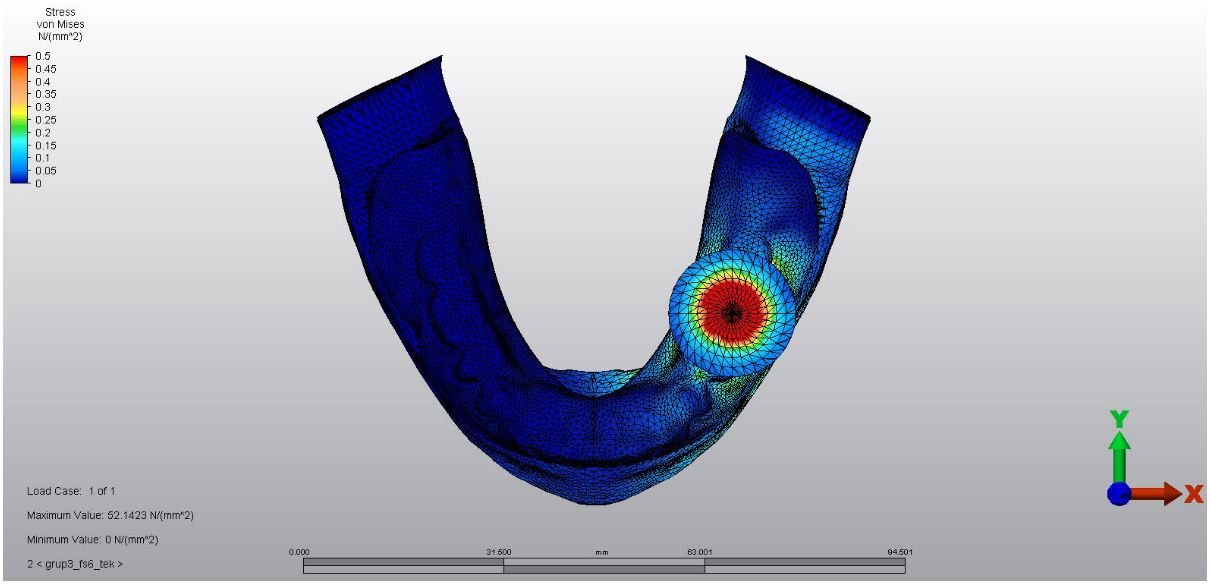

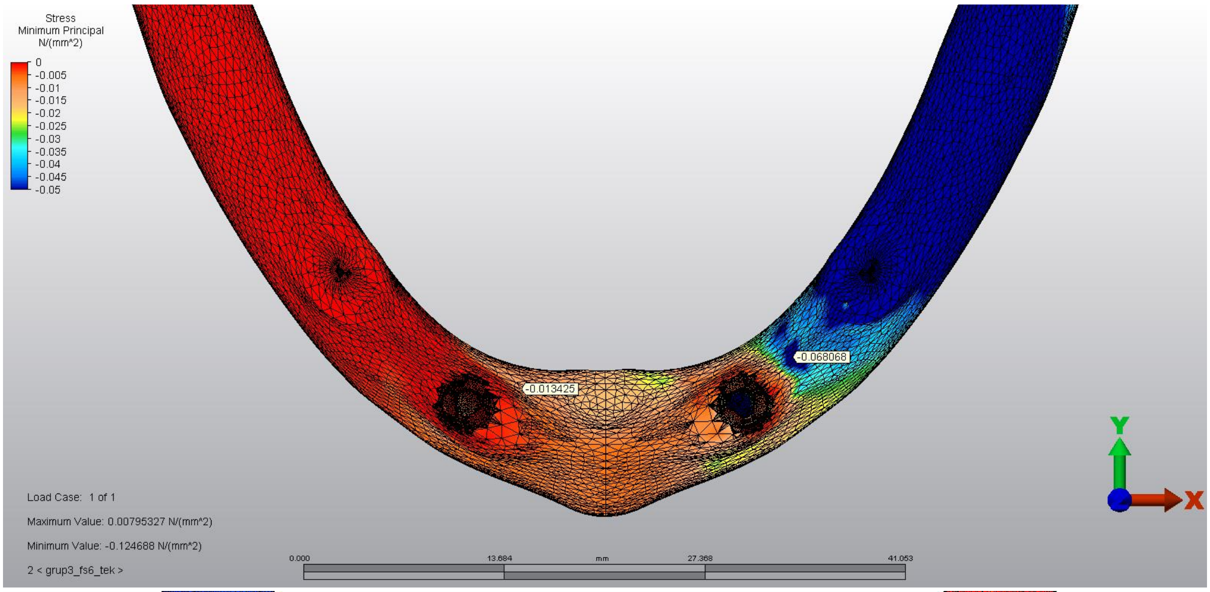

Stress
Minimum Principal
N/(mm^2)
0
-0.005
-0.01
-0.015
-0.02
-0.025
-0.03
-0.035
-0.04
-0.045
-0.05
-0.013425
-0.068068
Load Case: 1 of 1
Maximum Value: 0.00795327 N/(mm^2)
Minimum Value: -0.124688 N/(mm^2)
2 < grup3_fs6_tek >
0.000
13.684
mm
27.368
41.053
Y
X

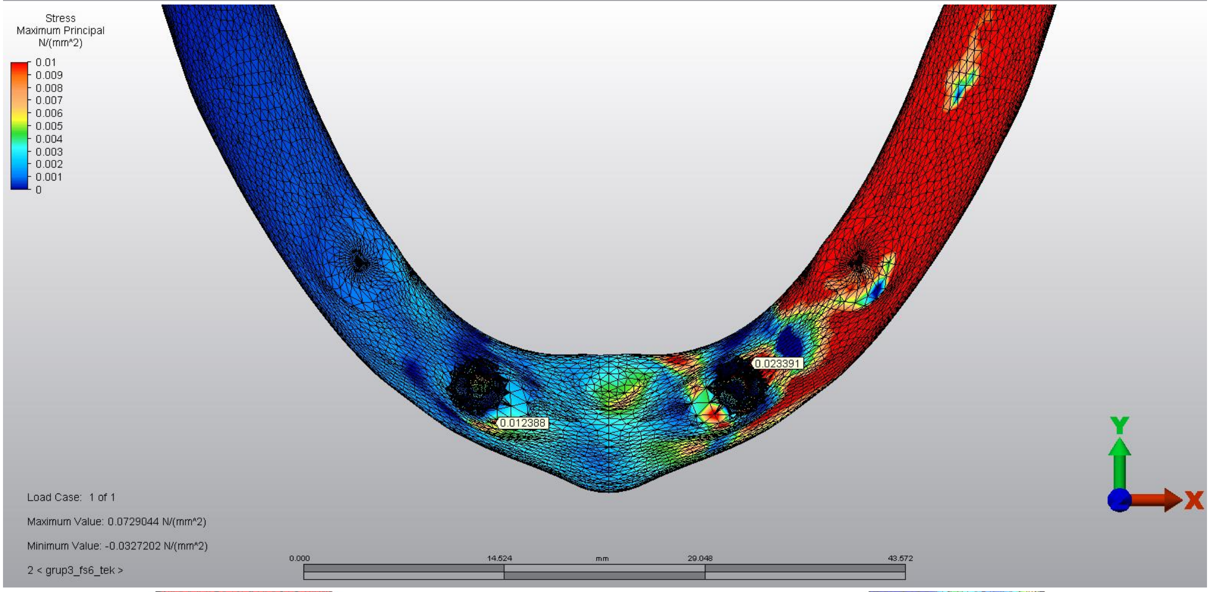

Stress
Maximum Principal
N/(mm^2)
0.01
0.009
0.008
0.007
0.006
0.005
0.004
0.003
0.002
0.001
0
0.023391
0.012388
Load Case: 1 of 1
Maximum Value: 0.0729044 N/(mm^2)
Minimum Value: -0.0327202 N/(mm^2)
2 < grup3_fs6_tek >
0.000
14.524
mm
29.048
43.572
Y
X

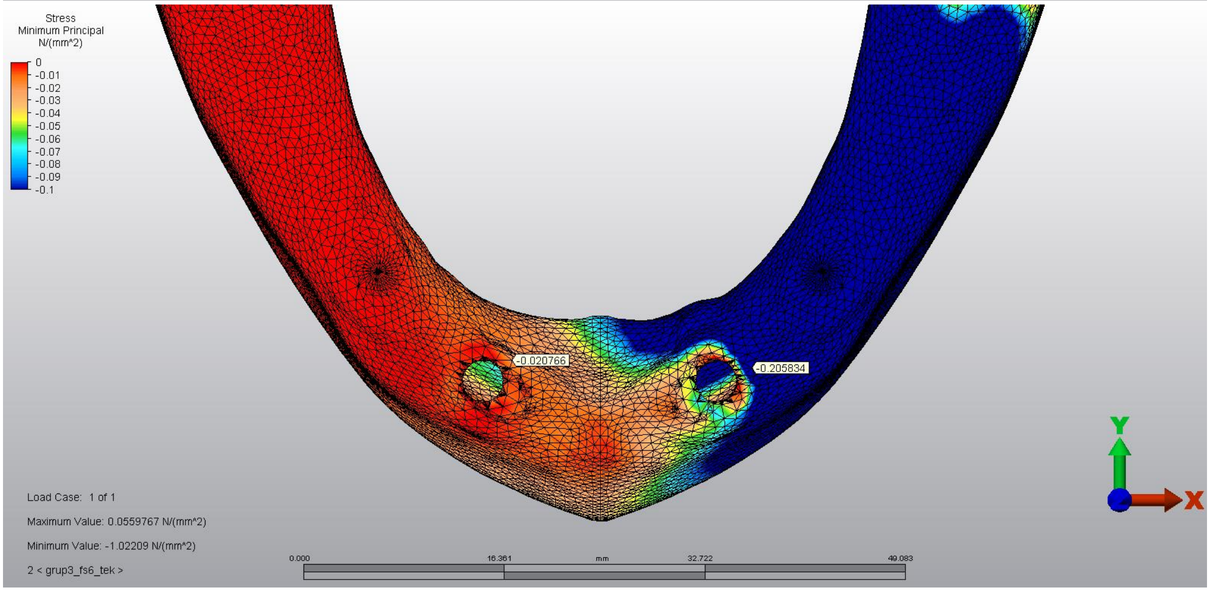

Stress
Minimum Principal
N/(mm^2)
0
-0.01
-0.02
-0.03
-0.04
-0.05
-0.06
-0.07
-0.08
-0.09
-0.1
-0.020766
-0.205834
Load Case: 1 of 1
Maximum Value: 0.0559767 N/(mm^2)
Minimum Value: -1.02209 N/(mm^2)
2 < grup3_fs6_tek >
0.000
16.361
mm
32.722
49.083
Y
X

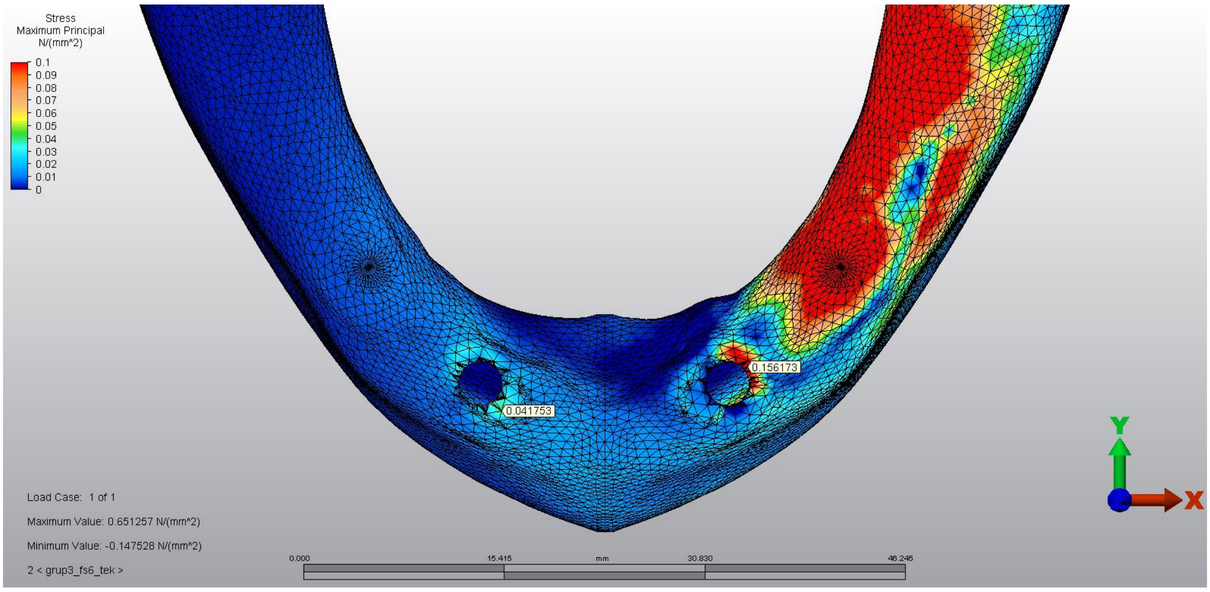
Stress
Maximum Principal
N/(mm^2)
0.1
0.09
0.08
0.07
0.06
0.05
0.04
0.03
0.02
0.01
0
0.156173
0.041753
Load Case: 1 of 1
Maximum Value: 0.651257 N/(mm^2)
Minimum Value: -0.147528 N/(mm^2)
2 < grup3_fs6_tek >
0.000
15.415
mm
30.830
46.245
Y
X

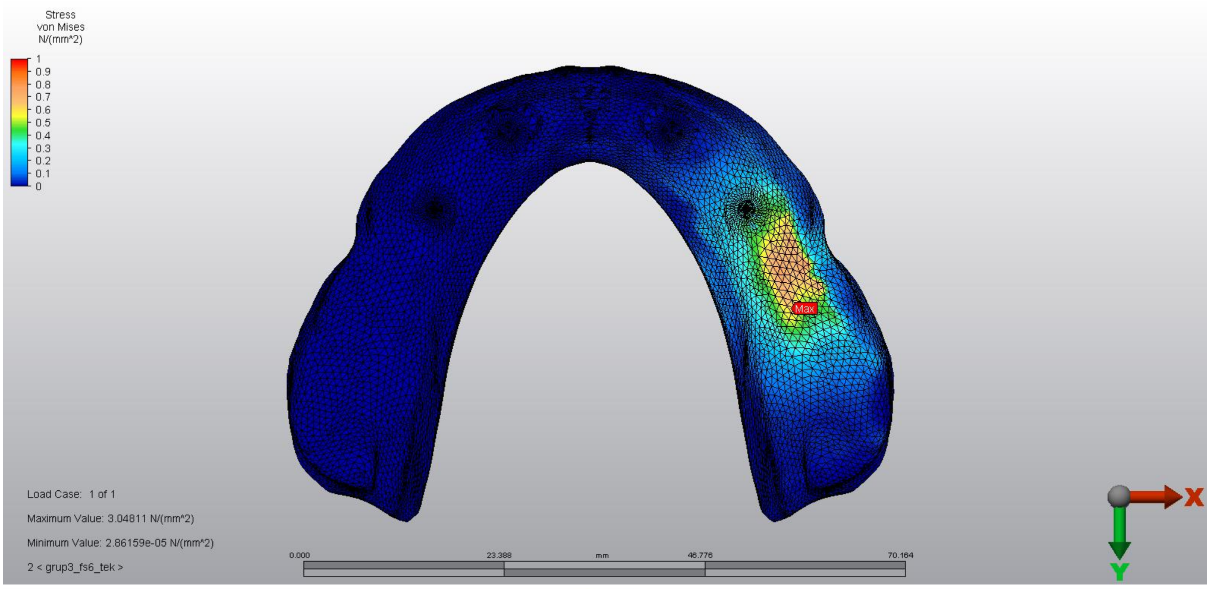
Stress
von Mises
N/(mm^2)
1
0.9
0.8
0.7
0.6
0.5
0.4
0.3
0.2
0.1
0
Max
Load Case: 1 of 1
Maximum Value: 3.04811 N/(mm^2)
Minimum Value: 2.86159e-05 N/(mm^2)
2 < grup3_fs6_tek >
0.000
23.388
mm
46.776
70.164
X
Y

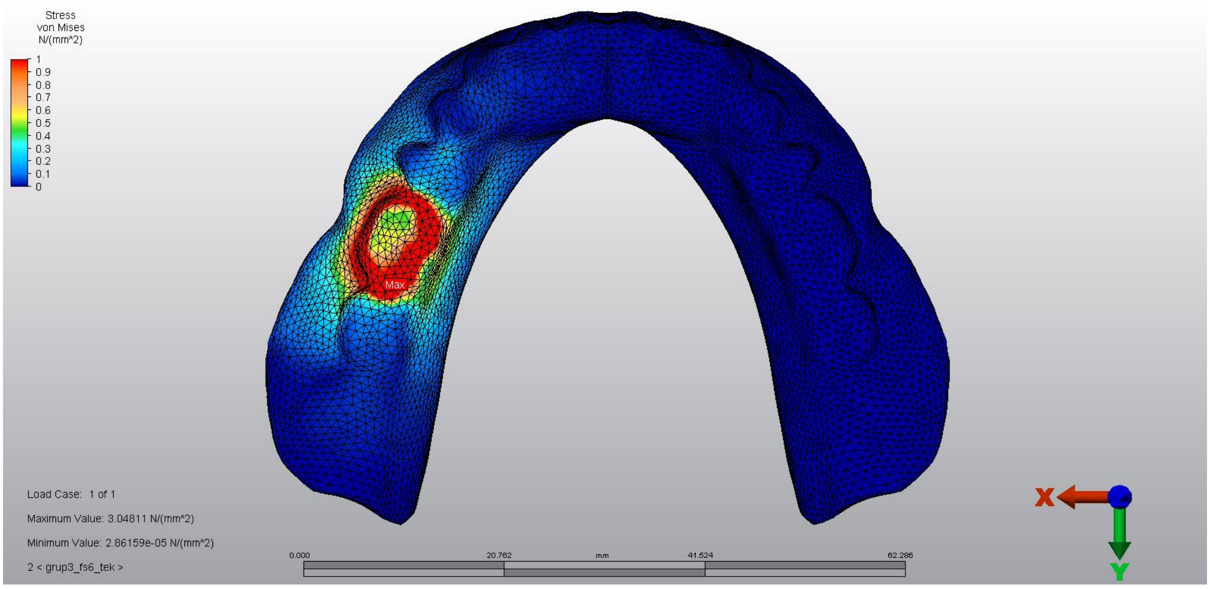
Stress
von Mises
N/(mm^2)
1
0.9
0.8
0.7
0.6
0.5
0.4
0.3
0.2
0.1
0
Max
Load Case: 1 of 1
Maximum Value: 3.04811 N/(mm^2)
Minimum Value: 2.86159e-05 N/(mm^2)
2 < grup3_fs6_tek >
0.000
20.762
mm
41.524
62.286
X
Y

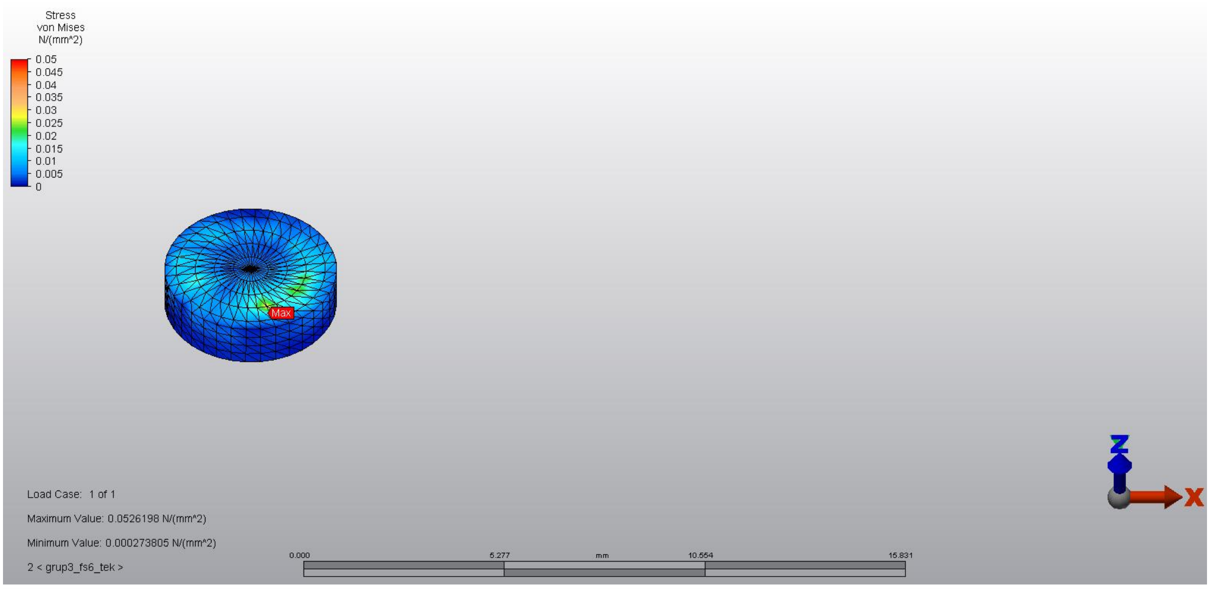
Stress
von Mises
N/(mm^2)
0.05
0.045
0.04
0.035
0.03
0.025
0.02
0.015
0.01
0.005
0
Max
Load Case: 1 of 1
Maximum Value: 0.0526198 N/(mm^2)
Minimum Value: 0.000273805 N/(mm^2)
2 < grup3_fs6_tek >
0.000
5.277
mm
10.554
15.831
Z
X

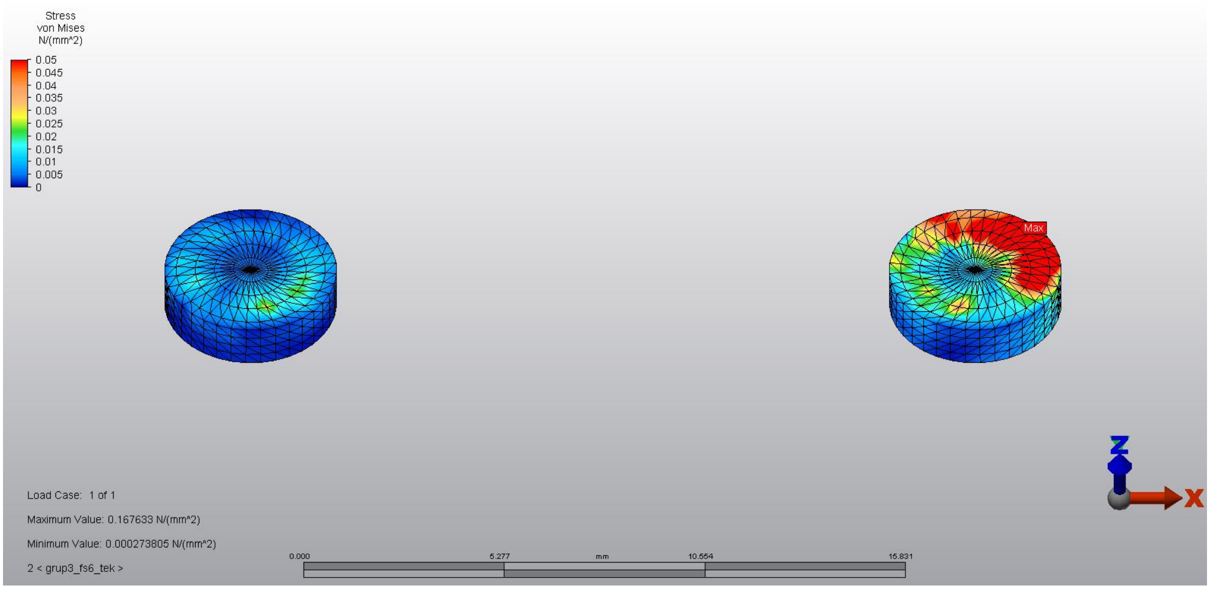
Stress
von Mises
N/(mm^2)
0.05
0.045
0.04
0.035
0.03
0.025
0.02
0.015
0.01
0.005
0
Max
Load Case: 1 of 1
Maximum Value: 0.167633 N/(mm^2)
Minimum Value: 0.000273805 N/(mm^2)
2 < grup3_fs6_tek >
0.000
5.277
mm
10.554
15.831
Z
X

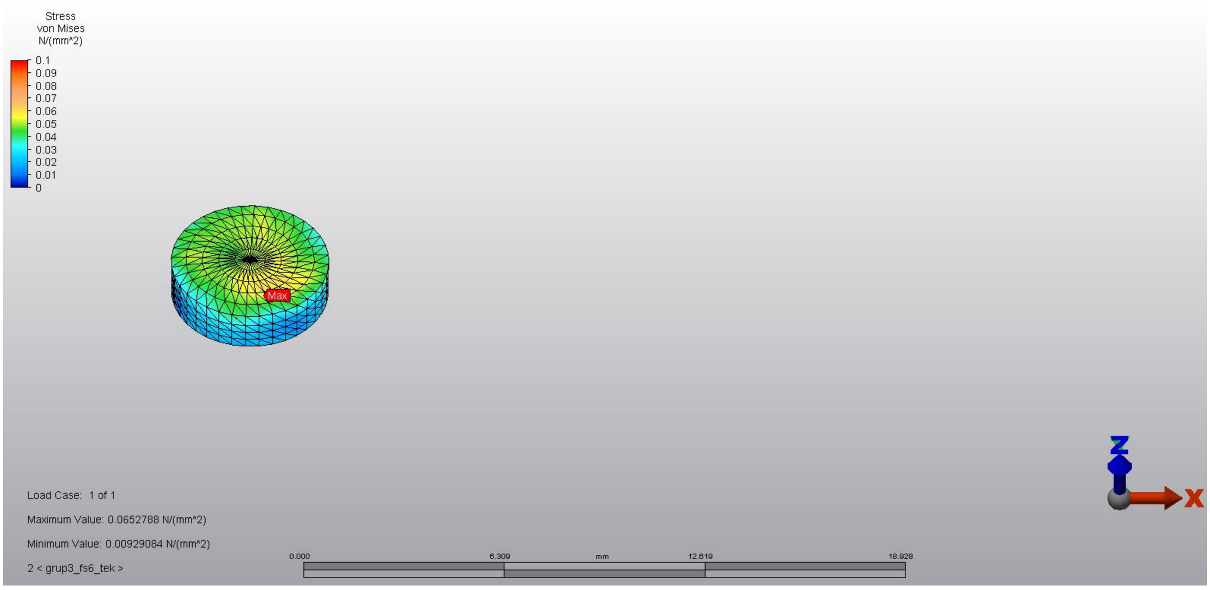
Stress
von Mises
N/(mm^2)
0.1
0.09
0.08
0.07
0.06
0.05
0.04
0.03
0.02
0.01
0
Max
Load Case: 1 of 1
Maximum Value: 0.0652788 N/(mm^2)
Minimum Value: 0.00929084 N/(mm^2)
2 < grup3_fs6_tek >
0.000
6.309
mm
12.619
18.928
Z
X

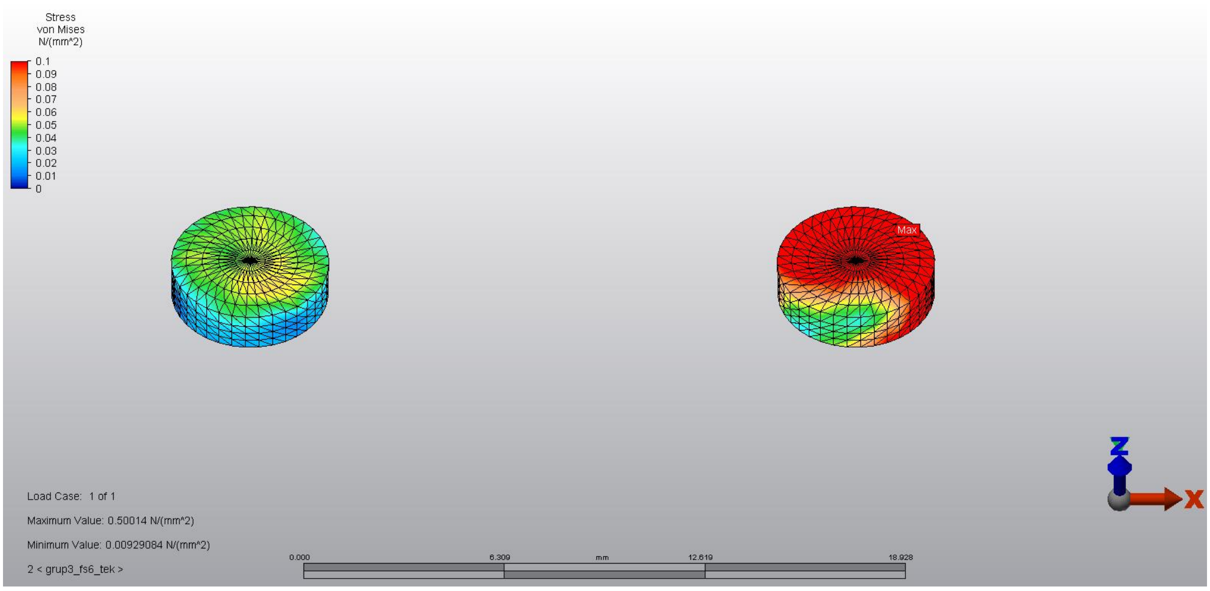
Stress
von Mises
N/(mm^2)
0.1
0.09
0.08
0.07
0.06
0.05
0.04
0.03
0.02
0.01
0
Max
Load Case: 1 of 1
Maximum Value: 0.50014 N/(mm^2)
Minimum Value: 0.00929084 N/(mm^2)
2 < grup3_fs6_tek >
0.000
6.309
mm
12.619
18.928
Z
X

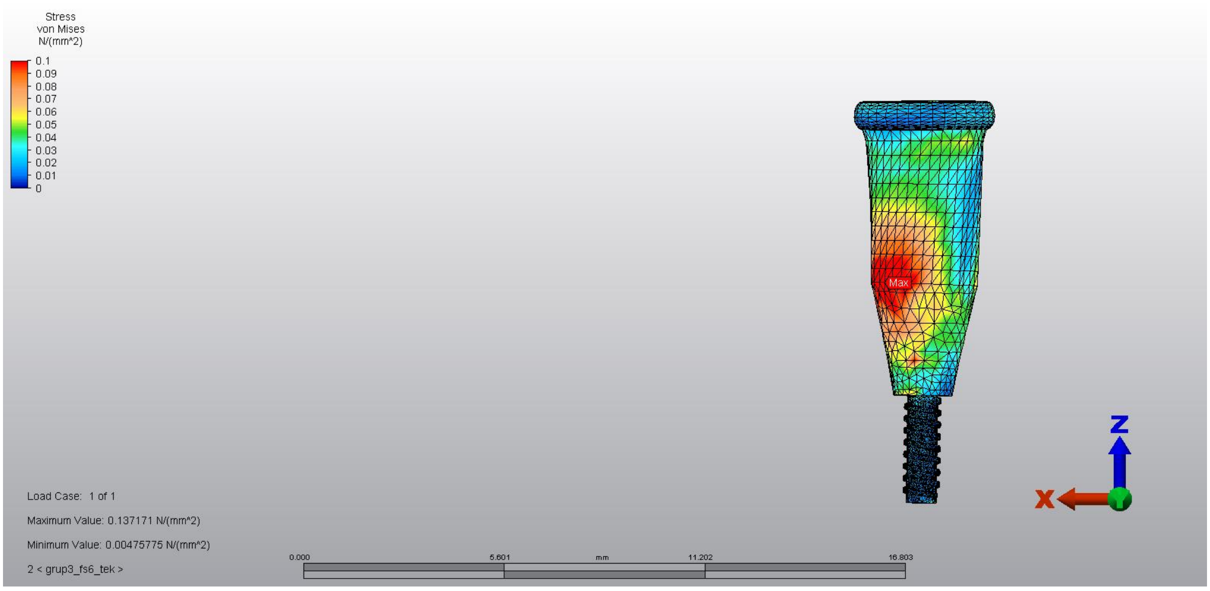
Stress
von Mises
N/(mm^2)
0.1
0.09
0.08
0.07
0.06
0.05
0.04
0.03
0.02
0.01
0
Max
Load Case: 1 of 1
Maximum Value: 0.137171 N/(mm^2)
Minimum Value: 0.00475775 N/(mm^2)
2 < grup3_fs6_tek >
0.000
5.601
mm
11.202
16.803
Z
X

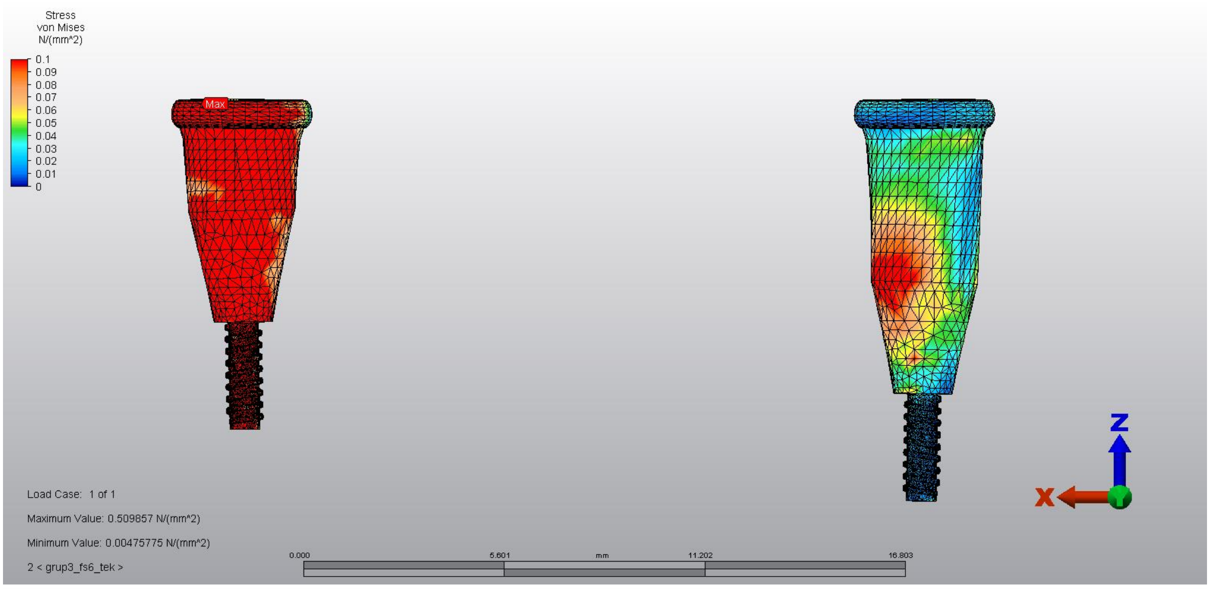

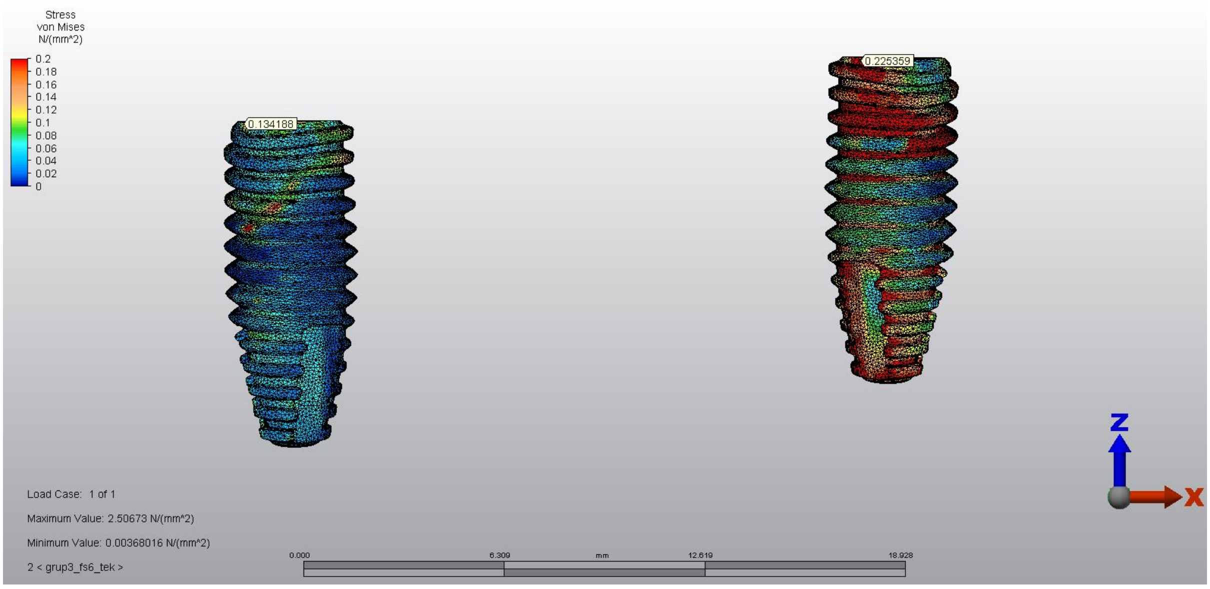

BILATERAL

Number of nodes = 144510

Number of elements = 725320

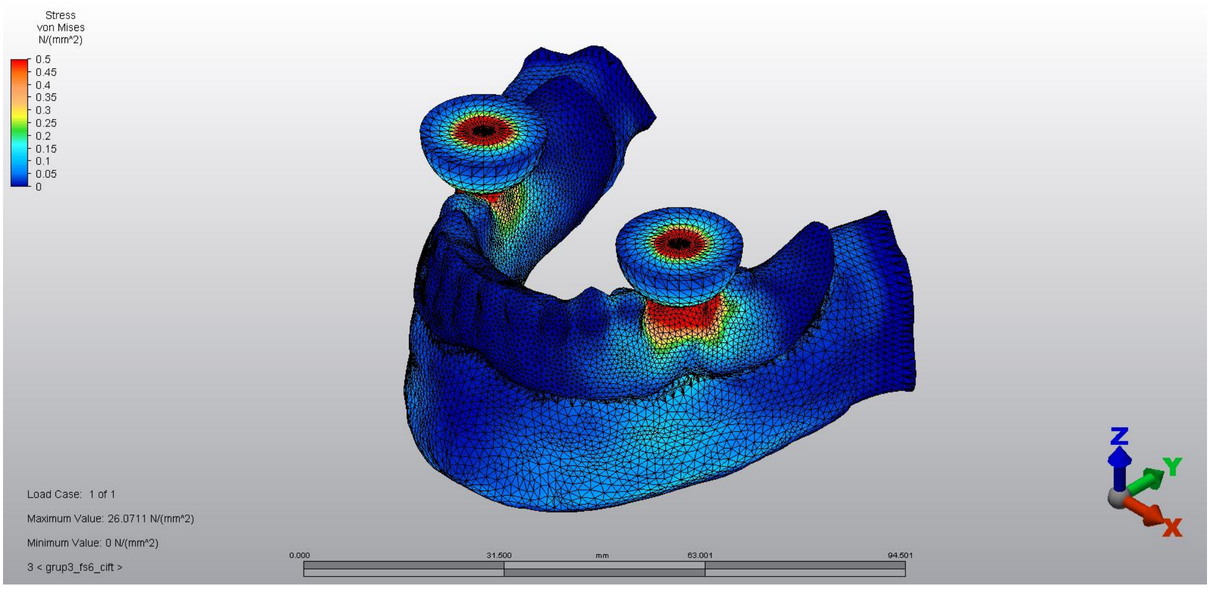
Stress
von Mises
N/(mm^2)
0.5
0.45
0.4
0.35
0.3
0.25
0.2
0.15
0.1
0.05
0
Load Case: 1 of 1
Maximum Value: 26.0711 N/(mm^2)
Minimum Value: 0 N/(mm^2)
3 < grup3_fs6_cift >
0.000
31.500
mm
63.001
94.501
Z
Y
X

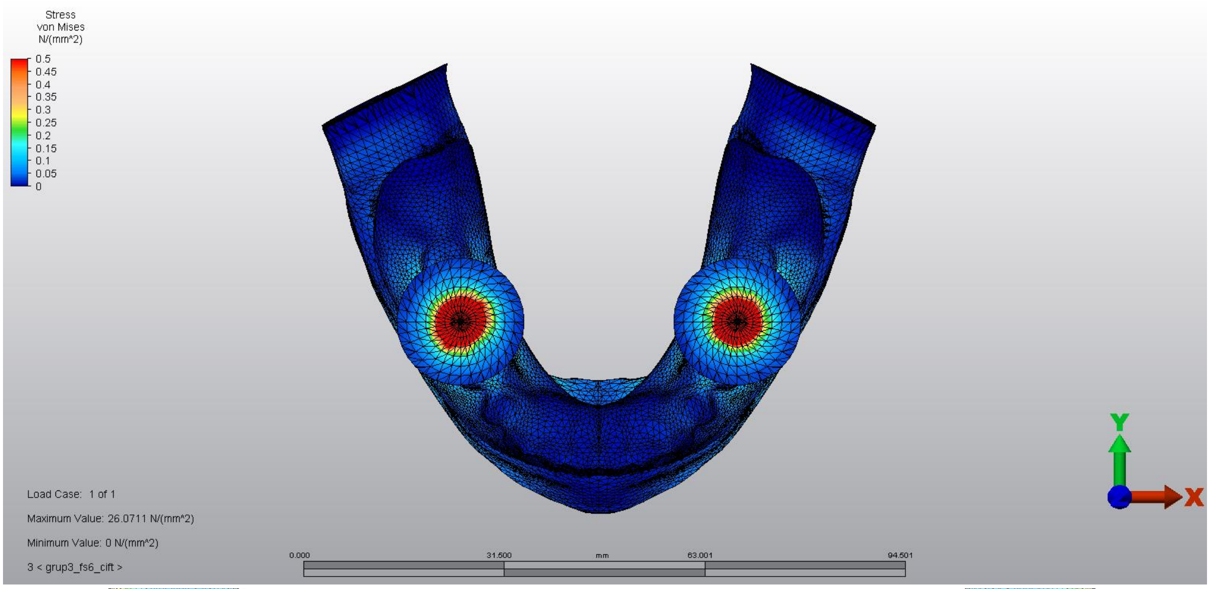
Stress
von Mises
N/(mm^2)
0.5
0.45
0.4
0.35
0.3
0.25
0.2
0.15
0.1
0.05
0
Load Case: 1 of 1
Maximum Value: 26.0711 N/(mm^2)
Minimum Value: 0 N/(mm^2)
3 < grup3_fs6_cift >
0.000
31.500
mm
63.001
94.501
Y
X

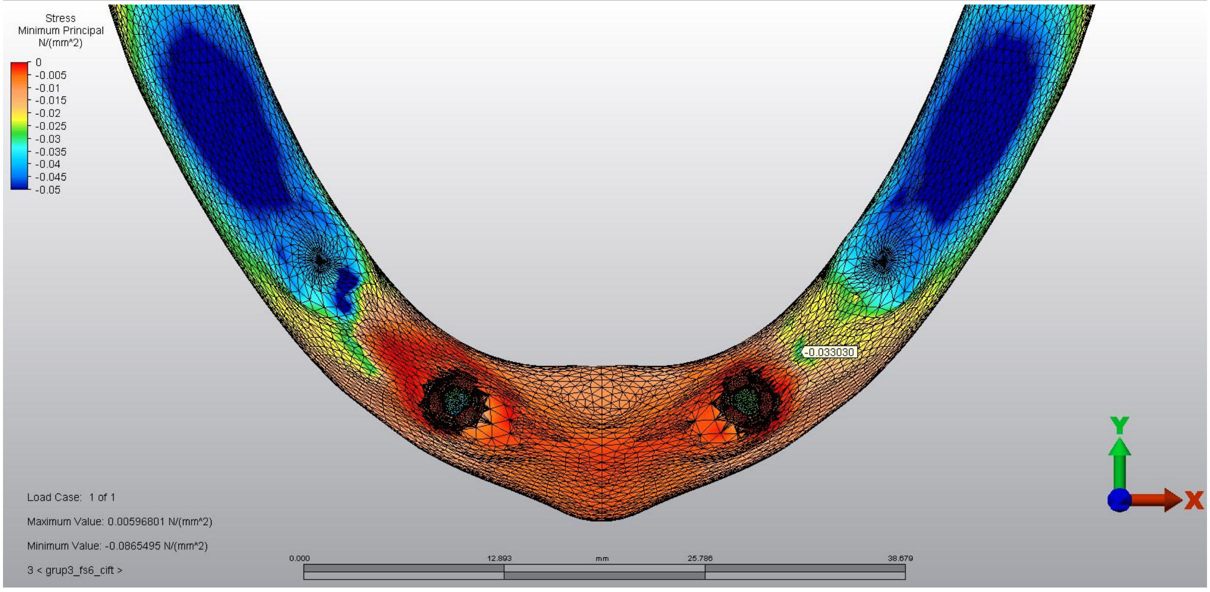
Stress
Minimum Principal
N/(mm^2)
0
-0.005
-0.01
-0.015
-0.02
-0.025
-0.03
-0.035
-0.04
-0.045
-0.05
-0.033030
Load Case: 1 of 1
Maximum Value: 0.00596801 N/(mm^2)
Minimum Value: -0.0865495 N/(mm^2)
3 < grup3_fs6_cift >
0.000
12.893
mm
25.786
38.679
Y
X

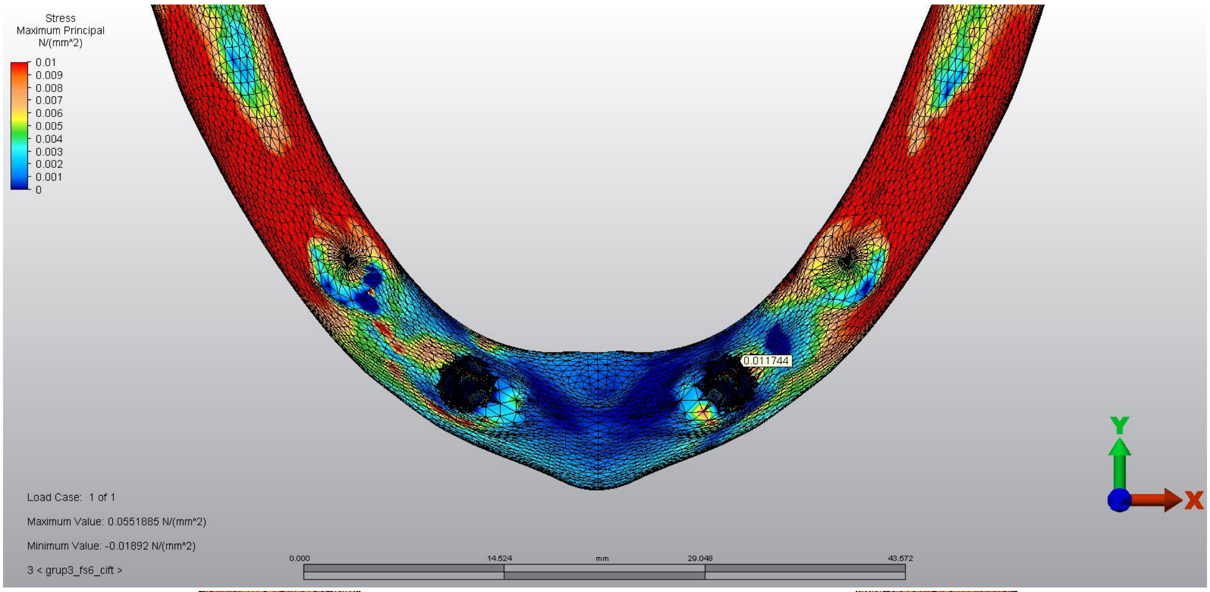
Stress
Maximum Principal
N/(mm^2)
0.01
0.009
0.008
0.007
0.006
0.005
0.004
0.003
0.002
0.001
0
0.011744
Load Case: 1 of 1
Maximum Value: 0.0551885 N/(mm^2)
Minimum Value: -0.01892 N/(mm^2)
3 < grup3_fs6_cift >
0.000
14.524
mm
29.048
43.572
Y
X

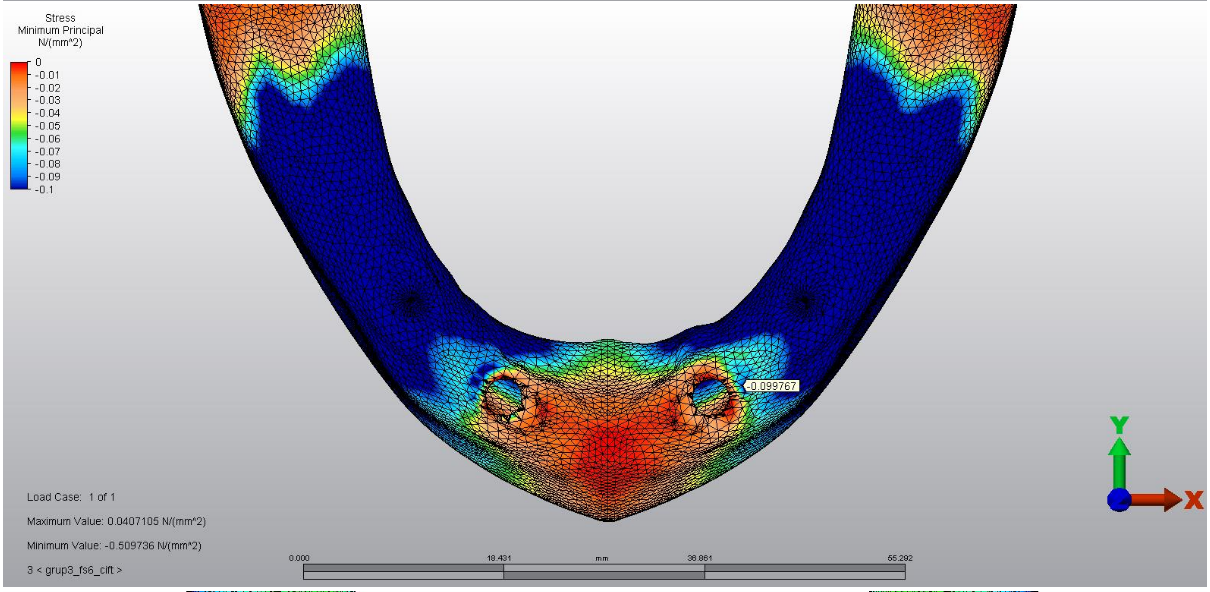
Stress
Minimum Principal
N/(mm^2)
0
-0.01
-0.02
-0.03
-0.04
-0.05
-0.06
-0.07
-0.08
-0.09
-0.1
-0.099767
Load Case: 1 of 1
Maximum Value: 0.0407105 N/(mm^2)
Minimum Value: -0.509736 N/(mm^2)
3 < grup3_fs6_cift >
0.000
18.431
mm
36.861
55.292
Y
X

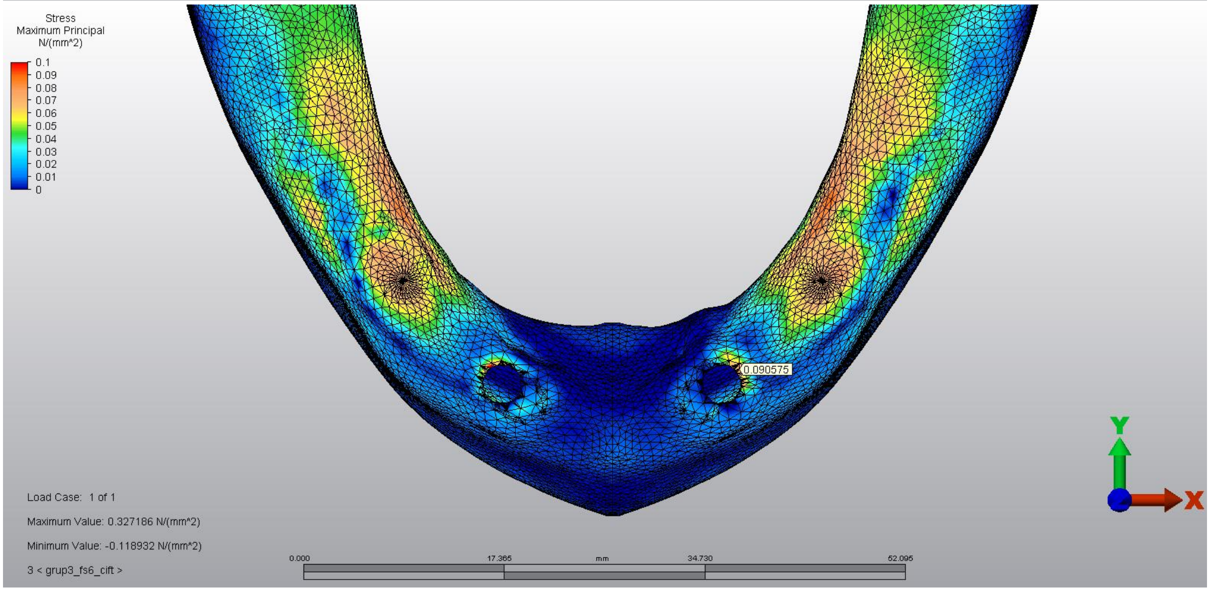
Stress
Maximum Principal
N/(mm^2)
0.1
0.09
0.08
0.07
0.06
0.05
0.04
0.03
0.02
0.01
0
0.090575
Load Case: 1 of 1
Maximum Value: 0.327186 N/(mm^2)
Minimum Value: -0.118932 N/(mm^2)
3 < grup3_fs6_cift >
0.000
17.365
mm
34.730
52.095
Y
X

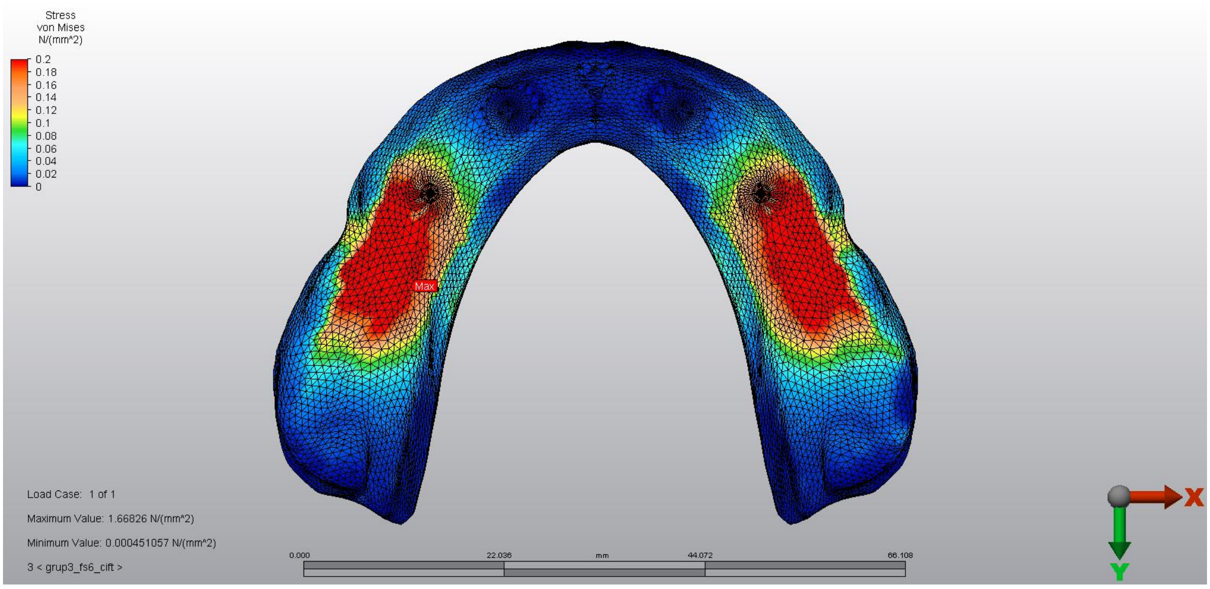
Stress
von Mises
N/(mm^2)
0.2
0.18
0.16
0.14
0.12
0.1
0.08
0.06
0.04
0.02
0
Max
Load Case: 1 of 1
Maximum Value: 1.66826 N/(mm^2)
Minimum Value: 0.000451057 N/(mm^2)
3 < grup3_fs6_cift >
0.000
22.036
mm
44.072
66.108
X
Y

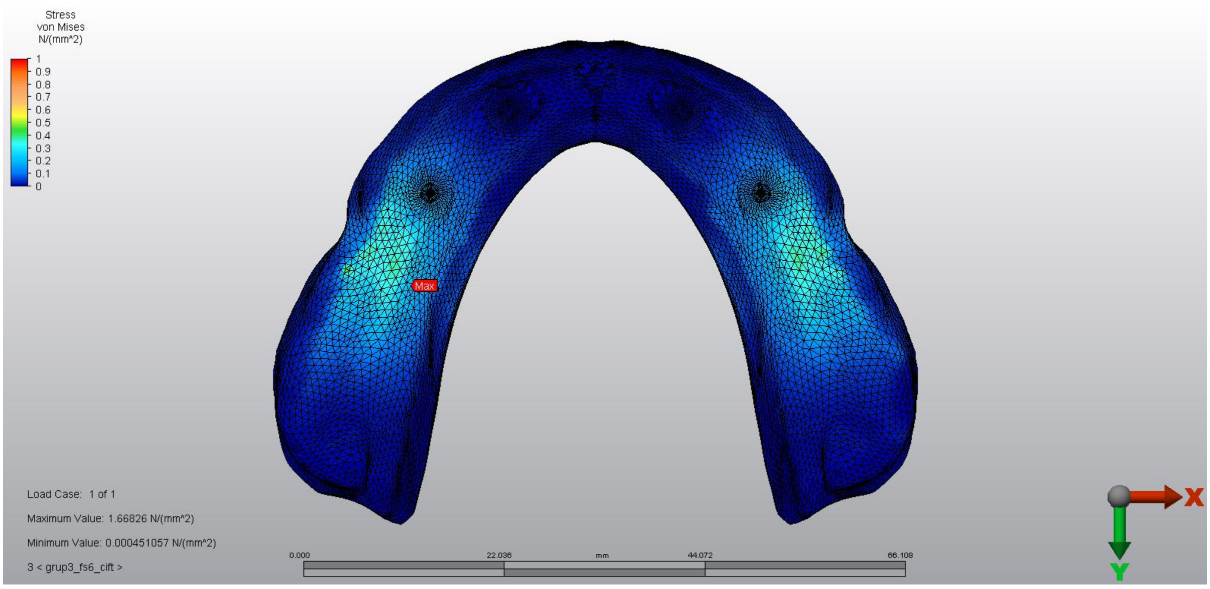
Stress
von Mises
N/(mm^2)
1
0.9
0.8
0.7
0.6
0.5
0.4
0.3
0.2
0.1
0
Max
Load Case: 1 of 1
Maximum Value: 1.66826 N/(mm^2)
Minimum Value: 0.000451057 N/(mm^2)
3 < grup3_fs6_cift >
0.000
22.036
mm
44.072
66.108
X
Y

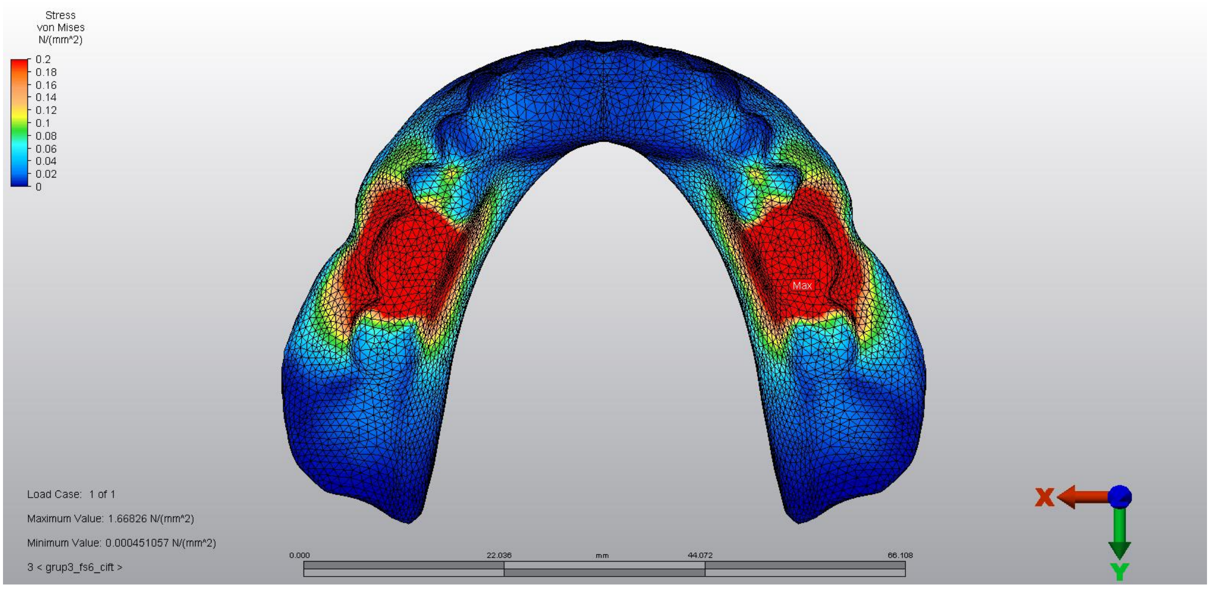
Stress
von Mises
N/(mm^2)
0.2
0.18
0.16
0.14
0.12
0.1
0.08
0.06
0.04
0.02
0
Max
Load Case: 1 of 1
Maximum Value: 1.66826 N/(mm^2)
Minimum Value: 0.000451057 N/(mm^2)
3 < grup3_fs6_cift >
0.000
22.036
mm
44.072
66.108
X
Y

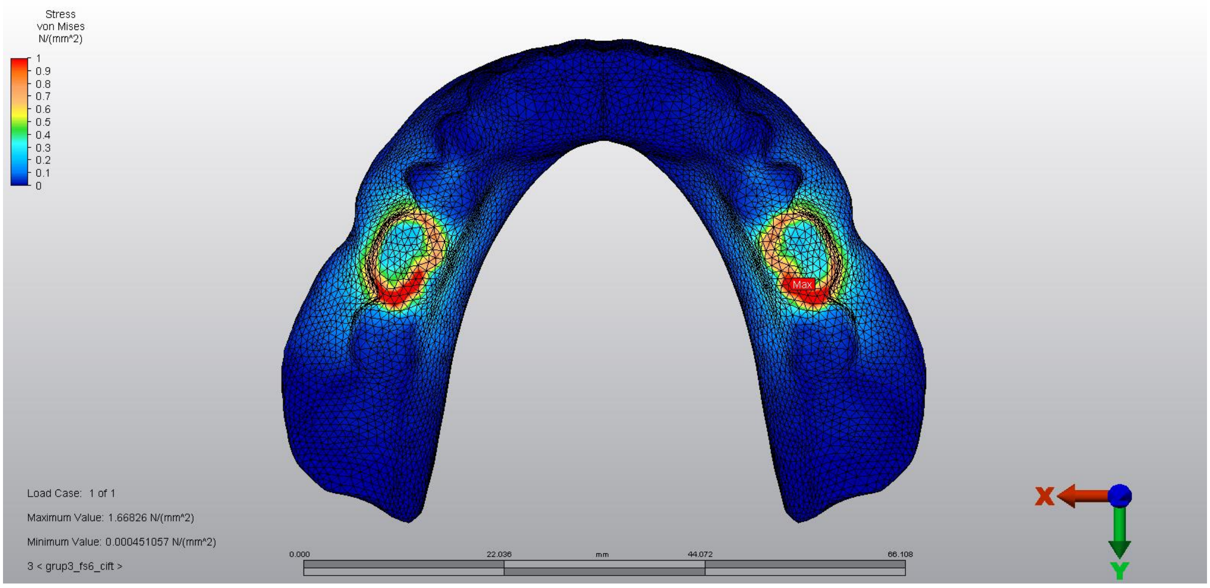
Stress
von Mises
N/(mm^2)
1
0.9
0.8
0.7
0.6
0.5
0.4
0.3
0.2
0.1
0
Max
Load Case: 1 of 1
Maximum Value: 1.66826 N/(mm^2)
Minimum Value: 0.000451057 N/(mm^2)
3 < grup3_fs6_cift >
0.000
22.036
mm
44.072
66.108
X
Y

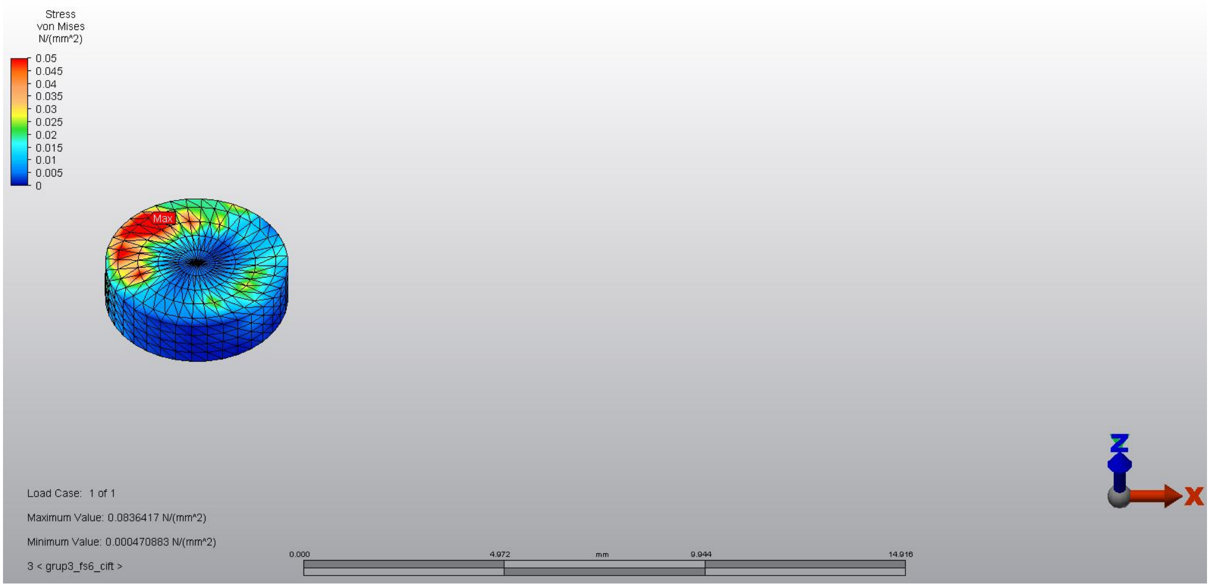
Stress
von Mises
N/(mm^2)
0.05
0.045
0.04
0.035
0.03
0.025
0.02
0.015
0.01
0.005
0
Max
Load Case: 1 of 1
Maximum Value: 0.0836417 N/(mm^2)
Minimum Value: 0.000470883 N/(mm^2)
3 < grup3_fs6_cift >
0.000
4.972
mm
9.944
14.916
Z
X

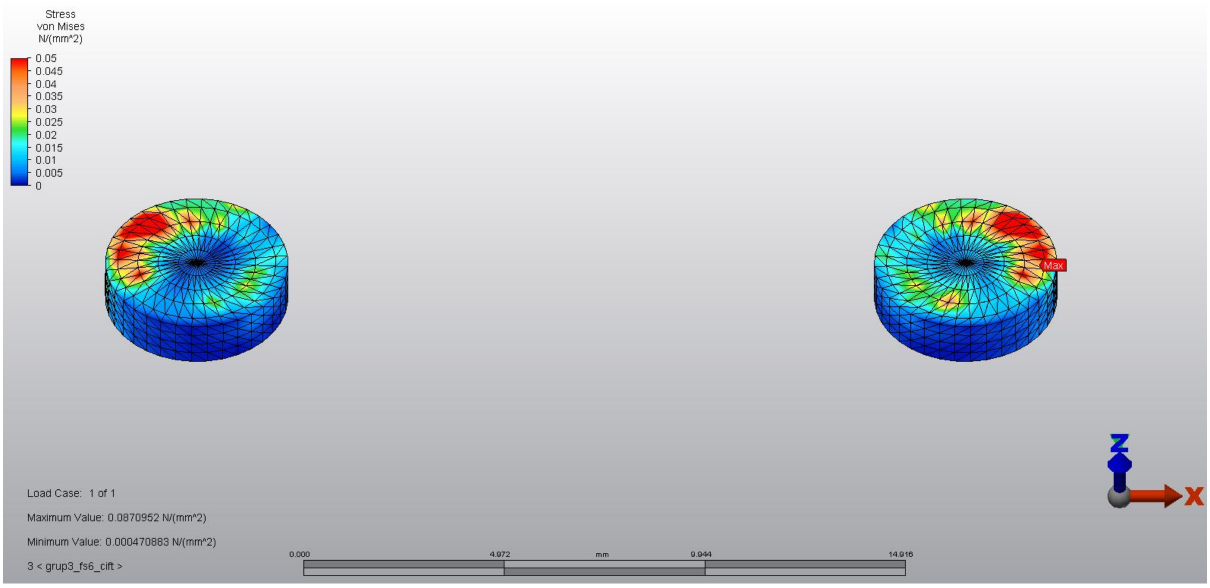
Stress
von Mises
N/(mm^2)
0.05
0.045
0.04
0.035
0.03
0.025
0.02
0.015
0.01
0.005
0
Max
Load Case: 1 of 1
Maximum Value: 0.0870952 N/(mm^2)
Minimum Value: 0.000470883 N/(mm^2)
3 < grup3_fs6_cift >
0.000
4.972
mm
9.944
14.916
Z
X

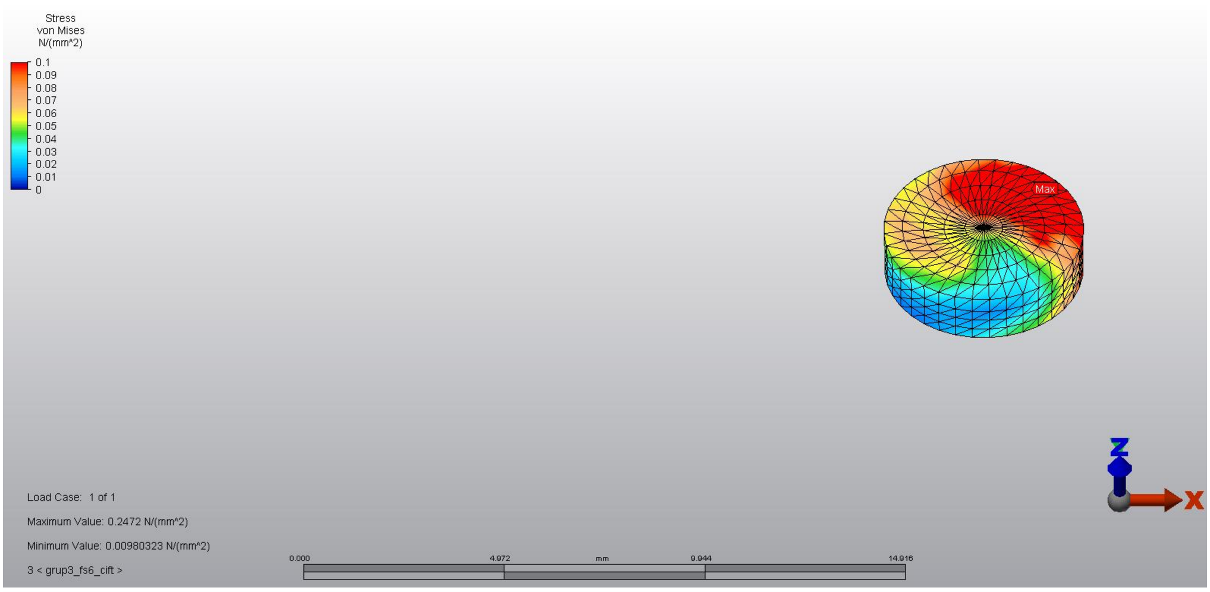
Stress
von Mises
N/(mm^2)
0.1
0.09
0.08
0.07
0.06
0.05
0.04
0.03
0.02
0.01
0
Max
Load Case: 1 of 1
Maximum Value: 0.2472 N/(mm^2)
Minimum Value: 0.00980323 N/(mm^2)
3 < grup3_fs6_cift >
0.000
4.972
mm
9.944
14.916
Z
X

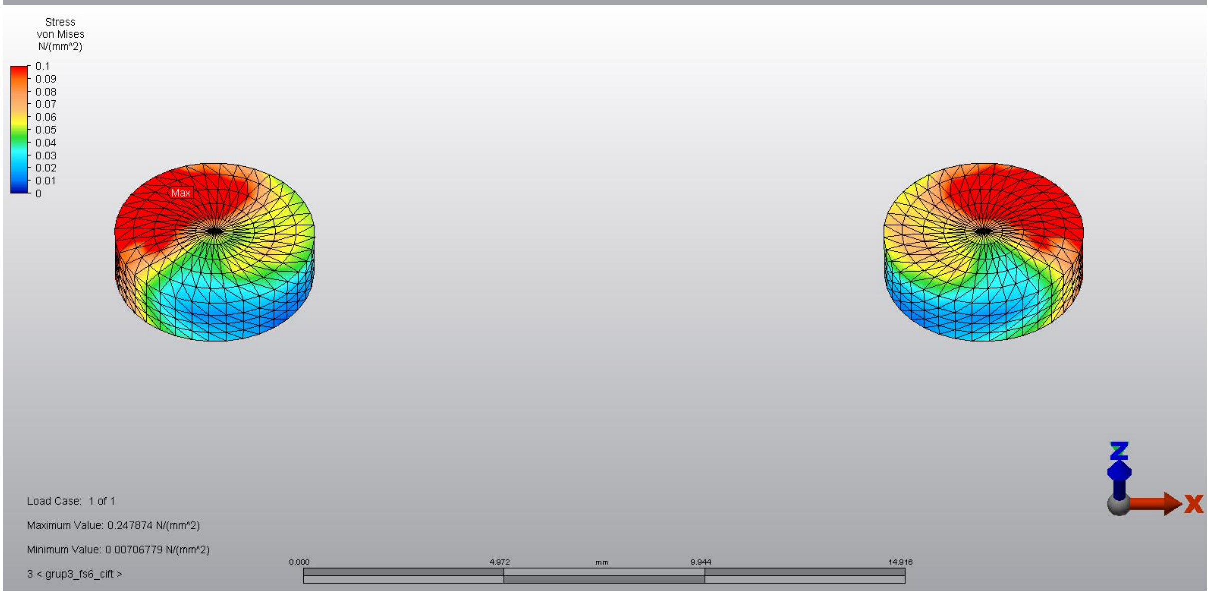
Stress
von Mises
N/(mm^2)
0.1
0.09
0.08
0.07
0.06
0.05
0.04
0.03
0.02
0.01
0
Max
Load Case: 1 of 1
Maximum Value: 0.247874 N/(mm^2)
Minimum Value: 0.00706779 N/(mm^2)
3 < grup3_fs6_cift >
0.000
4.972
mm
9.944
14.916
Z
X

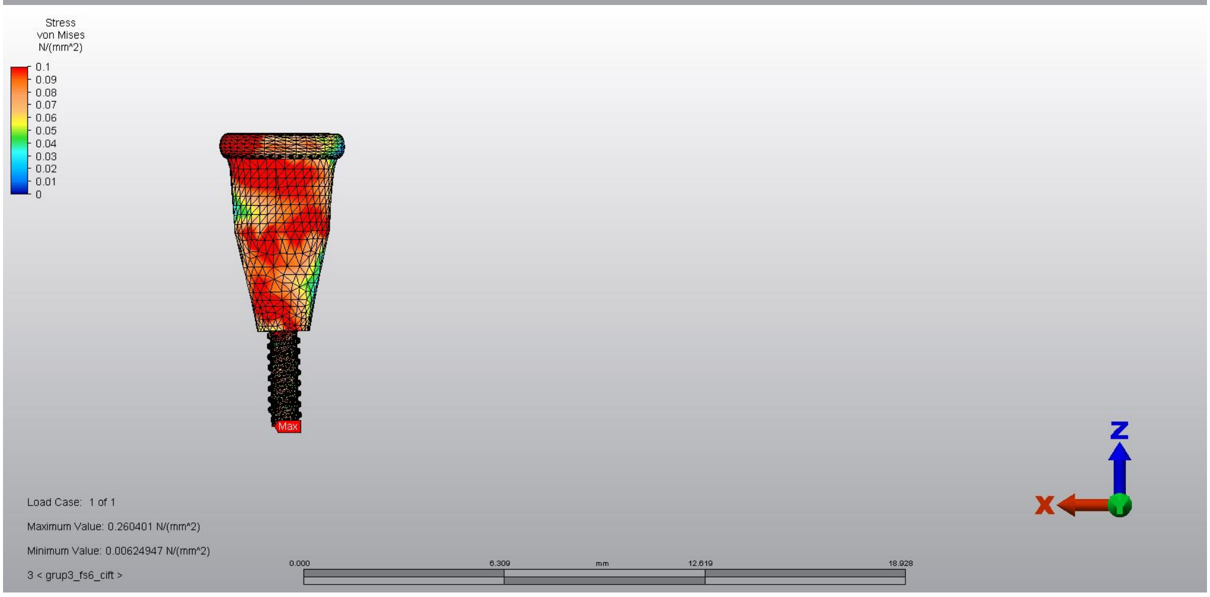
Stress
von Mises
N/(mm^2)
0.1
0.09
0.08
0.07
0.06
0.05
0.04
0.03
0.02
0.01
0
Max
Load Case: 1 of 1
Maximum Value: 0.260401 N/(mm^2)
Minimum Value: 0.00624947 N/(mm^2)
3 < grup3_fs6_cift >
0.000
6.309
mm
12.619
18.928
Z
X

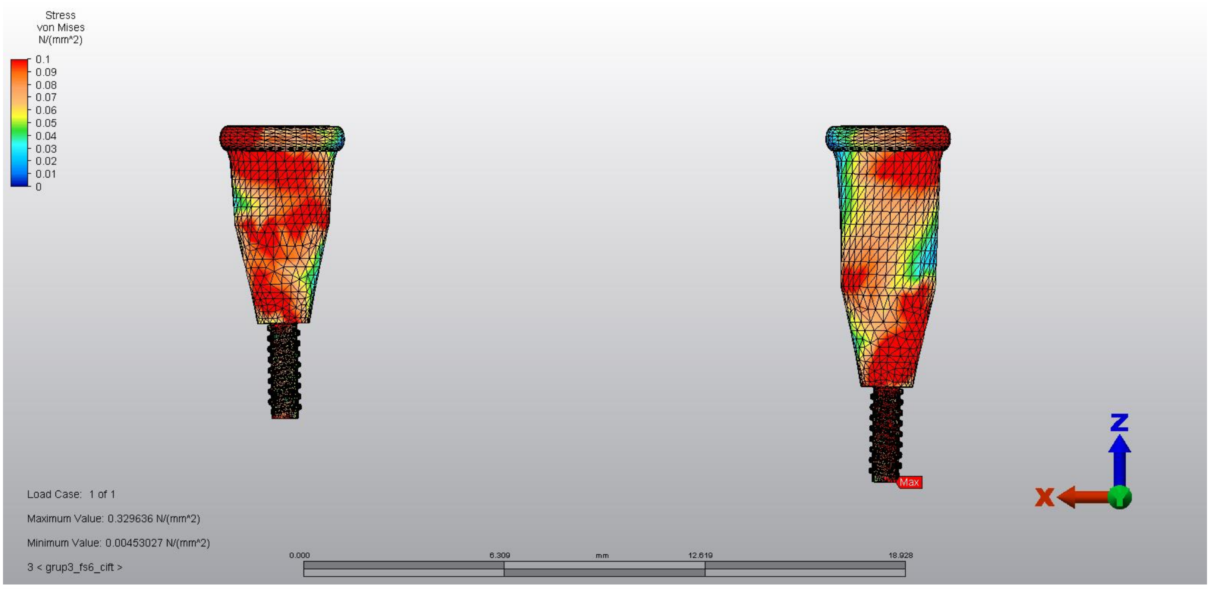
Stress
von Mises
N/(mm^2)
0.1
0.09
0.08
0.07
0.06
0.05
0.04
0.03
0.02
0.01
0
Max
Z
X
Load Case: 1 of 1
Maximum Value: 0.329636 N/(mm^2)
Minimum Value: 0.00453027 N/(mm^2)
3 < grup3_fs6_cift >
0.000
6.309
mm
12.619
18.928

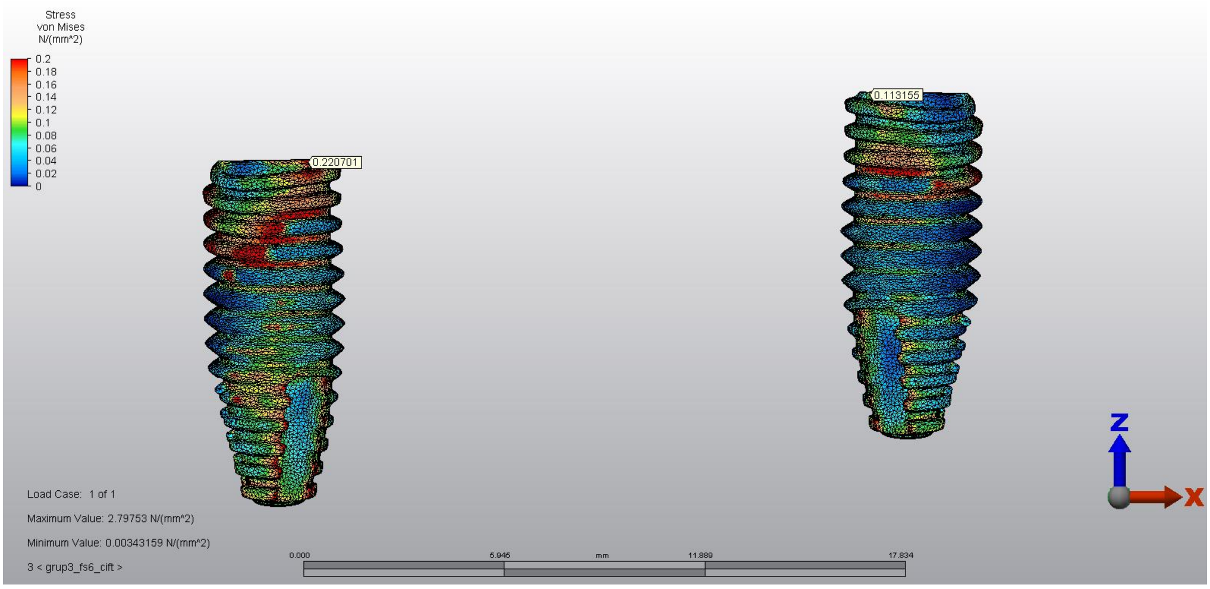
Stress
von Mises
N/(mm^2)
0.2
0.18
0.16
0.14
0.12
0.1
0.08
0.06
0.04
0.02
0
0.220701
0.113155
Z
X
Load Case: 1 of 1
Maximum Value: 2.79753 N/(mm^2)
Minimum Value: 0.00343159 N/(mm^2)
3 < grup3_fs6_cift >
0.000
5.945
mm
11.889
17.834
